# Chemoprevention for malaria with monthly intermittent preventive treatment with dihydroartemisinin–piperaquine in pregnant women living with HIV on daily co-trimoxazole in Kenya and Malawi: a randomised, double-blind, placebo-controlled trial

**DOI:** 10.1016/S0140-6736(23)02631-4

**Published:** 2024-01-12

**Authors:** Hellen C Barsosio, Mwayiwawo Madanitsa, Everlyne D Ondieki, James Dodd, Eric D Onyango, Kephas Otieno, Duolao Wang, Jenny Hill, Victor Mwapasa, Kamija S Phiri, Kenneth Maleta, Miriam Taegtmeyer, Simon Kariuki, Christentze Schmiegelow, Julie R Gutman, Feiko O ter Kuile

**Affiliations:** Kenya Medical Research Institute, Centre for Global Health Research, Kisumu, Kenya; Department of Clinical Sciences, Liverpool School of Tropical Medicine, Liverpool, UK; School of Global and Public Health, Kamuzu University of Health Sciences, Blantyre, Malawi; Academy of Medical Sciences, Malawi University of Science and Technology, Thyolo, Malawi; Kenya Medical Research Institute, Centre for Global Health Research, Kisumu, Kenya; Department of Clinical Sciences, Liverpool School of Tropical Medicine, Liverpool, UK; Kenya Medical Research Institute, Centre for Global Health Research, Kisumu, Kenya; Kenya Medical Research Institute, Centre for Global Health Research, Kisumu, Kenya; Department of Clinical Sciences, Liverpool School of Tropical Medicine, Liverpool, UK; Department of Clinical Sciences, Liverpool School of Tropical Medicine, Liverpool, UK; School of Global and Public Health, Kamuzu University of Health Sciences, Blantyre, Malawi; School of Global and Public Health, Kamuzu University of Health Sciences, Blantyre, Malawi; School of Global and Public Health, Kamuzu University of Health Sciences, Blantyre, Malawi; Department of Clinical Sciences, Liverpool School of Tropical Medicine, Liverpool, UK; Kenya Medical Research Institute, Centre for Global Health Research, Kisumu, Kenya; Department of Clinical Sciences, Liverpool School of Tropical Medicine, Liverpool, UK; Centre for Medical Parasitology, Department of Immunology and Microbiology, University of Copenhagen, Copenhagen, Denmark; Department of Gynaecology and Obstetrics, Copenhagen University Hospital – North Zealand, Hillerød, Denmark; Malaria Branch, Division of Parasitic Diseases and Malaria, National Center for Emerging and Zoonotic Infectious Diseases, Centers for Disease Control and Prevention, Atlanta, GA, USA; Kenya Medical Research Institute, Centre for Global Health Research, Kisumu, Kenya; Department of Clinical Sciences, Liverpool School of Tropical Medicine, Liverpool, UK

## Abstract

**Background:**

The efficacy of daily co-trimoxazole, an antifolate used for malaria chemoprevention in pregnant women living with HIV, is threatened by cross-resistance of *Plasmodium falciparum* to the antifolate sulfadoxine–pyrimethamine. We assessed whether addition of monthly dihydroartemisinin–piperaquine to daily co-trimoxazole is more effective at preventing malaria infection than monthly placebo plus daily co-trimoxazole in pregnant women living with HIV.

**Methods:**

We did an individually randomised, two-arm, placebo-controlled trial in areas with high-grade sulfadoxine–pyrimethamine resistance in Kenya and Malawi. Pregnant women living with HIV on dolutegravir-based combination antiretroviral therapy (cART) who had singleton pregnancies between 16 weeks’ and 28 weeks’ gestation were randomly assigned (1:1) by computer-generated block randomisation, stratified by site and HIV status (known positive *vs* newly diagnosed), to daily co-trimoxazole plus monthly dihydroartemisinin–piperaquine (three tablets of 40 mg dihydroartemisinin and 320 mg piperaquine given daily for 3 days) or daily co-trimoxazole plus monthly placebo. Daily co-trimoxazole consisted of one tablet of 160 mg sulfamethoxazole and 800 mg trimethoprim. The primary endpoint was the incidence of *Plasmodium* infection detected in the peripheral (maternal) or placental (maternal) blood or tissue by PCR, microscopy, rapid diagnostic test, or placental histology (active infection) from 2 weeks after the first dose of dihydroartemisinin–piperaquine or placebo to delivery. Log-binomial regression was used for binary outcomes, and Poisson regression for count outcomes. The primary analysis was by modified intention to treat, consisting of all randomised eligible participants with primary endpoint data. The safety analysis included all women who received at least one dose of study drug. All investigators, laboratory staff, data analysts, and participants were masked to treatment assignment. This trial is registered with ClinicalTrials.gov, NCT04158713.

**Findings:**

From Nov 11, 2019, to Aug 3, 2021, 904 women were enrolled and randomly assigned to co-trimoxazole plus dihydroartemisinin–piperaquine (n=448) or co-trimoxazole plus placebo (n=456), of whom 895 (99%) contributed to the primary analysis (co-trimoxazole plus dihydroartemisinin–piperaquine, n=443; co-trimoxazole plus placebo, n=452). The cumulative risk of any malaria infection during pregnancy or delivery was lower in the co-trimoxazole plus dihydroartemisinin–piperaquine group than in the co-trimoxazole plus placebo group (31 [7%] of 443 women *vs* 70 [15%] of 452 women, risk ratio 0·45, 95% CI 0·30–0·67; p=0·0001). The incidence of any malaria infection during pregnancy or delivery was 25·4 per 100 person-years in the cotrimoxazole plus dihydroartemisinin–piperaquine group versus 77·3 per 100 person-years in the co-trimoxazole plus placebo group (incidence rate ratio 0·32, 95% CI 0·22–0·47, p<0·0001). The number needed to treat to avert one malaria infection per pregnancy was 7 (95% CI 5–10). The incidence of serious adverse events was similar between groups in mothers (17·7 per 100 person-years in the cotrimoxazole plus dihydroartemisinin–piperaquine group [23 events] *vs* 17·8 per 100 person-years in the co-trimoxazole group [25 events]) and infants (45·4 per 100 person-years [23 events] *vs* 40·2 per 100 person-years [21 events]). Nausea within the first 4 days after the start of treatment was reported by 29 (7%) of 446 women in the co-trimoxazole plus dihydroartemisinin–piperaquine group versus 12 (3%) of 445 women in the co-trimoxazole plus placebo group. The risk of adverse pregnancy outcomes did not differ between groups.

**Interpretation:**

Addition of monthly intermittent preventive treatment with dihydroartemisinin–piperaquine to the standard of care with daily unsupervised co-trimoxazole in areas of high antifolate resistance substantially improves malaria chemoprevention in pregnant women living with HIV on dolutegravir-based cART and should be considered for policy.

**Funding:**

European and Developing Countries Clinical Trials Partnership 2; UK Joint Global Health Trials Scheme (UK Foreign, Commonwealth and Development Office; Medical Research Council; National Institute for Health Research; Wellcome); and Swedish International Development Cooperation Agency.

## Introduction

Co-infection with *Plasmodium falciparum* and HIV increases the risk of adverse maternal and fetal outcomes in women living with HIV of all gravidae.^1^ In malariaendemic areas, WHO recommends malaria chemoprevention with monthly sulfadoxine–pyrimethamine in pregnant women without HIV and daily co-trimoxazole in women living with HIV. Co-trimoxazole, a fixed-dose combination of trimethoprim plus sulfamethoxazole, is used to prevent opportunistic infections in people living with HIV and has antimalarial properties. Currently, high-level resistance of *Plasmodium falciparum* to sulfadoxine–pyrimethamine in east and southern Africa threatens the antimalarial efficacy of daily co-trimoxazole because both are sulfa-based antifolate drugs with similar antimalarial modes of action.^2^ Trials in women without HIV found that several long-acting alternatives to sulfadoxine–pyrimethamine were unsuitable as intermittent preventive treatment in pregnancy (IPTp) because of poor tolerability, including amodiaquine (alone or combined with sulfadoxine–pyrimethamine), mefloquine, and chloroquine (alone or combined with azithromycin).^3^ Dihydroartemisinin–piperaquine, a long-acting artemisinin-based combination therapy, is the only antimalarial drug to have shown promise to replace sulfadoxine–pyrimethamine as IPTp in women without HIV.^3^

Fewer trials of IPTp have been done in women living with HIV. Two trials that added IPTp with mefloquine to daily co-trimoxazole in areas with high sulfadoxine–pyrimethamine resistance in women living with HIV on nevirapine-based or efavirenz-based combined antiretroviral therapy (cART) showed the superiority of the combination in reducing clinical malaria and placental malaria compared with daily co-trimoxazole alone.^4,5^ However, IPTp with mefloquine was poorly tolerated^5,6^ and was associated with increased viral load and mother-to-child transmission of HIV,^5^ possibly reflecting a drug–drug interaction between mefloquine and nevirapine.^7^ IPTp with dihydroartemisinin–piperaquine has only been assessed in a small trial of 200 pregnant women living with HIV receiving efavirenz-based cART in Uganda.^8^ The study was inconclusive. The combination of daily co-trimoxazole plus monthly dihydroartemisinin–piperaquine was well tolerated, but no reduction in *Plasmodium* infections was achieved, likely because of reduced malaria transmission in the study area after the introduction of indoor residual spraying,^8^ and a clinically relevant drug–drug interaction between dihydroartemisinin–piperaquine and efavirenz resulting in a 27% reduction in exposure to dihydroartemisinin and 38% reduction in exposure to piperaquine.^9^ A modelling analysis suggested that less than 1% of women living with HIV on efavirenz-based cART receiving monthly IPTp with dihydroartemisinin–piperaquine would achieve protective piperaquine concentrations, defined as maintaining concentrations of more than 10·3 ng/mL for more than 95% of the time,^10^ a threshold previously associated with 95% protection against malaria in women without HIV.^11^

Following an update of WHO’s policy in 2018, many national HIV control programmes in Africa, including Kenya and Malawi, transitioned their first-line cART, including for pregnant women, from efavirenz-based to dolutegravir-based regimens. Unlike efavirenz, no clinically relevant drug–drug interactions have been seen between dihydroartemisinin–piperaquine and dolutegravir in pregnancy.^12^

We present the results of a trial designed to determine whether addition of monthly IPTp with dihydroartemisinin–piperaquine to daily co-trimoxazole is more effective at preventing malaria than the standard of care with daily co-trimoxazole alone in women receiving dolutegravir-based cART in areas with high sulfadoxine–pyrimethamine resistance.

## Methods

### Study design and participants

We did a two-arm multicentre, individually randomised placebo-controlled trial in six antenatal clinics in western Kenya (n=3) and Malawi (n=3) in areas with high-grade sulfadoxine–pyrimethamine resistance and perennial malaria transmission. Eligible women were those living with HIV, eligible for (or on) daily cART consisting of tenofovir, lamivudine, and dolutegravir, had ultrasound-confirmed viable singleton pregnancies between 16 weeks’ and 28 weeks’ gestation, were residents of the study area, and were willing to adhere to scheduled and unscheduled study visit procedures and deliver in a study clinic. Women with multiple pregnancies (eg, twin pregnancies), known heart conditions, advanced HIV disease at WHO clinical stages 3 and 4, confirmed or suspected tuberculosis disease, known allergy or contraindication to dihydroartemisinin–piperaquine, or HIV-negative or unknown HIV status were excluded. All participants provided written informed consent. Ethics committees of the Kenya Medical Research Institute (KEMRI), the College of Medicine in Malawi, and the Liverpool School of Tropical Medicine approved the study. The US Centers for Disease Control and Prevention approved the protocol through a reliance agreement with KEMRI. The protocol is available in the appendix (pp 22–132).

### Randomisation and masking

Balanced randomisation was done using computer-generated permuted block randomisation stratified by site and HIV status (women diagnosed with HIV before enrolment as documented in their existing health records [ie, known positive] *vs* newly diagnosed). An independent statistician not involved in the study generated the randomisation list for the trial pharmacists in Kenya and Malawi, who prepared sequentially numbered, sealed, opaque envelopes for each participant with the randomisation assignments. Contained in each opaque envelope were the pre-packed investigational products for the entire study duration for that participant.

In each study site, these opaque envelopes were opened sequentially upon enrolment of a study participant by the study staff responsible for participant enrolment. The eligible participants were allocated in the order of their study identification number by drawing the next sequentially numbered sealed envelope. The two study groups were daily co-trimoxazole combined with monthly placebo, and daily co-trimoxazole combined with monthly IPTp with active dihydroartemisinin–piperaquine. Placebo tablets had the same appearance as active dihydroartemisinin–piperaquine tablets.

The statistician in Liverpool also prepared another computer-generated list for each group and study site for the day of a single unannounced home visit to assess tolerance and adherence to the study drugs on days 2 or 3 after the first dose of one of the monthly IPTp courses, chosen randomly. The information for these unannounced home visits was included in the same sealed envelopes as the study allocation.

All investigators, laboratory staff, data analysts, and participants were masked to treatment assignment.

### Procedures

At enrolment, sociodemographic information and data on insecticide-treated net use and HIV care and treatment were collected. Participants had their medical and obstetric history recorded and underwent a clinical examination that included screening for tuberculosis and opportunistic infections, assessment of WHO HIV disease clinical stage, maternal weight, height, and mid-upper arm circumference assessment, and an ultrasound scan to assess gestational age. Routine urinalysis and routine point-of-care tests were done, including for HIV, syphilis, and anaemia. All participants received a long-lasting insecticide-treated net as part of routine antenatal care.

Eligible women with HIV on, or eligible for, daily cART consisting of tenofovir, lamivudine, and dolutegravir were enrolled. cART was provided as part of routine care at the prevention of mother-to-child transmission of HIV (PMTCT) clinics in the study facilities. In Malawi, the national roll-out of cART containing tenofovir, lamivudine, and dolutegravir in women of reproductive age started before the study; thus, all enrolled participants with known positive HIV status were already on this cART regimen. In Kenya, all pregnant women with newly diagnosed HIV were started on a tenofovir, lamivudine, and dolutegravir regimen from the second trimester onwards per national guidelines. Known HIV-positive pregnant women on an efavirenz-based cART regimen were switched to tenofovir, lamivudine, and dolutegravir at PMTCT clinics if they were in their second trimester and were adequately virally suppressed with viral loads up to 400 copies per mL per national guidelines.

Daily co-trimoxazole consisted of one double-strength tablet of 160 mg sulfamethoxazole and 800 mg trimethoprim (Sulfran-DS, Universal Corporation, Nairobi, Kenya). Each monthly dihydroartemisinin–piperaquine course consisted of a fixed dose of three tablets of 40 mg dihydroartemisinin and 320 mg piperaquine (D-Artepp, Fosun Pharma, Shanghai, China) given daily for 3 days until delivery. Placebo was also provided by Fosun Pharma (appendix p 2). The first cotrimoxazole, dihydroartemisinin–piperaquine, and placebo doses were given in the study clinic under direct observation, combined with a slice of dry bread or a biscuit. Subsequent doses were provided to the participant to self-administer at home. Adherence and tolerance were assessed for dihydroartemisinin–piperaquine and placebo by telephone calls on the second and third days of the course and during unannounced home visits. Adherence to daily co-trimoxazole was not assessed.

Malaria screening at enrolment using malaria rapid diagnostic tests (histidine-rich protein 2 or *Plasmodium* lactate dehydrogenase) was done for all women in Kenya and symptomatic women in Malawi as per national guidelines (appendix p 2). In both countries, symptomatic women with either documented fever or a history of fever were tested for malaria at scheduled and unscheduled visits. Test-positive women were given a standard 3-day treatment course with artemether–lumefantrine, and the next course of dihydroartemisinin–piperaquine or placebo was delayed until the next scheduled visit (median 18 days, IQR 9–24, range 3–28). All women continued using daily co-trimoxazole throughout.

Participants attended scheduled study clinics every 4 weeks with unscheduled visits in between if women were unwell or concerned about their pregnancy. A history of recent illness and any medication use was taken at each visit, clinical and obstetric examinations were done, and a blood sample was taken for malaria microscopy and PCR. Additionally, haemoglobin concentrations (Hemocue, HemoCue, Ängelholm, Sweden) were determined at around 35–38 weeks’ gestation. Routine urinalysis was done as part of routine care for symptomatic women at any visit.

At delivery, blood samples for the same malaria metrics and placental and cord blood samples for placental histology, malaria rapid diagnostic tests, microscopy, and PCR were done (appendix pp 6–7). Newborn assessments, including anthropometry and screening for congenital anomalies and jaundice, were done at delivery, day 7, and the final visit at 6–8 weeks, coinciding with childhood immunisation. PMTCT clinics initiated infant cART prophylaxis at delivery or at first contact with the mother and infant after delivery as per national guidelines. Infant cART prophylaxis included 6 weeks of zidovudine and nevirapine, followed by 6 weeks of daily nevirapine. In febrile or symptomatic infants, heel prick samples were taken for malaria metrics. Infant samples for HIV DNA PCR were collected at PMTCT clinics at the final visit, 6–8 weeks postpartum, and sent to Ministry of Health laboratories as part of routine HIV care.

Three sets of electrocardiograms were done in a subgroup of women to assess the heart-rate corrected QT intervals (QTc) before the first daily dose of dihydroartemisinin–piperaquine or placebo and 4 hours after the third daily dose at enrolment and twice more during pregnancy approximately 1–2 months and 3–4 months later (appendix p 3). Adverse events and serious adverse events were assessed during pregnancy and up to 6 weeks postpartum and graded using standardised severity criteria. Events were coded using Medical Dictionary for Regulatory Activities (MedDRA) coding. Tolerability was assessed by comparing the rates of vomiting within 30 min of drug intake in the clinic and other adverse events at each scheduled monthly visit (all women) or at home visits scheduled to take place on days 2 or 3 after the start of a monthly course of IPTp (random subgroup).

### Outcomes

The primary endpoint was the incidence of at least one *Plasmodium* infection detected in the peripheral (maternal) or placental (maternal) blood or tissue by PCR, microscopy, rapid diagnostic test, or placental histology (active infection) from 2 weeks after the first day of the first dose of the first course of dihydroartemisinin–piperaquine or placebo to delivery inclusive. Key secondary efficacy endpoints included the individual components of the primary endpoint, clinical malaria, maternal haemoglobin concentrations and anaemia (haemoglobin <11 g/dL, <9 g/dL, or <7 g/dL) measured in the third trimester and at delivery, maternal weight gain and mid-upper arm circumference measured at each scheduled monthly visit, and adverse pregnancy outcome, defined as a composite of either fetal loss (miscarriage or stillbirth), small vulnerable newborn (singleton livebirth born small for gestational age [less than tenth centile of the INTERGROWTH reference population, appendix p 5], or with low birthweight [<2500 g], or preterm [<37 weeks’ gestation]), or subsequent neonatal death by day 28, and the individual components of the composite adverse pregnancy outcome. Other maternal secondary outcomes included placental inflammation and chorioamnionitis, and antibodies against SARS-CoV-2 at birth. Other newborn secondary outcomes included birth length for gestational age and stunting (less than third centile of birth length for gestational age), and birthweight for gestational age and wasting (less than third centile of birthweight for gestational age), congenital malaria (malaria infection detected at birth or within the first week of life), cord blood haemoglobin concentration and congenital anaemia (haemoglobin <12·5 g/dL), early neonatal death (<7 days), and perinatal death (stillbirth or early neonatal death). All Z scores were obtained using INTERGROWTH reference populations. See appendix (pp 4–6) for a complete list of all endpoints and definitions. All outcome assessors were masked to the treatment groups.

### Statistical analysis

The study was designed to achieve 80% power to detect a 50% reduction in the cumulative incidence of *Plasmodium* infection from 12% in the co-trimoxazole plus placebo group to 6% in the co-trimoxazole plus dihydroartemisinin–piperaquine group (risk ratio [RR] 0·50, two-sided α=0·05), which required 898 participants (449 per group), allowing for 20% loss to follow-up. Log-binomial regression was used for dichotomous endpoints to obtain RRs and corresponding 95% CIs and modified Poisson regression in case of non-convergence (appendix p 7). Linear regression was used for continuous outcomes, and results were expressed as mean difference and 95% CIs. Poisson regression with a log-link function and follow-up time as an offset was used for count variables to obtain incidence rate ratios (IRRs) and 95% CIs and incidence rate difference. The number needed to treat to avert one event was calculated as the inverse of the incidence rate difference per 15·5 weeks (the average duration of follow-up; appendix p 7). The unadjusted (crude) analysis was the primary analysis and included the stratification factors of study site and HIV status (known positive and newly diagnosed) in all models. Secondary, covariate-adjusted analyses were done using several other prespecified baseline covariates in addition to HIV status and site, including gravidity, malaria status, socioeconomic status, season (average rainfall in the last 6 months before delivery), and malaria transmission intensity by study site (based on the prevalence of malaria at enrolment, continuous; appendix p 7). Subgroup analyses included baseline covariates and gestational age at enrolment, country, and number of IPTp courses received (appendix p 8). Post-hoc analyses assessed mean haemoglobin concentration at delivery or in the third trimester (haemoglobin at delivery was used if assessed before the onset of birth, and otherwise the haemoglobin measurement from the third trimester was used). Missing covariates were imputed using simple imputation. A two-sided p value less than 0·05 was used to define statistical significance. P values and the widths of the CIs for the primary and secondary endpoints have not been adjusted for multiplicity, so the values should not be used to infer definitive treatment effects. The modified intention-to-treat (ITT) population (ie, all randomly assigned eligible participants with endpoint data) was used for primary or secondary analyses. A sensitivity analysis was done using non-responder imputation^13^ to assess the impact of attrition bias. The per-protocol population included participants who attended every scheduled visit, took all scheduled IPTp courses, did not use prohibited medication, and contributed to the endpoint. For the safety analysis, women were included if they received at least one dose of study drug. All analyses were prespecified (unless otherwise indicated as post hoc) in a statistical analysis plan approved by the data and safety monitoring board. Statistical analyses were done with Stata version 17. This trial is registered with ClinicalTrials.gov, NCT04158713.

### Role of the funding source

The funders of the study had no role in study design, data collection, data analysis, data interpretation, or writing of the report.

## Results

**Figure 1:**
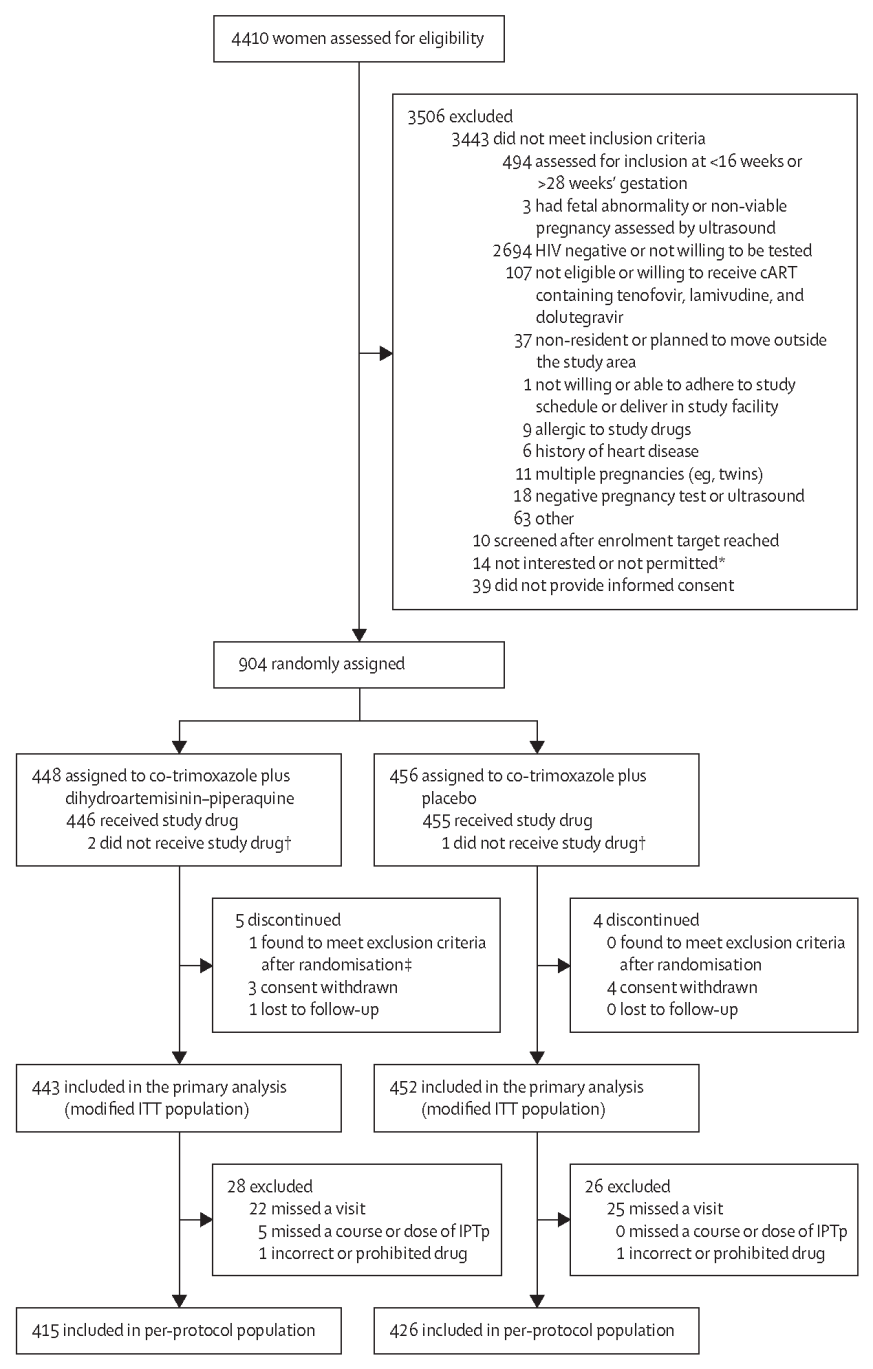
Trial profile cART=combination antiretroviral therapy. IPTp=intermittent preventive treatment in pregnancy. ITT=intention to treat. *Full eligibility criteria could not be assessed in these women, who either expressed hesitation in joining the study or whose partner, spouse, or another family member discouraged them from joining the research study. †Study drug refers to receipt of at least one course of intermittent preventive treatment with dihydroartemisinin–piperaquine or placebo. Three participants (co-trimoxazole plus dihydroartemisinin–piperaquine, n=2; co-trimoxazole plus placebo, n=1) did not receive study drug because they had malaria at enrolment and were treated with artemether–lumefantrine and were lost to follow-up before their first course of study drug scheduled 1 month later. ‡The participant was randomly assigned to co-trimoxazole plus dihydroartemisinin–piperaquine but later found to meet the exclusion criteria (twin pregnancy). Because the participant received the study drug, she is included in the safety population but not in the modified ITT population or per-protocol population.

From Nov 11, 2019, to Aug 3, 2021, 4410 women were screened for inclusion. Recruitment was stopped when 904 women had been randomly assigned (figure 1). Baseline characteristics of study participants are shown in table 1 and the appendix (p 10). Of the 904 participants, 152 (17%) were newly diagnosed with HIV infection and started on dolutegravir-based cART. 752 (83%) had known HIV infection, of whom 415 (55%) were already on dolutegravir-based cART, and 337 (45%) were switched from efavirenz-based to dolutegravir-based cART. At baseline, 151 (17%) of 904 women were infected with malaria (any diagnostic test; 75 [17%] of 448 women in the co-trimoxazole plus dihydroartemisinin–piperaquine group *vs* 76 [17%] of 456 women in the co-trimoxazole plus placebo group).

Of the 904 participants enrolled, one was found ineligible because of a twin pregnancy and excluded from the modified ITT population. 895 (99%) participants contributed to the primary endpoint analysis (figure 1). The proportion of eligible participants with missing primary endpoint data was equally distributed across study groups (appendix p 12).

The median follow-up time until delivery was 108 days (IQR 86–133; co-trimoxazole plus dihydroartemisinin–piperaquine group, 108 days [85–132]; co-trimoxazole plus placebo group, 110 days [87–135]). The median number of IPTp courses was 5 (IQR 4–6) in each group (appendix p 11). 4242 (97%) of the 4386 planned, scheduled antenatal visits were attended (2100 [97%] of 2173 in the cotrimoxazole plus dihydroartemisinin–piperaquine group *vs* 2142 [97%] of 2213 in the co-trimoxazole plus placebo group), and 722 (80%) of 903 women had a home visit to assess adherence (357 [80%] of 447 women in the cotrimoxazole plus dihydroartemisinin–piperaquine group *vs* 365 [80%] of 456 women in the co-trimoxazole plus placebo group). All women visited at home during random spot checks said they adhered to the correct number of dihydroartemisinin–piperaquine or placebo tablets (appendix p 11).

**Figure 2:**
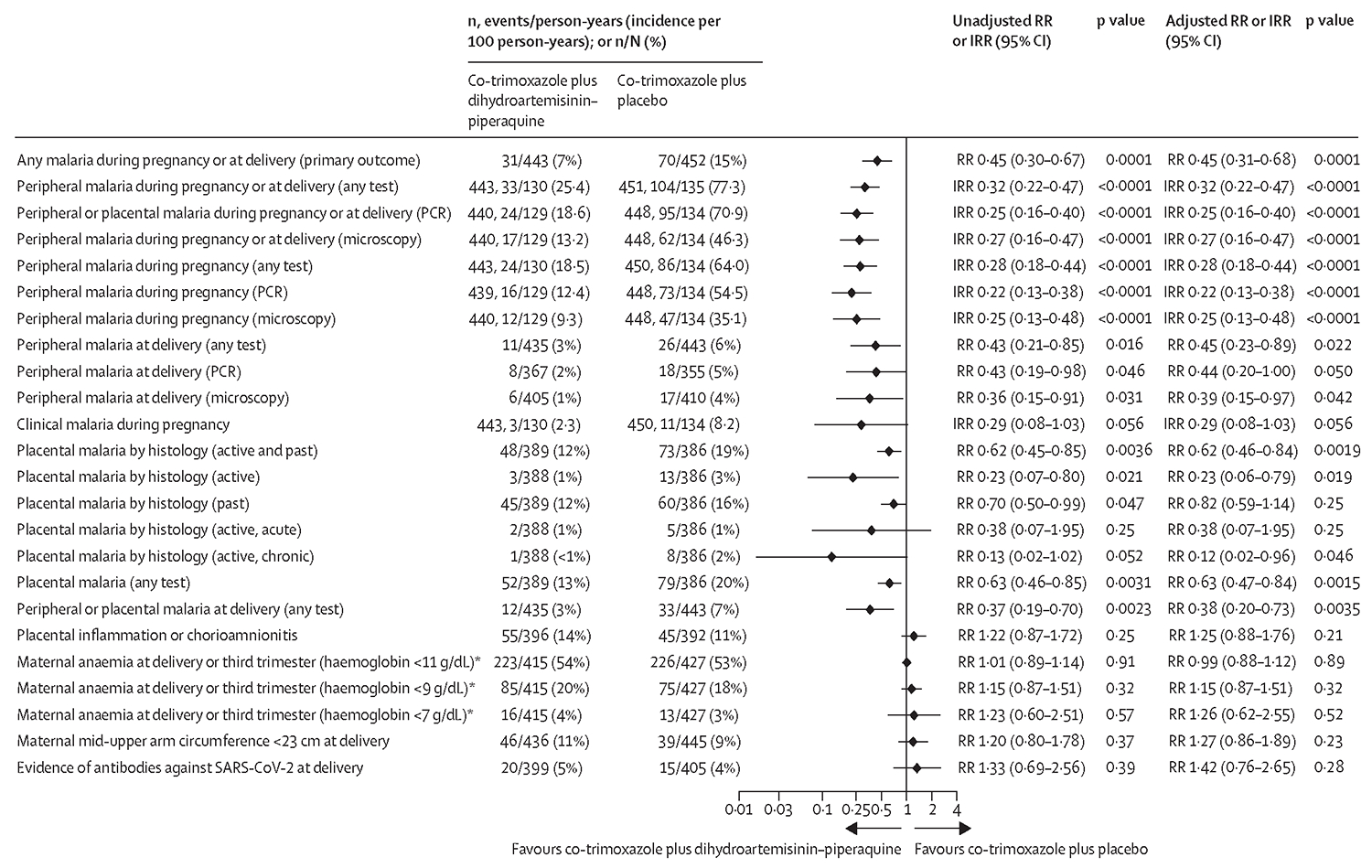
Primary endpoint and other secondary maternal endpoints (modified intention-to-treat population) The unadjusted RR and IRR values were obtained from log-binomial regression models (RR) and Poisson regression models (IRR) with the stratification factors of site and HIV status (known positive *vs* newly diagnosed HIV infection) included as covariates. The adjusted analyses were done using several other prespecified baseline covariates in addition to HIV status and site, including gravidity, malaria status, socioeconomic status, season (average rainfall in the last 6 months before delivery), and malaria transmission intensity by study site (based on the prevalence of malaria at enrolment, continuous). The term malaria refers to malaria infection. Clinical malaria refers to malaria infection detected by malaria rapid diagnostic test (histidine-rich protein 2 or *Plasmodium* lactate dehydrogenase) or microscopy in conjunction with documented fever (>37·5°C) or reported fever in the past 48 h. IRR=incidence rate ratio. RR=risk ratio. *Haemoglobin at delivery, or otherwise in the third trimester if the measurement at delivery was unavailable.

The cumulative risk of any malaria infection detected by any diagnostic test during pregnancy or delivery was lower in the co-trimoxazole plus dihydroartemisinin–piperaquine group than in the co-trimoxazole plus placebo group (31 [7%] of 443 women in the co-trimoxazole plus dihydroartemisinin–piperaquine group *vs* 70 [15%] of 452 women in the co-trimoxazole plus placebo group; RR 0·45, 95% CI 0·30–0·67; p=0·0001; figure 2). The total number of episodes of malaria infection detected in the peripheral blood, expressed as incidence per 100 person-years, was also lower in the co-trimoxazole plus dihydroartemisinin–piperaquine group than in the co-trimoxazole plus placebo group (25·4 per 100 person-years *vs* 77·3 per 100 person-years; IRR 0·32, 95% CI 0·22–0·47, p<0·0001). The number needed to treat to avert one malaria infection was 7 (95% CI 5–10). Similar results were obtained from the prespecified covariate-adjusted analyses (figure 2), per-protocol population analyses (appendix p 18), and a sensitivity analysis using non-responder imputation with missing endpoint data imputed (appendix p 19). Subgroup analyses showed that the effect was evident in both countries, all seasons, moderate and high transmission areas, in paucigravidae (primigravidae and secundigravidae) and multigravidae, in known HIV-positive women and newly diagnosed HIV-positive women, women enrolled before 21 weeks’ gestation or at 21 weeks’ gestation or later, and in those with or without malaria infection at enrolment. There was no evidence for a dose response by the number of courses received (appendix p 20).

During pregnancy, incidence of peripheral parasitaemia was lower in the co-trimoxazole plus dihydroartemisinin–piperaquine group than in the co-trimoxazole plus placebo group when detected by any test (IRR 0·28, 95% CI 0·18–0·44, p<0·0001), by PCR (IRR 0·22, 0·13–0·38, p<0·0001), or by microscopy (IRR 0·25, 0·13–0·48, p<0·0001). The incidence of clinical malaria during pregnancy was 2·3 per 100 person-years in the cotrimoxazole plus dihydroartemisinin–piperaquine group and 8·2 per 100 person-years in the co-trimoxazole plus placebo group (IRR 0·29 [95% CI 0·08–1·03], p=0·056, number needed to treat 60 [95% CI 31–1254]). Similar findings were observed with covariate-adjusted analysis (figure 2) or when results were expressed as cumulative risk (appendix p 21) or in the per-protocol population (appendix p 18).

At delivery, risk of malaria infection in peripheral or placental blood detected by any test was lower in the co-trimoxazole plus dihydroartemisinin–piperaquine group than in the co-trimoxazole plus placebo group (RR 0·37, 95% CI 0·19–0·70, p=0·0023; figure 2). Risk of any placental malaria (active and past) detected by histology was lower in the cotrimoxazole plus dihydroartemisinin–piperaquine group than in the co-trimoxazole plus placebo group (RR 0·62, 95% CI 0·45–0·85, p=0·0036); risk of active placental malaria detected by histology was also lower in the co-trimoxazole plus dihydroartemisinin–piperaquine group (RR 0·23, 0·07–0·80, p=0·021). Placental inflammation and chorioamnionitis assessed by placental histology did not differ between groups (figure 2).

Mean haemoglobin concentration in the third trimester or at delivery (post-hoc analysis) and prevalence of maternal anaemia detected at delivery or in the third trimester did not differ between groups (table 2, figure 2). Gestational weight gain per week was significantly lower in the co-trimoxazole plus dihydroartemisinin–piperaquine group than in the co-trimoxazole plus placebo group (mean difference −0·06 kg per week, 95% CI −0·09 to −0·03, p<0·0001). Differences between groups in weekly gains in gestational mid-upper arm circumference assessment during pregnancy were small (mean difference −0·02 mm per week, 95% CI −0·14 to 0·10, p=0·71), and the mean mid-upper arm circumference assessment at delivery was similar (mean difference 0·1 cm, −0·3 to 0·5, p=0·61; table 2, appendix p 13). 20 (5%) of 399 participants in the co-trimoxazole plus dihydroartemisinin–piperaquine group and 15 (4%) of 405 participants in the co-trimoxazole plus placebo group had antibodies against SARS-CoV-2 at delivery (RR 1·33, 95% CI 0·69–2·56, p=0·39; figure 2).

**Figure 3:**
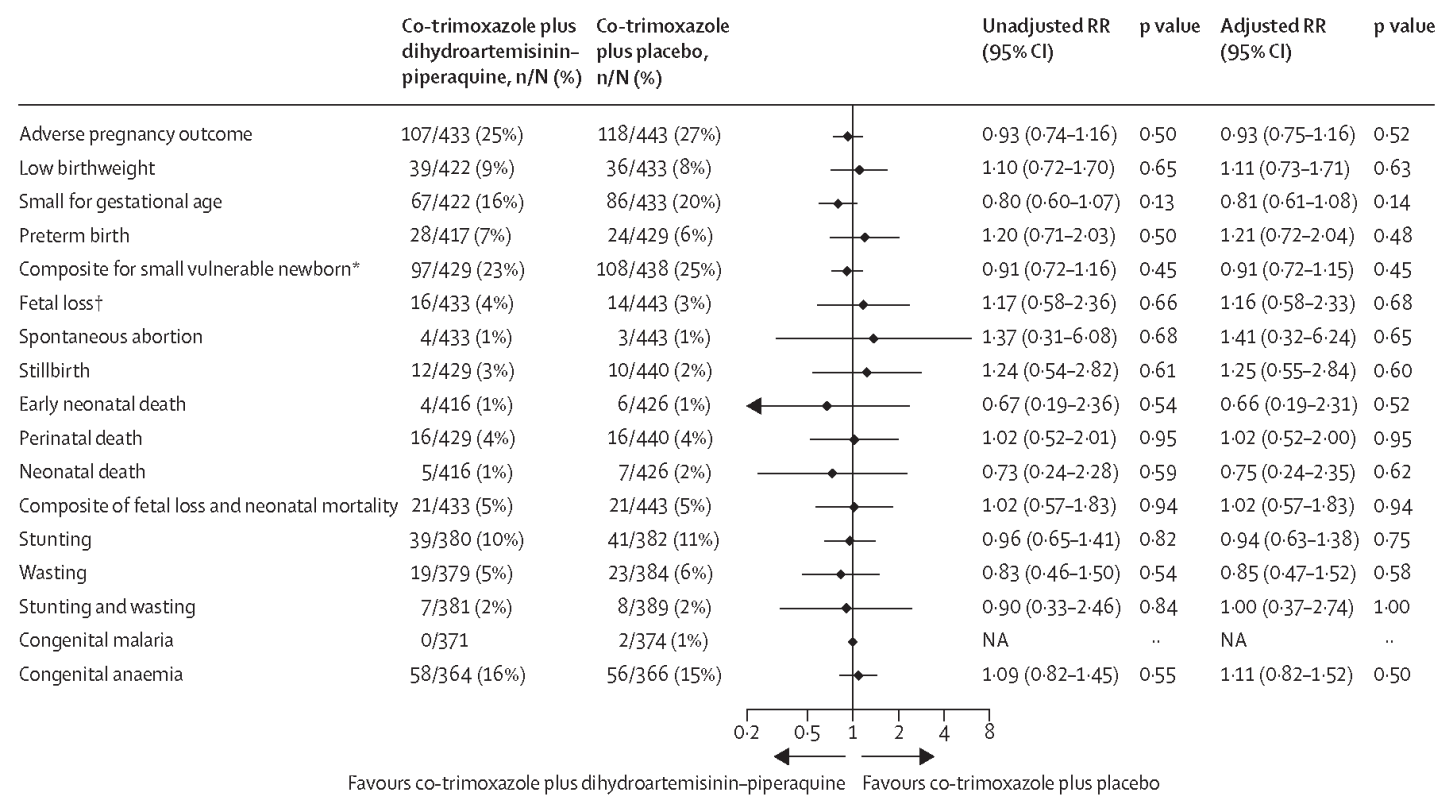
Adverse pregnancy outcomes (modified intention-to-treat population) The unadjusted RR values were obtained from log-binomial regression models with the stratification factors of site and HIV status (known positive *vs* newly diagnosed HIV infection) included as covariates. The adjusted analyses were done using several other prespecified baseline covariates in addition to HIV status and site, including gravidity, malaria status, socioeconomic status, season (average rainfall in the last 6 months before delivery), and malaria transmission intensity by study site (based on the prevalence of malaria at enrolment, continuous). NA=not applicable. RR=risk ratio. *Composite for low birthweight, small for gestational age, and preterm birth. †Fetal loss included miscarriage and stillbirth.

Occurrence of the composite adverse pregnancy outcome and its components, including low birthweight, small for gestational age, preterm birth, fetal loss, and neonatal mortality did not differ significantly between the co-trimoxazole plus dihydroartemisinin–piperaquine group and the co-trimoxazole plus placebo group (figure 3). Mean birthweight, Z scores for birthweight by gestational age, neonatal length, and Z score for neonatal length did not differ between groups (table 2, appendix p 13).

Both regimens were well tolerated. Overall, two (<1%) of 446 women in the co-trimoxazole plus dihydroartemisinin–piperaquine group and three (1%) of 455 women in the co-trimoxazole plus placebo group vomited within 30 min of an initial dose, with none vomiting after the repeat dose (table 3). Nausea within the first 4 days after the start of treatment was more common in the co-trimoxazole plus dihydroartemisinin–piperaquine group than in the co-trimoxazole plus placebo group (29 [7%] of 446 women *vs* 12 [3%] of 455 women; table 3, appendix pp 15–16). All events of nausea were transient (≤2 days), and most self-reported as mild (28 [97%] of 29 in the co-trimoxazole plus dihydroartemisinin–piperaquine group *vs* 12 [100%] of 12 in the co-trimoxazole plus placebo group). One event in the co-trimoxazole plus dihydroartemisinin–piperaquine group was self-reported as moderate. The incidence of serious adverse events in pregnant women or their infants was similar between groups, overall and by MedDRA system organ class (table 3). There were no statistical differences between groups in maternal mortality, congenital anomalies, mother-to-child transmission of HIV, and neonatal jaundice. The average prolongation in the QTc intervals was 17 ms in the cotrimoxazole plus dihydroartemisinin–piperaquine group and 0 ms in the co-trimoxazole plus placebo group (p<0·0001; table 3). All these events were asymptomatic; no participant had QTc values of more than 500 ms. One participant in the co-trimoxazole plus placebo group had QTc prolongation greater than 60 ms compared with none in the co-trimoxazole plus dihydroartemisinin–piperaquine group (appendix p 17).

## Discussion

This placebo-controlled trial compared the standard of care with daily co-trimoxazole versus daily co-trimoxazole combined with monthly IPTp with dihydroartemisinin–piperaquine in pregnant women living with HIV on a dolutegravir-based cART regimen in areas with moderate to high malaria transmission and high levels of *P falciparum* resistance to sulfa-based antifolate drugs. Addition of IPTp with dihydroartemisinin–piperaquine to daily co-trimoxazole was associated with a lower cumulative incidence of women with at least one malaria infection and a lower incidence of overall number of malaria infections compared with co-trimoxazole plus placebo. The incidence of clinical malaria was also lower, although this was not statistically significant (p=0·056). At delivery, the risk of active placental malaria was lower in the co-trimoxazole plus dihydroartemisinin–piperaquine group than in the co-trimoxazole plus placebo group. The risk of maternal anaemia in the third trimester or at delivery and the risk of adverse pregnancy outcomes, defined as a composite of fetal loss, being born small for gestational age, with low birthweight or preterm, or neonatal death, did not differ between groups. However, the study was not powered to address adverse pregnancy outcomes, and the results were too imprecise to provide assurances about the absence or presence of an effect on birth outcome. Both treatment regimens were well tolerated, although nausea was slightly more commonly reported with the combination. The rate of serious adverse events was similar in both groups. These results suggest that addition of monthly IPTp with an effective and well tolerated antimalarial drug such as dihydroartemisinin–piperaquine to the standard of care with daily unsupervised co-trimoxazole in areas of high antifolate resistance substantially improves malaria chemoprevention in pregnant women living with HIV and should be considered for policy.

This is one of two recent trials comparing the efficacy of adding IPTp with dihydroartemisinin–piperaquine to cotrimoxazole in women with HIV on dolutegravir-based cART. The reduction in malaria incidence is similar to that reported by González and colleagues in the near-identical trial done in Mozambique and Gabon.^14^ These results, however, are a marked contrast with the previous pilot study in 200 pregnant women with HIV on cART consisting of efavirenz, tenofovir, and lamivudine in Uganda.^8^ The study showed that monthly IPTp with dihydroartemisinin–piperaquine did not reduce the risk of placental or maternal malaria because of the clinically significant drug–drug interaction with efavirenz, resulting in a 27% lower area under the concentration (AUC; hours 0–8) time curve for dihydroartemisinin and 38% lower piperaquine AUC levels (days 0–21) than those in pregnant women without HIV and not on cART participating in a previous trial.^9,10^ By contrast with women on efavirenz-based cART, a known inducer of CYP3A4, which metabolises piperaquine, our findings suggest that the efficacy of dihydroartemisinin–piperaquine is retained in pregnant women on dolutegravir, consistent with the results of a parallel pharmacokinetic study in 13 pregnant women with HIV in Malawi, which showed that dolutegravir-based cART resulted in piperaquine exposure similar to that published previously for pregnant and non-pregnant women without HIV.^12^

The previous pharmacokinetic study in pregnant women in Malawi also showed that co-administration of dihydroartemisinin–piperaquine and dolutegravir resulted in modest increases in overall exposure to dolutegravir (AUC to 24 h post dose) by 30% (90% CI 11–52), peak plasma concentration (C_max_) by 31% (90% CI 13–51), and trough concentrations by 42% (90% CI 9–85), ensuring its efficacy.^15^ These findings are reassuring because other antimalarial drugs, including artemether–lumefantrine and amodiaquine–artesunate, reduce dolutegravir exposure.^16^ The mechanism behind increased dolutegravir exposure is unclear and could be attributed to improved bioavailability or delayed clearance of dolutegravir.^15^

Our efficacy results are consistent with the two previous trials in women with HIV in sub-Saharan Africa that added IPTp with mefloquine, a highly effective and long-acting antimalarial drug, to daily co-trimoxazole.^4,5^ A meta-analysis of these trials showed that, compared with co-trimoxazole alone, IPTp with mefloquine was associated with reductions in maternal peripheral parasitaemia at delivery and placental malaria, both detected by PCR (maternal peripheral parasitaemia RR 0·52, 95% CI 0·30–0·93; placental malaria RR 0·28, 95% CI 0·14–0·57).^17^ However, IPTp with mefloquine was poorly tolerated and not suitable for IPTp.^5,6,17^ Furthermore, significant drug–drug interactions between mefloquine and nevirapine resulted in reduced nevirapine plasma concentrations, which might have contributed to the increased risk of mother-to-child transmission of HIV in the co-trimoxazole plus mefloquine group compared with co-trimoxazole alone.^7^

Our results are also similar to the reductions in clinical malaria during pregnancy (IRR 0·32), any malaria at delivery (RR 0·39), and active placental malaria detected by histology (RR 0·29)^18^ seen in the six completed trials comparing IPTp with dihydroartemisinin–piperaquine versus the standard of care with IPTp with sulfadoxine–pyrimethamine in women without HIV.^19–24^ These findings suggest that monthly IPTp with dihydroartemisinin–piperaquine, when combined with daily co-trimoxazole in women with HIV on dolutegravir-based cART, is as effective in preventing malaria infections as IPTp with dihydroartemisinin–piperaquine alone in women without HIV.

Much larger studies would be required to provide a definitive answer on whether the combination of cotrimoxazole plus dihydroartemisinin–piperaquine results in better birth outcomes than co-trimoxazole alone. Mean birthweight, mean gestational age, and Z scores for birthweight by gestational age were approximately similar between the groups. Although the risk of the composite of adverse pregnancy outcome was slightly lower in the cotrimoxazole plus dihydroartemisinin–piperaquine group than in the co-trimoxazole plus placebo group (RR 0·93, 95% CI 0·74–1·16), as was the risk of the composite for small vulnerable newborn (low birthweight, preterm birth, or born small for gestational age; RR 0·91, 95% CI 0·72–1·16), the confidence intervals for the RRs were too wide to draw any meaningful conclusions. Nevertheless, the lack of an effect on birth outcomes was notable because malaria infections during pregnancy are harmful and their prevention results in improved pregnancy outcomes.^25^ The absence of an effect on adverse pregnancy outcomes, despite major reductions in malaria infections, is consistent with the two previous IPTp trials with mefloquine in women living with HIV,^4,5,17^ and with the recent trial by González and colleagues that also assessed adding monthly IPTp with dihydroartemisinin–piperaquine to daily co-trimoxazole in women living with HIV.^14^ Malaria is one of the many contributors to low birthweight and preterm delivery. In our study, only 15% of participants in the co-trimoxazole plus placebo group had a malaria infection at least once after enrolment, and one-third of these infections were below the detection level by microscopy. Thus, the attributable fraction of malaria to adverse pregnancy outcomes might have been modest. It is also possible that daily co-trimoxazole, even in areas with high-grade antifolate resistance, still exerts some antimalarial benefit by preventing some malaria infections or suppressing parasite densities in existing infections, thereby dampening malaria’s adverse effects on pregnancy outcomes. Furthermore, placental malaria, resulting from infected erythrocytes adhering to local chondroitin sulfate A receptors, triggers placental inflammation and subsequent placental pathology.^26^ Co-trimoxazole is known to have anti-inflammatory effects in children and adults living with HIV through its effects on the microbiome, gut epithelium, and innate immune cells,^27^ and this might further dampen the adverse effects of placental malaria. Similar anti-inflammatory effects have been postulated to occur with sulfadoxine–pyrimethamine, potentially explaining the absence of a beneficial effect of IPTp with dihydroartemisinin–piperaquine on adverse pregnancy outcomes in women without HIV compared with IPTp with sulfadoxine–pyrimethamine.^23^

The number of serious adverse events across the groups was similar, and the combination of co-trimoxazole plus dihydroartemisinin–piperaquine was well tolerated, which is important when drugs are considered for chemoprevention in asymptomatic pregnant women. However, nausea was more common in the co-trimoxazole plus dihydroartemisinin–piperaquine group (cumulative risk 7% *vs* 3% in the co-trimoxazole plus placebo group), but this did not affect adherence or contribute to dropouts. It is unclear whether nausea might have contributed to the reduced levels of maternal weight gain in women in the co-trimoxazole plus dihydroartemisinin–piperaquine group. Reduced maternal weight gain in the co-trimoxazole plus dihydroartemisinin–piperaquine group was also reported in the previous trials in women without HIV comparing dihydroartemisinin–piperaquine versus sulfadoxine–pyrimethamine and was hypothesised to reflect a malaria-independent effect of sulfadoxine–pyrimethamine through its antimicrobial effect on bacterial infections or through the gut microbiome or to reflect a potential anti-inflammatory effect of sulfadoxine–pyrimethamine.^23,28,29^ However, unlike these previous trials in women without HIV, the reduced maternal weight gain was not accompanied by lower maternal mid-upper arm circumference scores at delivery or reduced fetal growth. Indeed, the risk of infants born small for gestational age was lower in the co-trimoxazole plus dihydroartemisinin–piperaquine group than in the co-trimoxazole plus placebo group, but the confidence intervals for the RR were wide (RR 0·80, 95% CI 0·60–1·07). As expected, significant QTc prolongation was observed in the co-trimoxazole plus dihydroartemisinin–piperaquine group compared with the co-trimoxazole plus placebo group in the nested cardiac monitoring study, with a mean increase of 17 ms. No participant had QTc values that exceeded 500 ms. All changes were asymptomatic, consistent with QT prolongation associated with dihydroartemisinin–piperaquine in previous studies.^8,30,31^ With large-scale drug administration, there is a concern about the spread of drug resistance. Pregnant women living with HIV are a relatively small fraction of the population, and the corresponding selective drug pressure on the parasite population is likely to be small. However, with the updated 2022 malaria guidelines from WHO,^32^ dihydroartemisinin–piperaquine is likely to be increasingly considered for other indications, including post-discharge malaria chemoprevention, perennial malaria chemoprevention, and intermittent preventive treatment in schoolchildren. Careful monitoring is required to assess the effect of the widespread use of dihydroartemisinin–piperaquine for chemoprevention on parasite resistance to artemisinins and piperaquine.

A strength of this trial is that it was placebo-controlled to minimise assessment bias. Adherence to scheduled follow-up visits was excellent, with 97% of scheduled visits attended and 99% of participants contributing to the primary endpoint. A limitation is that this study was done in sites with high-grade antifolate resistance but where the prevalence of parasites with the highly resistant sextuple *pfdhfr/pfdhps* haplotype containing the *pfdhps* Ala581Gly mutation is still fairly uncommon (11% in western Kenya and 8% in southern Malawi),^23^ limiting our ability to assess potential effect modification by antifolate resistance level. Nevertheless, the generalisability of the study is good because this represents most of east and southern Africa. The exceptions are two main foci on the border of northern Rwanda, east Democratic Republic of the Congo, southwest Uganda, and northwest Tanzania, and in northeast Tanzania, where the prevalence of the sextuple *pfdhps* Ala581Gly mutant parasites exceeds 37%.^33^ Another limitation is that only the first-day course of monthly dihydroartemisinin–piperaquine was directly observed, and subsequent doses were taken home. Apart from the first dose each month, daily co-trimoxazole was also given unsupervised. It is reasonable to assume that the adherence to daily cotrimoxazole was similar between the two treatment groups because the IPTp component was placebo-controlled. Self-reported adherence to monthly dihydroartemisinin–piperaquine was 100%, which might reflect social desirability bias by participants. The study included multiple secondary endpoints without multiplicity adjustment, inflating the risk for type I errors. Lastly, the study was not powered to assess the effect on clinically relevant adverse birth outcomes.

In conclusion, addition of monthly dihydroartemisinin–piperaquine to daily co-trimoxazole was safe, well tolerated, and prevented approximately two out of every three malaria infections compared with the standard of care with daily co-trimoxazole plus monthly placebo. Addition of monthly treatment courses with dihydroartemisinin–piperaquine to daily co-trimoxazole should be considered for the chemoprevention of malaria in pregnancy in women living with HIV on dolutegravir-based antiretroviral drugs. Future studies of the feasibility and cost-effectiveness of implementing co-trimoxazole plus dihydroartemisinin–piperaquine in routine settings and any potential impact of using IPTp with dihydroartemisinin–piperaquine on parasite resistance are needed to inform WHO and national policies.

## Supplementary Material

supplement

## Figures and Tables

**Table 1:** Baseline characteristics by study group

	Co-trimoxazole plus dihydroartemisinin–piperaquine (n=448)	Co-trimoxazole plus placebo (n=456)

**Maternal characteristics**		
Newly diagnosed HIV infection	75/448 (17%)	77/456 (17%)
Known HIV infection	373/448 (83%)	379/456 (83%)
Already on dolutegravir-based cART	213/373 (57%)	202/379 (53%)
Previously on efavirenz-based cART and switched to dolutegravir-based cART	160/373 (43%)	177/379 (47%)
Maternal age (years)	29·2 (5·6)	29·2 (5·7)
Residence		
Rural	330/448 (74%)	338/456 (74%)
Semi-urban or urban	118/448 (26%)	118/456 (26%)
Marital status		
Single*	45/448 (10%)	53/456 (12%)
Married or cohabiting	403/448 (90%)	403/456 (88%)
Used bednet previous night	434/448 (97%)	437/456 (96%)
Attended school	442/448 (99%)	446/456 (98%)
School level completed		
None	58/448 (13%)	54/456 (12%)
Primary school	266/448 (59%)	261/456 (57%)
Secondary school	109/448 (24%)	114/456 (25%)
Higher	15/448 (3%)	27/456 (6%)
Socioeconomic status (terciles)		
Low	151/448 (34%)	150/456 (33%)
Medium	151/448 (34%)	151/456 (33%)
High	146/448 (33%)	155/456 (34%)
Pregnancy number (gravidity)		
First	32/448 (7%)	37/456 (8%)
Second	88/448 (20%)	91/456 (20%)
Third or higher	328/448 (73%)	328/456 (72%)
Gestational age (weeks)	22 (3·7)	22 (3·8)
Weight (kg)	63 (10·8)	62 (10·1)
Height (cm)	160 (7·4)	160 (7·6)
Mid-upper arm circumference (cm)	27·0 (3·4)	27·0 (3·2)
BMI (kg/m^2^)	24·4 (3·9)	24·2 (3·6)
**Laboratory findings**		
HIV viral load ≥400 copies per mL†	0/151	0/172
Detectable SARS-CoV-2 antibodies	5/403 (1%)	9/413 (2%)
Haemoglobin (g/dL)	10·5 (1·8)	10·4 (1·9)
Malaria infection		
Malaria rapid diagnostic test‡	49/348 (14%)	48/353 (14%)
Microscopy	40/448 (9%)	39/456 (9%)
PCR	63/432 (15%)	56/436 (13%)
Any§	75/448 (17%)	76/456 (17%)

Data are mean (SD) or n/N (%), unless otherwise specified. Some percentages do not add up to 100% because of rounding. See appendix p 10 for baseline characteristics by country. cART=combination antiretroviral therapy.

* Divorced, separated, widowed, or not cohabiting.

† HIV viral load was only assessed for participants switching from efavirenz to dolutegravir-based antiretroviral drugs.

‡ Malaria rapid diagnostic tests were done in all women in Kenya and symptomatic women in Malawi as per national guidelines.

§ Any malaria infection detected by malaria rapid diagnostic test, microscopy, or PCR.

**Table 2:** Secondary efficacy endpoints (continuous; modified intention-to-treat population)

	Co-trimoxazole plus dihydroartemisinin–piperaquine		Co-trimoxazole plus placebo		Unadjusted*		Adjusted†	
	n	Mean (SD)	n	Mean (SD)	Mean difference (95% CI)	p value	Mean difference (95% CI)	p value

Infant birthweight (adjusted) at delivery (g)	422	3079 (502)	433	3124 (500)	−46 (−112 to 20)	0·17	−47 (−113 to 19)	0·16
Z score for birthweight by gestational age from INTERGROWTH	379	−0·35 (0·98)	384	−0·35 (1·00)	0·00 (−0·14 to 0·14)	0·95	−0·00 (−0·14 to 0·14)	0·98
Gestational age at delivery (days)	433	275 (16)	441	277 (17)	−2 (−4 to 0)	0·098	−2 (−4 to 1)	0·14
Neonatal length (cm)	416	47·9 (3·0)	424	48·2 (3·1)	−0·3 (−0·7 to 0·1)	0·19	−0·3 (−0·7 to 0·1)	0·21
Z score for neonatal length from INTERGROWTH	380	−0·67 (1·39)	382	−0·64 (1·38)	−0·03 (−0·22 to 0·15)	0·72	−0·04 (−0·23 to 0·14)	0·66
Maternal mid-upper arm circumference at delivery (cm)	436	27·0 (3·4)	445	26·9 (3·3)	0·1 (−0·3 to 0·5)	0·61	0·1 (−0·3 to 0·5)	0·68
Maternal mid-upper arm circumference gain (mm per week)	436	−0·06 (0·97)	445	−0·04 (0·88)	−0·02 (−0·14 to 0·10)	0·71	−0·02 (−0·14 to 0·09)	0·68
Serial maternal mid-upper arm circumference gain (mm per week)‡	439	0·28 (0·98)	449	0·31 (1·03)	−0·02 (−0·14 to 0·10)	0·78	−0·03 (−0·15 to 0·10)	0·67
Maternal gestational weight gain (kg per week)	432	0·20 (0·22)	440	0·25 (0·20)	−0·06 (−0·09 to −0·03)	<0·0001	−0·06 (−0·09 to −0·03)	<0·0001
Serial maternal gestational weight gain (kg per week)‡	438	0·22 (0·27)	449	0·27 (0·23)	−0·04 (−0·07 to −0·01)	0·0088	−0·04 (−0·07 to −0·01)	0·0069
Maternal mean haemoglobin at delivery or in the third trimester (g/dL)§	415	10·6 (2·0)	427	10·8 (1·9)	−0·1 (−0·4 to 0·1)	0·40	−0·1 (−0·4 to 0·1)	0·40
Cord blood haemoglobin (g/dL)	364	14·7 (2·3)	366	14·7 (2·2)	−0·0 (−0·4 to 0·3)	0·80	−0·1 (−0·4 to 0·3)	0·72

* The unadjusted models include stratification factors site and HIV status as covariates.

† The adjusted analyses were done using several other prespecified baseline covariates in addition to HIV status and site, including gravidity, malaria status, socioeconomic status, season (average rainfall in the last 6 months before delivery), and malaria transmission intensity by study site (based on the prevalence of malaria at enrolment, continuous).

‡ Serial maternal weight gain and mid-upper arm circumference gain were analysed by mixed models for repeated measures, adjusting for the baseline value. All other effect estimates were obtained by standard linear regression.

§ Post-hoc analysis. Haemoglobin at delivery, or otherwise in the third trimester if the delivery haemoglobin was unavailable.

**Table 3:** Safety and tolerability endpoints (safety population)

	Co-trimoxazole plus dihydroartemisinin–piperaquine	Co-trimoxazole plus placebo	p value

**Prevalence measures, n/N (%)**			
Vomiting within 30 min following study drug administration			
Vomiting initial dose (first course), events/participants	0/446	0/455	NA
Vomiting initial dose at least once (any course), events/participants (%)	2/446 (0·4%)	3/455 (0·7%)	0·67
Vomiting dose (any course), events/number of courses (%)	2/2119 (0·1%)	3/2160 (0·1%)	0·67
QTc >500 ms on ECG after the third dose, n/N			
Any course	0/88	0/90	NA
First course	0/78	0/80	NA
Middle course	0/81	0/76	NA
Last course	0/69	0/66	NA
**Continuous measures, mean (SD)**			
Change in QTc (after third dose minus before first dose of IPTp), ms			
All measurements	17 (14·3)	0(12·2)	<0·0001
First course	18 (15·2)	−1 (12·2)	<0·0001
Middle course	18 (14·9)	−1 (10·9)	<0·0001
Last course	16 (12·7)	2 (13·5)	<0·0001
**Incidence measures, number of events (incidence per 100 person-years) or prevalence measure, n/N (%)**			
Adverse events within 7 days after first dose of IPTp*			
Pyrexia	0 (0·0)	0 (0·0)	NA
Asthenia	0 (0·0)	0 (0·0)	NA
Headache	8 (6·1)	6 (4·5)	0·54
Abdominal pain	0 (0·0)	1 (0·7)	0·98
Myalgia	0 (0·0)	1 (0·7)	0·98
Nausea	33 (25·4)	12 (8·9)	0·0012
Rash	1 (0·8)	1 (0·7)	0·94
Diarrhoea	0 (0·0)	0 (0·0)	NA
Vomiting†	3 (2·3)	2 (1·5)	0·61
Dizziness	14 (10·8)	14 (10·4)	0·80
Other gastrointestinal complaints	0 (0·0)	0 (0·0)	NA
Serious adverse event any time from randomisation (pregnant woman)*‡			
Any	23 (17·7)	25 (17·8)	>0·99
Maternal mortality	2/446 (0·4%)	0/455	NA
By system organ class			
Infections and infestations	0 (0·0)	1 (0·7)	0·98
Investigations	0 (0·0)	1 (0·7)	0·98
Pregnancy, puerperium, and perinatal conditions	22(16·9)	21 (14·9)	0·65
Surgical and medical procedures	0 (0·0)	2 (1·5)	0·98
Serious adverse event any time in the first 6 weeks of life (infant)‡§			
Any	23 (45·4)	21 (40·2)	0·66
Mother-to-child-transmission of HIV¶	1/239 (0·4%)	1/244 (0·4%)	0·91
Neonatal jaundice	1/429 (0·2%)	1/417 (0·2%)	0·92
By system organ class			
Congenital, familial, and genetic disorders	6 (12·4)	7 (14·1)	0·83
Infections and infestations	1 (2·1)	2 (4·0)	0·58
Pregnancy, puerperium, and perinatal conditions	10 (18·6)	8 (14·1)	0·56
Respiratory, thoracic, and mediastinal disorders	6 (12·4)	4 (8·0)	0·48

ECG=electrocardiogram. IPTp=intermittent preventive treatment in pregnancy. NA=not available because of zero values. QTc=corrected QT interval.

* Co-trimoxazole plus dihydroartemisinin-piperaquine group, n=446; co-trimoxazole plus placebo group, n=455.

† Late vomiting (>30 min following drug administration). All of these events occurred within the first 3 days after the start of drug intake—ie, during or within the 24 h after each daily dose, but excluding the first 30 min.

‡ Individual serious adverse events were assessed and graded according to standardised criteria at every scheduled monthly clinic visit, unscheduled visit, delivery, and postpartum.

§ Co-trimoxazole plus dihydroartemisinin-piperaquine group, n=4l6; co-trimoxazole plus placebo group, n=428.

¶ Results available for Kenya only.

## Data Availability

Individual participant data will be available from the Worldwide Antimalarial Resistance Network (WWARN) repository approximately 3 months after publication.

## Research in context

### Evidence before this study

In October, 2017, the WHO Malaria Policy Advisory Committee (MPAC) noted that daily unsupervised co-trimoxazole provided only partial protection against malaria for pregnant women living with HIV in areas with high-grade antifolate resistance. MPAC highlighted the need for research of new strategies, including alternative medicines for intermittent preventive treatment in pregnancy (IPTp), to be safely co-administered with daily co-trimoxazole prophylaxis. This suggestion was based on two trials done between 2012 and 2013, combining co-trimoxazole with IPTp with mefloquine in pregnant women living with HIV. The meta-analysis of these two trials showed that, compared with daily co-trimoxazole alone, the combination of IPTp with mefloquine plus co-trimoxazole was associated with a reduction in maternal peripheral parasitaemia at delivery (risk ratio [RR] 0·52, 95% CI 0·30–0·93) and a reduction in placental malaria (RR 0·28, 0·14–0·57). However, mefloquine was unsuitable for use as IPTp because it was poorly tolerated, and drug–drug interactions between mefloquine and nevirapine resulted in reduced plasma concentrations of nevirapine, contributing to a potential increased risk of mother-to-child transmission of HIV. Trials in women without HIV also found that amodiaquine, alone or combined with sulfadoxine–pyrimethamine, and chloroquine, alone or combined with azithromycin, were unsuitable as IPTp because of poor tolerability. Dihydroartemisinin–piperaquine was the only antimalarial drug to show promise as a potential alternative to sulfadoxine–pyrimethamine for IPTp in women without HIV. We did an electronic literature search for studies of IPTp with dihydroartemisinin–piperaquine in pregnant women living with HIV. We searched ClinicalTrials.gov, the WHO International Clinical Trials Registry Platform, PubMed, Web of Science, and the Malaria in Pregnancy Library (which consists of references from Web of Knowledge, Scopus, CINAHL, Bioline, the Cochrane Library databases, and WHO Global Health Library) from database inception to Aug 1, 2023. The Malaria in Pregnancy Library also contains grey literature and conference abstracts. No language restrictions were used. We identified one trial of dihydroartemisinin–piperaquine plus co-trimoxazole in pregnant women living with HIV, involving 200 women on combination antiretroviral therapy (cART) consisting of efavirenz, tenofovir, and lamivudine, done in Uganda between 2014 and 2016. Addition of monthly IPTp with dihydroartemisinin–piperaquine to daily co-trimoxazole did not reduce the risk of malaria infection because of a low malaria burden due to the introduction of indoor residual spraying in the study area. Furthermore, a subsequent pharmacokinetic analysis of the data showed a clinically significant drug–drug interaction with efavirenz, resulting in a 27% reduction in exposure to dihydroartemisinin and 38% reduction in exposure to piperaquine. Another pharmacokinetic–pharmacodynamic study in 13 pregnant women living with HIV in Malawi showed that, unlike with efavirenz-based cART, dolutegravir-based cART resulted in piperaquine drug concentrations similar to those published previously for pregnant and non-pregnant women. No negative effects of dihydroartemisinin–piperaquine on dolutegravir exposure were seen. Our literature search also identified one other trial done between 2018 and 2023 using a similar design to ours, comparing co-trimoxazole plus dihydroartemisinin–piperaquine versus co-trimoxazole plus placebo in 666 pregnant women living with HIV in Mozambique and Gabon.

### Added value of this study

This is one of two trials comparing the efficacy of adding IPTp with dihydroartemisinin–piperaquine to daily unsupervised co-trimoxazole in women living with HIV in the context of the newer WHO-recommended first-line dolutegravir-based cART regimen in areas with moderate to high malaria transmission and high levels of *Plasmodium falciparum* resistance to sulfa-based antifolate drugs. After enrolment, approximately 15% of women in the co-trimoxazole plus placebo group had evidence of at least one malaria infection during pregnancy or delivery. Addition of monthly dihydroartemisinin–piperaquine to daily co-trimoxazole reduced the incidence of malaria infection compared with placebo plus co-trimoxazole. The risk of maternal anaemia or adverse birth outcomes did not differ between groups. The combination of co-trimoxazole plus dihydroartemisinin–piperaquine was well tolerated, and the frequency of serious adverse events was similar to that in the co-trimoxazole plus placebo group. By contrast with women on efavirenz-based cART in the previous Ugandan trial, our findings suggest that the efficacy of dihydroartemisinin–piperaquine is retained in pregnant women on dolutegravir, consistent with the results of the pharmacokinetic study in pregnant women living with HIV in Malawi.

### Implications of all the available evidence

Addition of monthly IPTp with an effective and well tolerated long-acting antimalarial drug such as dihydroartemisinin–piperaquine to the standard of care with daily unsupervised co-trimoxazole in areas of high antifolate resistance has the potential to substantially improve malaria chemoprevention in pregnant women living with HIV on dolutegravir-based cART and could be considered for policy. Future studies of the feasibility and cost-effectiveness of implementing the combination of daily co-trimoxazole plus monthly dihydroartemisinin–piperaquine in routine settings are needed to inform WHO and national policies.
