# Supplementary material for: Chemoprevention for malaria with monthly intermittent preventive treatment with dihydroartemisinin–piperaquine in pregnant women living with HIV on daily co-trimoxazole in Kenya and Malawi: a randomised, double-blind, placebo-controlled trial
Source: Lancet. Author manuscript; Available in PMC 2025 Jan 27. (PMC10865779; doi:10.1016/S0140-6736(23)02631-4)
Supplement: supplement [file NIHMS1965437-supplement-supplement.pdf]

THE LANCET

Supplementary appendix

This appendix formed part of the original submission and has been peer reviewed. We post it as supplied by the authors.

Supplement to: Barsosio HC, Madanitsa M, Ondieki ED, et al. Chemoprevention for malaria with monthly intermittent preventive treatment with dihydroartemisinin–piperaquine in pregnant women living with HIV on daily co-trimoxazole in Kenya and Malawi: a randomised, double-blind, placebo-controlled trial. *Lancet* 2024; published online Jan 12. https://doi.org/10.1016/S0140-6736(23)02631-4.

Supplementary appendix

Supplement to: Barsosio HC, Madanitsa M, Ondieki ED, et al. Chemoprevention for malaria with monthly intermittent preventive treatment with dihydroartemisinin-piperaquine in pregnant women living with HIV on daily cotrimoxazole in Kenya and Malawi: a double-blind, randomised placebo-controlled trial

Table of Contents

Supplemental methods ... **2**
Supplement 1: Details of study interventions ... 2
Supplement 2: Screening for Plasmodium infection at enrolment ... 2
Supplement 3: Procedures for cardiac monitoring ... 3
Supplement 4: Methods used to determine birthweight, gestational age and gestational age z-scores ... 3
Supplement 5: Definition of endpoints ... 4
Supplement 6: Laboratory procedures ... 6
Supplement 7: Statistical methods ... 7
Supplement 8: Deviations from study protocol ... 8
Supplemental references ... **8**
Supplemental tables ... **10**
Supplemental figures ... **18**
Consort checklist ... **22**

Supplemental methods

Supplement 1: Details of study interventions

IPTp-DP plus daily CTX

Participants in the IPTp-DP arm received a fixed dose (non-weight-based) of 3 tablets of DP daily for three days provided at enrolment and each subsequent monthly ANC visit in the 2nd and 3rd trimester. Each tablet of DP (D'Artepp, Fosun Pharmaceutical (Group) Co., Ltd. ["Fosun Pharma"], Shanghai, China) contained 40 mg of dihydroartemisinin and 320 mg of piperaquine. The fixed dose of 3 tablets instead of a weight-based regimen was preferred for pragmatic reasons and based on the promising results of this regimen in a previous trial in HIV-negative pregnant women in Uganda,[1] and subsequent pharmacokinetic/pharmacodynamic analysis showing that pregnant women with a low body mass index (BMI) have lower piperaquine exposures due to increased clearance (2% increase per unit decrease in BMI) compared to pregnant women with higher BMI. Due to the association between low BMI and higher clearance, weight-based dosing was associated with an increased disparity between piperaquine levels. These findings suggested that weight-based dosing for pregnant women may not be needed, as heavier women can achieve adequate exposure when given fixed-dose regimens.[2] Administration of the first daily doses of DP was directly observed in the clinic. The second and third daily doses were taken at home.

The participants in this arm also received daily CTX (see below).

Daily CTX plus placebo IPTp-DP

Participants in the dCTX arm were given one double-strength tablet of 160mg of sulfamethoxazole and 800mg of trimethoprim orally once daily (Sulfran-DS, Universal Corporation Ltd, Nairobi, Kenya). The administration of the first daily dose of CTX was also provided in the clinic, and the remaining daily doses were taken at home until the next scheduled visit when the next monthly supply of CTX was given.

Participants in this arm also received monthly placebo IPTp-DP with the same appearance and number of tablets as active DP. The placebo was also provided by Fosun Pharmaceutical (Group) Co., Ltd., Shanghai, China. Administration of the first daily doses of DP placebo was directly observed in the clinic. The second and third daily doses were taken at home.

Drug vomiting

The full daily dose of DP and CTX was repeated in case of vomiting within 30–60 mins. If the repeat DP dose was vomited, the IPTp dose was withheld.

Supplement 2: Screening for Plasmodium infection at enrolment

In Kenya, women are routinely screened for Plasmodium infection at the first antenatal booking visit, which is common practice in the malaria-endemic counties in Kenya but not in Malawi. Therefore, in Kenya, all participants were screened for Plasmodium infection at enrolment using histidine-rich protein 2 (HRP2)/plasmodium lactate dehydrogenase (pLDH) combination mRDTs (CareStart™ Malaria Pf/PAN (HRP2/pLDH) Ag Combo mRDT). All mRDT-positive women in Kenya received a standard 3-day treatment dose of artemether-lumefantrine, with the first dose provided as directly observed therapy in the clinic and the remaining doses self-administered at home. The first dose of IPTp was delayed until the next scheduled visit four weeks later for women who received artemether-lumefantrine at enrolment.

Supplement 3: Procedures for cardiac monitoring

Nested cardiac monitoring was conducted in a sub-group of 160 participants to determine the difference in QTc prolongation between the CTX-alone arm and the CTX+DP arm and the change in magnitude of DP-related QTc prolongation with each subsequent course.

Procedures

ECGs were taken before the first dose and then again 4 hours after the 3rd dose of the first course among women in both arms. ECGs were repeated approximately 1-2 and 3-4 courses later after the end of the second to the early third trimester and mid to end of the third trimester, respectively. ECGs were taken by dedicated staff at the study clinic, and local clinicians read the results.

Sample size

A sample size of 80 in each arm was estimated to provide 90% power to detect a 10ms difference in QTc prolongation (e.g. 30 *vs* 20ms) after the first course (two-sided α=0.05, standard deviation 18.3ms, t-test for independent samples, allowing for 10% non-contributors [e.g. due to missing ECG at Tmax on Day-2]). The standard deviation of 18.3ms was based on a previous trial in Indonesia.[3]

Analysis

As described previously,[4] to obtain QTc values that are independent of the RR intervals, three different heart-rate correction methods were used, based on the generic formula generic $QTc = {QT}/{RR^{\beta}}$, where β is a correction exponent, the value of which depends on the method used;[5] for the Bazett method (QTcB), the value of β is 0.5; for Fridericia's method (QTcF), the value of β is 0.33. With the third method, the value of β was study-derived (QTcD), where β was calculated based on the value of the slope from the linear regression as determined using the log-transformed values of QT and RR and the equation log(QTc)=log(QT)-log(RR)*β. QTcD was calculated using the pre-dose ECGs at baseline (i.e. before the first dose of the first course of DP). We then applied the same study-derived correction factor to all subsequent ECGs.

To QTc values obtained by the three correction methods were then plotted against the corresponding RR values, and the slopes calculated for the three linear regressions lines based on the equation QTc = a + b*RR + e, where a is the intercept, b is the slope, and e is the residual error. The method with a slope closest to zero, i.e. a horizontal regression line, was then chosen as the best correction method and used as the primary method in the subsequent analyses.

The mean QTc values on day 3 and changes from baseline on day 3 were compared using linear regression. The results were expressed as mean difference (95% CI) between study arms with stratification factors gravidity and site entered as covariates. The proportion of participants with QTc values >480 ms, >500 ms for absolute values and >60 ms change from baseline were compared using log-binomial regression with study and baseline QTc values as covariates.

Supplement 4: Methods used to determine birthweight, gestational age and gestational age z-scores

Corrected birthweights

All birthweights in the analyses refer to corrected birthweights taken within seven days (168 hours) after birth. Birthweights taken more than 1 hour after delivery were corrected for the physiological fall in birth weight in breastfed infants in the first days following delivery using daily birthweight data collected from participants in a previous study in Tanzania.[6] For newborn weight >1 hour after delivery, the corrected birthweight was imputed using a table of correction factors by Mtove et al. based on the sex of the infant, its measured weight (increasing from 2kg – 4kg in increments of 10g), and time from birth to weighing (in hourly increments from 1hr to 168hrs).[6] For newborns where sex

was unavailable, the midpoint between girls and boys was used for that time point. For infants for which the exact hour of birth and /or measurement of (birth)weight was unavailable, the weighing timepoint was assumed to be 0 hours (if birth and measurement on the same day), then 24, 48, 72, etc. hours if the child was weighed 1, 2, 3, etc. days after delivery.

Gestational age at delivery

Gestational age in days at delivery was defined as the number of days between delivery and the date that gestational age was assessed at enrolment, plus the gestation age in days assessed at enrolment. The following methods were used in order of priority: Ultrasound data were used for all subjects (904/904). The following biometric measures were used to define gestational age at enrolment: crown-rump length (CRL) was used for participants in the first trimester. In the second trimester, the combination of head circumference (HC) and femur length (FL) was used if both were available; otherwise, only the head circumference was used if femur length measures were unavailable or only femur length if head circumference measures were unavailable.

Z-scores for maternal and fetal growth monitoring

Z-scores for adjusted birthweight for gestational age for newborns with gestational ages at birth ranging between 168 and 300 days were calculated using the INTERGROWTH reference population, derived from a multi-ethnic cohort of low-risk, well-nourished mothers with uncomplicated pregnancies.[7] Z-scores for gestational weight gain were calculated using the INTERGROWTH reference population for Gestational Weight Gain[8] Z-scores for newborn size at birth were calculated using the INTERGROWTH reference population for newborn size.[7]

Supplement 5: Definition of endpoints

Efficacy endpoints

Primary endpoints

The primary endpoint was the cumulative incidence of Plasmodium infection detected in the peripheral (maternal) or placental (maternal) blood/tissue by PCR, microscopy, RDT or placental histology (active infection) from 2 weeks after the first day of the first dose of the first course to delivery inclusive.

Secondary maternal endpoints

- Clinical malaria, defined as a positive malaria mRDT (HRP2 or pLDH) or malaria smear in conjunction with documented fever (>37.5 ^{o}C) or reported fever in the past 48 hours.
- The incidence of any Plasmodium infection during pregnancy, defined as the composite of malaria detected in the peripheral blood by mRDT (for point of care), or microscopy (not for point of care), or PCR (not for point of care), from 2 weeks after enrolment and excluding delivery.
- Patent Plasmodium infection during pregnancy: Plasmodium infection during pregnancy detected by microscopy and/or mRDT, from 2 weeks after enrolment and excluding delivery.
- Sub-patent Plasmodium infection during pregnancy: Plasmodium infection detected by PCR, with negative microscopy and/or mRDT, from 2 weeks after enrolment and excluding delivery.
- Any Plasmodium infection at delivery: Any *Plasmodium* infection detected in peripheral or placental blood by PCR, microscopy, mRDT or histopathology (active infection, i.e., acute or chronic infections)
- The individual components of the composite Plasmodium infection endpoints at delivery
- Any placental malaria by histology (active or past infection)
- Placental inflammation or chorioamnionitis detected by placental histology

- Mean haemoglobin (Hb in g/dL) in the third trimester, excluding delivery, using Hb measured in either venous or capillary blood.
- Maternal anaemia in the third trimester (any anaemia [Hb<11.0g/dL], moderate-severe anaemia [Hb<9.0g/dL], severe anaemia [Hb<7.0g/dL])
- Mean haemoglobin (Hb in g/dL) at delivery, measured just before delivery of the baby in either venous or capillary blood just before delivery.
- Maternal anaemia at delivery (any anaemia [Hb<11.0g/dL], moderate-severe anaemia [Hb<9.0g/dL], severe anaemia [Hb<7.0g/dL])
- Maternal mid-upper arm circumference (MUAC) at delivery
- Low maternal MUAC at delivery, defined as less than 23 cm measured at delivery or at the last time point in the third trimester if MUAC at delivery is not available
- Maternal MUAC gain in cm per week, defined as the maternal MUAC at the last measured time point at or before delivery (e.g., in the third trimester or at delivery) minus the MUAC enrolment, divided by the number of weeks between these dates
- Gestational weight gain in kilogram per week, defined as the maternal weight measured at the last time point before delivery (e.g., in the third trimester) minus the maternal weight at enrolment, divided by the number of weeks between these dates
- Z-scores for gestational weight gain using the INTERGROWTH reference

Secondary newborn endpoints

- Z-scores for femur length, head circumference and crown-rump length using the INTERGROWTH-21st reference
- Z-score for fetal growth using the INTERGROWTH-21st reference
- Mean birthweight in kilogram (for methods, see Corrected birthweights, above)
- Mean gestational age at delivery in days (for methods, see, Supplement 4, appendix p 3)
- Z-score for birthweight using the INTERGROWTH-21st reference
- Z-scores for birthweight for gestational age using the INTERGROWTH-21st reference
- Low birth weight (LBW): A corrected birthweight < 2,500g measured
- Preterm birth (PTD): A gestational age of < 37 weeks at birth.
- Small-for-gestational-age: A birthweight <10th percentile of the INTERGROWTH-21st reference
- Adverse live birth, defined as either LBW, preterm or SGA (SGA-LBW-PT composite)
- Adverse pregnancy outcome: A composite of either fetal loss (spontaneous miscarriage or stillbirth) or adverse live birth (defined as either low birth weight [<2500 grams], small-for-gestational-age [SGA, <10th percentile relative to INTERGROWTH-21st gender-specific chart], or preterm delivery [<37 weeks gestation]), or neonatal death.
- Birth length in cm, defined as a length measured within 24 hours of birth
- Z-score for birth length at birth (cm) using the INTERGROWTH-21st reference
- Stunting at birth, defined as <3rd centile of birth length for gestational age using the INTERGROWTH-21st reference.
- Wasting at birth, defined as <3rd centile of corrected birthweight for gestational age using the INTERGROWTH-21st reference.
- Both stunting and wasting at birth
- Spontaneous miscarriage: Loss of foetus before 28 weeks gestational age
- Stillbirth: Loss of foetus at or after 28 weeks gestation or birth of foetus showing no signs of life

- Foetal loss: Stillbirth or miscarriage
- Early neonatal death, defined as the death of a live-born baby within the first week of life
- Perinatal death: stillbirths and deaths in the first week of life
- Neonatal death: death within 28 days of birth
- Composite of fetal loss and neonatal mortality
- Congenital Plasmodium infection, defined as Plasmodium infection detected in fetal cord blood or peripheral blood of the newborn at birth or within 7 days (168 hours) after birth by either mRDT (pLDH only) or microscopy
- Mean cord blood haemoglobin in g/dL, measured in umbilical cord blood at birth
- Fetal anaemia in g/dL, defined as Hb<12.5 g/dL in umbilical cord blood at birth
- Congenital anomalies: Physical abnormality of live-born baby detected at delivery or newly noted anomaly during the infant follow-up visits (7 days or 6-8 weeks post-natal)

Safety endpoints

- QTc-prolongation, defined as mean QTc, mean delta QTc, and QTc values >480ms and >500ms, or delta QTc >60ms, alone or combined with cardiac symptoms (e.g. syncope)
- Maternal mortality, defined as death at any time from enrolment onward until 42 days after delivery or termination of pregnancy
- Other Serious Adverse Events (SAEs) and Adverse Events (Aes), defined according to the Medical Dictionary for Regulatory Activities (MedDRA), overall and by system organ class and preferred term

Tolerance endpoints

- Vomiting of study drug, defined as vomiting within 30 minutes of taking the study drug at any scheduled administration
- Later vomiting at any point within 96hrs (4 days) of the first drug administered during a treatment cycle, excluding drug vomiting within 30 minutes
- Nausea at any point within 96hrs (4 days) of the first drug administered during a treatment cycle
- Dizziness at any point within 96hrs (4 days) of the first drug administered during a treatment cycle
- Gastrointestinal complaints are defined as nausea or vomiting at any point within 96hrs (4 days) of the first drug administered during a treatment cycle, excluding drug vomiting within 30 minutes

Supplement 6: Laboratory procedures

Blood smears

Blood smears were stained with Giemsa and read in duplicate by certified microscopists. A blood smear was considered negative when the examination of 100 high-power fields did not reveal the presence of asexual parasites. A third microscopist would read any discrepancies between the first and second reader. Blood smear results were considered discrepant if one reader scored them as negative and another as positive.

PCR

Dried blood spots for PCR were taken at enrolment and delivery in all women and in a random sample of one-third of all participants in each site during the scheduled follow-up visits between enrolment and delivery (i.e. approximately every four weeks). DNA was extracted from dried blood spots (50 microL) using a commercial DNA extraction kit (E Z 96 Tissue DNA Kit®, Omega BIO-TEK,

VWR, Denmark, vacuum protocol) described in detail previously.[9] The resulting DNA extracts were tested for the presence of *P. falciparum* DNA on a VIIA 7 Applied Biosystems Real-Time PCR System (Applied Biosystems, CA) using the qPCR method and conditions as described by Hofmann et al.[10] targeting the high-copy telomere-associated repetitive element 2 (TARE-2,*250 copies/genome) of *P. falciparum.* DNA of 3 mL was added into 15 mL of PowerUpTM SYBRTM Green master mix (Applied Biosystems, CA).[9] The method has a lower limit of detection of 0.03 to 0.15 parasites per µL of blood.[10]

SP resistance markers

SP resistance markers using baseline samples were collected as part of a previous study in HIV-uninfected women conducted in the same study sites and by the same study team conducted 2 years previously.[4] Illumina targeted amplicon sequencing of *P. falciparum* genes *Pfdhfr* and *Pfdhps* was used in *Plasmodium*-positive samples as described previously.[4,11] Only full haplotype profiles at codons 51, 59, 108 and 164 of *Pfdhfr* and codons 431, 436, 437, 540, 581 and 613 of *Pfdhps* were used for analysis. Mixed haplotype infections were omitted.

Supplement 7: Statistical methods

Sample size calculations

The study was designed to achieve 80% power trial to detect a ≥50% reduction in the primary endpoint from 12.0% with CTX-alone to 6.0% (RR=0.50) in the CTX+DP arm (α=0.05) and required a sample size of 898 participants (449 per arm) allowing for 20% loss-to-follow-up (N=90). One interim analysis was planned during the design phase. However, this was not implemented following further discussions with the trial steering committee and the data safety monitoring board. Thus, no alpha adjustment for multiple analyses was made.

A key secondary outcome was 'adverse pregnancy outcome' (appendix p 5). The sample size of 718 contributors (359 per arm) was estimated to provide 80% power to detect a 27.6% reduction (RR=0.724) in adverse pregnancy outcomes from 34.5% with CTX-alone to 25.0% in the CTX+DP arm (alpha=0.05). The 34.5% proportion for this outcome in the CTX-alone arm was based on data from the previously completed pregnancy trials in Malawi[12] and Kenya.[13] The frequency of this outcome was 23.0% in HIV-negative women receiving IPTp-SP. It was assumed that this outcome would be at least 1.5 times more common in HIV-infected women on cART (i.e. 1.5 x 23.0 = 34.5%) based on a meta-analysis comparing the risk of low birthweight and preterm delivery in HIV-infected *vs* HIV-negative women.[14]

Methods used for non-convergence

Log-binomial regression was used for the primary and dichotomous secondary endpoints and compared using risk ratios (RR) and corresponding 95% confidence intervals (CIs). In the case of non-convergence of the log-binomial model, modified Poisson regression with robust standard errors was used.

Methods used to determine the incidence rate difference and number needed to treat

Absolute differences for incidence rates per person-year were computed using the margins command in Stata with the predict option after fitting the Poisson regression model, followed by the pwcompare command to obtain the incidence rate difference (IRD) per person-year and corresponding 95% confidence intervals. The number-needed-to-treat (NNT) and corresponding 95% CIs to avert one event were then calculated as the inverse of the IRD and 95% CIs, where the IRD was converted to reflect the differences in the number of events per 15.5 weeks (instead of per person-year), which was the average duration of follow-up across both arms pooled.

Methods used for sub-group analysis

Prespecified subgroup analyses were performed for the primary outcome on the mITT population. The treatment effect on the primary outcome within each sub-group was estimated by repeating the crude log-binomial regression models for each subgroup. Models with and without a subgroup#Arm interaction were compared by the likelihood-ratio method to obtain a p-value for subgroup/study-arm interaction.

Supplement 8: Deviations from study protocol

The study protocol included sampling for nested pharmacokinetic studies. The results will be reported in subsequent publications. Drug-drug interactions between dolutegravir and piperaquine were addressed in a stand-alone study conducted in Malawi.[15] One interim analysis was planned during the design phase. However, this was not implemented following further discussions with the trial steering committee and the data safety monitoring board.

Supplemental references

1. Kajubi R, Ochieng T, Kakuru A, et al. Monthly sulfadoxine-pyrimethamine versus dihydroartemisinin-piperaquine for intermittent preventive treatment of malaria in pregnancy: a double-blind, randomised, controlled, superiority trial. *Lancet* 2019; **393**(10179): 1428-39.

2. Hughes E, Imperial M, Wallender E, et al. Piperaquine Exposure Is Altered by Pregnancy, HIV, and Nutritional Status in Ugandan Women. *Antimicrobial agents and chemotherapy* 2020; **64**(12).

3. Ahmed R, Poespoprodjo JR, Syafruddin D, et al. Efficacy and safety of intermittent preventive treatment and intermittent screening and treatment versus single screening and treatment with dihydroartemisinin-piperaquine for the control of malaria in pregnancy in Indonesia: a cluster-randomised, open-label, superiority trial. *The Lancet Infectious diseases* 2019; **19**(9): 973-87.

4. Madanitsa M, Barsosio HC, Minja DTR, et al. Effect of monthly intermittent preventive treatment with dihydroartemisinin-piperaquine with and without azithromycin versus monthly sulfadoxine-pyrimethamine on adverse pregnancy outcomes in Africa: a double-blind randomised, partly placebo-controlled trial. *Lancet* 2023; **401**(10381): 1020-36.

5. Funck-Brentano C, Ouologuem N, Duparc S, et al. Evaluation of the effects on the QT-interval of 4 artemisinin-based combination therapies with a correction-free and heart rate-free method. *Sci Rep* 2019; **9**(1): 883.

6. Mtove G, Abdul O, Kullberg F, et al. Weight change during the first week of life and a new method for retrospective prediction of birthweight among exclusively breastfed newborns. *Acta Obstet Gynecol Scand* 2022; **101**(3): 293-302.

7. Villar J, Cheikh Ismail L, Victora CG, et al. International standards for newborn weight, length, and head circumference by gestational age and sex: the Newborn Cross-Sectional Study of the INTERGROWTH-21st Project. *Lancet* 2014; **384**(9946): 857-68.

8. Cheikh Ismail L, Bishop DC, Pang R, et al. Gestational weight gain standards based on women enrolled in the Fetal Growth Longitudinal Study of the INTERGROWTH-21st Project: a prospective longitudinal cohort study. *Bmj* 2016; **352**: i555.

9. Saidi Q, Minja D, Njau J, Hansson H, Kavishe R, Alifrangis M. Ultrasensitive qPCR-Based Detection of Plasmodium falciparum in Pregnant Women Using Dried Blood or Whole Blood Pellet Samples Processed through Different DNA Extraction Methods. *The American journal of tropical medicine and hygiene* 2021; **106**(3): 846-9.

10. Hofmann N, Mwingira F, Shekalaghe S, Robinson LJ, Mueller I, Felger I. Ultra-sensitive detection of Plasmodium falciparum by amplification of multi-copy subtelomeric targets. *PLoS Med* 2015; **12**(3): e1001788.

11. Nag S, Ursing J, Rodrigues A, et al. Proof of concept: used malaria rapid diagnostic tests applied for parallel sequencing for surveillance of molecular markers of anti-malarial resistance in Bissau, Guinea-Bissau during 2014-2017. *Malaria journal* 2019; **18**(1): 252.
12. Madanitsa M, Kalilani L, Mwapasa V, et al. Scheduled Intermittent Screening with Rapid Diagnostic Tests and Treatment with Dihydroartemisinin-Piperaquine versus Intermittent Preventive Therapy with Sulfadoxine-Pyrimethamine for Malaria in Pregnancy in Malawi: An Open-Label Randomized Controlled Trial. *PLoS Med* 2016; **13**(9): e1002124.
13. Desai M, Gutman J, L'Lanziva A, et al. Intermittent screening and treatment or intermittent preventive treatment with dihydroartemisinin-piperaquine versus intermittent preventive treatment with sulfadoxine-pyrimethamine for the control of malaria during pregnancy in western Kenya: an open-label, three-group, randomised controlled superiority trial. *Lancet* 2015; **386**(10012): 2507-19.
14. Xiao PL, Zhou YB, Chen Y, et al. Association between maternal HIV infection and low birth weight and prematurity: a meta-analysis of cohort studies. *BMC Pregnancy Childbirth* 2015; **15**: 246.
15. Banda CG, Nkosi D, Allen E, et al. Impact of Dolutegravir-Based Antiretroviral Therapy on Piperaquine Exposure following Dihydroartemisinin-Piperaquine Intermittent Preventive Treatment of Malaria in Pregnant Women Living with HIV. *Antimicrobial agents and chemotherapy* 2022: e0058422.

Supplemental tables

TABLE-S1: BASELINE CHARACTERISTICS BY STUDY ARM AND COUNTRY

	Kenya		Malawi	
	CTX (n=353)	CTX+DP (n=348)	CTX (n=103)	CTX+DP (n=100)
**Maternal characteristics**				
Newly-diagnosed HIV Infection	17.3% (61/353)	17.8% (62/348)	15.5% (16/103)	13.0% (13/100)
Known HIV infection	82.7% (292/353)	82.2% (286/348)	84.5% (87/103)	87.0% (87/100)
Already on dolutegravir-based retroviral therapy	41.4% (121/292)	47.6% (136/286)	93.1% (81/87)	88.5% (77/87)
Previously on efavirenz-based cART and switched to dolutegravir-based cART	58.6% (171/292)	52.4% (150/286)	6.9% (6/87)	11.5% (10/87)
Maternal age (years)	29.3 (5.43)	29.1 (5.32)	29.1 (6.60)	29.7 (6.37)
Residence Rural *vs* Semi-urban/Urban	79.0% (279/353)	76.7% (267/348)	57.3% (59/103)	63.0% (63/100)
Marital status Single* *vs* Married/Cohabiting	11.9% (42/353)	10.6% (37/348)	10.7% (11/103)	8.0% (8/100)
Used bednet last night	98.6% (348/353)	98.3% (342/348)	86.4% (89/103)	92.0% (92/100)
Attended school	99.7% (352/353)	100.0% (348/348)	91.3% (94/103)	94.0% (94/100)
Schooling level				
None	13.0% (46/353)	14.9% (52/348)	7.8% (8/103)	6.0% (6/100)
Primary school	55.2% (195/353)	57.8% (201/348)	64.1% (66/103)	65.0% (65/100)
Secondary school	24.6% (87/353)	23.3% (81/348)	26.2% (27/103)	28.0% (28/100)
Higher	7.1% (25/353)	4.0% (14/348)	1.9% (2/103)	1.0% (1/100)
SES (terciles)				
Low	25.8% (91/353)	24.4% (85/348)	57.3% (59/103)	66.0% (66/100)
Medium	37.7% (133/353)	39.4% (137/348)	17.5% (18/103)	14.0% (14/100)
High	36.5% (129/353)	36.2% (126/348)	25.2% (26/103)	20.0% (20/100)
Pregnancy number (gravidity)				
First	6.2% (22/353)	8.0% (28/348)	14.6% (15/103)	4.0% (4/100)
Second	20.4% (72/353)	18.4% (64/348)	18.4% (19/103)	24.0% (24/100)
Third or higher	73.4% (259/353)	73.6% (256/348)	67.0% (69/103)	72.0% (72/100)
Gestational age (weeks)	21 (3.8)	22 (3.7)	22 (3.6)	21 (3.7)
Weight (kg)	63 (10.2)	64 (10.7)	59 (9.4)	59 (10.6)
Height (cm)	162 (7.4)	161 (7.4)	156 (6.9)	156 (6.1)
MUAC (cm)	27.0 (3.27)	27.1 (3.49)	26.8 (2.97)	26.8 (3.18)
BMI (kg/m^2)	24.2 (3.66)	24.4 (3.95)	24.3 (3.23)	24.0 (3.76)
**Laboratory findings**				
HIV viral load ≥400 copies/mL*	0.0% (0/162)	0.0% (0/145)	0.0% (0/6)	0.0% (0/10)
Detectable SARS-Cov-2 antibodies	2.7% (9/328)	1.6% (5/319)	0.0% (0/85)	0.0% (0/84)
Haemoglobin (g/dL)	10.3 (2.02)	10.3 (1.90)	11.0 (1.36)	10.9 (1.37)
Malaria infection				
mRDT†	13.6% (48/353)	14.1% (49/348)	-	-
Microscopy	9.9% (35/353)	11.2% (39/348)	3.9% (4/103)	1.0% (1/100)
PCR	14.9% (51/343)	17.1% (58/340)	4.9% (5/103)	5.0% (5/100)
Any‡	19.5% (69/353)	20.1% (70/348)	6.8% (7/103)	5.0% (5/100)

Unless otherwise specified, data are mean (SD) or n/N (%). Some percentages do not add up to 100 because of rounding. BMI=body mass index. CTX=cotrimoxazole. DP=dihydroartemisinin-piperaquine. g/dL=grams per decilitre. HIV= human immunodeficiency virus. mRDT=malaria rapid diagnostic test. MUAC=mid-upper arm circumference. PCR=polymerase chain reaction. SARS-CoV-2=severe acute respiratory syndrome coronavirus 2. SES=socioeconomic status. *Divorced, separated, widowed, or not cohabiting. †HIV viral load was only assessed for participants switching from efavirenz to dolutegravir-based ARVs. ‡mRDTs were conducted in all women in Kenya and symptomatic women in Malawi as per national guidelines. §Any malaria infection detected by mRDT, microscopy, or PCR.

TABLE-S2: FOLLOW-UP VISITS SCHEDULE (MODIFIED INTENTION TO TREAT POPULATION)*

	Overall (n=903)*	CTX (n=456)	CTX+DP (n=447)*
Duration of follow-up until delivery, pregnancy loss, or loss-to-follow-up (in days)			
Median (IQR) (range)	108 (86-133), (11-186)	110 (87-135), (13-186)	108 (85-132), (11-165)
Possible no. of scheduled visits† adjusted for early delivery‡, including enrolment, excluding delivery, no (%)			
1	0 (0.0%)	0 (0.0%)	0 (0.0%)
2	0 (0.0%)	0 (0.0%)	0 (0.0%)
3	85 (9.4%)	42 (9.2%)	43 (9.6%)
4	238 (26.4%)	128 (28.1%)	110 (24.6%)
5	301 (33.3%)	141 (30.9%)	160 (35.8%)
6	279 (30.9%)	145 (31.8%)	134 (30.0%)
Total planned scheduled visit	4386	2213	2173
Total achieved scheduled visits	4242	2142	2100
% achieved of planned visits	96.7%	96.8%	96.6%
Number of IPTp received, no(%)§			
0	3 (0.4%)	1 (0.2%)	2 (0.4%)
1	16 (1.8%)	8 (1.8%)	8 (1.8%)
2	29 (3.2%)	16 (3.5%)	13 (2.9%)
3	101 (11.2%)	51 (11.2%)	50 (11.2%)
4	245 (27.1%)	127 (27.9%)	118 (26.4%)
5	261 (28.9%)	120 (26.3%)	141 (31.5%)
6	219 (24.3%)	118 (25.9%)	101(22.6%)
7	29 (3.2%)	15 (3.3%)	14 (3.1%)
Total number of IPTp courses	4179	2114	2065
Number of IPTp courses, median (IQR, range)	5 (4-6, 0-7)	5 (4-6, 0-7)	5 (4-6, 0-7)
Number of women visited at home to assess adherence	722/903 (80.0%)	365/456 (78.5%)	357/447 (79.9%)
% of women correctly self-administering DP or DP placebo doses on days 2 and 3 at home as prescribed (random home visits)¶	722/722 (100.0%)	365/365 (100.0%)	357/357 (100.0%)

CTX=cotrimoxazole. DP=dihydroartemisinin-piperaquine. IPTp=intermittent preventive treatment in pregnancy. IQR=interquartile range.

* The modified intention-to-treat population excludes 1 participant from the intention-to-treat population randomised to the CTX+DP group, who was later found to meet the exclusion for randomisation.

† The number of monthly scheduled visits was dependent on the gestational age at enrolment

‡ Adjusted for early delivery (i.e. excludes all planned antenatal visits that could not have occurred because the pregnancy ended before that scheduled date)

§ The number of IPTp courses received can be more than the number of planned scheduled visits for women that delivered after 40 weeks of gestation

¶ Adherence was assessed during random home visits at least once during pregnancy on days 2 and 3 after a scheduled visit.

TABLE-S3: PROPORTION OF WOMEN WITH MISSING DATA FOR THE PRIMARY ENDPOINT BY TREATMENT ARM (MODIFIED ITT POPULATION)

	CTX	CTX+DP	Risk Ratio (95% CI), p-value CTX v CTX+DP
no/No (%) of patients with missing/partial primary endpoint			
Overall	4/456 (0.9%)	4/447 (0.9%)	1.03 (0.26, 4.08), p-value 0.97
Kenya	4/353 (1.1%)	3/347 (0.9%)	0.77 (0.17, 3.41), p-value 0.73
Malawi	0/103 (0.0%)	1/100 (1.0%)	NA

CTX=cotrimoxazole. DP=dihydroartemisinin-piperaquine. NA=not available because of zero events in one of the groups.

Table S4: Secondary efficacy endpoints (continuous) by country (modified ITT population)

	Number of women, mean (SD)				Mean difference (95% CI), p-value	
	CTX		CTX+DP		Unadjusted*	Adjusted
	N	Mean (SD)	N	Mean (SD)		
**Infant birthweight (adjusted) at delivery (g)**						
Overall	433	3124 (500)	422	3079 (502)	-46.10 (-112, 20), p=0.17	-47.01 (-113, 19), p=0.16
Kenya	336	3178 (487)	329	3122 (456)	-56.22 (-128, 16), p=0.13	-56.92 (-129, 15), p=0.12
Malawi	97	2939 (505)	93	2926 (617)	-10.61 (-169, 148), p=0.90	-19.32 (-181, 143), p=0.82
**Z score for birthweight by gestational age from INTERGROWTH**						
Overall	384	-0.35 (0.997)	379	-0.35 (0.975)	0.00 (-0.14, 0.14), p=0.95	-0.00 (-0.14, 0.14), p=0.98
Kenya	314	-0.32 (0.991)	314	-0.35 (0.946)	-0.02 (-0.18, 0.13), p=0.75	-0.03 (-0.18, 0.12), p=0.73
Malawi	70	-0.48 (1.017)	65	-0.35 (1.114)	0.14 (-0.22, 0.50), p=0.43	0.12 (-0.24, 0.48), p=0.51
**Gestational age at delivery (days)**						
Overall	441	277 (16.9)	433	275 (15.7)	-2 (-4, 0), p=0.098	-2 (-4, 1), p=0.14
Kenya	342	278 (16.8)	335	277 (12.8)	-1 (-3, 2), p=0.56	-1 (-3, 2), p=0.59
Malawi	99	273 (16.6)	98	267 (21.3)	-6 (-11, -0), p=0.038	-5 (-10, 0), p=0.060
**Neonatal length (cm)**						
Overall	424	48.2 (3.11)	416	47.9 (3.05)	-0.3 (-0.7, 0.1), p=0.19	-0.3 (-0.7, 0.1), p=0.21
Kenya	328	48.7 (3.00)	325	48.3 (2.71)	-0.3 (-0.8, 0.1), p=0.13	-0.3 (-0.8, 0.1), p=0.13
Malawi	96	46.4 (2.88)	91	46.4 (3.68)	-0.0 (-1.0, 0.9), p=0.95	0.0 (-0.9, 1.0), p=0.95
**Z score for neonatal length from INTERGROWTH**						
Overall	382	-0.64 (1.375)	380	-0.67 (1.386)	-0.03 (-0.22, 0.15), p=0.72	-0.04 (-0.23, 0.14), p=0.66
Kenya	313	-0.44 (1.324)	315	-0.54 (1.301)	-0.10 (-0.30, 0.10), p=0.34	-0.11 (-0.31, 0.10), p=0.31
Malawi	69	-1.56 (1.231)	65	-1.30 (1.608)	0.27 (-0.18, 0.73), p=0.24	0.30 (-0.16, 0.76), p=0.20
**Maternal MUAC at delivery (cm)**						
Overall	445	26.9 (3.33)	436	27.0 (3.44)	0.1 (-0.3, 0.5), p=0.61	0.1 (-0.3, 0.5), p=0.68
Kenya	345	26.9 (3.41)	339	27.0 (3.52)	0.1 (-0.4, 0.6), p=0.61	0.1 (-0.4, 0.6), p=0.65
Malawi	100	26.8 (3.07)	97	26.9 (3.19)	0.0 (-0.8, 0.9), p=0.92	-0.0 (-0.9, 0.8), p=0.91
**Maternal MUAC gain (mm/week)**						
Overall	445	-0.04 (0.882)	436	-0.06 (0.969)	-0.02 (-0.14, 0.10), p=0.71	-0.02 (-0.14, 0.09), p=0.68
Kenya	345	-0.07 (0.924)	339	-0.09 (1.009)	-0.01 (-0.16, 0.13), p=0.84	-0.02 (-0.16, 0.12), p=0.80
Malawi	100	0.06 (0.712)	97	0.01 (0.811)	-0.05 (-0.25, 0.15), p=0.62	-0.05 (-0.26, 0.15), p=0.62
**Serial maternal MUAC gain (mm/week) †**						
Overall	449	0.31 (1.034)	439	0.28 (0.975)	-0.02 (-0.14, 0.10), p=0.78	-0.03 (-0.15, 0.10), p=0.67
Kenya	347	0.37 (0.986)	341	0.34 (0.974)	-0.01 (-0.15, 0.12), p=0.84	-0.02 (-0.16, 0.11), p=0.76
Malawi	102	0.11 (1.166)	98	0.07 (0.954)	-0.01 (-0.29, 0.26), p=0.92	-0.04 (-0.31, 0.24), p=0.80
**Maternal gestational weight gain (kg/week)**						
Overall	440	0.25 (0.196)	432	0.20 (0.222)	-0.06 (-0.09, -0.03), p=<0.0001	-0.06 (-0.09, -0.03), p=<0.0001
Kenya	343	0.26 (0.185)	338	0.20 (0.208)	-0.06 (-0.09, -0.03), p=0.0001	-0.06 (-0.09, -0.03), p=<0.0001
Malawi	97	0.23 (0.229)	94	0.18 (0.267)	-0.05 (-0.12, 0.02), p=0.17	-0.05 (-0.12, 0.02), p=0.17
**Serial maternal gestational weight gain (kg/week) †**						
Overall	449	0.27 (0.227)	438	0.22 (0.274)	-0.04 (-0.07, -0.01), p=0.0088	-0.04 (-0.07, -0.01), p=0.0069
Kenya	347	0.27 (0.214)	341	0.23 (0.259)	-0.04 (-0.08, -0.01), p=0.018	-0.04 (-0.08, -0.01), p=0.015
Malawi	102	0.25 (0.266)	97	0.20 (0.320)	-0.04 (-0.12, 0.03), p=0.23	-0.05 (-0.12, 0.02), p=0.18
**Maternal mean Hb at delivery or in the 3rd trimester (g/dL)‡**						
Overall	427	10.8 (1.89)	415	10.6 (1.97)	-0.1 (-0.4, 0.1), p=0.40	-0.1 (-0.4, 0.1), p=0.40
Kenya	337	10.6 (2.03)	333	10.5 (2.09)	-0.1 (-0.4, 0.2), p=0.48	-0.1 (-0.4, 0.2), p=0.46
Malawi	90	11.3 (1.12)	82	11.2 (1.26)	-0.1 (-0.5, 0.3), p=0.57	-0.1 (-0.5, 0.2), p=0.49
**Maternal mean Hb in the 3rd trimester (g/dL)**						
Overall	420	10.6 (1.79)	410	10.4 (1.85)	-0.1 (-0.4, 0.1), p=0.30	-0.1 (-0.4, 0.1), p=0.28
Kenya	332	10.4 (1.89)	328	10.3 (1.94)	-0.1 (-0.4, 0.2), p=0.40	-0.1 (-0.4, 0.1), p=0.35
Malawi	88	11.2 (1.14)	82	11.1 (1.23)	-0.1 (-0.5, 0.2), p=0.46	-0.2 (-0.5, 0.2), p=0.38
**Maternal Hb at delivery (g/dL)**						
Overall	176	11.1 (2.06)	190	10.7 (2.17)	-0.4 (-0.8, 0.1), p=0.11	-0.3 (-0.8, 0.1), p=0.12
Kenya	170	11.1 (2.08)	180	10.7 (2.19)	-0.4 (-0.8, 0.1), p=0.11	-0.4 (-0.8, 0.1), p=0.12
Malawi	6	11.9 (1.08)	10	11.8 (1.47)	0.3 (-1.3, 1.9), p=0.72	-0.4 (-2.0, 1.3), p=0.66
**Cord blood Hb (g/dL)**						
Overall	366	14.7 (2.23)	364	14.7 (2.30)	-0.0 (-0.4, 0.3), p=0.80	-0.1 (-0.4, 0.3), p=0.72
Kenya	294	14.9 (2.20)	299	14.9 (2.31)	-0.0 (-0.4, 0.3), p=0.95	-0.0 (-0.4, 0.3), p=0.91
Malawi	72	14.1 (2.28)	65	14.1 (2.12)	-0.2 (-0.8, 0.4), p=0.59	-0.3 (-0.9, 0.4), p=0.42

CTX=cotrimoxazole. DP=dihydroartemisinin-piperaquine. CI=confidence interval. Cm=centimetre. g/dL=grams per decilitre. Hb=haemoglobin. Kg=kilogram. MUAC=mid-upper arm circumference. SD=standard deviation * The unadjusted models

include stratification factors site and HIV status as covariates. †Serial maternal weight gain and MUAC gain were analysed by mixed models for repeated measures, adjusting for the baseline value. All other effect estimates were obtained by standard linear regression. ‡ *post-hoc.* Hb at delivery, or otherwise in the 3rd trimester if the delivery Hb was unavailable.

Table S5: Drug tolerability within the four days after the start of an IPTp course

	Number of women, n/N(%)		crude RR/IRR* (95% CI), p-value
	CTX	CTX+DP	CTX v CTX+DP
**Vomiting after 30 minutes**			
Any	2/455 (0.4%)	3/446 (0.7%)	1.51 (0.26, 8.90), 0.65
Month 1	0/455 (0.0%)	0/446 (0.0%)	NA
Month 2	0/448 (0.0%)	2/436 (0.5%)	NA
Month 3	1/437 (0.2%)	1/426 (0.2%)	1.02 (0.07, 16.10), 0.99
Month 4	1/392 (0.3%)	0/388 (0.0%)	NA
Month 5	0/267 (0.0%)	0/278 (0.0%)	NA
Month 6	0/143 (0.0%)	0/128 (0.0%)	NA
Month 7	0/18 (0.0%)	0/17 (0.0%)	NA
Rate (IRR)	14.9	23.1	1.59 (0.27, 9.51), 0.61
**Nausea**			
Any	12/455 (2.6%)	29/446 (6.5%)	2.46 (1.29, 4.69), 0.0063
Month 1	3/455 (0.7%)	9/446 (2.0%)	2.99 (0.82, 10.87), 0.096
Month 2	3/448 (0.7%)	8/436 (1.8%)	2.78 (0.75, 10.27), 0.13
Month 3	3/437 (0.7%)	7/426 (1.6%)	2.41 (0.63, 9.19), 0.20
Month 4	2/392 (0.5%)	7/388 (1.8%)	3.58 (0.75, 17.03), 0.11
Month 5	1/267 (0.4%)	2/278 (0.7%)	2.09 (0.19, 22.71), 0.55
Month 6	0/143 (0.0%)	0/128 (0.0%)	NA
Month 7	0/18 (0.0%)	0/17 (0.0%)	NA
Rate (IRR)	89.2	253.7	2.99 (1.54, 5.78), 0.0012
**Abdominal pain**			
Any	1/455 (0.2%)	0/446 (0.0%)	NA
Month 1	0/455 (0.0%)	0/446 (0.0%)	NA
Month 2	1/448 (0.2%)	0/436 (0.0%)	NA
Month 3	0/437 (0.0%)	0/426 (0.0%)	NA
Month 4	0/392 (0.0%)	0/388 (0.0%)	NA
Month 5	0/267 (0.0%)	0/278 (0.0%)	NA
Month 6	0/143 (0.0%)	0/128 (0.0%)	NA
Month 7	0/18 (0.0%)	0/17 (0.0%)	NA
Rate (IRR)	7.4	0	p=0.98
**Diarrhoea**			
Any	0/455 (0.0%)	0/446 (0.0%)	NA
Month 1	0/455 (0.0%)	0/446 (0.0%)	NA
Month 2	0/448 (0.0%)	0/436 (0.0%)	NA
Month 3	0/437 (0.0%)	0/426 (0.0%)	NA
Month 4	0/392 (0.0%)	0/388 (0.0%)	NA
Month 5	0/267 (0.0%)	0/278 (0.0%)	NA
Month 6	0/143 (0.0%)	0/128 (0.0%)	NA
Month 7	0/18 (0.0%)	0/17 (0.0%)	NA
Rate (IRR)	0	0	NA

**Other gastrointestinal complaints**			
Any	0/455 (0.0%)	0/446 (0.0%)	NA
Month 1	0/455 (0.0%)	0/446 (0.0%)	NA
Month 2	0/448 (0.0%)	0/436 (0.0%)	NA
Month 3	0/437 (0.0%)	0/426 (0.0%)	NA
Month 4	0/392 (0.0%)	0/388 (0.0%)	NA
Month 5	0/267 (0.0%)	0/278 (0.0%)	NA
Month 6	0/143 (0.0%)	0/128 (0.0%)	NA
Month 7	0/18 (0.0%)	0/17 (0.0%)	NA
Rate (IRR)	0	0	NA
**Dizziness**			
Any	14/455 (3.1%)	10/446 (2.2%)	0.75 (0.34, 1.64), 0.47
Month 1	3/455 (0.7%)	6/446 (1.3%)	2.02 (0.52, 7.87), 0.31
Month 2	4/448 (0.9%)	3/436 (0.7%)	0.78 (0.18, 3.42), 0.74
Month 3	456/437 (104.3%)	3/426 (0.7%)	0.77 (0.18, 3.41), 0.74
Month 4	3/392 (0.8%)	2/388 (0.5%)	0.69 (0.12, 4.05), 0.68
Month 5	0/267 (0.0%)	0/278 (0.0%)	NA
Month 6	0/143 (0.0%)	0/128 (0.0%)	NA
Month 7	0/18 (0.0%)	0/17 (0.0%)	NA
Rate (IRR)	104.0	107.6	1.10 (0.53, 2.31), 0.80
**Headache**			
Any	5/455 (1.1%)	7/446 (1.6%)	1.42 (0.46, 4.41), 0.55
Month 1	1/455 (0.2%)	2/446 (0.4%)	2.03 (0.19, 22.11), 0.56
Month 2	2/448 (0.4%)	2/436 (0.5%)	1.02 (0.14, 7.15), 0.99
Month 3	1/437 (0.2%)	1/426 (0.2%)	1.02 (0.07, 16.10), 0.99
Month 4	1/392 (0.3%)	1/388 (0.3%)	1.04 (0.07, 16.46), 0.98
Month 5	0/267 (0.0%)	1/278 (0.4%)	NA
Month 6	0/143 (0.0%)	0/128 (0.0%)	NA
Month 7	0/18 (0.0%)	0/17 (0.0%)	NA
Rate (IRR)	37.1	53.8	1.48 (0.47, 4.66), 0.50
**Rash**			
Any	1/455 (0.2%)	1/446 (0.2%)	1.02 (0.07, 16.10), 0.99
Month 1	0/455 (0.0%)	0/446 (0.0%)	NA
Month 2	0/448 (0.0%)	1/436 (0.2%)	NA
Month 3	0/437 (0.0%)	0/426 (0.0%)	NA
Month 4	0/392 (0.0%)	0/388 (0.0%)	NA
Month 5	1/267 (0.4%)	0/278 (0.0%)	NA
Month 6	0/143 (0.0%)	0/128 (0.0%)	NA
Month 7	0/18 (0.0%)	0/17 (0.0%)	NA
Rate (IRR)	7.4	7.7	1.12 (0.07, 17.93), 0.94

RR=Risk ratio. IRR=Incident Rate Ratio. CI=confidence interval. NA=Not available because of zero values. Tolerability was assessed by assessing adverse events within the first four days of an IPTp course. CTX=contrimoxazole. DP=dihydroartemisinin-piperaquine.

TABLE-S6: ECG MONITORING QTC

Dose	Day and Time		Measure	CTX n=91	CTX+DP n=88	MD (95% CI) or RR (95% CI)*	p-value
First	Day-0 ((baseline)	QTc	Mean (SD)	427 (16.2)	427 (14.8)	0.2 (-4.7, 5.0)	0.95
			Range	373-468	395-456		
			>480ms, n (%)	0/81 (0%)	0/78 (0%)	-	-
			>500ms, n (%)	0/81 (0%)	0/78 (0%)	-	-
	Day-2 + 4hr		Mean (SD)	426 (15.9)	445 (18.9)	18.8 (13.4, 24.3)	<0.0001
			Range	377-463	397-484		
			>480ms, n (%)	0/80 (0%)	2/78 (3%)	-	-
			>500ms, n (%)	0/80 (0%)	0/78 (0%)	-	-
	from Day-0 first course	dQTc	Mean (SD)	-1 (12.2)	18 (15.2)	18.7 (14.4, 23.0)	<0.0001
			>60ms, n (%)	0/80 (0%)	0/78 (0%)	-	-
Middle	Day-0	QTc	Mean (SD)	425 (14.0)	422 (16.1)	-2.2 (-6.9, 2.5)	0.37
			Range	392-452	388-460		
			>480ms, n (%)	0/78 (0%)	0/82 (0%)	-	-
			>500ms, n (%)	0/78 (0%)	0/82 (0%)	-	-
	Day-2 + 4hr		Mean (SD)	423 (14.0)	441 (18.5)	17.2 (12.0, 22.3)	<0.0001
			Range	392-452	403-494		
			>480ms, n (%)	0/77 (0%)	3/81 (4%)	-	-
			>500ms, n (%)	0/77 (0%)	0/81 (0%)	-	-
	from Day-0 middle course	dQTc	Mean (SD)	-1 (10.9)	18 (14.9)	19.1 (15.0, 23.2)	<0.0001
			>60ms, n (%)	0/77 (0%)	0/81 (0%)	-	-
	from Day-0 first course	dQTc	Mean (SD)	-3 (10.7)	13 (14.5)	16.8 (12.5, 21.1)	<0.0001
			>60ms, n (%)	0/66 (0%)	0/71 (0%)	-	-
Last	Day-0	QTc	Mean (SD)	420 (14.1)	422 (15.7)	1.5 (-3.5, 6.6)	0.55
			Range	373-453	386-460		
			>480ms, n (%)	0/67 (0%)	0/70 (0%)	-	-
			>500ms, n (%)	0/67 (0%)	0/70 (0%)	-	-
	Day-2 + 4hr		Mean (SD)	422 (16.7)	438 (17.9)	15.5 (9.6, 21.4)	<0.0001
			Range	386-498	405-485		
			>480ms, n (%)	1/66 (2%)	1/69 (1%)	1.02 (0.07, 15.84)	0.99
			>500ms, n (%)	0/66 (0%)	0/69 (0%)	-	-
	from Day-0 last course	dQTc	Mean (SD)	2 (13.5)	16 (12.7)	13.8 (9.3, 18.3)	<0.0001
			>60ms, n (%)	1/66 (2%)	0/69 (0%)	-	-
	from Day-0 first course	dQTc	Mean (SD)	-3 (17.1)	11 (15.2)	14.2 (8.3, 20.1)	<0.0001
			>60ms, n (%)	1/58 (2%)	0/60 (0%)	-	-
All visits	Day-0	QTc	Mean (SD)	424 (15.0)	424 (15.7)	-0.4 (-4.4, 3.6)	0.83
			Range	373-468	386-460		
			>480ms, n (%)	0/91 (0.0%)	0/88 (0.0%)		
			>500ms, n (%)	0/91 (0.0%)	0/88 (0.0%)		
	Day-2 + 4hr		Mean (SD)	424 (15.6)	441 (18.6)	16.9 (12.6, 21.3)	<0.0001
			Range	377-498	397-494		
			>480ms, n (%)	1/90 (1.0%)	6/88 (6.0%)	6.54 (0.81, 53.04)	0.079
			>500ms, n (%)	0/90 (0.0%)	0/88 (0.0%)		
	from Day-0 current course	dQTc	Mean (SD)	0 (12.2)	17 (14.3)	17.4 (14.9, 19.9)	<0.0001
			>60ms, n (%)	1/90 (1.1)	0/88 (0.0)		
	from Day-0 first course	dQTc	Mean (SD)	-2 (13.4)	15 (15.2)	16.9 (13.4, 20.4)	<0.0001
			>60ms, n (%)	1/80 (1.3)	0/78 (0.0)		
Difference in dQTc between courses	Middle v first course	Diff dQTc	Mean (SD)	-2 (10.9) p=0.074	-5 (18.9) p=0.032	-2.5 (-7.7, 2.8)	0.35
	Last v first course	Diff dQTc	Mean (SD)	-2 (15.5) p=0.25	-7 (17.9) p=0.0034	-4.8 (-10.9, 1.3)	0.12

*Linear mixed model with arm, HIV status, site and visit as fixed effects and participant as random effect. ECG=electrocardiogram. QTc: QT interval corrected for heart rate using study-derived correction factor (see appendix p 3). CTX=cotrimoxazole. DP=dihydroartemisinin-piperaquine. MD=mean difference. RR=Risk ratio. SD=standard deviation. Ms=milliseconds. CI=confidence interval.

Supplemental figures

Figure S1: Primary endpoint and other secondary maternal endpoints (per protocol population)

Maternal Outcomes	CTX+DP, N, events/PY (IR) or n/N (%)*	CTX, N, events/PY (IR) or n/N (%)*	Crude RR or IRR (95% CI)	P-value	Adjusted RR or IRR (95% CI)	P-value
Outcome						
Any malaria during pregnancy or at delivery (primary outcome) (n/N [%])	28/415 (6.7%)	66/426 (15.5%)	RR 0.44 (0.29, 0.66)	0.0001	RR 0.45 (0.29, 0.68)	0.0001
Peripheral malaria during pregnancy or at delivery (any measure) (incidence/100py)	415, 29/122 (23.8)	426, 99/128 (77.5)	IRR 0.30 (0.20, 0.46)	<0.0001	IRR 0.30 (0.20, 0.46)	<0.0001
Peripheral or placental malaria during pregnancy or at delivery (PCR) (incidence/100py)	415, 20/122 (16.4)	425, 92/128 (72.1)	IRR 0.22 (0.14, 0.36)	<0.0001	IRR 0.22 (0.14, 0.36)	<0.0001
Peripheral malaria during pregnancy or at delivery (microscopy) (incidence/100py)	415, 14/122 (11.5)	425, 60/128 (47.0)	IRR 0.24 (0.13, 0.42)	<0.0001	IRR 0.24 (0.13, 0.42)	<0.0001
Peripheral malaria during pregnancy (any measure) (incidence/100py)	415, 21/122 (17.2)	426, 82/128 (64.2)	IRR 0.26 (0.16, 0.42)	<0.0001	IRR 0.26 (0.16, 0.42)	<0.0001
Peripheral malaria during pregnancy (PCR) (incidence/100py)	414, 13/122 (10.7)	425, 70/128 (54.9)	IRR 0.19 (0.11, 0.34)	<0.0001	IRR 0.19 (0.11, 0.34)	<0.0001
Peripheral malaria during pregnancy (microscopy) (incidence/100py)	415, 10/122 (8.2)	425, 46/128 (36.1)	IRR 0.22 (0.11, 0.44)	<0.0001	IRR 0.22 (0.11, 0.44)	<0.0001
Peripheral malaria at delivery (any measure) (n/N [%])	10/410 (2.4%)	25/423 (5.9%)	RR 0.42 (0.20, 0.85)	0.02	RR 0.44 (0.22, 0.90)	0.03
Peripheral malaria at delivery (PCR) (n/N [%])	7/355 (2.0%)	18/350 (5.1%)	RR 0.40 (0.17, 0.94)	0.03	RR 0.41 (0.17, 0.95)	0.04
Peripheral malaria at delivery (microscopy) (n/N [%])	5/391 (1.3%)	16/405 (4.0%)	RR 0.33 (0.12, 0.90)	0.03	RR 0.36 (0.13, 0.97)	0.04
Clinical malaria during pregnancy (incidence/100py)	415, 1/122 (0.8)	426, 11/128 (8.6)	IRR 0.09 (0.01, 0.73)	0.02	IRR 0.09 (0.01, 0.73)	0.02
Placental malaria by histology (active and past) (n/N [%])	46/377 (12.2%)	72/381 (18.9%)	RR 0.62 (0.44, 0.86)	0.004	RR 0.62 (0.46, 0.85)	0.003
Placental malaria by histology (active) (n/N [%])	2/376 (0.5%)	13/381 (3.4%)	RR 0.16 (0.04, 0.69)	0.01	RR 0.16 (0.04, 0.70)	0.01
Placental malaria by histology (past) (n/N [%])	44/377 (11.7%)	59/381 (15.5%)	RR 0.72 (0.50, 1.02)	0.06	RR 0.84 (0.60, 1.17)	0.31
Placental malaria by histology (active - acute) (n/N [%])	2/376 (0.5%)	5/381 (1.3%)	RR 0.39 (0.08, 1.97)	0.25	RR 0.39 (0.08, 2.00)	0.26
Placental malaria by histology (active - chronic) (n/N [%])	0/376 (0.0%)	8/381 (2.1%)	NA	.	NA	.
Placental malaria (any measure) (n/N [%])	50/377 (13.3%)	78/381 (20.5%)	RR 0.63 (0.46, 0.86)	0.004	RR 0.63 (0.47, 0.84)	0.002
Peripheral or placental malaria at delivery (any measure) (n/N [%])	11/410 (2.7%)	32/423 (7.6%)	RR 0.36 (0.18, 0.69)	0.002	RR 0.38 (0.19, 0.74)	0.004
Placental inflammation or chorioamnionitis (n/N [%])	52/384 (13.5%)	45/386 (11.7%)	RR 1.21 (0.86, 1.71)	0.28	RR 1.22 (0.86, 1.74)	0.26
Maternal anaemia at delivery or third trimester (Hb<11 g/dL) (n/N [%])†	211/394 (53.6%)	213/410 (52.0%)	RR 1.02 (0.90, 1.16)	0.76	RR 1.00 (0.89, 1.14)	0.95
Maternal anaemia at delivery or third trimester (Hb<9 g/dL) (n/N [%])†	81/394 (20.6%)	70/410 (17.1%)	RR 1.17 (0.88, 1.56)	0.27	RR 1.17 (0.89, 1.56)	0.26
Maternal anaemia at delivery or third trimester (Hb<7 g/dL) (n/N [%])†	15/394 (3.8%)	13/410 (3.2%)	RR 1.14 (0.55, 2.36)	0.72	RR 1.19 (0.58, 2.43)	0.63
Maternal mid-upper-arm circumference <23cm at delivery (n/N [%])	44/413 (10.7%)	38/423 (9.0%)	RR 1.18 (0.79, 1.76)	0.43	RR 1.27 (0.85, 1.89)	0.25
Evidence of antibodies against SARS-Cov-2	19/382 (5.0%)	15/389 (3.9%)	RR 1.26 (0.65, 2.43)	0.49	RR 1.36 (0.72, 2.56)	0.35

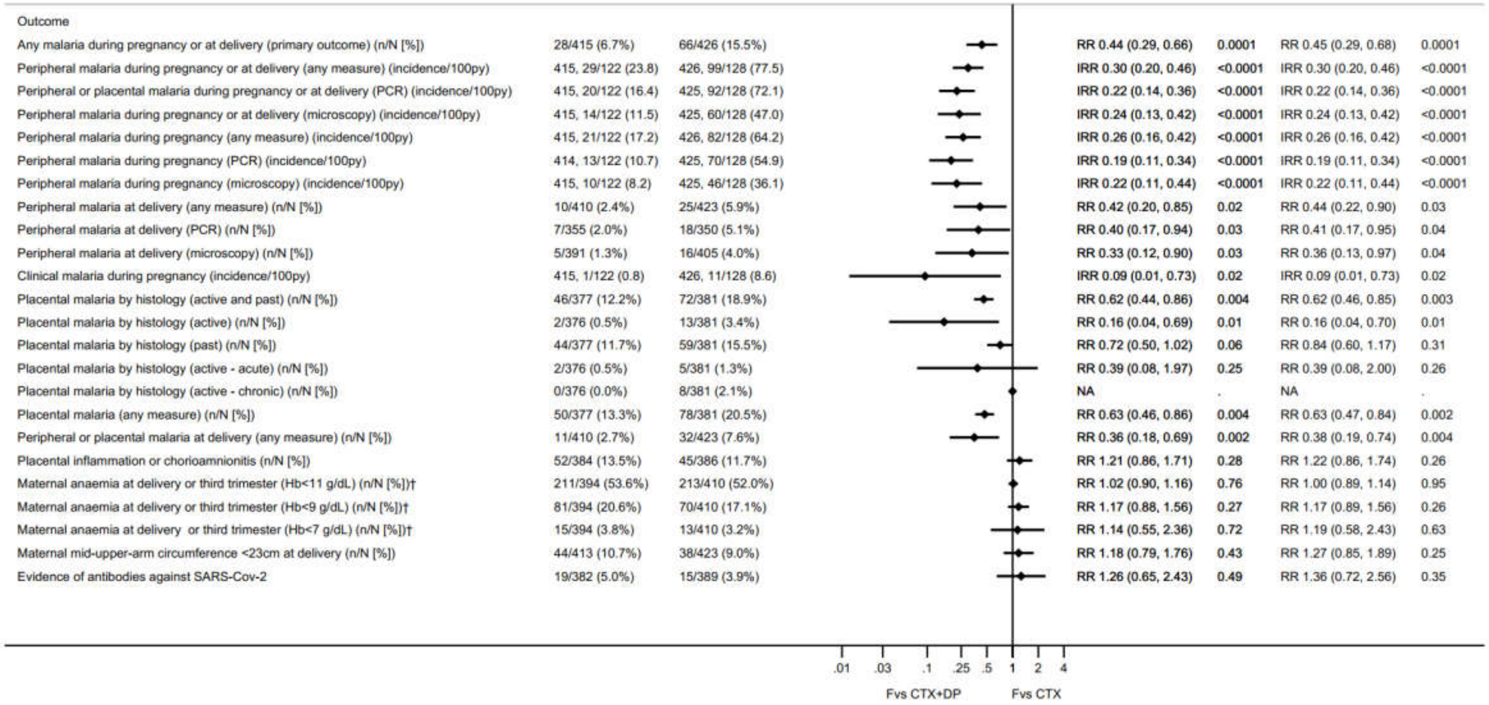

The crude RR values were obtained from log-binomial regressions with the stratification factors site, and HIV status included as covariates. The adjusted RR values were adjusted for site and HIV status, and five other prespecified covariates. The term malaria refers to malaria infection. Clinical malaria refers to malaria infection detected by mRDT (HRP2 or pLDH) or microscopy in conjunction with documented fever (>37.5 oC) or reported fever in the past 48 hours. CI=confidence interval. CTX=cotrimoxazole. DP=dihydroartemisinin-piperaquine. Fvs=favours. g/dL=grams per decilitre. Hb=haemoglobin concentration. IR=incidence rate per 100 person years. IRR=incidence rate ratio. ITT=intention-to-treat. PCR=polymerase chain reaction. PY=person-year. RR=risk ratio. *Data are N, events/person-years (incidence rate) or n/N (%).†Hb at delivery, or otherwise in the 3rd trimester if the delivery Hb was unavailable.

FIGURE S2: SENSITIVITY ANALYSIS OF THE PRIMARY ENDPOINT WITH MISSING ENDPOINTS IMPUTED (INTENTION TO TREAT POPULATION)

Outcome	CTX+DP, n/N(%)	CTX, n/N(%)	Crude RR (95% CI)	P-value	Adjusted RR (95% CI)	P-value
Cumulative incidence of malaria (primary analysis)	443	452	0.45 (0.30, 0.67)	0.0001	0.45 (0.31, 0.68)	0.0001
Cumulative incidence of malaria (imputed missing values)	447	456	0.45 (0.30, 0.67)	0.0001	0.45 (0.30, 0.67)	0.0001

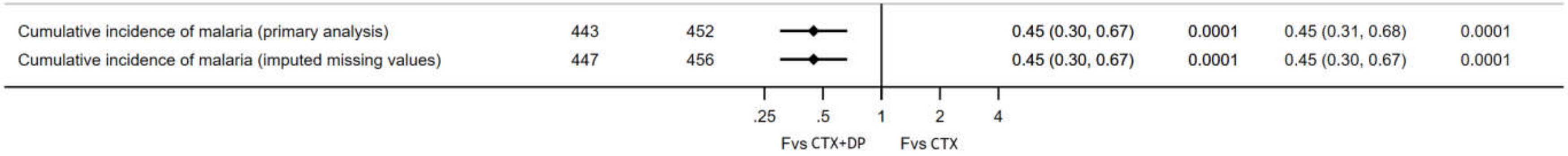

The crude RR values were obtained from log-binomial regressions with the stratification factors site, and HIV status included as covariates. The adjusted RR values were adjusted for site and HIV status, and five other prespecified covariates. The term malaria refers to malaria infection. CTX=cotrimoxazole. DP=dihydroartemisinin-piperaquine. ITT=intention to treat. RR=risk ratio. CI=confidence interval. Fvs=favours.

FIGURE S3: SUBGROUP ANALYSIS OF THE PRIMARY ENDPOINT (MODIFIED INTENTION TO TREAT POPULATION)

Group	Subgroup	CTX+DP n/N(%)	CTX n/N(%)	Crude RR (95% CI)	P-value (interaction)
Country					
	Kenya	25/344 (7.3%)	54/349 (15.5%)	0.46 (0.29, 0.72)	0.82
	Malawi	6/99 (6.1%)	16/103 (15.5%)	0.41 (0.17, 0.99)	
Study site					
	Kenya - Ahero	9/122 (7.4%)	28/123 (22.8%)	0.32 (0.16, 0.65)	0.31
	Kenya - Akala	8/101 (7.9%)	17/101 (16.8%)	0.46 (0.21, 1.01)	
	Kenya - Rabour	8/121 (6.6%)	9/125 (7.2%)	0.92 (0.37, 2.30)	
	Malawi - Madziabango	0/11 (0.0%)	2/11 (18.2%)	NA	
	Malawi - Mpemba	5/36 (13.9%)	8/39 (20.5%)	0.68 (0.25, 1.88)	
	Malawi - Zomba	1/52 (1.9%)	6/53 (11.3%)	0.17 (0.02, 1.36)	
Malaria transmission intensity					
	Moderate	16/229 (7.0%)	40/226 (17.7%)	0.38 (0.22, 0.66)	0.34
	High	15/214 (7.0%)	30/226 (13.3%)	0.56 (0.31, 1.00)	
Malaria status by microscopy					
	Negative	5/40 (12.5%)	12/39 (30.8%)	0.40 (0.15, 1.06)	0.042
	Positive	26/403 (6.5%)	58/413 (14.0%)	0.46 (0.30, 0.71)	
Season					
	Dry	14/157 (8.9%)	23/139 (16.5%)	0.51 (0.28, 0.95)	0.57
	Intermediate	6/140 (4.3%)	24/159 (15.1%)	0.29 (0.12, 0.69)	
	Wet	11/146 (7.5%)	23/154 (14.9%)	0.49 (0.25, 0.95)	
Gravidity					
	Paucigravidae	9/114 (7.9%)	25/127 (19.7%)	0.44 (0.21, 0.90)	0.53
	Multigravidae	22/329 (6.7%)	45/325 (13.8%)	0.47 (0.29, 0.76)	
Gestational age at enrolment					
	<median (21 wks)	17/222 (7.7%)	42/228 (18.4%)	0.38 (0.22, 0.64)	0.13
	>=median (21 wks)	14/221 (6.3%)	28/224 (12.5%)	0.50 (0.27, 0.92)	
HIV status					
	Known positive	25/368 (6.8%)	53/376 (14.1%)	0.48 (0.31, 0.76)	0.52
	Newly diagnosed	6/75 (8.0%)	17/76 (22.4%)	0.35 (0.15, 0.82)	
Social-Economic Status (SES)					
	Low	11/148 (7.4%)	32/150 (21.3%)	0.35 (0.18, 0.65)	0.085
	Medium	10/150 (6.7%)	23/151 (15.2%)	0.43 (0.21, 0.85)	
	High	10/145 (6.9%)	15/151 (9.9%)	0.69 (0.32, 1.47)	
Number of IPTp courses received					
	1-2	1/19 (5.3%)	0/21 (0.0%)	NA	0.87
	3-4	12/168 (7.1%)	32/178 (18.0%)	0.40 (0.21, 0.77)	
	5+	18/256 (7.0%)	38/253 (15.0%)	0.44 (0.26, 0.75)	

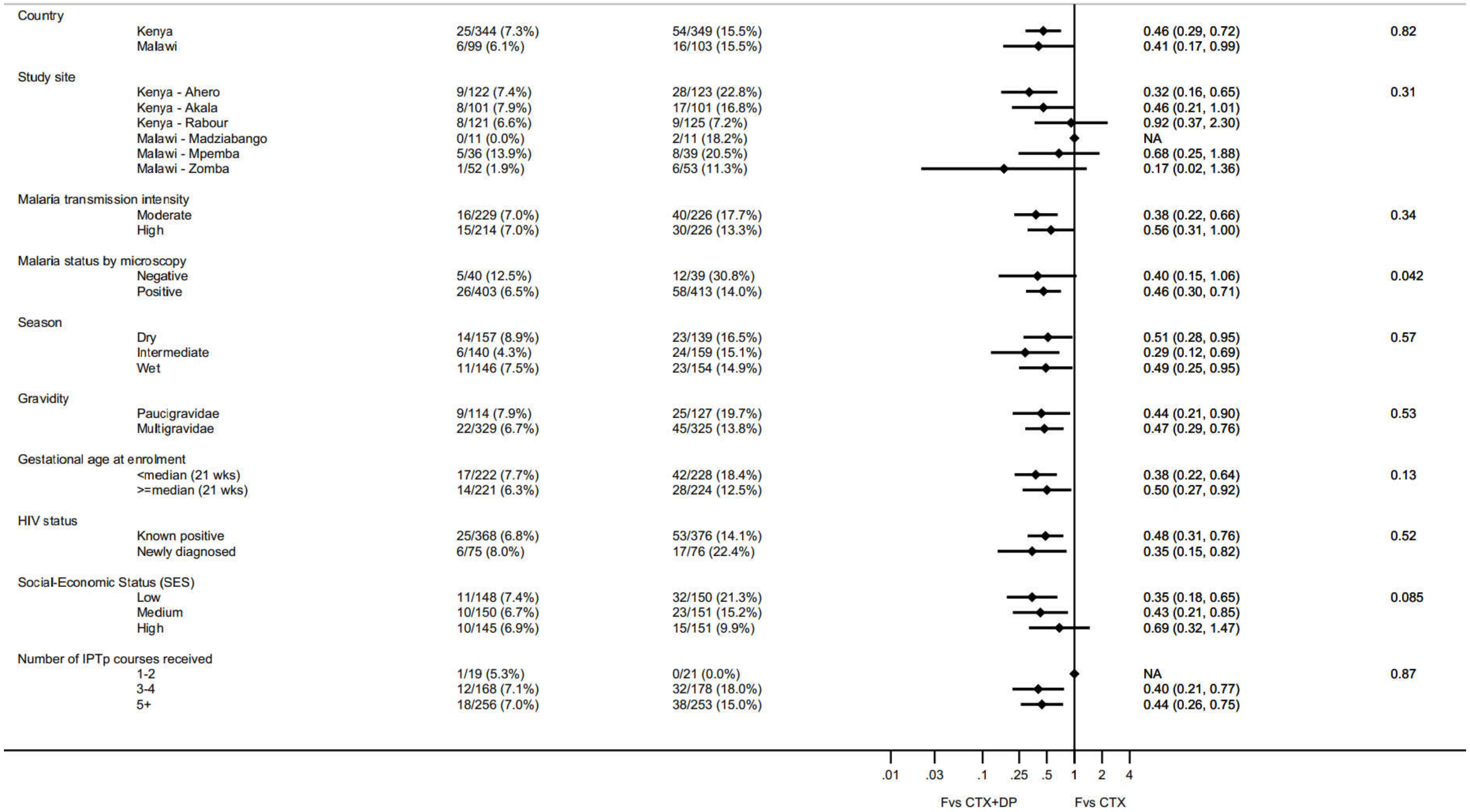

The crude RR values were obtained from log-binomial regressions with the stratification factors site, and HIV status included as covariates. CTX=cotrimoxazole. DP=dihydroartemisinin-piperaquine. ITT=intention to treat. RR=risk ratio. CI=confidence interval. Fvs=favours.

FIGURE S4: OTHER MATERNAL SECONDARY EFFICACY ENDPOINTS (MODIFIED INTENTION TO TREAT POPULATION)

Pregnancy and Newborn Outcomes	CTX+DP, n/N(%)	CTX, n/N(%)	Crude RR (95% CI)	P-value	Adjusted RR (95% CI)	P-value
Outcome						
Peripheral malaria during pregnancy or at delivery (any measure) (cumulative risk) *	30/443 (6.8%)	66/452 (14.6%)	**0.46 (0.31, 0.69)**	**0.0002**	0.47 (0.31, 0.70)	0.0002
Peripheral or placental malaria during pregnancy or at delivery (PCR) (cumulative risk)	21/440 (4.8%)	58/448 (12.9%)	**0.37 (0.23, 0.59)**	**<0.0001**	0.38 (0.23, 0.61)	0.0001
Peripheral malaria during pregnancy or at delivery (microscopy) (cumulative risk)	16/440 (3.6%)	50/448 (11.2%)	**0.32 (0.19, 0.56)**	**<0.0001**	0.33 (0.19, 0.57)	0.0001
Peripheral malaria during pregnancy or at delivery (RDT) (cumulative risk)	4/443 (0.9%)	9/452 (2.0%)	**0.45 (0.14, 1.44)**	**0.18**	0.43 (0.14, 1.39)	0.16
Peripheral malaria during pregnancy (any measure) (cumulative risk)	23/443 (5.2%)	58/450 (12.9%)	**0.40 (0.25, 0.63)**	**0.0001**	0.40 (0.25, 0.63)	0.0001
Peripheral malaria during pregnancy (PCR) (cumulative risk)	15/439 (3.4%)	49/448 (10.9%)	**0.31 (0.18, 0.54)**	**<0.0001**	0.32 (0.18, 0.56)	0.0001
Peripheral malaria during pregnancy (microscopy) (cumulative risk)	12/440 (2.7%)	37/448 (8.3%)	**0.33 (0.17, 0.62)**	**0.0005**	0.33 (0.18, 0.63)	0.0007
Peripheral malaria during pregnancy (RDT) (cumulative risk)	3/442 (0.7%)	9/450 (2.0%)	**0.34 (0.09, 1.24)**	**0.10**	0.32 (0.09, 1.18)	0.087
Clinical malaria during pregnancy (cumulative risk)	3/443 (0.7%)	10/450 (2.2%)	**0.31 (0.09, 1.10)**	**0.069**	0.29 (0.08, 1.04)	0.058
Maternal anaemia in the third trimester (Hb<11 g/dL) (n/N [%])	244/410 (59.5%)	237/420 (56.4%)	**1.05 (0.94, 1.18)**	**0.35**	1.03 (0.93, 1.15)	0.55
Maternal anaemia in the third trimester (Hb<9 g/dL) (n/N [%])	87/410 (21.2%)	80/420 (19.0%)	**1.11 (0.85, 1.44)**	**0.46**	1.11 (0.86, 1.45)	0.42
Maternal anaemia in the third trimester (Hb<7 g/dL) (n/N [%])	16/410 (3.9%)	11/420 (2.6%)	**1.46 (0.69, 3.10)**	**0.32**	1.48 (0.71, 3.11)	0.30
Maternal anaemia at delivery (Hb<11 g/dL) (n/N [%])	91/190 (47.9%)	84/176 (47.7%)	**1.01 (0.82, 1.25)**	**0.91**	1.04 (0.84, 1.29)	0.69
Maternal anaemia at delivery (Hb<9 g/dL) (n/N [%])	45/190 (23.7%)	25/176 (14.2%)	**1.71 (1.10, 2.66)**	**0.018**	1.70 (1.09, 2.65)	0.019
Maternal anaemia at delivery (Hb<7 g/dL) (n/N [%])	8/190 (4.2%)	5/176 (2.8%)	**1.52 (0.51, 4.55)**	**0.45**	1.63 (0.53, 4.98)	0.39

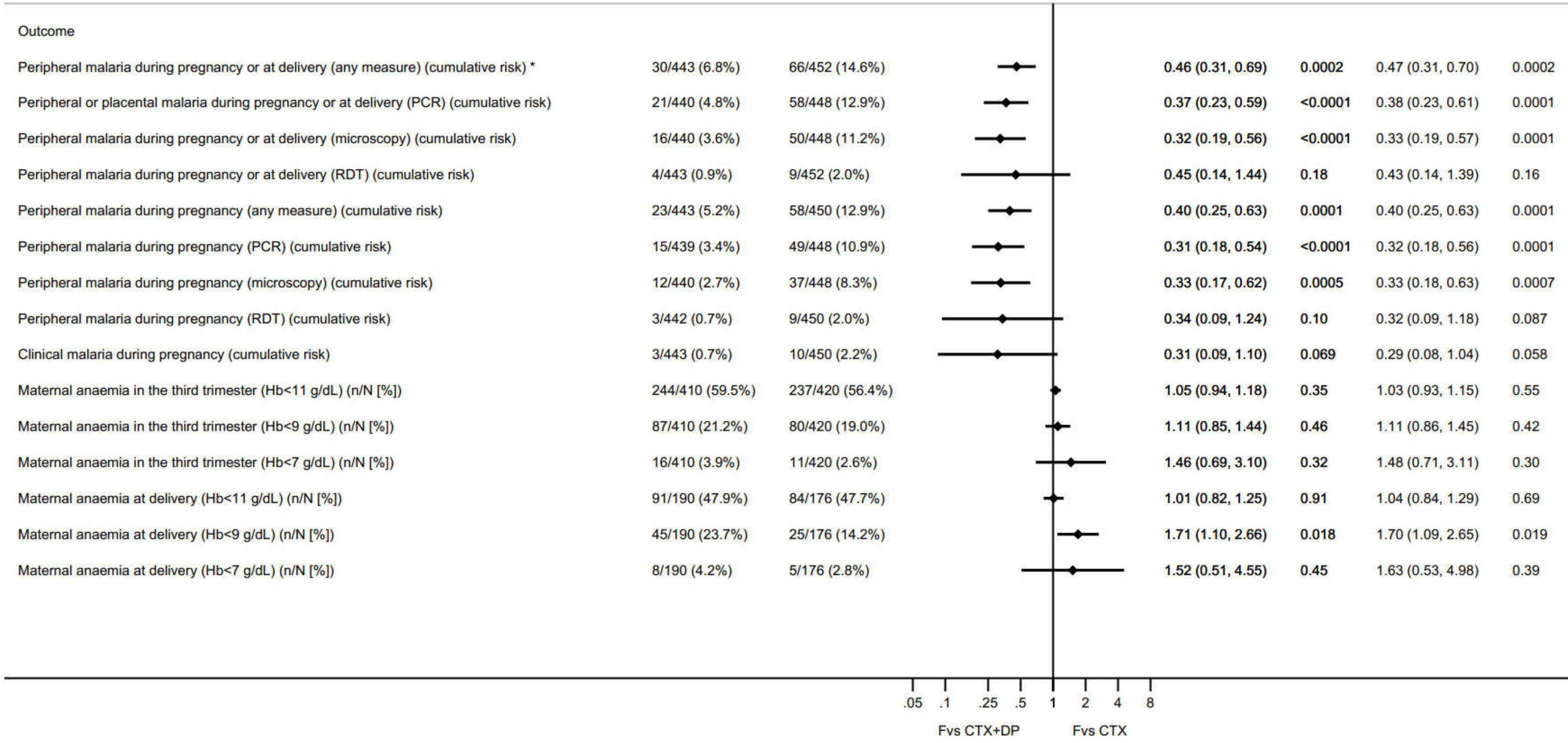

The crude RR values were obtained from log-binomial regressions with the stratification factors site, and HIV status included as covariates. CTX=cotrimoxazole. DP=dihydroartemisinin-piperaquine. The term malaria refers to malaria infection. Clinical malaria refers to malaria infection detected by mRDT (HRP2 or pLDH) or microscopy in conjunction with documented fever (>37.5 oC) or reported fever in the past 48 hours. ITT=intention to treat. RR=risk ratio. CI=confidence interval. PCR=polymerase chain reaction. Fvs=favours.

Chemoprevention with monthly IPTp with dihydroartemisinin-piperaquine for malaria in HIV-infected pregnant participants on daily cotrimoxazole in Kenya and Malawi: a multi-centre placebo-controlled trial

Short Title: Improving PRegnancy Outcomes with PReVEntive therapy in Africa-2 (IMPROVE-2)

Trial Identifiers:		
Kenya SERU<br>178/3784	Malawi COMREC<br>01/19/2578	UK LSTM REC<br>17-005
KPPB<br>[#####]	PMPB<br>[#####]	Clinicaltrials.gov<br>[#####]

Chief Investigator: Prof Feiko ter Kuile, MD, PhD, LSTM, Address: Liverpool School of Tropical Medicine, Pembroke Place, Liverpool L3 5QA, UK, Mobile: +44 (0)7846 377 369 (UK), +254 (0)708 739 228 (Kenya); Email: feiko.terkuile@lstmed.ac.uk

Co-Principal Investigators:
Malawi: *Dr Mwayiwawo Madanitsa*, MD, PhD, College of Medicine, University of Malawi, Private Bag 360, Chichiri, Blantyre 3, Mobile: +265(0) 999 616 537; E-mail: mmadanitsa@medcol.mw, mwayi.madanitsa@gmail.com

Kenya: a) *Dr Hellen Barsosio*, MD, KEMRI Centre for Global Health Research (CGHR) and CDC Collaboration, P.O. Box 1578, Kisumu 40100. Mobile: +254 719 436 833; E-mail: hellen.barsosio@lstmed.ac.uk

b) *Dr Simon Kariuki*, PhD, KEMRI Centre for Global Health Research (CGHR) and CDC Collaboration, P.O. Box 1578, Kisumu 40100. Mobile: + 254 725 389 246; E-mail: SKariuki@kemricdc.org

Co-investigators: See page 9 for investigators and page 73 for the role of investigators

Funder: Joint Global Health Trials Scheme, Medical Research Council/Wellcome Trust/DFID, UK; and the European and Developing Countries Clinical Trials Partnership (EDCTP)

Sponsor: Liverpool School of Tropical Medicine (LSTM); Pembroke Place, Liverpool L3 5QA, UK, Phone: +44 0151 7053794; Email: lstmgov@lstmed.ac.uk

Revision chronology:

Version and date of protocol	Details of Changes	Authors (see also page 9)	Signature Chief Investigator
v1.0-17Apr19	*Original*	*HB, MM, CB, EMH, JD, FtK*	

Confidentiality Statement: This document contains confidential information that must not be disclosed to anyone other than the sponsor, the investigator team, host institution, relevant ethics committee and regulatory authorities

DESCRIPTION OF AMENDMENTS

DETAILS OF KEY CHANGES TO PROTOCOL VERSION

None yet

TABLE OF CONTENT

1 Abbreviations 7

2 Administrative information 9
2.1 Title 9
2.2 Investigators and collaborators 9
2.2.1 Chief Investigator 9
2.2.2 Co-Principal investigators 9
2.2.3 Co-investigators 9
2.2.4 Collaborators 9
2.2.5 Institutions 9
2.3 Trial registration and protocol summaries 10
2.3.1 Trial Registration data 10
2.3.2 Lay summary 12
2.3.3 Short technical protocol summary 13
2.3.4 Long technical protocol summary 14

3 Introduction 19
3.1 The burden malaria and HIV and viral infections in pregnancy 19
3.1.1 Malaria and HIV in pregnancy 19
3.1.2 Viral co-infections in pregnancy 19
3.2 Malaria prevention in HIV-infected women 19
3.2.1 Antifolate resistance 19
3.2.2 Chemoprevention with Dihydroartemisinin-Piperaquine (DP) 20
3.2.3 DP and other viral infections 20
3.2.4 Recent changes in first-line combination antiretroviral therapy (cART) from efavirenz (EFV) to dolutegravir (DTG) based cARTs 21
3.3 Drug-drug interactions 23
3.3.1 Drug-drug interaction between DP and dolutegravir 23
3.4 Safety of study drugs in pregnancy 23
3.4.1 CTX 23
3.4.2 Dihydroartemisinin-Piperaquine 23
3.4.3 Dolutegravir (DTG) 24

4 Justification for the study 25
4.1 Why is this study needed now? 25
4.2 Other relevant research ongoing elsewhere 25
4.2.1 HIV-infected women 25
4.2.2 HIV-uninfected women 25
4.3 Hypotheses 25

5 Aim & Objectives 26
5.1 Primary objective 26
5.2 Secondary objectives 26

6 Trial Design & design considerations 26
6.1 Overview of design 26
6.2 Design considerations 27
6.2.1 Rational for a fixed dose rather than a dose based on bodyweight 27
6.2.2 Rationale for nested pharmacokinetic studies 27
6.2.3 Design as a superiority trial 27

7 Methods: Participants, interventions, and outcomes 28
7.1 Study setting 28

7.1.1 Kenya ... 28
7.1.2 Malawi ... 28
7.2 Eligibility criteria ... 29
7.2.1 Inclusion criteria ... 29
7.2.2 Exclusion criteria ... 29
7.3 Interventions ... 29
7.3.1 Trial Medications and interventions ... 29
7.3.2 Criteria for discontinuing or modifying allocated interventions for a given trial participant ... 32
7.3.3 Antiretroviral regimens ... 33
7.3.4 Permitted and prohibited concomitant medication and care ... 34
7.3.5 Strategies to improve adherence to study protocol and medication ... 34
7.3.6 Removal of participants from study ... 35
7.4 Outcome measures ... 36
7.4.1 Primary outcome ... 36
7.4.2 Secondary outcome measures ... 36
7.5 Participants timeline ... 37
7.5.1 Timeline ... 37
7.5.2 Antenatal booking visit ... 37
7.5.3 Interim monthly scheduled visits ... 38
7.5.4 Unscheduled visits during pregnancy ... 39
7.5.5 Delivery visit ... 39
7.5.6 Postnatal visits ... 40
7.6 Sample size and power ... 41
7.6.1 Primary outcome ... 41
7.6.2 Power calculations for key secondary outcomes ... 41
7.6.3 Sample size for cardiac monitoring study ... 42
7.7 Recruitment and retention ... 42
7.7.1 Recruitment procedures ... 42
7.7.2 Planned recruitment rate ... 42
7.7.3 Rate of loss to follow-up ... 42
7.7.4 Problems with compliance and Strategies for retention ... 42
8 Methods: Assignment of interventions ... 43
8.1 Allocation ... 43
8.2 Blinding and unblinding ... 43
8.2.1 Blinding ... 43
8.2.2 Emergency unblinding ... 44
9 Methods Data collection, management and analysis ... 44
9.1 measurement of efficacy outcomes and safety ... 44
9.1.1 Efficacy ... 44
9.1.2 Safety ... 45
9.1.3 Pharmacokinetic studies and drug-drug interactions ... 46
9.1.4 Laboratory assays ... 46
9.2 Data collection methods & storage ... 47
9.2.1 Data management ... 47
9.3 Statistical methods ... 48
9.3.1 General principles ... 48
9.3.2 Analyses populations ... 48
9.3.3 Primary outcome analyses ... 48
9.3.4 Secondary outcomes analyses ... 49
9.3.5 Subgroup analyses ... 49

9.3.6 Safety 49
9.3.7 Interim sample size re-estimation and/or interim analyses and criteria for termination of the trial 49

10 Methods: Monitoring 50
10.1 Data monitoring and trial oversight committees 50
10.1.1 Data Monitoring and Ethics Committee (DMEC) 50
10.1.2 Trial Steering Committee (TSC) 50
10.2 Safety Monitoring and reporting 51
10.2.1 Definitions 51
10.2.2 Identifying, managing adverse events 52
10.2.3 Assessment of causality 52
10.2.4 Reporting adverse event procedures 52
10.3 Trial monitoring and auditing 53
10.3.1 Trial monitoring 53
10.3.2 Auditing 54
10.3.3 Role of sponsor 54
10.4 Other quality control measures 54
10.4.1 Safety monitors 54
10.4.2 Trial monitoring 54
10.4.3 Training 54
10.4.4 Quality assurance/control of laboratory tests 54

11 Ethics and dissemination 55
11.1 Declaration of Helsinki 55
11.2 Research ethics and regulatory approval 55
11.2.1 Review process 55
11.2.2 Protocol amendments 55
11.2.3 Sanctioning of the protocol by hospitals 55
11.2.4 Regulatory approval 55
11.3 Informed consent procedures 56
11.3.1 Consent procedures 56
11.4 Protection of privacy and confidentiality 56
11.4.1 Privacy 56
11.4.2 Privacy of individual 56
11.4.3 Confidentiality of data 57
11.5 Declaration of interest 57
11.6 Access to source data/documents 57
11.7 Risks and benefits 57
11.7.1 Risks to study participants 57
11.7.2 Benefits to study participants 58
11.7.3 Risk to the population 58
11.7.4 Other ethical considerations 58
11.8 Ancillary and post-trial care 59
11.8.1 Health care during the trial 59
11.8.2 Trial insurance 59
11.8.3 Post-trial care 59
11.9 Expenses reimbursement and incentives 59
11.10 Dissemination and application of the results 60
11.10.1 Result dissemination and publication policy 60
11.10.2 Impact 61
11.10.3 Authorship and publications 62
11.10.4 Data Sharing Statement 62

12 Timeframe and duration of the study 62
12.1 Timeline 62
12.1.1 Project Gantt chart 63
13 References 63
14 Financial aspects and conflict of interest 72
14.1 Funding for the trial 72
14.2 Provision of study drugs 72
15 Budget & budget justification 72
16 Appendices 73
16.1 Appendix I. Role of Investigators and collaborators 73
16.1.1 Definition investigator and collaborator 73
16.1.2 Protocol development: authors' contributions 73
16.1.3 Role Investigators 73
16.1.4 Collaborators 74
16.2 Appendix II. Terms of reference oversight committees 75
16.2.1 Trial Management Group (TMG) 75
16.2.2 Trial Steering Committee (TSC) 75
16.2.3 Data Monitoring and Ethics Committee (DMEC) 77
16.3 Appendix III. Declaration of Helsinki 79
16.4 Appendix IV. Budget and budget justification 82
16.4.1 Budget 82
16.4.2 Budget Justification 82
16.5 Appendix V. Participant information sheets and informed consent statements 83
16.5.1 Participant Information Sheet: Main trial (all participants) (English) 83
16.5.2 Consent statement for screening and participation in the main trial (all participants) (English) 83
16.5.3 Consent Statement for long term storage of samples and use for future research (all participants) (English) 83
16.5.4 Participant Information Sheet for cardiac safety monitoring (subgroup) (English) 83
16.5.5 Consent Statement for cardiac safety monitoring (sub-group) (English) 83
16.6 Appendix VI. Description of other clinical and laboratory methods 84
16.6.1 Pharmacokinetic studies of drug-drug interactions between piperaquine and standard antiretroviral therapy 84
16.6.2 Impact of antimalarial resistance on protective efficacy 85
16.6.3 Impact on maternal antibody, trans-placental antibody transfer and multi-pathogen neonatal cell-mediated immune responses 86
16.6.4 Impact on infant adaptive and innate-like immunity 86
16.6.5 Impact on biomarkers of placental function and adverse birth outcomes 87
16.6.6 Impact on arboviral co-infections 88
16.6.7 Use of mobile phone Unstructured Supplementary Service Data (USSD) platform to enhance protocol adherence and monitoring of tolerance 88
16.6.8 Performance of a highly sensitive diagnostic test for malaria 89
16.7 Appendix VII. Product characteristics 90
16.7.1 Dihydroartemisinin-piperaquine product insert (D-ARTEPP, Guilin Pharma) 90
16.7.2 Dihydroartemisinin-piperaquine product insert (Eurartesim, AlfaSigma) 92
16.7.3 Cotrimoxazole 98
16.7.1 Fixed dose combination of TLD (TDF/3TC/DTG) 100
16.8 Appendix VIII. SPIRIT 2013 checklist clinical trial protocol 106
16.9 Appendix IX. Questionnaires 111

1 ABBREVIATIONS

3TC	Lamivudine
95% CI	95 per cent Confidence Interval
ACT	Artemisinin-Based Combination Therapy
AE	Adverse Event
AIDS	Acquired Immunodeficiency Syndrome
AL	Artemether-Lumefantrine
ANC	Antenatal Care
ART	Antiretroviral Therapy
AUC	Area Under the Curve
AZT	Zidovudine
cART	Combination Antiretroviral Therapy
CDC	US Centers for Disease Control and Prevention
CHW	Community Health Worker
CHEW	Community Health Extension Worker
Cmax	Maximum Drug Concentration
COM	College of Medicine, University of Malawi
CRF	Case Record Form
CRO	Contract Research Organization
CT	Counselling and Testing
CTX	Cotrimoxazole
CYP450	Cytochrome P450
DHA	Dihydroartemisinin
DBS	Dried Blood Spot (On Filter Paper)
DP	Dihydroartemisinin-Piperaquine
DMEC	Data Monitoring and Ethics Committee
DS	Double Strength
DTG	Dolutegravir
ECG	Electrocardiogram
EDCTP	European And Developing Countries Clinical Trials Partnership
EFV	Efavirenz
ELISA	Enzyme-Linked Immunosorbent Assay
EMTCT	Elimination of Mother to Child Transmission
ERG	Evidence Review Group from WHO
FDA	Food and Drug Administration
GCP	Good Clinical Practice
GLM	Generalised Linear Model
GMP	Good Manufacturing Practice
Hb	Haemoglobin
HIV	Human Immunodeficiency Virus
IRB	Institutional Review Board
IRS	Indoor Residual Spraying
IPTp	Intermittent Preventive Treatment in Pregnancy
ITNs	Insecticide Treated Nets
ITT	Intention to Treat
KCMC	Kilimanjaro Christian Medical Centre
KEMRI	Kenya Medical Research Institute
LBW	Low Birthweight

LLINS	Long-Lasting Insecticide Treated Nets
LSHTM	London School of Hygiene and Tropical Medicine
LSTM	Liverpool School of Tropical Medicine
MDA	Mass Drug Administration
MoH	Ministry of Health
MPAC	Malaria Policy Advisory Committee
MRC	Medical Research Council, UK
MTCT	Mother to Child Transmission
NMCP	National Malaria Control Programme
NVP	Nevirapine
PCR	Polymerase Chain Reaction
PD	Pharmacodynamics
PK	Pharmacokinetics
PMTCT	Prevention of Mother to Child Transmission
PT	Preterm
PQ	Piperaquine
RCT	Randomised Controlled Trial
RDT	Rapid Diagnostic Test
REC	Research Ethics Committee
SAE	Serious Adverse Event
SGA	Small for Gestational Age
SMX-TMP	Sulfamethoxazole-Trimethoprim
SOP	Standard Operating Procedure
SP	Sulphadoxine-Pyrimethamine
SS	Single Strength
STI	Sexually Transmitted Infection
$T_{1/2}$	Plasma Half-Life
QTc	Rate Corrected Time Qt Interval on Electrocardiogram (ECG)
QTcB	Rate Corrected Time Qt Interval on ECG Using Bazett's Correction
QTcF	Rate Corrected Time Qt Interval on ECG Using Fridericia's Correction
TDF	Tenofovir Disoproxil Fumarate
TLD	Tenofovir Disoproxil Fumarate /Lamivudine/Dolutegravir Fixed Dose
TLE	Tenofovir Disoproxil Fumarate /Lamivudine/Efavirenz Fixed Dose
Tmax	Time to Maximum Plasma Concentration
TSC	Trial Steering Committee
VHV	Village Health Volunteer
VHW	Village Health Worker
VHEV	Village Health Extension Volunteer
VHEW	Village Health Extension Worker
WHO	World Health Organization

2 Administrative information

2.1 Title

Chemoprevention with monthly IPTp with dihydroartemisinin-piperaquine for malaria in HIV-infected pregnant participants on daily cotrimoxazole in Kenya and Malawi: a multi-centre placebo-controlled trial (IMPROVE-2)

2.2 Investigators and collaborators

2.2.1 Chief Investigator	*2.2.2 Co-Principal investigators*
Prof Feiko ter Kuile, MD, PhD[1,2]	Dr Hellen Barsosio, MD (Kenya)[1,2]
	Dr Simon Kariuki, PhD[2] (Kenya)
	Dr Mwayiwawo Madanitsa, MD, PhD[3] (Malawi)

2.2.3 Co-investigators

Prof Duolao Wang, PhD[1]	Prof Victor Mwapasa, MD, MPH, PhD[3]
Mr James Dodd, MSC[1]	Prof Kamija Phiri, MD, PhD[3]
Dr Titus Kwambai, MD[2]	Prof Ken Maleta, MD, PhD[3]
Dr George Githuka, MD, MSc[4]	Dr Clifford Banda[3]

2.2.4 Collaborators

Dr George Warimwe[5]	Prof Karen Barnes, PhD[10]
Dr Charles Nyaigoti[5]	Dr Ann Moormann, PhD[11]
Prof Philip Bejon, MD, PhD[5]	Prof Kevin Kain, MD, PhD[12]
Dr Aaron Samuels, MD[6]	Prof Stephen Rogerson, MBBS, PhD[13]
Dr Meghna Desai, PhD[6]	Dr Gonzalo Domingo, MD, PhD[14]
Dr Julie Gutman, MD, MSc[6]	Dr David Bell, PhD[15]
Dr Jenny Hill, PhD[1]	Dr Pascal Voiriot, MD[16]
Dr Emily Adams, PhD[1]	Dr Cristiana Cairo, PhD[17]
Ass. Prof Dr Michael Alifrangis, PhD[7]	Prof Miriam Laufer, PhD[17]
Assist Prof Matthew Chico, MPH[8]	Prof. Wilson Mandala[18]
Mrs Silke Fernandes[8]	Mr Manota Mphande[19]
Prof Kara Hanson[8]	Dr Maureen Kimani, MD, MPH[5]
Dr Eva Maria Hodel, PhD[1,4]	Dr Kigen Bartilol, MD MMeD (Obs/Gyn)[5]

2.2.5 Institutions

1 Liverpool School of Tropical Medicine, UK	11 University of Massachusetts, Worcester, USA
2 Kenya Medical Research Institute, Kenya	12 University of Toronto, Canada
3 College of Medicine, University of Malawi, Malawi	13 University of Melbourne, Melbourne, Australia
4 National AIDS and STI's Control Programme, Kenya	14 PATH, Seattle, Washington, USA
5 KEMRI-Wellcome Trust Research Programme	15 Intellectual Ventures Lab., Washington, USA
6 CDC, Atlanta, Georgia, USA	16 Cardiabase, Banook Group, Nancy, France
7 University of Copenhagen, Denmark	17 University of Maryland, Baltimore
8 London School of Hygiene and Trop. Medicine, UK	18 Malawi University of Science and Technology
9 University of Tampere, Finland	19 Logic Bay Systems Ltd, Malawi
10 University of Cape, South Africa	

2.3 TRIAL REGISTRATION AND PROTOCOL SUMMARIES

2.3.1 Trial Registration data

<table>
<tr><td>Data Category</td><td colspan="3">Information</td></tr>
<tr><td>Primary registry and trial identifying number</td><td colspan="3">Clinicaltrials.gov: [#####]</td></tr>
<tr><td>Date of registration in primary registry</td><td colspan="3">[#####]</td></tr>
<tr><td rowspan="2">Secondary identifying numbers</td><td>Kenya SERU
#178/3784</td><td>Malawi COMREC
#01/19/2578</td><td>UK LSTM REC
#17-005</td></tr>
<tr><td>KPPB
[#####]</td><td>PMPB
[#####]</td><td></td></tr>
<tr><td>Source(s) of monetary or material support</td><td colspan="3">Joint Global Health Trials Scheme, Medical Research Council/Wellcome Trust/DFID, UK; and the European and Developing Countries Clinical Trials Partnership (EDCTP)</td></tr>
<tr><td>Primary sponsor</td><td colspan="3">Liverpool School of Tropical Medicine (LSTM); Pembroke Place, Liverpool L3 5QA, UK, Phone: +44 0151 7053794; Email: lstmgov@lstmed.ac.uk</td></tr>
<tr><td>Secondary sponsor(s)</td><td colspan="3">NA</td></tr>
<tr><td>Contact for public queries</td><td colspan="3">Prof Feiko ter Kuile, LSTM, Pembroke Place, Liverpool, L3 5QA, UK, E-mail: feiko.terkuile@lstmed.ac.uk</td></tr>
<tr><td>Contact for scientific queries</td><td colspan="3">Prof Feiko ter Kuile, LSTM, Pembroke Place, Liverpool, L3 5QA, UK, E-mail: feiko.terkuile@lstmed.ac.uk</td></tr>
<tr><td>Public title</td><td colspan="3">Improving PRegnancy Outcomes with PReVEntive therapy in Africa-2 (IMPROVE-2)</td></tr>
<tr><td>Scientific title</td><td colspan="3">Chemoprevention with monthly IPTp with dihydroartemisinin-piperaquine for malaria in HIV-infected pregnant participants on daily cotrimoxazole in Kenya and Malawi: a multi-centre placebo-controlled trial</td></tr>
<tr><td>Countries of recruitment</td><td colspan="3">Malawi; Kenya</td></tr>
<tr><td>Health condition(s) or problem(s) studied</td><td colspan="3">Pregnancy; HIV; Malaria</td></tr>
<tr><td rowspan="2">Intervention(s)</td><td colspan="3">CTX-alone: Daily, one double-strength tablet of 160mg of sulfamethoxazole and 800mg of trimethoprim plus monthly placebo-DP, given as a fixed dose of 3 placebo-DP tablets daily for three days until delivery</td></tr>
<tr><td colspan="3">CTX-DP: Daily, one double-strength tablet of 160mg of sulfamethoxazole and 800mg of trimethoprim plus monthly DP, given as a fixed dose of 3 tablets (40 mg of dihydroartemisinin and 320 mg of piperaquine) daily for three days until delivery.</td></tr>
<tr><td rowspan="4">Study type</td><td colspan="3">Interventional</td></tr>
<tr><td colspan="3">Allocation: randomised; intervention model: parallel assignment; arms: 2; allocation ratio: 1:1; stratified by site (hospital) and HIV-status (known-positive and newly-diagnosed). Masking: Placebo controlled</td></tr>
<tr><td colspan="3">Primary purpose: Treatment and prevention</td></tr>
<tr><td colspan="3">Phase-III</td></tr>
<tr><td>Date first enrolment</td><td colspan="3">[dd mmm yyyy]</td></tr>
<tr><td>Target sample size</td><td colspan="3">898 (449 per arm) (359 contributors and 90 non-contributors)</td></tr>
<tr><td>Recruitment status</td><td colspan="3">Not yet recruiting</td></tr>
</table>

Data Category	Information
Primary Objective	To determine if monthly IPTp-DP in HIV-infected pregnant women receiving daily CTX is safe and superior to daily CTX-alone for controlling malaria infection in areas with high antifolates resistance to SP and CTX in Malawi and Kenya.
Key inclusion criteria	• HIV-infected pregnant women between 16-28 weeks' gestation<br>• Viable singleton pregnancy<br>• On or eligible for cARTs and CTX<br>• A resident of the study area<br>• Willing to adhere to scheduled and unscheduled study visit procedures<br>• Willing to deliver in a study clinic or hospital<br>• Provide written informed consent
Key exclusion criteria	• Multiple pregnancies (i.e. twin/triplets)<br>• HIV-negative or HIV status unknown<br>• Known heart ailment<br>• Severe malformations or non-viable pregnancy if observed by ultrasound<br>• Participants with advanced HIV-disease at WHO clinical stage 3 and 4<br>• Confirmed or suspected TB infection,<br>• Unable to give consent<br>• Known allergy or contraindication to any of the study drugs
Primary outcome(s)	Cumulative incidence of malaria infection detected from 2 weeks after the first day of the first dose of the first course to delivery inclusive
Key secondary outcomes	
Efficacy outcomes	• Composite of foetal loss and neonatal mortality.<br>• SGA-LBW-PT composite.<br>• The individual components of the above composites<br>• Neonatal length and stunting.<br>• Incidence of clinical malaria.<br>• Incidence of malaria infection detected by microscopy and PCR (not for point of care).<br>• Placental malaria by microscopy, PCR and histology (past and active infections).
Safety outcomes	• QTc-prolongation.<br>• Congenital malformations.<br>• Other SAEs and AEs.
Tolerance	• History of vomiting study drug (<60 min).<br>• Dizziness.<br>• Gastrointestinal complaints.
Antimicrobial activity and resistance	• Frequency of molecular markers of drug resistance in *Plasmodium falciparum* infections during pregnancy and delivery.

2.3.2 Lay summary

Context of the research: In malaria-endemic Africa, HIV and malaria conspire to increase the risks of pregnancy loss, preterm delivery, and growth retardation resulting in small babies. HIV-negative women in these areas receive intermittent preventive therapy in pregnancy (IPTp) with the antimalarial sulphadoxine-pyrimethamine (SP) to mitigate these effects. HIV-infected women receive daily prevention with cotrimoxazole (CTX), an antibiotic which also has antimalarial activity. However, the malaria parasite is increasingly resistant to SP and CTX. Recent trials with IPTp with mefloquine, when provided in addition to daily CTX, suggested that chemoprevention with an effective antimalarial markedly improves the protection against malaria in HIV-infected women compared to daily CTX alone. However, mefloquine was not well tolerated and other antimalarials are needed.

The long-acting antimalarial dihydroartemisinin-piperaquine (DP) is well tolerated and has shown great promise in trials in HIV-negative women in Uganda and Kenya. Chemoprevention with monthly DP has also been explored in a small study (N=200) of HIV-infected women on daily CTX in Uganda. Unfortunately, the study was inconclusive because malaria transmission was low. It also showed a drug-drug interaction with the anti-retroviral efavirenz (EFV), which markedly reduced the protective drug levels of DP. Since this first trial, the preferred first-line combination antiretroviral therapy (cART) recommended by WHO for use in the 2nd and 3rd trimester changed to dolutegravir (DTG) based cARTs. No such drug-drug interaction is expected between DTG and DP. We will, therefore, assess the safety and efficacy of malaria chemoprevention with monthly DP in HIV-infected women on daily CTX and DTG-based cARTs.

Aim: To determine whether monthly IPTp-DP in HIV-infected pregnant women on cARTs and daily CTX improves current policies to control malaria in areas with high resistance to CTX and high prevalence of malaria in East & Southern Africa.

Methods: This is a 2-arm multi-centre trial involving 898 (449 per arm) women in 8 hospitals in Kenya and Malawi comparing daily CTX alone versus daily CTX plus monthly DP. The primary outcome is the incidence of malaria infection. This study is conducted alongside a sister trial among HIV-negative women (IMPROVE trial) also evaluating monthly DP.

Potential benefits and risks to participants: CTX and DP are currently thought to be safe for the mother and foetus during the second and third trimesters of pregnancy. The drugs have not yet been given concurrently in HIV-infected women on DTG-based cARTs, and the study will, therefore, include a nested cardiac monitoring component and a pharmacokinetic component to ensure women have adequate and safe drug levels. Examinations will be non-invasive, except for blood sampling which may cause minor discomfort. Participants experiencing illness between visits will be seen and treated free of charge as part of the study.

Potential applications: The study is designed to inform WHO policy in countries experiencing high levels of malaria parasite resistance and may potentially pave the way for new strategies to combat malaria in HIV-infected pregnant women hopefully resulting in healthier pregnancies and newborns.

2.3.3 Short technical protocol summary

Background: Pregnant women represent a vulnerable population for malaria. HIV-infected women are particularly at risk. In HIV-infected pregnant women, WHO recommends daily cotrimoxazole (CTX), an antifolate drug, for malaria chemoprevention and prophylaxis against opportunistic infection. However, there is cross-resistance with sulphadoxine-pyrimethamine (SP), and high levels of antifolate resistance threatens the antimalarial effect of CTX. Recent trials with intermittent preventive therapy (IPT) with mefloquine in HIV-infected women on daily CTX, suggested that chemoprevention with an effective antimalarial markedly improves the protection against malaria compared to daily CTX alone. However, mefloquine was not well tolerated.

The long-acting combination of dihydroartemisinin–piperaquine (DP) is well tolerated and has shown great promise as IPTp in HIV-negative women in East-Africa. Chemoprevention with monthly DP has also been explored in HIV-infected pregnant women on daily CTX in Uganda. Unfortunately, the study was inconclusive because malaria transmission was too low and a clinically relevant drug interaction with efavirenz (EFV) was found reducing the exposure to DP. WHO now recommends dolutegravir (DTG) based combination antiretroviral therapy (ARTs) as the preferred firstline regimen including for pregnant women in the 2nd and 3rd trimester of pregnancy for the prevention of mother-to-child transmission of HIV. As a result, many countries in Africa are now transitioning to DTG-based combination antiretroviral therapy (cARTs). No such drug-drug interaction is expected between DTG and DP. We will, therefore, assess the safety and efficacy of malaria chemoprevention with monthly DP in HIV-infected women on daily CTX and DTG-based cARTs.

Objectives and methods: This is a 2-arm, individually-randomized, multi-centre, placebo-controlled superiority trial comparing the safety and efficacy of daily CTX plus monthly DP ('CTX-DP') versus daily CTX plus monthly placebo-DP (i.e. 'CTX-alone', control arm) to reduce malaria and the adverse effects of malaria in 898 (449 per arm) HIV-infected pregnant women on DTG-based cARTs. The study will be conducted in 8 hospitals in Kenya and Malawi in high SP-resistance areas with a high prevalence of malaria. These are the same sites where the sister trial in HIV-uninfected women is being conducted in Kenya and Malawi (IMPROVE trial). Both the mother and baby will be followed for 6-8 weeks after delivery. The study is powered at 80% (alpha=0.05) to detect ≥50% relative risk reduction (RR=0.50) in the primary outcome (cumulative incidence of malaria infection) from 12% in the CTX-alone arm (control arm) to 6% in in the interventions arm allowing for 20% non-contributors. The trial includes a pharmacokinetic assessment, cardiac monitoring for safety, assessment of antimalarial drug and the impact on immune responses to malaria and other pathogens.

2.3.4 Long technical protocol summary

Title: Chemoprevention with monthly IPTp with dihydroartemisinin-piperaquine for malaria in HIV-infected pregnant participants on daily cotrimoxazole in Kenya and Malawi: a multi-centre placebo-controlled trial(IMPROVE-2).

Background and rationale: In malaria-endemic Africa, HIV and malaria conspire to increase the risks of adverse pregnancy outcomes. For HIV-infected pregnant women, WHO recommends daily cotrimoxazole (CTX) for chemoprevention for malaria and prophylaxis against opportunistic infection. However, there is cross-resistance with SP, and high levels of antifolate resistance threaten the antimalarial effect of CTX. Recent trials in HIV-infected pregnant women who received daily CTX plus IPTp with mefloquine, suggested that chemoprevention with an effective antimalarial markedly reduces the risk of malaria compared to daily CTX alone. However, mefloquine was not well tolerated. The long-acting combination of dihydroartemisinin–piperaquine (DP) is well tolerated and has shown great promise as IPTp in HIV-negative women in East-Africa. Chemoprevention with monthly DP has also been explored in HIV-infected women on daily CTX in Uganda. Unfortunately, the study was inconclusive as malaria transmission was too low. Furthermore, a clinically relevant drug-drug interaction between DP and efavirenz (EFV) was found to reduce DP drug levels. Following the recommendation from WHO, many countries in Africa are transitioning from EFV-based to dolutegravir (DTG) based combination antiretroviral therapy (cARTs). WHO now recommends DTG-based cARTS as the preferred first-line cART regimen in the 2nd and 3rd trimester of pregnancy. No such drug-drug interaction is expected between DTG and DP. We will therefore assess the safety and efficacy of malaria chemoprevention with monthly DP in HIV-infected women on daily CTX and DTG-based cARTs. We will therefore assess the safety and efficacy of malaria chemoprevention with monthly IPTp with DP in women on daily CTX and DTG-based cARTs and daily CTX.

Overall aim: To provide WHO with evidence on whether monthly IPTp-DP can improve current policies to control malaria in HIV-infected pregnant women on daily CTX in areas with high levels of parasite resistance and malaria in East and Southern Africa.

Primary objective: To determine if monthly IPTp-DP in HIV-infected pregnant women receiving daily CTX is safe and superior to daily CTX-alone for controlling malaria infection in areas with high antifolates resistance to SP and CTX in Malawi and Kenya.

Hypotheses: Monthly IPTp-DP in women receiving daily CTX is superior to daily CTX-alone in controlling malaria infection during pregnancy in HIV infected women on antiretroviral therapy.

Overview Study Design: This multi-centre trial will be conducted in antenatal clinics in 8 hospitals in Kenya and Malawi located in areas with high prevalence of HIV and malaria and with high anti-folate resistance of the malaria parasite. These are the same sites where the sister trial in HIV-uninfected women (all gravidae) is being conducted in Kenya and Malawi (IMPROVE trial: NCT03208179). Overall, 898 (449 per arm) HIV-infected pregnant women who are 16 to 28 weeks pregnant assessed by ultrasound dating, will be randomised to receive one of the two interventions. Permuted block randomisation stratified by site (i.e. hospital) and HIV-status (known-positive and newly-diagnosed) will be used. Allocation concealment will be ensured by using sequentially numbered, sealed, opaque envelopes. The study will include pharmacokinetic studies and cardiac monitoring in a sub group of women. Other components include molecular marker studies of antimalarial resistance. We will also look at the impact on biomarkers of placental function and trans-placental antibody transfer and multi-pathogen neonatal cell mediated immune responses.

Study Interventions: Daily CTX for all trial participants receiving cARTs in addition to: a) monthly-DP ('CTX-DP'), or b) monthly DP-placebo ('CTX-alone') (control).

Follow-up procedures: Monthly visits during pregnancy, and then at delivery. Mother and newborn follow-up at 7 days and 6-8 weeks post-partum.

Primary outcome: The cumulative incidence of malaria infection detected from 2 weeks after the first day of the first dose of the first course to delivery inclusive, defined as the presence of peripheral (maternal) or placental (maternal) Plasmodium infection detected by either molecular diagnostics (henceforth referred to as PCR), microscopy, RDT or placental histology (active infection).

Sample size: 898 (449 per arm), allowing for 20% loss to follow-up.

Data Analysis: Log-binomial regression will be used to analyse the primary outcome, controlled for site and gravidity.

Primary partner institutions: KEMRI, Kenya; College of Medicine, University of Malawi, Malawi; Liverpool School of Tropical Medicine (LSTM); London School of Hygiene and Tropical Medicine (LSHTM); US Centers for Disease Control and Prevention (CDC); University of Bergen, Norway.

Funding: Joint Global Health Trials Scheme, Medical Research Council/Wellcome Trust/DFID, UK; and the European and Developing Countries Clinical Trials Partnership (EDCTP).

Sponsor: Liverpool School of Tropical Medicine (LSTM); Pembroke Place, Liverpool L3 5QA, UK, Phone: +44 0151 7053794; Email: lstmgov@lstmed.ac.uk.

Table 1: Summary Table of Study Design and Schedule of Assessment

**Phase**	Recruitment Phase	Enrolment	Antenatal follow-up			Delivery	Postnatal follow-up mother and newborn		
**Visit type number**<br>**Visit type description**	#1<br>Screening [b]	#1<br>Enrolment & baseline	#2[a]<br>Scheduled monthly ANC visit	#3<br>Unscheduled antenatal visits	#4[a]<br>Home visit or phone call	#5<br>Maternal and birth outcome assessment	#6<br>1 week Postnatal	#7<br>6-8 weeks Postnatal	#8<br>Unscheduled postnatal visits
**Recruitment**									
Pre-screening eligibility	X								
Prior consent discussion	X								
**Enrolment**									
Eligibility screen		X							
Informed consent		X							
Study code issued		X							
Randomisation		X							
Allocation		X							
**Interventions** [d]									
CTX-DP		DP<br>Day: 0,1,2	DP:<br>Day 0,1,2						
CTX-alone (Placebo-DP)		DPplac:<br>Day 0,1,2	DPplac:<br>Day 0,1,2						
**Assessments**<br>**Clinical measures**									
Copy Clinic/Lab data from ANC card and ANC and other clinic registers		X	X	X g		X	X	X	X g
Study CRF		X	X	X g	X	X	X	X	X g
Physical Exam.		X	X	X g		X	X	X	X g
Maternal anthropometrics[s]		X	X			X	X	X	
Ultra Sound Scan[e]		X							
Drug vomiting questionnaire[f] & AE		X [f]	X [f]			X	X	X	X
Newborn anthropometrics[m]						X	X	X	
Cardiac monitoring ECG[c]		X	X						
Drug adherence and tolerance					X				
**Samples**									
Blood sample [g-l]		VP [h l]	VP [i,l]	X[g]		VP [h] /CB [j]	HP [k]	HP [k]	HP[g]
Placental samples [m]						X [m]			

Visit type 1: Pre-screening interview, Screening, Consent, Enrolment, Randomisation, and Allocation. First IPTp treatment dose given under direct observation.
Visit type 2: Monthly scheduled ANC follow up visits for assessment.
Visit type 3 and 8: Unscheduled follow-up visits in between scheduled follow-up visits due to acute illness or other health concerns
Visit type 4: Telephone contact to assess drug adherence and tolerance will be conducted after each dose. Women without access to a mobile phone will be visited at home to monitor adherence. In addition, all participants will be visited at home at least once for random spot checks of adherence and to determine the availability, use of LLITNs the previous night, and integrity of LLITNs on a random basis.
Visit type 5: Delivery visit. To be conducted as soon as possible after birth without interrupting the initiation of breastfeeding or any necessary care required to the mother or newborn. A surface examination shall be conducted to assess for external congenital abnormalities.
Visits type 6 and 7: Routine postnatal care, physical examination and follow-up of the mother-newborn pair.

a. Scheduled visits spaced approximately one month apart. The range of monthly visits will be 3 to 7 visits depending on the gestational age of the woman at enrolment and delivery. Since recruitment is restricted to 16-28 weeks gestation, a median of 4 to 5 monthly visits is expected.
b. Pre-study screening can take place any time women start visiting the ANC; i.e. even prior to 16 weeks gestation, but the intervention can only start at 16 weeks.
c. Cardiac safety monitoring: In a subgroup of 160 women (80808080 in each arm), a total of six ECGs will be taken per woman as three sets of two ECGs (one before and one after the course); One ECG prior to 1st dose and one 4-6 hours after the 3rd dose of the 3-day DP course at enrolment and then twice more spaced at least one month apart over the course of the pregnancy, just before and 4h after a monthly DP dose.
d. Drug administration: The first dose (day-0) of each monthly course to be given under direct observation and subsequent 2 doses are to be taken at home (day-1 and day-2). Women will be called or receive text messages to remind them of or verify drug intake.
e. Baseline ultra sound scans in all women to assess the gestational age.
f. Vomiting and other adverse events (AEs) occurring within 30 minutes of the first dose will be assessed. Subsequent AEs will be captured in all women through passive surveillance and 1/3 of women through home visits for random spot checks (~1/3 of women). Participants will be encouraged to report to the study clinic for any subsequent AEs that may arise after each study visit at any time before the next study visit.
g. Unscheduled visit in between scheduled visits as part of continuous passive surveillance for acute illness or any other health problems. If a visit is for acute illness, a venepuncture (Mother, upto 6mL) or heelprick (HP, newborn) blood sample (~250-500 µL) will be taken for malaria RDT/smear, Hb, and DBS for malaria, and plasma for detection PCR and antibody assays for viral pathogens. A maternal 5 mL urine sample will also be taken for detection of urinary tract infections and arboviruses.
h. Blood sample on enrolment & delivery by venepuncture (VP) (up to 12 mL): All women all sites: haemoglobin level, HIV test (unless known to be HIV+), maternal HIV viral load, syphilis, baseline tests before starting cARTs (biochemistry and full-blood count), malaria smear, dried blood spots (DBS) for molecular malaria diagnostics (not for point of care, DBS for multi-pathogen antibody assays and drug resistance), detection PCR and antibody assays for viral pathogens. Sub-group: Drug levels; antibody and cell mediated immune responses to malaria and other pathogens; biomarkers of placental function, inflammation and gestational age; malaria diagnostics, and drug levels. DBS, plasma, serum and red cell pellets will be stored.
i. Blood sample scheduled monthly visits by venepuncture (up to 12 mL): Same assays as for baseline, except for syphilis. Maternal HIV viral load testing will be done once in the third trimester (and/or delivery).
j. Blood sample umbilical cord blood (CB) (~up to 300 mL): Haemoglobin level, malaria molecular diagnostics, antibody and cell mediated immune responses to malaria and other pathogens), detection PCR and antibody assays for viral pathogens, and drug levels. DBS, blood smear, plasma and red cell pellets will be stored.
k. Blood sample newborn by heel prick (HP) (~250-500 µL) in all babies at week 1 postnatal visit (mRDT, malaria smear, DBS for molecular malaria diagnostics), week 6 postnatal visit (infant HIV PCR DNA viral detection at week 6-8), mRDT, malaria smear, DBS for molecular malaria diagnostics). At sick visits infants with fever or

other symptoms heel prick samples collected for malaria RDT, microscopy and dried blood spot, and in a subgroup of asymptomatic infants for immunological assays.

l. Blood sample ECG component by venepuncture 2 ml blood sample taken 4-6 hours after last dose of the initial course and then monthly just before the first dose and 4h after the third dose of a monthly course of DP (cMax) for biochemistry, blood sugars and drug levels (subgroup of 240 women in ECG component)
m. Placental samples at delivery/termination of pregnancy: placental section for histopathology, amnion roll, placental impression smear, maternal placental blood for malaria diagnostics (not for point of care), drug levels, and cord blood (i.e. newborn's side) (see cord blood sample).
n. Maternal anthropometric measurements: Enrolment: height; All visits: bodyweight, and MUAC. Infant anthropometric measurements at birth, 1, and 6-8 weeks post-partum: bodyweight and length, head, abdominal, mid-upper arm circumference.

3 INTRODUCTION

3.1 THE BURDEN MALARIA AND HIV AND VIRAL INFECTIONS IN PREGNANCY

3.1.1 Malaria and HIV in pregnancy

The double burden of HIV infection and malaria is borne heaviest in sub-Saharan Africa, where they conspire to threaten maternal and newborn health. Pregnancy increases the susceptibility of women to malaria with devastating consequences for mother and foetus.[1] The burden of human immunodeficiency virus (HIV) on maternal health has been superimposed on that of malaria, particularly in malaria-endemic regions in Africa where women account for approximately 57% of all people living with HIV[2,3] and where approximately 12% of women are co-infected with HIV and malaria during pregnancy.[4,5] HIV-infected women are at particular risk and consistently experience more peripheral and placental malaria, higher parasite densities, and more febrile illnesses, severe anaemia, and adverse birth outcomes than their HIV-uninfected counterparts, particularly in multigravidae[5,6] and, in some studies, increased mother-to-child transmission of HIV (MTCT).[6,7] Thus, HIV alters the typical gravidity-specific pattern of malaria immunity and risk by shifting the burden from, primarily, primigravidae and secundigravidae to all pregnant women.[6] Similar to malaria, maternal HIV infection increases the risk of miscarriage, stillbirth, and other adverse birth outcomes[2,7,8] and the combination of pregnancy, HIV and malaria poses a triple threat to maternal health and foetal development.[9]

The World Health Organization (WHO) currently recommends a three-pronged approach for the control of malaria in pregnancy; case-management of symptomatic malaria; providing long lasting insecticide-treated bed nets (LLITNs) to reduce exposure to infective mosquito bites; and intermittent preventive treatment of malaria in pregnancy (IPTp) for prevention of malaria in HIV-negative pregnant women and daily cotrimoxazole (CTX) in HIV-infected women.[4,10,11] IPTp consists of the administration of treatment doses of sulfadoxine-pyrimethamine (IPTp-SP) during the second and third trimesters of pregnancy at every scheduled antenatal visit at least a month apart.[4,10,11]

3.1.2 Viral co-infections in pregnancy

In East and Southern Africa, maternal viral infections including cytomegalovirus (CMV), Epstein-Barr-virus (EBV), measles virus, Respiratory syncytial virus (RSV) and several mosquito vector-borne viruses such as Dengue, Chikungunya, Zika, Yellow Fever, Rift Valley Fever[12]are co-endemic with malaria and HIV, and are each known to be associated with pregnancy loss and pre-term delivery[13-15] and may compound the adverse pregnancy outcomes associated with malaria and HIV. The impact of maternal viral co-infections or the potential effect of antimalarials on some of these viral infections has not yet been documented.

3.2 MALARIA PREVENTION IN HIV-INFECTED WOMEN

3.2.1 Antifolate resistance

Sulphadoxine-pyrimethamine (SP), an anti-folate drug, is the only antimalarial currently recommended by the WHO for IPTp. However, IPTp-SP is contraindicated in HIV-infected pregnant women receiving daily CTX, both of which are sulpha-based anti-folate drugs, due to their overlapping mechanisms of action and synergistic risk of adverse drug reactions when used in combination.[11] CTX has some anti-malarial properties, and in HIV-infected pregnant women, plays a dual role as both prophylaxis against opportunistic infections and malaria and, has been associated with reduced incidence of malaria infection and maternal anaemia[16] and improved pregnancy outcomes.[17]

However, because SP and CTX as anti-folate drugs are thought to share the same drug targets, the increasing resistance of the malaria parasite to SP, also threatens the efficacy daily CTX for malaria prophylaxis in HIV-infected women.[18-21] Recent IPTp trials in HIV-infected women who received the combination of daily CTX plus an additional 3 courses of IPTp with mefloquine, suggested that chemoprevention with an effective antimalarial markedly improves the protection against malaria compared to daily CTX alone. However, IPTp-MQ was poorly tolerated and was associated with increased viral load and MTCT of HIV.[22,23] There remains a need for enhanced chemoprevention of malaria in HIV-infected pregnant women.[2,9,24,25]

3.2.2 Chemoprevention with Dihydroartemisinin-Piperaquine (DP)

3.2.2.1 IPTp with DP in HIV-negative women

Unlike IPTp with mefloquine, amodiaquine or chloroquine based combinations,[26-28] the long-acting combination of dihydroartemisinin–piperaquine (DP) is well tolerated and has shown great promise as IPTp in HIV-negative women in two recently completed trials conducted in Uganda[29]and Kenya.[30,31] A recent meta-analysis of these two trials suggests a 73% (95% CI 43 to 88) and 75% (37 to 90) reduction in clinical malaria and malaria infection during pregnancy respectively, a 65% (95% CI 30 to 83) reduction in placental malaria, and a non-significant reduction in adverse pregnancy outcomes of 17% (-10 to 38).[32]

3.2.2.2 Experience to date with IPTp with DP in HIV-positive women

IPTp with DP has only been evaluated in a single trial (n~200) in Uganda comparing daily CTX alone to daily CTX plus monthly DP. This trial showed no statistical difference in the risk of malaria during pregnancy or at delivery.[33] The study was inconclusive because of the small sample size and the very low malaria transmission in the study area following the successful introduction of Indoor Residual Spraying (IRS)[34]; the rate of placental malaria in the CTX arm was approximately 10-fold lower than the risk observed in HIV-infected daily CTX recipients in the previous years in this same study area.[33] Furthermore, a clinically relevant drug-drug interaction between DP and efavirenz (EFV) was found to reduce DP drug levels by 38%. Based on this study alone, it would be wrong to conclude that HIV-infected women require no additional antenatal malaria prevention beyond daily CTX because the power of this study was undermined by the low prevalence of the primary outcome, which was unexpected and beyond the control of the investigators.[24]

Encouragingly, the addition to CTX of monthly DP was well tolerated, which is a necessary precondition for any candidate prevention measure. Furthermore, there were no indications that the piperaquine-associated corrected QT prolongations were worse in HIV-infected women receiving CTX and EFV-based combination antiretroviral therapy (cART) than in HIV-uninfected women receiving DP and were also not associated with pregnancy status or, importantly, with the number of previous IPTp courses taken.[35]

3.2.3 DP and other viral infections

A recent study showed chloroquine, a quinoline-based anti-malarial demonstrated in-vitro prophylactic and therapeutic effects on Chikungunya virus[36] as well as conferred protection against Zika virus infection in pregnant mice.[37] Piperaquine, has a similar chemical structure as chloroquine, but its impact on arboviruses has not yet been explored. Additionally, artemisinins have been shown to have robust inhibitory effects against some viruses such as CMV and EBV.[38,39] If DP is shown to inhibit the growth of arboviruses and other maternal viral co-infections, they may lead the way for integrated approaches towards the prevention of malaria and arboviral and other viral infections in HIV-uninfected and infected pregnant women with monthly DP and use of insecticide-treated nets.

3.2.4 Recent changes in first-line combination antiretroviral therapy (cART) from efavirenz (EFV) to dolutegravir (DTG) based cARTs

Following the recent recommendations by WHO in 2018, a transition is occurring in 2018-2019 from EFV-based cARTs to dolutegravir (DTG) based cARTs in sub-Saharan Africa, which in many countries may be completed by 2020.[40-43] DTG is associated with fewer drug-drug interactions, is more effective than EFV-based regimens in suppressing viral load in late pregnancy[44,45] and has a higher genetic barrier to resistance than EFV. In its most recent guidelines from December 2018,[43] WHO is now recommending the use of DTG-based combinations (TDF+3TC (or FTC) + DTG) as the preferred first-line regimen in the 2nd or 3rd trimester and breastfeeding women (see highlighted text in Table 2: WHO updated preferred first-line regimens in pregnant and breastfeeding women and women of childbearing potential, page 22).[43] Because the safety of dolutegravir at the time of conception and in early first trimester pregnancy remains to be established,[46] WHO continues to recommend EFV-based cARTs (TDF+3TC (or FTC)+EFV[47]) in the first trimester and for women of children bearing age who wish to become pregnant or do not have access to 'consistent and reliable contraception', unless a woman chooses to use DTG-based cARTs 'after informed choice' (see 3.4.3, Dolutegravir, page 24).

Many countries in sub-Saharan Africa are recommending the continued use of EFV in pregnant women because of concerns about the complexity of WHO's regimen. However, several countries are in the process of reviewing their cART and PMTCT guidelines in the light of these updated recommendations from WHO[43] and recent findings from the DOLPHIN-II trial showing that DTG-based combinations are much more effective than EFV-based combination in rapidly suppressing viral load below the level of detection when initiated late in pregnancy.[44,45] It is therefore anticipated that more countries in sub-Saharan Africa will consider WHO guidelines and use DTG-based regimens as the preferred first-line cART during the 2nd and 3rd trimester of pregnancy. The Malawian[48] PMTCT guidelines were reviewed in March 2019 and will now include the use of DTG-based cART regimens in the 2nd and 3rd trimester of pregnancy (Clifford Banda, personal communications). Kenya is scheduled to review their guidelines in late 2019, and currently recommends EFV-based combinations in women of child-bearing potential including during pregnancy.[49] However, the Kenyan PMTCT guidelines also state that: a) 'DTG is safe during pregnancy and breastfeeding if initiated 8 weeks after conception (although women need to be counseled on the risk of becoming pregnant while breastfeeding and provided with effective contraception)' and b) 'Women and adolescent girls of childbearing potential may opt to remain on DTG and should be supported in their decision and offered effective contraception as well as counseling on the potential risk of birth defects when DTG is used around the time of conception.'[49]

Table 2: WHO updated preferred first-line regimens in pregnant and breastfeeding women and women of childbearing potential

<table>
<tr><th colspan="4">Populations</th><th>Preferred first line regimen</th><th>Alternative first line regimen(s)</th><th>Special situations</th></tr>
<tr><td colspan="4">Adult men and adolescent boys</td><td rowspan="5">TDF + 3TC (or FTC) + DTG</td><td rowspan="5">TDF + 3TC (or FTC) + EFV 600mg
TDF + 3TC (or FTC) + EFV 400mg</td><td rowspan="5">AZT + 3TC + EFV 600mg
TDF + 3TC (or FTC) + PI/r[b]</td></tr>
<tr><td rowspan="5">Adult women and adolescent girls</td><td colspan="3">Pregnant or breastfeeding[a]</td></tr>
<tr><td colspan="3">Not of childbearing potential</td></tr>
<tr><td rowspan="3">of child-bearing potential</td><td colspan="2">Offered and using effective contraception</td></tr>
<tr><td rowspan="2">Offered but not using effective contraception or
without access to contraception or
want to become pregnant[g]</td><td>Choose to use DTG after informed choice</td></tr>
<tr><td>Choose to use EFV after informed choice</td><td>TDF + 3TC (or FTC) + EFV 600mg</td><td>TDF + 3TC(or FTC) + EFV 400mg
TDF + 3TC (or FTC) + ATV/r[b]</td><td>AZT + 3TC + EFV 600mg
TDF + 3TC (or FTC) + RAL</td></tr>
<tr><td colspan="4">Children</td><td>ABC + 3TC + DTG[c]</td><td>ABC + 3TC + LPV
ABC + 3TC + RAL[d]</td><td>ABC + 3TC + EFV[e] (or NVP)
AZT + 3TC + EFV[e] (or NVP)
AZT + 3TC + LPV/r (or RAL)</td></tr>
<tr><td colspan="4">Neonates</td><td>AZT + 3TC + RAL</td><td>AZT + 3TC + NVP</td><td>AZT + 3TC + LPV/r[f]</td></tr>
</table>

[a] Based on programmatic practicality and uncertainty surrounding possible DTG effects after the neural tube closes at 28 days of gestation as noted by the originator and FDA, previous safe period after 8 weeks is now extended to after the first trimester. In practice, the majority of women will not yet know that they are pregnant during the first 8-12 weeks of pregnancy.

[b] If the national prevalence of pre-treatment resistance to EFV or NVP is 10% or higher or if no other alternatives are available.

[c] For age and weight groups with approved DTG dosing.§

[d] RAL can be used as an alternative regimen if LPV/r solid formulations are not available.

[e] EFV should not be used for children younger than three years of age.

[f] If starting after 2 weeks of age.

[g] Women of childbearing potential who intend to become pregnant or who are not otherwise using or accessing effective contraception can receive DTG based regimens if they have been informed of the potential increase in the risk of neural tube defects (at conception and up to the end of first trimester) (See tables on page 24-25). However, many vulnerable and at-risk adolescent girls and women may not be able to negotiate when they want to become pregnant and/or might not be aware they are pregnant.

Source: World Health Organisation, Updated recommendations on first-line and second-line antiretroviral regimens and post-exposure prophylaxis and recommendations on early infant diagnosis of HIV: Interim Guidelines. Supplement to the 2016 consolidated guidelines on the use of antiretroviral drugs for treating and preventing HIV infection. December 2018. HIV treatment. Geneva: World Health Organisation. Table 1, Preferred and alternative first-line regimens in adults and adolescents, page 21 of WHO's guidelines.[43] The authors of this protocol added the highlights.

3.3 DRUG-DRUG INTERACTIONS

3.3.1 Drug-drug interaction between DP and dolutegravir

There are no previous studies of DP and DTG-based cART regimens. DTG is associated with fewer drug-drug interactions than efavirenz[40,50] because it is metabolized primarily by UDP glucuronosyltransferase (UGT)1A1 and is not an inducer nor inhibitor of CYP isoenzymes[50] minimizing the risk of clinically relevant drug-drug interactions that would decrease the concentrations of piperaquine which is mainly metabolised by CYP3A4 (and to a lesser extent by CYP2C9 and CYP2C19). Previous drug-drug interaction studies between dolutegravir and artemether-lumefantrine or artesunate-amodiaquine showed that dolutegravir did not significantly change the maximum concentration in plasma, the time to maximum concentration, and the area under the concentration-time curve (AUC) for artemether, dihydroartemisinin, lumefantrine, and desbutyl-lumefantrine, nor did it significantly alter the AUC for artesunate, dihydroartemisinin, amodiaquine, and desethylamodiaquine.[51]

It is less clear if there is potential for DP to affect dolutegravir concentrations. Dihydroartemisinin does not induce or inhibit UGT1A1, and piperaquine is a weak inducer. Nevertheless, coadministration of dolutegravir with artesunate-amodiaquine resulted in 42 and 24% approximate decreases in the DTG trough concentrations and the AUC, respectively.[51] The mechanisms for these interactions are unclear. These decreases in DTG concentrations are unlikely to be of clinical significance since for all subjects who received DTG with artesunate-amodiaquine trough concentrations were comparable to or above 1,100 ng/ml (10) trough concentrations observed in prior dolutegravir phase 3 adult trials.[51] Furthermore, through concentrations of 300 ng/ml[52] or 150ng/mL (the 5th percentile of the DTG trough concentration distribution) and are associated with good efficacy of DTG.[52] The combinations were well tolerated with no serious adverse events.

Nevertheless, since DP, like artesunate-amodiaquine, is a 4-aminoquinoline ACT and no previous pharmacokinetic studies of DP and DTG-based cART regimens have been conducted, we propose to investigate this through a pharmacokinetic sub-study as part of the current IMPROVE-2 study.

3.4 SAFETY OF STUDY DRUGS IN PREGNANCY

3.4.1 CTX

CTX has been used for many years as prophylaxis for opportunistic infections in pregnancy as part of routine HIV care.

3.4.2 Dihydroartemisinin-Piperaquine

3.4.2.1 Experience with DP in pregnancy

There is also increasing experience with DP in pregnancy, both for treatment (case management) and chemoprevention (IPTp). Treatment with DP has been shown to be safe in the 2nd and 3rd trimester of pregnancy.[29,30,53-59] A single course DP is safe for use in the 2nd and 3rd trimesters, is recommended by WHO for treatment of malaria in the 2nd and 3rd trimester and has been first-line treatment for this indication in Papua, Indonesia, for over 10 years.

In addition to the extensive experience using this drug for treatment in pregnancy, approximately 3,282 women have received either 3-course or monthly IPTp-DP in Kenya,[30] Uganda,[29,60] our recently completed IPTp-DP trial in Indonesia (STOPMIP-Indonesia,[61] ISRCTN34010937) and in our ongoing IMPROVE trial in HIV-negative women. The three recent trials in Uganda and Kenya showed that

monthly IPTp with DP was well tolerated with no noticeable differences to SP in side effect profiles. There is also no indication that IPTp-DP is associated with an increased risk of congenital malformation or pregnancy loss. The existing evidence in Africa suggests a 61% [95% CI 13-83] reduction in the risk of foetal loss compared to SP in HIV-negative women.[29,30,32] The single trial to date in HIV-infected women showed IPTp-DP to be well tolerated (N=184).[62]

3.4.2.2 Piperaquine-associated QTc prolongation

The main safety concern with DP is a dose-dependent QTc prolongation associated with piperaquine, like that observed with other antimalarials like chloroquine, but less severe than with halofantrine and quinine. Because of its slow elimination, monthly doses of DP could result in dose accumulation of piperaquine. However, the experience in over 2,200 HIV-uninfected pregnant women who received either 3-course or monthly IPTp-DP in Kenya, Uganda, Malawi and Indonesia[29,30,63-65] (Clinicaltrials.gov NCT02793622 and NCT03009526) does not suggest that the known piperaquine associated QTc prolongation increases with the number of previous courses received, i.e. the magnitude of QTc prolongation was similar after 3 to 5 courses to the prolongation observed after the 1st course and similar to that observed in patients treated for acute malaria. This is likely because piperaquine-associated QTc prolongation is transient and lasts for a few days only. This is also consistent with experience with ITP in Ugandan children provided with 18 monthly courses of DP from 6 to 24 months of age the magnitude of transient QTc prolongation remained constant and was similar after ≥12 monthly courses compared to a single course.[66]

There is less experience (n=184) with IPTp with DP when provided to HIV-infected women who receive daily CTX and cARTS, although in the single previous study with IPTp-DP in HIV-infected women on EFV-based cART, all pre- and post-dosing QTc intervals were within normal limits (<450 msec).[67]

3.4.3 Dolutegravir (DTG)

The DTG-based fixed combination cART tenofovir/lamivudine/dolutegravir (TLD) is recommended by WHO for use in the 2nd and 3rd trimester of pregnancy (see Table 2: WHO updated preferred first-line regimens in pregnant and breastfeeding women and women of childbearing potential, page 22). This is based on the lower costs and superior efficacy of TLD, relative to EFV-based combination, in rapidly suppressing viral load and the available safety data from approximately 2,000 pregnancies initiating DTG-based ARTs in the ongoing Tsepamo birth study in Botswana. This Tsepamo study showed that the rate of adverse birth outcomes and congenital malformations were similar among pregnant women who initiated DTG-based and EFV-based cART at least eight weeks after conception.[9, 90]

There are concerns however about the use of DTG-based cARTs in women of childbearing age because results from the Tsepamo birth study in Botswana also indicated that DTG was associated with an increased risk of neural tube defect when used at the time of conception. This resulted in the WHO updated treatment guidelines from December 2018. These new guidelines recommend that women of children bearing potential should only be offered DTG-based cARTs if they are using effective contraception, or choose to use DTG after informed choice. More recent collective data involving monitoring of 198 pregnancies from prospective antiretroviral pregnancy exposure registries (APR and EPPICC) as well as data from 3 other studies with 104 pregnancy outcomes identified no birth defect signal, either in defect prevalence of defect type. In addition, among the birth defects reported after DTG exposure during pregnancy or at the time of conception, none involved neural tube defects. These data provide some reassurance to women whose pregnancies have already been exposed to DTG or who have limited options. They also highlight the importance of continuing prospective monitoring of pregnancies exposed to DTG around the time of conception.

4 Justification for the Study

4.1 Why is this study needed now?

IPT with DP has great promise to reduce the burden of malaria during pregnancy in HIV-negative women in Africa, but because of the drug-drug interaction with EFV, the benefits are lost in HIV-infected women receiving EFV-based cARTs. A recent WHO Malaria Policy Advisory Committee (MPAC) recommendations based on WHO Evidence Review Group (ERG) on malaria in pregnancy from October 2017 [25,68] highlighted the need for alternative strategies for chemoprevention in HIV-infected women as CTX provides only partial protection against malaria; and the need for PK/PD studies of antimalarials in pregnancy to advice on dosage modification including in HIV-infected women on different cART regimens. The proposed IMPROVE-2 trial will address these gaps and will determine the efficacy of monthly IPTp-DP when added to daily CTX on antenatal malaria infection.

4.2 Other relevant research ongoing elsewhere

4.2.1 HIV-infected women

We are aware of one other EDCTP2-funded trial similar to IMPROVE2 to be conducted in Mozambique and Gabon between 2018 and 2023: A randomised double-blind placebo-controlled trial to evaluate the safety and efficacy of DHA-PPQ for IPTp in HIV-infected pregnant women receiving CTXp and ARV drugs and using LLITNs (MAMAH)[69]. A search of WHO's trial registry indicated no other registered IPTp-DP, or DP prophylaxis trials in HIV positive pregnant women are ongoing in Africa.

4.2.2 HIV-uninfected women

However, five studies with IPTp with DP in HIV-negative women are ongoing or have been completed involving a total of 4,519 HIV-negative participants who received or will receive IPTp with DP in Africa. This includes 3,120 IPTp-DP recipients in our own (sister) trial in involving a total of 4,680 women in Kenya, Malawi and Tanzania (IMPROVE trial). The IMPROVE trial (separate stand-alone protocol) and IMPROVE-2 trial (this protocol) are conducted in the same sites in Kenya and Malawi. We are also aware of two smaller trials comparing monthly IPTp-DP and IPTp-SP in a high SP resistance area in eastern Uganda, published recently,[60] and in Malawi (PMI-funded, Julie Gutman, CDC, personal communications, Clinicaltrials.gov NCT03009526, approximately 602 women, 301 per arm). These five trials involving over 4,500 IPTp-DP recipients will be sufficient to address the impact of IPTp-DP versus IPTp-SP on adverse birth outcomes in a prospective meta-analysis of individual participant data.

We are also aware of one other study in Africa looking at the cardiac safety of single course (rather than repeat course) of IPTp with DP in pregnancy (n=100) in north-eastern Tanzania (Matthew Chico et al, LSHTM; Clinicaltrials.gov NCT02909712).

Lastly, our STOPMIP-Indonesia trial,[65] comparing IPTp-DP with screening strategies involved a further 681 women on monthly IPTp-DP, but the efficacy results cannot be extrapolated to Africa because the study is conducted in low transmission areas where *P. falciparum* and *P. vivax* co-exist, and the control arm does not involve IPTp-SP.

4.3 Hypotheses

We propose to conduct a 2-arm trial in HIV-infected women on anti-retroviral drugs and daily CTX, comparing the current strategy with CTX alone, against a new intervention consisting of monthly IPTp with DP. The study has the following hypothesis: Monthly IPTp-DP in women receiving daily CTX is

superior to daily CTX-alone in controlling malaria infection during pregnancy in HIV infected women on antiretroviral therapy.

5 AIM & OBJECTIVES

The overall aim of the study is to provide WHO with evidence on whether monthly IPTp-DP can improve current policies to control malaria in HIV-infected pregnant women on daily CTX in areas with high levels of parasite resistance and malaria in East and Southern Africa.

5.1 PRIMARY OBJECTIVE

To determine if monthly IPTp-DP in HIV-infected pregnant women receiving daily CTX is safe and superior to daily CTX-alone for controlling malaria infection in areas with high antifolates resistance to SP and CTX in Malawi and Kenya.

5.2 SECONDARY OBJECTIVES

1. To determine if monthly IPTp-DP in HIV-infected pregnant women receiving daily CTX is safe and superior to daily CTX-alone for preventing adverse pregnancy outcomes due to malaria.
2. To determine the effect of co-administration of DP and current first-line cARTs on the pharmacokinetic properties of piperaquine, first line cARTs and CTX
3. To determine if the level of SP drug resistance, assessed by molecular markers, affects the potential impact of CTX.
4. To determine the safety of monthly-DP in pregnant women receiving daily CTX and cARTs by conducting nested cardio monitoring studies.
5. To determine if monthly IPTp-DP in HIV-infected pregnant women receiving daily CTX affects the risk of mother-to-child transmission of HIV infection.
6. To determine if monthly IPTp-DP in HIV-infected pregnant women receiving daily CTX reduces the risk of viral infections.
7. To determine the impact of the interventions and prenatal exposure to HIV and/or malaria on adaptive and innate-like immune responses

6 TRIAL DESIGN & DESIGN CONSIDERATIONS

6.1 OVERVIEW OF DESIGN

This will be a multi-centre, 2-arm, parallel, placebo-controlled, individually randomised, phase-3, superiority trial in 898 (449 per arm) HIV-infected pregnant women receiving daily CTX and cARTs to compare the efficacy of monthly IPTp-DP (‘CTX-DP’) against monthly placebo-DP (‘CTX-alone’) (allocation ratio 1:1). The study will be conducted in about 8 sites in high malaria endemic and high HIV endemic areas in southern Malawi and western Kenya. These are the same sites in Malawi and Kenya where the trial in HIV-negative women (IMPROVE) will be conducted.

HIV-infected pregnant women (all gravidae) attending for antenatal care (ANC) between 16-28 weeks’ gestation irrespective of CTX or cART use, assessed by ultrasound dating, will be eligible. Participants will be seen monthly until delivery. Mothers and infants will be followed-up for 6 to 8 weeks post-partum. Sub-studies of cardiac-safety and pharmacokinetics will be conducted.

The trial is powered at 80% (alpha=0.05) to detect a 50% reduction in the cumulative incidence of malaria infection and will take 24 months to complete.

6.2 Design considerations

6.2.1 Rational for a fixed dose rather than a dose based on bodyweight

There are pragmatic benefits to using a fixed dose rather than a dose based on bodyweight, but the risk is that heavier women are under-dosed as they receive less drug per kg bodyweight. However, the data from the two Ugandan trials in pregnant women, both of which used a fixed monthly dose of 3x3 tablets per day, showed that in contrast to expectations, women with a lower BMI have lower (rather than higher) piperaquine concentrations compared to those with higher BMI, despite the fact that women with the lower BMI received more drug per kg bodyweight.[29,33,35,70,71] Women with a lower BMI appear to eliminate piperaquine more quickly, resulting in lower plasma drug concentrations relative to the dose in mg/kg received. This was observed in both HIV-infected women (all on EFV-based cARTs) and HIV-uninfected pregnant women. In HIV-uninfected women, women in the higher weight-based category achieved adequate drug concentrations with the fixed dose of 960 mg and the fixed-dose regimen was very effective and only 3 out of 373 women got malaria in the monthly IPTp-DP arm (Rada Savic and Erika Wallender, UCSF, personal communications). which reflected a >90% reduction in malaria compared to the monthly SP arm.[72] Models predict that HIV-infected women on DTG-based regimens (i.e. assuming no drug-drug interaction affecting piperaquine concentrations) will achieve the same adequate trough levels with a fixed dose of 3 tablets per day (960 mg/day) as observed in HIV-uninfected women (courtesy of Rada Savic and Erika Wallender, UCSF, personal communications). This has important potential policy implications as it suggests that women in the heavier bodyweight categories (60-80kg and >80kg) are likely to achieve adequate piperaquine concentrations with a fixed monthly dose of 3x3 tablets, avoiding the need for a more complex weight-based loading dose or weekly regimens.

6.2.2 Rationale for nested pharmacokinetic studies

Because no previous studies have looked at the combination DP and DTG-based cARTs, there is a need for further PK profiling of piperaquine-DTG drug-drug interactions to verify the pharmacological model predictions. This includes an assessment of the effect of the cARTs on piperaquine pharmacokinetics, and the effect of repeated piperaquine dosing on the steady state concentration of the cARTs and CTX.

6.2.3 Design as a superiority trial

Daily cotrimoxazole is cheap, well tolerated, safe, widely available and with the double benefit of conferring protection against malaria and opportunistic infections in HIV-infected participants. In contrast, DP will be more expensive, result in more adverse effects, and confer a greater pill burden during implementation as it will be given in addition to daily CTX and cART in this population. Therefore, the health benefits need to be sufficiently greater than observed with daily CTX-alone to outweigh the burden of an additional monthly 3-day DP regimen and the additional cost associated with the drug and implementing a new policy in HIV-infected pregnant participants.

7 METHODS: PARTICIPANTS, INTERVENTIONS, AND OUTCOMES

7.1 STUDY SETTING

For the study to have a high likelihood of showing a differential impact, it must be conducted in areas with moderate to intense malaria transmission and high SP resistance, which is a proxy of CTX resistance. The prevalence of placental infection detected by PCR and histopathology among paucigravidae in the SP arms of our recent trials in HIV-negative participants was 50% in Kenya[30] and 57% in Malawi.[73] These high rates in both countries suggest both a failure of SP to clear or prevent infections and high transmission intensity. In the sites in Kenya and Malawi, the prevalence of the quintuple mutant is >98% and approximately 6-9% of participants in 2013 were infected with parasites carrying the sextuple *dhfr/dhps* mutation (Pfdhps-A581G in combination with the quintuple mutant). The National Malaria Control Programmes (NMCP) in these two countries regard SP resistance as a major threat to the existing IPTp-SP policy.

The study will be conducted in antenatal clinics and delivery units of Government or private (e.g. mission) hospitals and clinics with at least 30 new antenatal attendants per month in areas with year-round malaria transmission. The final choice of sites will depend on the available infrastructure and absence of planned malaria control interventions in the catchment area that might have a major impact on the malaria transmission intensity during the study period (e.g. planned mass campaigns to reduce malaria transmission such as indoor residual spraying [IRS]).

7.1.1 Kenya

The study is part of a multi-year collaboration between LSTM and the Kenya Medical Research Institute (KEMRI), Kisumu, western Kenya, and the US Centers for Diseases Control and Prevention (CDC)-Kenya, and will be conducted in approximately 3 to 5 Hospitals and satellite ANC clinics in the counties of the previous Western and Nyanza Provinces; including, but not limited to Busia, Siaya, Kisumu, Homa Bay and Migori counties in high malaria transmission areas in western Kenya.

7.1.1.1 Suggested primary hospitals

Ahero sub-county hospital, Rabuor sub-county hospital and Akala sub-county hospital and the corresponding satellite clinics that refer patients to these hospitals.

7.1.1.2 *Backup and expansion hospitals and clinics*

Homa Bay County Referral Hospital, Rongo sub-county hospital, Migori County Referral Hospital, St Mary's Hospital, Mumias, Mukumu Mission Hospital, Kakamega, Kisumu District Hospital, Pap Onditi sub-county hospital, Sondu sub-county hospital, Busia District Hospital, Nyamira District Hospital, Bondo County Hospital, Siaya County Referral Hospital, Madiany County Hospital, Kisumu County Referral Hospital, Jaramogi Oginga Odinga Teaching and Referral Hospital, Rachounyo sub county hospital, Kendu Bay sub-county hospital and Kendu Adventist hospital. This list is not exclusive and another hospital may be considered for inclusion in western Kenya if they fulfil the entry criteria for size and malaria transmission and control.

7.1.2 Malawi

The sites in southern Malawi will include 3 to 5 district hospitals and satellite ANC clinics where several pregnancy studies have been conducted. The Research Support Centre from the College of Medicine (CoM), University of Malawi, and supported by Malawi-Liverpool-Wellcome Trust Clinical Research-Programme linked with the CoM in Blantyre will be the coordination institutions providing administrative and laboratory support.

7.1.2.1 Suggested primary hospitals

Mangochi District Hospital, Zomba Central Hospital, Chikwawa District Hospital, Madziabango and Mpemba Health Centers and, the corresponding satellite clinics that refer patients to these facilities.

7.1.2.2 *Backup and expansion hospitals and clinics*

These included, but are not limited to Machinga District Hospital, Lungwena Health Center, Makanjira Health Center, Monkeybay Community Hospital, Domasi Rural Hospital, Mfera Health Center, Chapananga Health Center, Balaka District Hospital, Chiradzulu District Hospital, Mwanza District Hospital and Ntcheu District Hospital. This list is not exclusive as other hospitals may be considered for inclusion in southern Malawi if they fulfil the entry criteria for size and malaria transmission and control.

7.2 ELIGIBILITY CRITERIA

7.2.1 Inclusion criteria

Includes: HIV-infected pregnant participants between 16-28 (both inclusive) weeks gestation, singleton pregnancy, on or eligible for DTG-based cARTs and CTX, resident of the study area, willing to adhere to scheduled and unscheduled study visit procedures, willing to deliver in a study clinic or hospital.

7.2.2 Exclusion criteria

Includes: Multiple pregnancies (i.e. twin/triplets), HIV-negative or HIV-status unknown, known heart ailment, severe malformations or nonviable pregnancy observed by ultrasound, unable to give consent, confirmed or suspected TB infection, known allergy or contraindication to any of the study drugs.

7.3 INTERVENTIONS

7.3.1 Trial Medications and interventions

7.3.1.1 Study arms

At enrolment, all participants will be randomly allocated to receive one of two regimens:

Table 3: Dosing regimen per course

Study arm	Drug regimen	Number of tablets once daily per course (In addition to daily CTX)		
		Day-0	Day-1	Day-2
CTX-alone	Daily CTX plus monthly placebo-DP	3	3	3
CTX-DP	Daily CTX plus monthly DP	3	3	3

CTX-alone: Daily, one double-strength tablet of 160mg of sulfamethoxazole and 800mg of trimethoprim plus monthly placebo-DP, given as a fixed dose of 3 placebo-DP tablets daily for three days until delivery.

CTX-DP: Daily, one double-strength tablet of 160mg of sulfamethoxazole and 800mg of trimethoprim plus monthly DP, given as a fixed dose of 3 tablets (40 mg of dihydroartemisinin and 320 mg of piperaquine) daily for three days until delivery.

All participants will (continue to) receive daily cotrimoxazole (CTX) (one double-strength tablet of 160mg of sulfamethoxazole and 800mg of trimethoprim) and anti-retroviral drugs (see section 7.3.1.4, Other concomitant intervention, page 30).

7.3.1.2 Placebos

The study will be placebo-controlled such that each woman will receive an identical number of tablets per day (3) for an identical number of days (3) at enrolment and monthly after that.

7.3.1.3 Methods of drug administration

7.3.1.3.1 Enrolment

The first-day dose of each DP will be given in the clinic under direct observation (day-0). Participants will be given clean water for swallowing the tablets and will then be observed for thirty minutes. Participants will be given the remaining day-1 and day-2 doses to take at home, with instructions to take one set of the pre-packaged tablets on each of the two days. If vomiting occurs in the ANC during the 30 min observation period on day-0, the full dose will be repeated. If the participant vomits again after the repeat dose, she will be instructed to take the next daily dose the following day. Women will be given instructions when going home that if she vomits her drugs within 30 minutes on either day-1 or day-2, she should come back to the clinic or a replacement dose can be brought to her home for 30 min observed dosing of that day's drug. If intolerance continues, she will not be given DP again for that course but receive her next course of DP or DP-placebo during the next monthly visit according to the normal schedule.

Methods to monitor adherence to the 3-day dose regimen are described in section 7.3.5, Strategies to improve adherence to study protocol and medication, page 34.

7.3.1.3.2 Monthly DP dose

Participants will be given each subsequent monthly DP course using the same procedures as for the enrolment course; i.e. the first daily dose is given in the clinic under supervision and the remaining two daily doses will be taken at home. Methods to monitor adherence to the monthly dose are described in section 7.3.5, Strategies to improve adherence to study protocol and medication, page 34.

7.3.1.4 Other concomitant intervention

All participants will (continue to) receive daily cotrimoxazole and anti-retroviral drugs consisting of the fixed combination tenofovir/lamivudine/dolutegravir (TLD) containing Tenofovir (TDF, 300mg) and Lamivudine (3TC, 300mg) and dolutegravir (DTG, 50mg). In addition, all participants will receive a long-lasting insecticide-treated net (LLIN) on enrolment as part of routine antenatal care. The study will ensure the clinics have access to the standard (low-dose of folic acid (0.4mg) as some clinics still use high-dose folic acid (5mg) which may interact with CTX (an antifolate drug).

7.3.1.5 Manufacturing, regulatory approval and supply of study drugs

7.3.1.5.1 Dihydroartemisinin-piperaquine and matching placebo

Dihydroartemisinin-piperaquine and matching placebo will be sourced for this study from Guilin Pharma, based in China which is GMP certified manufacturer of D-ARTEPP®, which is produced as 40/320, 60/480 and 80/640 dihydroartemisinin/piperaquine tablet strengths (see section 16.7 Appendix VII. Product characteristics, page 90). Alternatively, it will be sourced from Alfasigma (formerly Sigma-Tau), Rome, Italy who manufacture DP as Eurartesim® (which makes adult tablet containing 40 mg of dihydroartemisinin and 320 mg of piperaquine).

7.3.1.5.2 Cotrimoxazole (CTX)

CTX is usually provided to HIV-infected participants at government clinics for free. Where required, CTX will be purchased from either Durbin PLC, South Harrow, Middlesex, UK, or other international specialist medical suppliers of quality controlled products that distribute pharmaceuticals worldwide or as generic by other GMP manufacturers (e.g. Universal or Cosmos, Nairobi). Cotrimoxazole is approved for use in Kenya and Malawi under various brand names.

7.3.1.6 Product labelling, shipment and accountability

7.3.1.6.1 Labelling and shipment

Each manufacturer will be responsible for the labelling of the active drug and placebo and will arrange shipment to the respective countries. The study will cover the shipment costs and customs clearance fees. The manufacturers will also provide the certificate of good manufacturing practice (GMP), certificates of analysis for active drug and placebo, where appropriate, the most recent Investigator Brochure, and the summary of product characteristics (SmPC) and the Public Assessment Report (PAR) (released by FDA / EMA), a certificate of donation, and an invoice. All of these are required by the countries to obtain regulatory clearance and an import license for the study drug.

7.3.1.6.2 Packaging

Blister packs or envelopes with the study arm specific active drug and placebo combinations will be prepared by the manufacturers or an equivalent organisation or the study pharmacist, such that one box or envelope contains all the study drugs for that participant. Each box or envelope will contain smaller envelopes each containing a blister pack for a specific course. The specific study drug blisters will be kept in the box/envelope until dispensing. Packaging used for all treatment arms will be identical, labelled and blinded to the contents. The study drugs will be prepared and shipped in at least two different batches, to avoid any shelf life limitations.

7.3.1.6.3 Product storage

All study drugs will be stored in a secure area with access limited to investigators and authorised study site personnel, and under appropriate storage conditions.

7.3.1.6.4 Product accountability

The site-PI will be responsible for establishing a system for the correct handling of study drug to ensure that:

1. Deliveries of study drug from the sponsor are correctly received by a responsible person (e.g. pharmacist assistant)
2. Accurate records are maintained for the receipt of study drug, for the dispensing of study drug to subjects and for the returned study drug.
3. Certificates of delivery and return must be signed preferably by the investigator or authorised personnel and copies retained in the trial site file.
4. The study drug is to be handled and stored safely and properly and in agreement with the given storage instructions.
5. The study drug is to be prescribed only by the principal investigator, co-investigators or study site personnel authorised to do so by the principal investigator.
6. The study drug is dispensed only to study subjects in accordance with the protocol.
7. Subjects must return all unused medication and empty containers to the investigator.
8. At the end of the study, delivery records must be reconciled with records of usage and returned stock. Any discrepancies must be accounted for in writing.

9. Once accounted for, any returned and unused study treatment at the site will be returned to the sponsor for destruction or destroyed locally upon agreement with the sponsor. Drug destruction certificates will be issued that refers to the subject study numbers for subject-specific medication that was destroyed.

7.3.1.6.5 Pharmacist, pharmacist assistant or dispenser

All efforts will be made for the preparation, packaging and labelling of the blinded study drug to be performed and documented in accordance with Good Manufacturing Practice (GMP).

7.3.2 Criteria for discontinuing or modifying allocated interventions for a given trial participant

7.3.2.1 Malaria infection or symptomatic malaria

7.3.2.1.1 Malaria infection diagnosed at enrolment visit

Participants who are febrile or have a recent history of fever or other symptoms of malaria during the enrolment visit will not be excluded. They will follow the standard enrolment and randomisation procedures during that initial visit. In addition, a malaria RDT will be taken. In Kenya, the policy is to screen all women for malaria by RDT or microscopy regardless of the presence of symptoms as part of routine care. In Malawi, this is restricted to symptomatic women. Participants who have uncomplicated malaria confirmed by RDT, regardless of the presence of symptoms, will be treated with a 3-day course of artemether-lumefantrine (AL) for clinical malaria as per national guidelines and will not yet be given DP. The first study specific antimalarial drugs (i.e. the DP or placebo-DP) will then be given during the next scheduled monthly visit approximately four weeks later. This guarantees that both arms (included the CTX-only arm) will receive an active ACT when diagnosed with malaria infection at enrolment. This will be recorded on the CRF. Participants with negative RDT will be given study drug as per randomisation arm.

7.3.2.1.2 Febrile participants during scheduled visits

Similarly, participants in any group who attend a scheduled study appointment with fever and malaria confirmed by RDT will be treated with a 3-day course of AL for clinical malaria. If their scheduled monthly dose of DP or placebo-DP falls within the same three AL treatment days, their DP dose will be delayed until the day after the completion of the last dose of AL. After the delayed course of DP has been taken, they will then continue with monthly DP approximately four weeks later. This will be recorded on the CRF. Participants with negative RDT will continue with the original schedule as per randomisation arm.

7.3.2.1.3 Unscheduled visits

Participants diagnosed with symptomatic uncomplicated malaria during the course of the study will also be treated with AL and where appropriate the next monthly dose of DP or placebo-DP delayed using the same schedule as described in 7.3.2.1.2 Febrile participants during scheduled visits. This will be recorded on the CRF.

7.3.2.2 Allergy to study medication or other safety reasons

Other reasons leading to withdrawal from study interventions include suspected or confirmed allergic reaction to the study drug or for safety reasons as judged by the investigator, study safety monitor or DMEC. See also paragraph 7.3.6, Removal of participants from therapy and/or assessment.

7.3.3 Antiretroviral regimens

7.3.3.1 The rationale for switching cARTs

The counselling and cARTs will be provided by these routine HIV care clinics in each country; the PMTCT/ART clinics in Malawi and the PMTCT/patient support centres (PSC) in Kenya. The study will be conducted during a period when countries are in transition from EFV-based cARTs to DTG-based cARTs in the general population (Malawi) or in restricted populations excluding women of child-bearing potential.

At enrolment, if women in Malawi and Kenya are not yet on DTG-based cARTs (TLD) they will be started on, or switched to (see 7.3.3.2), daily TLD at the time of enrolment, as per WHO's guidelines for preferred first-line treatment in the 2nd and 3rd trimester.

Approximately two weeks post-partum (a period of lactational amenorrhoea when women are unlikely to conceive), women will be counselled by the PMTCT/ART clinics (Malawi) or PMTCT/patient support centres (PSC) (Kenya) about their options for preferred first-line treatment in women of childbearing potential. These options currently vary between Malawi and Kenya, and we will, therefore, follow the existing or upcoming national guidelines at that time in each country. A letter from the secretary of Health and Population In Malawi was recently (March 21, 2019) circulated to all health care providers about an upcoming HIV policy update. This suggests that in Malawi, the new national guidelines will include the provision of a routine counselling visit in the first few weeks post-partum and the option for women of childbearing potential to continue on/use TLD without a requirement for contraception. In Kenya, current guidelines stipulate that this routine counselling visit takes place around two weeks postpartum. EFV-based regimens are currently the preferred first-line treatment for women of childbearing age in Kenya, but it is anticipated that these guidelines may be revised. Currently, the Kenyan guidelines also state that 'women and adolescent girls who are on effective contraception may opt to use DTG and should be supported in their decision.'[49]

All cARTs will be provided by the PMTCT/ART (Malawi) and PMTCT/PSC (Kenya) clinic teams within each study health facilities. The study staff will liaise closely with these clinics to ensure the women receive the appropriate cART postpartum according to the prevailing national guidelines.

7.3.3.2 Method of switching from TLE to TLD during pregnancy and from TLD to TLE after delivery

For known HIV-positive women on TLE, we will use a straightforward switch from the fixed combination of TLE (Tenofovir/Lamivudine/Efavirenz) to TLD (Tenofovir/Lamivudine/Dolutegravir) during pregnancy and from TLD to TLE, in the first few weeks postpartum (about two weeks according to the Kenyan national guidelines). The straightforward switch is based on the switching guidelines provided by British HIV Association guidelines 2016 (page 72),[74] which state that 'Mathematical modelling suggests that dolutegravir concentrations achieved target minimum effective concentrations by 3 days following efavirenz discontinuation (6 days in efavirenz poor metabolisers). Importantly, there was no time point where both the efavirenz and dolutegravir concentrations were predicted to be sub-therapeutic.[75] On that basis, straightforward (with no dose adjustment) substitution of efavirenz with dolutegravir is recommended'.[74] In Kenya, straightforward switching is implemented in individuals with evidence of adequate viral load suppression (<400 copies/mL). If the most recent viral load was >=400 copies/mL a different switching protocol applies in Kenya to differentiate between cART adherence problem and acquired drug resistance. This will be an exclusion criterion.

7.3.4 Permitted and prohibited concomitant medication and care

7.3.4.1 Routine care and permitted concomitant medication

All participants will be offered routine antenatal care according to local policy and the principles of 'focused antenatal care'. This includes the provision of insecticide-treated bed nets (ITNs) free of charge, and screening of malaria at enrolment if this is the local policy (e.g. in western Kenya). Routine antenatal care also includes screening at the first appointment for syphilis, anaemia and HIV, and the provision of appropriate treatment for these conditions. Participants confirmed as HIV-negative prior to enrolment will be excluded from the trial and referred to routine care for assessment or for enrolment in the sister IMPROVE trial in HIV-negative women.

Participants will be counselled to avoid concomitant medications not prescribed by the study clinic, specifically antimalarial drugs or drugs that may be associated with QTc prolongation. All concomitant medications taken during the study will be recorded in the appropriate sections of the CRF with indication, dose information, and dates of administration.

All routine medication used for antenatal care is permitted, with the exception listed under the paragraph 7.3.4.2, Prohibited medication, page 34. Permitted medication includes the use of AL (first line) and quinine or amodiaquine-artesunate (second line) for treatment of uncomplicated malaria, or artesunate or quinine for severe malaria as per national guidelines. As part of their antenatal care participants will be given iron and folic acid supplements (folic acid dose 0.4 mg to 0.6 mg / day) and tetanus vaccination if applicable. Where this is local policy, helminth infections will be treated presumptively with albendazole 400 mg single dose or mebendazole 400mg for hookworm, trichuriasis and ascariasis after the first trimester.

7.3.4.2 Prohibited medication

Prohibited medication includes antimalarial drugs not prescribed within the trial protocol, and other drugs with antimalarial properties including SP, unless prescribed by the study staff. It also includes high dose folic acid supplementation (>1.5. mg/day such as the commonly used 5 mg tablets per day). Randomised participants who take prohibited medications will remain in the trial and will be included in the primary, intention-to-treat analysis, but excluded from the per-protocol analysis.

7.3.5 Strategies to improve adherence to study protocol and medication

7.3.5.1 Adherence to study protocol and medication

Adequate education on the number of tablets to be taken at home on days 1 and 2 of each monthly course will be provided at each contact point during scheduled and unscheduled visit. Where feasible, a take-away pictorial drug administration chart will be provided to aid adherence to the dose and the visit schedule. Where feasible, study participants will be reminded about any follow-up visits through mobile phone contact. Where participants cannot be reached by mobile phones, home visits will be arranged where feasible. All information will be recorded on the appropriate sections of the CRF. Subjects judged to be non-compliant may continue in the study but will be counselled on the importance of taking their study medication as prescribed.

7.3.5.2 Monitoring adherence

Adherence will be monitored by mobile phone interviews and/or text messages after each daily dose of each monthly course. Women without access to a mobile phone will be visited at home to monitor adherence. Further exploratory evaluation of the use of mobile phone technology utilising an Unstructured Supplementary Service Data (USSD) platform to monitor adherence and safety events, shall be implemented in some sites.

In addition, all participants will be visited by a fieldworker at least once to check whether they have taken the tablets and whether they have had any problems. This visit shall also serve to determine the availability, use of LLITNs the previous night, and integrity of LLITNs on a random basis.

7.3.5.3 Strategies for retention

During screening and consent procedures, participants will be asked whether they live in the catchment area, are willing to adhere to the study protocol, and deliver in a study clinic or hospital. Specifically, potential participants will be asked whether they will be willing and able to comply with the frequent follow-up schedule and whether they need to travel out of the study area for an extended period during the follow-up period. The 'study catchment area' will be defined for each study site before the start of the study. All participants will be reimbursed for transportation costs to and from the clinic.

GPS position and otherwise, detailed directions to participants' homes as well as contact information, including mobile phone information, will be recorded at enrolment. If participants do not return for scheduled follow-up visits, the study team will call them and ask them to come to the clinic for evaluation, offering transport reimbursement, or if participants do not have access to a mobile phone, study staff may visit their house to help arrange transport to the clinic if the participants are willing to come to the clinic, or, alternately, a study staff member may go to their home for clinical evaluation and to assess if they still wish to participate in the study. The participants' travel costs will be reimbursed. All information will be recorded on the appropriate sections of the CRF. Subjects judged to be non-compliant may continue in the study but should be counselled on the importance of taking their study medication as prescribed.

7.3.6 Removal of participants from study

7.3.6.1 Removal of participants from therapy and/or assessment

Participants can discontinue from the study for any one of the following reasons:

1. Screening error resulting in incorrect enrolment (found that subject did not meet required inclusion / exclusion criteria)
2. Withdrawal of consent at any stage or subject not willing to continue in the study
3. Suspected or confirmed allergic reaction to the study drug (removal from therapy only)
4. Safety reasons as judged by the investigator, study safety monitor or DMEC (removal from therapy only)

Where feasible, the participants who discontinue from the study treatment or from the study entirely will be asked about the reason(s) for their discontinuation and the presence of adverse events. Participants have the right to unconditionally withdraw from participation in the study without providing a reason. If a subject discontinues, data captured will contribute to the analysis up to the day prior to discontinuation, unless the subject indicates that she withdraws consent for any data captured up to that point. Every effort will be made to follow-up participants who discontinue due to drug related adverse events to determine the birth outcome. If a subject discontinues due to drug-related adverse events, all the assessments that would have been carried out at the next scheduled visit will be conducted at day 28 where reasonably possible (unless consent is withdrawn). This will be recorded in the CRFs. The study drug will be returned by the subject. Subjects that have discontinued the study prematurely will not be replaced.

7.3.6.2 Discontinuation from storage of samples for future studies

The applicability of long-term storage of study samples for future research shall be in respect to the regulations of the country-specific ethics board. When a subject's consent for long-term storage is withdrawn, the stored sample will be destroyed, and the withdrawal noted in the CRF. If the request is received after the dataset has been anonymised, the stored sample can no longer be withdrawn.

7.4 OUTCOME MEASURES

7.4.1 Primary outcome

7.4.1.1 Cumulative incidence of malaria infection

The primary outcome will be the cumulative incidence of malaria infection detected from 2 weeks after the first day of the first dose of the first course to delivery inclusive, defined as the presence of peripheral (maternal) or placental (maternal) Plasmodium infection detected by either molecular diagnostics (henceforth referred to as PCR), microscopy, RDT or placental histology (active infection).

7.4.2 Secondary outcome measures

7.4.2.1 Efficacy

1. Incidence of malaria infection in the peripheral blood during pregnancy defined as the composite of malaria detected in the peripheral blood by RDT (for point of care), or microscopy (not for point of care), or by PCR (not for point of care), excluding enrolment and delivery.
2. The individual components of the composite malaria infection endpoints
3. Incidence of clinical malaria.
4. Malaria infection at delivery, detected in maternal peripheral or placental blood by either molecular diagnostics, microscopy, RDT or placental histology (active infection).
5. Placental malaria by histology (active, past, and active and past infections pooled)
6. Placental malaria by any measure
7. Maternal peripheral malaria infection at delivery by any measure
8. Placental inflammation or chorioamnionitis
9. Adverse pregnancy outcome: the composite of foetal loss (spontaneous abortion or stillbirth), or singleton live births born small-for-gestational-age (SGA), or with low birthweight (LBW), or preterm (PT) (SGA-LBW-PT), or subsequent neonatal death by day 28. Small for gestational age will be defined using the new INTERGROWTH population reference's 10th percentile.[76]
10. Composite of foetal loss and neonatal mortality.
11. SGA-LBW-PT composite.
12. The individual components of the above composites
13. Neonatal length and stunting.
14. Evidence of arboviral infections

7.4.2.2 Safety

1. QTc-prolongation.
2. Congenital malformations.
3. Maternal mortality
4. Other SAEs and AEs.
5. Mother to child transmission of HIV

7.4.2.3 Tolerance

1. History of vomiting study drug (<30 min).
2. Dizziness.
3. Gastrointestinal complaints.

7.4.2.4 Antimicrobial activity and resistance

1. Frequency of molecular markers of drug resistance in *Plasmodium falciparum* infections during pregnancy and delivery.

7.4.2.5 Pharmacokinetic parameters

1. Standard pharmacokinetic parameters for dolutegravir, piperaquine and CTX.

7.5 PARTICIPANTS TIMELINE

7.5.1 Timeline

The total duration of the trial is 24 months. Actual participant recruitment and follow up is expected to take up to 19 months (12 months recruitment plus 7 months of mother-infant follow-up until the child is 6 weeks old) (see 12.1, Timeline, page 62).

7.5.2 Antenatal booking visit

If a woman makes first contact with the study team between 16 and 28 weeks gestation (inclusive), the first study visit will immediately follow eligibility screening. If a woman makes first contact before 16 weeks gestation, she will be invited to be enrolled into the study at the next clinic visit when she would have reached the eligible gestational age where all screening and enrolment procedures will be conducted.

7.5.2.1 Enrolment

After informed consent has been signed, participants will be randomly allocated to either of the two study arms (see section 8.1, Allocation, page 43) and issued with a trial identifier with their unique study identification number.

At this stage study staff will ask the participant for a home or mobile telephone number if they have one, and home address or a description of the location of their home, and for their verbal consent to be called or visited at home whenever they do not attend scheduled appointments.

7.5.2.2 Baseline assessment

A baseline assessment will be conducted for each participant comprising demographic information, socioeconomic information, ITN and IRS use, medical and obstetric history, and clinical assessment. Any relevant information already collected during the screening process or at previous antenatal appointments for the same pregnancy will be copied from the antenatal cards and HIV care and treatment cards onto the CRF, so that questions, tests and examinations are not unnecessarily duplicated, unless for quality control.

Study-relevant information recorded at the first visit will include age; area of residence; prior use of ITNs/IRS; the number of previous known pregnancies and number of previous births and corresponding pregnancy outcomes. Clinical assessment will include screening for TB and opportunistic infections, height and body weight, mid-upper arm circumference, fundal height, reported date of last menstrual period and estimated gestation. An ultrasound scan will be done to determine the gestational age.

7.5.2.3 Biological samples

A venous (up to 12 mL) will be taken and used for both routine and study-specific testing as described in Table 1: Summary Table of Study Design and Schedule of Assessment, page 16. This will be obtained after consent for eligibility screening has been obtained but before consent for enrolment in the trial; participants who have not been tested for HIV and syphilis in their current pregnancy will be tested for HIV and syphilis, and all participants will be tested for anaemia and haemoglobin levels. Participants not previously tested for syphilis in this pregnancy will be tested and treated if positive. The same blood sample will be prepared for study-specific diagnostic testing for malaria (e.g. not for point-of-care in Malawi, and for mRDT screening in Kenya). A filter-paper sample will be prepared and stored for parasite genetic studies. Additionally, baseline assessment tests will be done at enrolment for those starting first-line cARTs (newly diagnosed HIV positive at enrolment) as per the national guidelines and could include full blood count, biochemistry for electrolytes, liver and renal function test. Where feasible maternal viral load tests will be conducted for all women at enrolment. If this was done as part of routine care in the previous 3 months, including any CD4 count, the information will be copied from clinic registers.

In a subgroup of participants enrolled in the nested cardiac monitoring study, an additional 2 mL of whole blood will be required for drug level assessment, full blood count, biochemistry for electrolytes, liver and renal function test, 4-6 hours after the 3rd dose of DP loading dose.

All samples collected will be recorded on case report forms and will include participant ID, type, date, time of sample collection and any other relevant information required by the laboratories. No names will be recorded on any of the biological samples or corresponding CRFs.

7.5.2.4 Study intervention

The first course of study drug will be administered to asymptomatic participants at the visit as described in section 7.3, Interventions, page 29. If participants have malaria, they will be treated as described in section 7.3.2.1. Malaria infection diagnosed at enrolment visit, page 32.

7.5.2.5 Routine antenatal care and treatment of illness

Routine antenatal care and treatment of any illness identified at this visit will be provided as described in section 7.3.4.1, Routine care and permitted concomitant medication, page 34. This will include the provision of an ITN if the woman does not already have one, and advice on sleeping under the net for the entire duration of the pregnancy.

7.5.2.6 Recording of morbidity and medication taken

All participants will be asked about any symptoms or illnesses they have had in the last month, and any medication taken. These will be recorded in the adverse events section of the CRF. All AEs shall also be evaluated for seriousness and reported accordingly.

7.5.3 Interim monthly scheduled visits

Subsequent clinic visits will be scheduled monthly after the first visit. The range of monthly visits will be 3 to 7 visits depending on the gestational age of the woman at enrolment and delivery. Since recruitment is restricted to 16-28 weeks gestation, a median of 4 to 5 monthly visits is expected.

7.5.3.1 Serial ultrasound scans

A subgroup of participants in selected sites shall be requested to undergo two more additional ultrasound scans at interim monthly visits at the approximate gestational ages of 25-28 weeks and 32-35 weeks (or otherwise at the nearest scheduled monthly visit) (subject to funding).

7.5.3.2 Biological samples

A venous (up to 12 mL) will be taken for the same assays as the baseline sample and for maternal viral load during the third trimester visits as described in Table 1: Summary Table of Study Design and Schedule of Assessment, page 16. Where appropriate viral load assay result will be copied from clinic registers if they were done, or are scheduled to be done, in the third trimester as part of routine care. The samples taken for malaria diagnostics, including malaria smears and PCR will be taken for later diagnostic analysis and not for patient care. A standard malaria RDT will be taken from participants with fever or a history of fever in the last 48 hours for patient care (7.3.2.1.2, Febrile participants during scheduled visits, page 32).

At each scheduled visit, the subgroup of women enrolled in the cardiac monitoring study will have an additional 2mL of whole blood drawn 4-6 hours after the third dose of a monthly course; for the assessment of drug levels, full blood count, and biochemistry for electrolytes, liver and renal function tests. The sample will be drawn just after the respective ECGs have been taken at these time points.

7.5.3.3 Study intervention

The monthly DP or placebo will be administered at these visits as described in section 7.3.1.1, Study arms, page 29. If women are symptomatic with evidence of malaria confirmed by RDT they will be treated as described in section 7.3.2.1.2, Febrile participants during scheduled visits, page 39.

7.5.3.4 Routine antenatal care and treatment of illness

Routine antenatal care and treatment of any other illness identified at this visit will also be provided as appropriate.

7.5.3.5 Recording of morbidity and medication

Participants will be asked about any symptoms or illnesses they have had since the start of pregnancy, and any medication is taken, and these will be appropriately documented in the CRFs.

7.5.4 Unscheduled visits during pregnancy

Participants will be encouraged to visit the clinic if they feel unwell between scheduled appointments. Participants who present between appointments will be examined by study staff. Presenting symptoms, axillary temperature and blood pressure will be recorded. A venous blood sample (upto 6mL) will be taken for Hb measurement (if clinically indicated) and malaria RDT for point of care (if clinically indicated), and a malaria smear and dried blood spot for later analysis of malaria. A 5mL urine sample will also be taken for detection of urinary tract infections if clinically indicated and for viral infections. Any illness will be treated as appropriate and according to standard local care. The date of attendance, diagnosis and treatment will be recorded on the CRF, and any adverse events reported according to standard procedures.

7.5.5 Delivery visit

Participants will be encouraged to deliver on the maternity wards of the participating clinics, in which case they will be assisted to deliver by regular clinic midwives, and a specially trained staff will visit them on the ward and perform relevant examinations shortly after delivery. Participants who deliver at home will be identified using a network of community health workers such as health surveillance staff or village health volunteers/workers and will be requested to report to the health facility within 48 hours, to collect, as far as possible, the same information. Infants will be referred to the PMTC teams in the hospital to initiate cART prophylaxis in line with the existing national guidelines as soon as the first contact with the mother and infant after delivery. The current infant prophylaxis consists

of 12 weeks in which zidovudine (AZT, Retrovir) and nevirapine (NVP) are given for the first six weeks, followed by six weeks of daily NVP.

7.5.5.1 Biological sampling mother

A similar venous (up to 12 mL) blood sample (~250 µL) will be taken as for the scheduled follow-up visits for the same assays as described in Table 1: Summary Table of Study Design and Schedule of Assessment.

7.5.5.2 Placental sampling

A blood sample will be collected from the delivered placenta by making an incision on the maternal side and collecting the pooled blood. This sample will be tested for malaria using standard microscopy of blood smear and PCR, and drug levels.

A 2cm x 2cm x 1cm specimen of placental tissue will be taken from the maternal side for histopathology testing for current and past malarial infection. An amnion roll will be included in the collection of the placental sample for histological evaluation of chorio and/ or amnionitis.

7.5.5.3 Umbilical cord sampling

An umbilical cord blood sample will be taken for haemoglobin level, malaria molecular diagnostics, antibody and cell mediated immune responses to malaria and other pathogens such as viral infections and drug levels. We will collect up to 300ml of cord blood where feasible. At term, the maternal blood that flows through the placenta is approximately 600-700ml/min[77.] At the time of delivery, delayed cord clamping as per obstetrics standard of care allows blood to flow from the placenta to the baby and thus reducing placental volume by approximately half. Where feasible, we will collect the remaining cord blood in a blood transfusion bag which can be up to approximately 300mL.

7.5.5.4 Examination of the baby

All babies shall be examined for vital status, jaundice and the presence of congenital abnormalities. Birthweight, using digital scales, head circumference, length, and umbilical abdominal circumference shall be measured.

7.5.6 Postnatal visits

7.5.6.1 Seven-day postnatal visit

Participants and their babies will be seen at the clinic by a study nurse around seven days after birth. The baby will be examined for the presence of jaundice and for any congenital anomalies that may have been missed at delivery and basic anthropometric measures taken. A heelprick sample (about 500 microL) will be taken in all babies with fever or other symptoms for testing by RDT, microscopy and DBS, and in a subgroup of asymptomatic babies for assays to assess immune responses to malaria and other infectious diseases. All treatment in symptomatic infants will be provided based on the RDT results. Standard postnatal advice and healthcare will be provided as needed for mother and baby including infant ARV prophylaxis and mother adherence to cART during breastfeeding, and any treatment and clinical findings for the baby recorded on the CRF. Routine postnatal care will be provided for the participants.

Participants who do not attend this appointment will be visited at home if possible. Any infant deaths occurring before the visit will be recorded; the date and probable cause of death will be ascertained by verbal autopsy.

7.5.6.2 Six,- to eight-week postnatal visit

Participants and their babies will be seen again at the clinic around six to eight weeks after birth, at their baby's second vaccination visit. The baby will be examined for the presence of congenital anomalies that may have been missed at delivery or the first visit.

A similar heel prick sample (about 500 microL) as on day 7 will be taken again for malaria diagnosis and for immunological assays with treatment provided based on the RDT results. Part of the sample will be used for DNA PCR for HIV virus detection at 6 weeks in line with national and/or WHO guidelines. Standard postnatal advice and healthcare will be provided as needed for mother and baby, and any treatment and clinical findings for the baby recorded on the CRF. Routine postnatal care will be provided for the participants.

Participants who do not attend the scheduled appointment will be visited at home if possible. Any infant deaths occurring before the visit will be recorded; the date and probable cause of death will be ascertained by verbal autopsy.

7.5.6.3 Unscheduled postnatal visits

Participants will be encouraged to attend the clinic if they or their baby become unwell during the follow-up postnatal period. Physical examinations and relevant blood samples will be conducted to guide management. Any illness will be treated as appropriate and according to standard local care. The date of attendance, diagnosis and treatment will be recorded on the CRF, and any adverse events reported according to standard procedures. Clinical care will also be provided for participants who attend because of their own illness, but this will not be recorded as part of the study.

In a sub-group of participants seen during sick visits, dried blood spots or red pellets will be taken and analysed for detection of molecular markers of anti-malarial resistance.

7.6 SAMPLE SIZE AND POWER

7.6.1 Primary outcome

The primary outcome will be the cumulative incidence of malaria infection detected from 2 weeks after the first day of the first dose of the first course to delivery inclusive, defined as the presence of peripheral (maternal) or placental (maternal) Plasmodium infection detected by either molecular diagnostics (henceforth referred to as PCR), microscopy, RDT or placental histology (active infection). A sample size of 898 (449 per arm) (359 contributors and 90 non-contributors) is required to detect a 50% reduction (RR=0.50) from 12% in the CTX-alone arm (control arm) to 6% in the intervention arm with 80% power (alpha=0.05) allowing for 20% non-contributors (N=90). The effect size of 50% is more conservative than the 68% pooled effect size observed in fixed effects meta-analysis from the three completed trials to date that compared IPTp-DP vs IPTp-SP in HIV-negative women (95% CI 61% to 73%, I-squared=0%, P<0.0001).[29,30,72]

7.6.2 Power calculations for key secondary outcomes

7.6.2.1 Adverse pregnancy outcome

A key secondary outcome is 'adverse pregnancy outcome' defined as the composite of foetal loss (spontaneous abortion or stillbirth), or singleton live births born small-for-gestational-age (SGA), or with low birthweight (LBW), or preterm (PT) (SGA-LBW-PT), or subsequent neonatal death by day 28. The sample size of 718 contributors (359 per arm) provides 80% power to detect a 27.6% reduction (RR=0.724) in adverse pregnancy outcomes from 34.5% with CTX-alone (control) to 25.0% in the DP arm (alpha=0.05). The 34.5% proportion for this outcome in the control arm (CTX-alone) is based on

data from our recently completed pregnancy trials in Malawi[78] and Kenya.[30] The frequency of this outcome was 23.0% in HIV-negative women receiving IPTp-SP. It was assumed that this outcome is at least 1.5 times more common in HIV-infected women on cART (i.e. 1.5x23.0=34.5%), based on a recent meta-analysis comparing the risk of low birthweight and pre-term delivery in HIV-infected vs HIV-negative women.[8]

7.6.3 Sample size for cardiac monitoring study

Overall, 80 women will be recruited per arm (160 in total). Each woman will have a total of six ECGs taking as three sets of two ECGs; one before and one 4-6h after the first course at enrolment and then again one before and one 4-6h after two of the subsequent monthly courses spaced at least one month apart throughout the pregnancy. The research question of interest concerns the QTC prolongation at enrolment. Previous studies have shown that most of the piperaquine related QTc prolongation occurs after the first course of DP with a subsequent decline in QTc prolongation with subsequent monthly courses.[60,79] However, since the study is placebo-controlled, women from both arms will need to be recruited. A sample size of 80 in the CTX-DP and 80 in the CTX-alone arm will provide 90% power to detect a 10ms difference in QTc prolongation (e.g. 30 vs 20ms) after the first course (two-sided $\alpha=0.05$, standard deviation 18.3ms, t-test for independent samples, allowing for 10% non-contributors [e.g. due to missing ECG at Tmax on Day-2]). The standard deviation of 18.3ms is based on our recent observations in our recently completed trial in Indonesia (ter Kuile et al., unpublished observations).

7.7 Recruitment and retention

7.7.1 Recruitment procedures

Eligible women who are resident in the study area will be recruited when they start attending antenatal care in one of the ANC clinics in the participating hospitals. The study will provide staff and dedicated study staff to deal with the additional workload. Where required, the trial will fund minor improvements in the infrastructure, such as the refurbishment of a dedicated study room, or pay rent to the hospital to use facilities.

7.7.2 Planned recruitment rate

Recruitment of 898 (449 per arm) women (all gravidae) from 2 countries will require 12 months, with an additional seven months to complete pregnancy plus infant follow-up. The recruitment rate of 37 HIV-positive women per month is feasible in the hospitals where the larger sister trial is ongoing in Malawi and Kenya.

7.7.3 Rate of loss to follow-up

This trial will be conducted by the same study teams and use similar outcomes as in the previous trials in these countries.[30,78,80] The sample size allows for 20% loss to follow-up, which is conservative as the primary endpoint is the cumulative incidence of malaria infection and most women are expected to contribute observation time to this endpoint during pregnancy even if observations at the time of delivery may be missed.

7.7.4 Problems with compliance and Strategies for retention

7.7.4.1 Strategies for retention

During screening, women will be asked whether they live in the catchment area, are willing to adhere to the study protocol, and deliver in a study clinic or hospital. GPS position and otherwise, detailed

directions to participants' homes as well as contact information, including mobile phone information, will be recorded at enrolment. If women do not return for scheduled follow-up visits, the study team will call them and ask them to come to the clinic for evaluation, offering transport reimbursement, or if women do not have access to a mobile phone, study staff may visit their house to help arrange transport to the clinic if the women are willing to come to the clinic, or, alternately, a study staff member may go to their home for clinical evaluation and to assess if they still wish to participate in the study. The women's travel costs will be reimbursed. All information will be recorded on the appropriate sections of the CRF. Subjects judged to be non-compliant may continue in the study but should be counselled on the importance of taking their study medication as prescribed.

7.7.4.2 Drug adherence

The first dose of each course will be given under supervision of study staff on day 0, and the remaining 2 doses for days 1 and 2 will be taken at home by the study participants. Participants will be reminded through mobile phone contact to take the assigned tablets for the second and third dose. A sub-sample of participants will be visited at home by random spot checks (some on Day 1 and other participants on Day 2, with Day 0 being the first day of treatment) to assess adherence to the study drugs. In our previous trial in western Kenya documented adherence using this method was very high (>95%).[30] Adherence to dosing will be assessed by questionnaire during each monthly scheduled visit, and by random spot checks at home or by mobile phone. Where feasible a drug administration pictorial chart will be provided to aid adherence in between scheduled visits.

8 METHODS: ASSIGNMENT OF INTERVENTIONS

8.1 ALLOCATION

Balanced randomisation will be used using permuted block randomisation methods stratified by site (i.e. hospital) and two HIV-status groups (known-positive and newly-diagnosed). A statistician at LSTM in the UK will generate the randomisation lists using dummy coding (e.g. A, B), and another independent statistician who is not involved in the study will assign the dummy codes to each of the two study arms. The list with the allocation sequence will then be forwarded to the trial pharmacists based in Kenya and Malawi who will then prepare sequentially numbered, sealed, opaque boxes or envelopes for each participant with the randomisation assignments. Contained within each of these envelopes or boxes will be the pre-packed investigational product for the entire duration of the study for that participant. These opaque boxes/envelopes will be opened sequentially upon enrolment of a study participant in each site. Stratification by HIV-status (known-positive vs newly-diagnosed) is essential to ensure balanced distribution among study arms as women newly diagnosed with HIV will not yet be on daily cotrimoxazole and may thus have a higher risk of malaria at enrolment. The latter is an important determinant of the primary outcome of malaria infection. Minimisation or distance randomisation using central randomisation services by internet is not feasible because of the unreliable internet connections and telephone services in some of the proposed sites.

8.2 BLINDING AND UNBLINDING

8.2.1 Blinding

The study will placebo-controlled involving a placebo for DP, which will be identical in size, shape and colour to the active DP tablet to ensure blinding of all investigators and study staff during allocation and for the duration of the trial. To further minimise bias, we will use an objective primary outcome measure and mask all laboratory staff to the treatment assignment of individual women. The trial

statistician will also be blinded regarding the treatment code when he develops the statistical analysis plan and writes the statistical programmes, which will be validated and completed using dummy randomisation codes. The actual allocation will only be provided to the study team after locking of the database for the primary outcome, and after the statistical analysis plan has been signed off by the Chief Investigator, the trial statistician, and by the independent Data Monitoring and Ethics Committee (DMEC) and a signed copy has been e-mailed to them for their records.

8.2.2 Emergency unblinding

Emergency unblinding will be on an individual basis to ensure the blinding of other participants is not affected. Emergency unblinding will be considered in these instances: suspected unexpected serious adverse reaction to the study drug (SUSAR), serious adverse reaction to the study drug, or serious expected adverse drug-drug interactions between the study drug and other drugs provided to the participant undergoing treatment, as judged by the study physician or study safety monitor. The first alert will be raised by the study physician within 24 hours of becoming aware of the event in an expedited report on the SAE form (10.2.4.1, Expedited reporting, page 52) to the chief investigator, ethics committees, safety monitor, sponsor and DMEC chair. The final decision for emergency unblinding will be advised by the country Principal investigator and/or the Chief investigator in consultation with the safety monitor and/or DMEC chair. The trial pharmacist in each country will be responsible for the safekeeping of the treatment allocation of each participant and will be contacted by phone and email by the principal investigator and/or chief investigator if emergency unblinding will be advised. Furthermore, the allocation code for each participant is also kept by the independent statistician at LSTM in the UK who is not involved in the study as an investigator. This independent statistician will be available when emergency unblinding is requested by the DMEC chair. The date, reason and name of the person breaking the code must be documented. If so clinically indicated, the subject will be withdrawn from receiving further study drug. Other than the written or verbal disclosure of the code in any of the confidential correspondence about the participant between the principal or chief investigator and the safety monitor or the DMEC chair, the actual allocation will NOT be disclosed to the participant and/or other study personnel including other site personnel, monitors, corporate sponsors or project office staff.

9 METHODS DATA COLLECTION, MANAGEMENT AND ANALYSIS

9.1 MEASUREMENT OF EFFICACY OUTCOMES AND SAFETY

9.1.1 Efficacy

9.1.1.1 Malaria infection

In febrile participants (women and their new-borns) or those with a history of fever in the last 48 hours, RDTs will be used for point-of-care diagnosis at all scheduled and unscheduled visits, with RDT-positivity defined as either pLDH or HRP2 antigen positivity. For all participants, including those who are asymptomatic, samples will be taken for the later determination of the presence of malaria infection by PCR or LAMP, microscopy, and RDTs. This will be conducted in central laboratories (i.e. not at point of care and not for participant's care). At delivery, additional samples will be collected for placental histology (active and past malaria infections) and later detection of the presence of malaria infection in cord blood.

9.1.1.2 SGA-LBW-PT

New-borns will be weighed within 24 hours of delivery using digital scales (± 10 gr) and LBW defined as < 2,500g. If the birthweight cannot be obtained within 24 hours, birthweights obtained up to day 7 inclusive can be used and a correction factor to adjust for the physiological fall in birthweight will be applied. Gestational age will be assessed using ultrasound dating at enrolment and preterm (PT) defined as <37 weeks gestation also within 24 hours, and otherwise within 96 hours. Small for gestational age (SGA) will be defined as birthweight below the tenth percentile for a given gestational age and sex, using the new INTERGROWTH-21st reference population.[76] This will also allow the calculation of z-scores.

9.1.1.3 Pregnancy outcome and neonatal death

Foetal loss will be assessed monthly at scheduled ANC visits. If women do not turn-up they will be contacted by mobile phone and/or visited at home to document this. Foetal loss before 28 weeks gestation will be defined as induced or spontaneous miscarriage, and foetal loss at or after 28 weeks gestation as stillbirth. Neonatal death will be assessed at the 6-week follow-up visit. Neonatal mortality will be defined an infant death within 28 days if the date of birth and date of death are known, and otherwise within 1 month (30 days) if one of these dates are not exactly known.

9.1.2 Safety

9.1.2.1 Congenital abnormalities, neonatal jaundice and vital status

The presence of congenital abnormalities and jaundice will be assessed at delivery, day 7, and at 1 month. The vital status of participants will be recorded at each scheduled and unscheduled visit, and through follow-up phone calls and home visits if participants do not attend the scheduled visit. Maternal mortality will be defined as the death of a woman while pregnant or within 42 days of termination of pregnancy, irrespective of the duration and site of the pregnancy, from any cause related to or aggravated by the pregnancy or its management but not from accidental or incidental causes. A verbal autopsy questionnaire will attempt to determine the cause of death.

9.1.2.2 Mother to child transmission of HIV

The occurrence of mother to child transmission of HIV will be assessed at delivery and at week 6-8 post-natal visit. Maternal viral load will be measured, at enrolment, third trimester visit and/or delivery visit. Infant blood samples for DNA PCR for HIV virus detection will done at 6-8 weeks in line with national and/or WHO guidelines for both study arms. Where available results will be obtained from clinic registers if these assays are done as part of routine care to avoid duplication.

9.1.2.3 Routine monitoring cART tolerance and safety

As part of routine care, women initiating cART will be monitored closely as part of their routine HIV care for development of adverse drug events and to identify and address barriers to adherence. This includes a complete medical history taken, a thorough physical examination and appropriate laboratory investigations, which includes viral load, haemoglobin assessment (or full blood count where available), and renal and liver function tests. As this is part of routine care, and related to the study, this will be conducted by routine clinic staff, not by study staff. The information will be copied for the routine clinic registers.

9.1.2.4 Procedures for cardiac monitoring

9.1.2.4.1 Overview

Cardiac monitoring will be conducted in the first 160 women (80 in each arm) who provide informed consent to join this component of the study. The objective of this cardiac monitoring sub-study is

twofold; 1) to determine whether there is a difference in QTc prolongation between the CTX-alone arm and the CTX-DP arm. 2) to determine whether the magnitude of DP related QTc prolongation on the ECG, observed between the pre-treatment value (day 0) and the QTc time 4-6 hours after the 3-day IPTp-DP course (peak piperaquine levels) increases with each subsequent course, and to determine if this is different compared to HIV-negative women getting monthly IPT with DP. A venous sample for drug levels will be taken at the same time to determine the relationship between piperaquine level and magnitude of QTc prolongation and determine the impact of monthly dosing on hepatic and renal function.

9.1.2.4.2 Electrocardiogram (ECG)

Each woman will have a total of six ECGs taking as three sets of two ECGs; one before and one 4-6h after the first course at enrolment and then again one before and one 4-6h after two of the subsequent monthly courses spaced at least one month apart throughout the pregnancy. ECG will be conducted by study staff at the clinic and results read by local clinicians; Approximately half of ECGs will also be read off-site by an experienced cardiologist (Cardiabase, Banook Group, Nancy, France). The following stopping rules will be applied as a contraindication for subsequent IPTp courses with DP. 1) Confirmed QT values >480ms & QTc>500ms combined with a heart rate of <65bpm and >60ms increase in QTc relative to baseline, or 2) any QT>480ms combined with a heart rate of <65bpm in a woman with syncope or seizures within 24 hours of the monthly dose. In such events, women will continue in the study but will on receive daily CTX. The results of the external expert reading of ECGs will be available on a secure server (provided by the study or by the external experts, e.g. by Cardiabase). The external experts and the site Principal Investigator will be alerted by e-mail as soon as the ECGs and results respectively have been uploaded onto the server. In the event of any findings with clinical significance, participants shall be traced and requested to report to the study clinic as soon as possible for repeat ECG and communication of the findings and appropriate clinical management.

9.1.2.4.3 Plasma piperaquine levels, full blood count, renal function and liver function

A few minutes after the last ECG (i.e. 4-6 hours after the last dose of each course), a 2 ml venous blood sample will be obtained for piperaquine, cARTs and CTX drug levels to determine the relationship between drug level and magnitude of QTc prolongation. The sample will also be used to determine the impact of monthly dosing on full blood count, renal and hepatic function. Furthermore, at delivery, maternal placental blood and a cord blood sample will be used for the same drug levels to quantify drug exposure to placental parasites and the neonate, respectively.

9.1.3 Pharmacokinetic studies and drug-drug interactions

We will conduct pharmacokinetic studies in a subset of the first 80 of the 160 women undergoing cardiac monitoring who provide informed consent for the additional sampling. The blood sample will be used to measure drug levels of piperaquine, cotrimoxazole, DTG and other cARTs to determine the pharmacokinetic profile of these drugs and any clinically relevant drug-drug interactions when co-administered. All these women will also take part in the cardiac monitoring study to determine the association between QTc prolongation and drug levels (for further details, see 16.6.1, Pharmacokinetic studies of drug-drug interactions between piperaquine and standard antiretroviral therapy, page 84).

9.1.4 Laboratory assays

A detailed description of the assays and analyses are provided in section 16.6, Appendix VI. Description of other clinical and laboratory methods, from page 84 onwards. Some laboratory assays will be conducted at collaborating institutions in Australia, United States of America, Canada, United Kingdom, and Europe.

9.2 DATA COLLECTION METHODS & STORAGE

9.2.1 Data management

9.2.1.1 Methodologies for data collection / generation

This will be combined with the data management of the larger sister trial in HIV-negative women. In brief: A combination of paper-based and electronic record forms will be used. For the questionnaires that are administered in paper format, the study will utilise HP Teleform software to design the paper-based CRFs for semi-automated transcribing into an electronic database using scanning and Optical Character Recognition, intelligent document recognition and data validation using checksum algorithms, cross-field validation, and human verification of information against source documents. Once validated, the data will be transferred to the target database along with a PDF of the original image of the CRFs, such that there is an electronic copy of all paper-based documents. For the electronic-only data capture, such as home visits to administer drug adherence questionnaires, we will use tablets with integrated sim cards to transfer encrypted data to the ODK servers at LSTM, UK. This has worked well in previous studies.

9.2.1.2 Data quality and standards

The quality of questionnaire data collection and data entry will be maximised through training of field staff in the standardised questionnaire administration methodology. Field staff will be trained in the methodology for collecting data and will be expected to demonstrate competence before conducting fieldwork. All electronic CRFs and data validations processes for data captured through Teleform will incorporate range and consistency checks.

9.2.1.3 Managing, storing and curating data

Verified and validated data from both countries will be stored via the cloud on a secure, highly fault tolerant, storage area network servers at KEMRI and at LSTM in Liverpool, which is accessed by a series of blade servers configured in a high availability cluster. The PIs from each site will have access to their site data on the central server. All country PIs will have full access to their country level data. Locally, data will be backed-up on a continuous basis on a secure off-site server in Liverpool, and on encrypted standalone hard drives.

Once the data validation phase is completed by the central data manager in Liverpool, the database will be locked and transferred to a statistical programmer at LSTM who will do further syntax-driven consistency checks and syntax-driven data cleaning (e.g. in Stata). The statistical programmer will have access to the PDF copies of the source data. He/she will then prepare the database for data analysis by the trial statistician by creating the final variables for data analysis, such as the creation of the composite endpoints. The final cleaned database will be available as SAS, STATA and in SPSS format, with an embedded data dictionary.

9.2.1.4 Metadata standards and data documentation

The full study protocol, supporting documents including the data management Standard Operating Procedures (SOPs) and the full individual participant level database will be made publicly available once the study findings have been published. The data manager and statistical programmer will produce a document summarising the methods used to generate the data with a full description of all procedures, analyses, data capture tools, coding and description of variables. This document will be published alongside the research database.

9.2.1.5 Data preservation strategy and standards

The majority of the data collected will be captured using scannable paper-based forms. The scanned PDF copies will be used for archiving, and the original paper questionnaires kept in a dedicated lockable data storage room in country and then destroyed after a minimum period subject to the prevailing laws in each country. The research data will be stored in the long-term in the original electronic format, in a unified large database and a public database that contains all research data other than participant identifiable data. The public database will be updated when needed if software becomes obsolescent to achieve long-term preservation. The data will be preserved in this way for 10 years or longer if still being accessed at that stage.

9.3 STATISTICAL METHODS

9.3.1 General principles

All statistical analyses will be described in detail in the Statistical Analysis Plan (SAP) and finalised and signed before unblinding the study. One interim analysis and/or one sample size re-estimation will be conducted as described in section 7.6.1, Sample size and power, page 41 and section 9.3.7, Interim sample size re-estimation and/or interim analyses and criteria for termination of the trial, page 49. The study is designed as a superiority trial, so all tests will be two-sided and unless otherwise defined, *P*-values <0.05 will be used to define statistical significance for analysis involving comparisons between the control arm and the intervention arm for the primary and secondary outcomes. The analysis will be done using Stata.

9.3.2 Analyses populations

Primary analyses will be based on the Intention-to-treat (ITT) population and secondary supportive analyses will be done on the Per-protocol (PP) population. The safety analysis will be performed on the safety population. These analytical populations are defined as follows: 1) The ITT population consists of all randomised subjects with valid informed consent; 2) The PP population is a subset of the ITT population, where subjects with major protocol deviations will be excluded. Major protocol deviations will be defined in the Statistical Analysis Plan (SAP). 3) The safety population is a subset of the ITT population, consisting of all randomised subjects who receive at least one dose or a partial dose of study drug.

9.3.3 Primary outcome analyses

The generalised linear model (GLM) with the log-link function and binomial distribution (log-binomial regression) will be used to analyse the primary outcome. The GLM model will have the treatment arm as the only predictor variable and the balancing factors site and HIV-status (known-positive and newly-diagnosed) as covariates, from which the risk ratio (RR) and its 95% confidence intervals (CI) of having a primary outcome will be derived. Covariate-adjusted analyses for the primary outcome will also be conducted by adding other pre-specified covariates into the above unadjusted GLM analysis to derive the adjusted RR (95% CI). Imputation for baseline missing covariates will be made for covariate-adjusted analysis. These same covariates will be included in subgroup analyses. Assessment of the homogeneity of treatment effect by a subgroup variable will be conducted by a GLM with the treatment, subgroup variable, and their interaction term as predictors, and the *P*-value presented for the interaction term. In the case that convergence with the interaction models cannot be achieved, this will be done by separate GLM models for each category of a subgroup, and the difference between subgroup compared using the Altman-Bland method.[81] In addition, the risk difference (RD) of the primary outcome will be derived from the overall and country stratified GLM models with binomial distribution function, and identity link functions, respectively.

9.3.4 Secondary outcomes analyses

All secondary outcomes will be analysed as a superiority design and two-sided 95% CIs for the treatment differences in these outcomes between two treatment groups will be calculated and presented. Secondary outcome analyses will be based on the ITT population unless specified. The secondary outcomes will also be analysed using the same GLMs will treatment arm as the only predictor variable and the balancing factors site and HIV-status (known-positive and newly-diagnosed) as covariates. The point estimate of the treatment effects with their two-sided 95% CI will be derived via the specification of a GLM model for each secondary outcome depending on its distribution. For a binary outcome such as the components of the primary outcome, similar analysis to the primary outcome analysis will be performed and the treatment effects measured as RR and RD will be generated. For a continuous outcome like birthweight, a Gaussian distribution will be assumed, and an identity link function will be used. Correspondingly the mean difference and its 95%CI between two arms will be calculated. For a count outcome such as the number of SAEs or number of malaria infections or clinical malaria episodes after enrolment, Poisson distribution and log link function will be used (or negative binormal distribution if overdispersion is found), from which incidence rate ratio (IRR) and its two-sided 95% CI will be computed.

9.3.5 Subgroup analyses

We will perform subgroup analyses of the primary outcome and its components on a series of pre-specified baseline characteristics of women including study country, gravidity (paucigravidae [1st and 2$^{nd]}$ vs multigravidae [3rd+]), malaria status (positive-negative, based on a composite of microscopy and PCR), socio-economic status (terciles), HIV-status (known-positive and newly-diagnosed) season (terciles of average rainfall last 6 months before delivery) and viral load (1,000 copies/ml), and study site (i.e. hospital) characteristics: malaria transmission intensity (terciles), expressed as prevalence of PCR positivity on enrolment across two arms; and degree of SP parasite resistance (as a proxy for CTX resistance), expressed as prevalence of the *Pdhps*-A581G mutation as a proxy 'sextuple' *dhfr/dhps* mutant parasites using (e.g. >30% *Pdhps*-A581G). We will also conduct subgroup analysis for the effect of the number of monthly doses received (e.g. median). The definition of subgroups in the statistical analysis plan will supersede those in the protocol. The treatment effect within each category of the above-selected variables will be estimated and the interaction effect between treatment and each variable assessed to explore effect modification. The modifying effects of bednets cannot be assessed as all women will receive LLINs on enrolment as part of the national policy.

9.3.6 Safety

For the safety analysis, women in the DP arm will be considered by arm and by both DP arms pooled (overall) and by the number of DP courses received to explore dose-responses, and the mean QTc change before the 1st dose and after the last dose of each course compared using GLM.

9.3.7 Interim sample size re-estimation and/or interim analyses and criteria for termination of the trial

9.3.7.1 Sample size re-estimation

One interim sample size-estimation will be conducted when approximately 50% of participants have been recruited to verify if the trial is not under-powered based on potential differences between the observed event and dropout rate pooled across the two arms (i.e. blinded) and the estimates of these pooled rates used in the sample size calculations, which were based on historical data from the study areas. To maintain Type 1 error and trial integrity and safeguard the power of the trial, this will be

conducted blinded; i.e. the effect size will not be taken into consideration as no details by arm will be provided to the statistician conducting the sample size re-estimation

9.3.7.2 Interim analysis

At the discretion of the DMEC, an interim analysis will be conducted on the primary outcome when approximately 75% of participants have enrolled or at a time point suggested by the TSC or DMEC. The interim analysis will be performed by an independent statistician, blinded for the treatment allocation. The statistician will report to the independent DMEC. The DMEC will have unblinded access to all data and will discuss or report the results of the interim-analysis with the Trial Steering Committee (TSC), e.g. in a joint meeting. The TSC decides on the continuation of the trial and will report to the ethics committees.

The Lan-DeMets spending function with O'Brien-Fleming type boundaries will be employed to preserve the overall one-sided type I error rate for efficacy at the final analysis. If the stopping boundary is crossed at the interim analysis and the RR is less than 0.5, it will be concluded that the study has demonstrated that the efficacy of the intervention is superior to that of IPTp-SP in the prevention of the primary outcome. The trial recruitment can then be stopped unless the DMEC advises otherwise Statistics will not be the sole basis for the decision to stop or continue and the DMEC can advise to continue recruiting in the trial, or stop recruiting but continue to complete the intervention as per randomisation in the remaining active participants, even if statistically the stopping boundary is crossed, e.g. in order to continue collecting more safety information or data for further sub-group analyses etc. The trial can also be stopped in case of futility.

A detailed plan for interim analysis, the provisional stopping rules and how the stopping rules will be applied, will be drawn up prior to the start of the interim analysis and documented in the study statistical analysis plan.

10 METHODS: MONITORING

10.1 DATA MONITORING AND TRIAL OVERSIGHT COMMITTEES

10.1.1 Data Monitoring and Ethics Committee (DMEC)

Since the study is a clinical trial an independent Data Monitoring and Ethics Committee (DMEC) will be set up. The DMEC will be critical to ensure that the subjects are protected from harm, while also ensuring that the study integrity is not compromised. The DMEC will consist of 3 to 4 independent members knowledgeable in the conduct of clinical trials. They will meet regularly (e.g. twice yearly or more frequently if so required) during data collection period to provide a review of blinded (and if requested unblinded) data to ensure the safety, rights and well-being of trial participants. In addition, regular review of the quality of the study data will be conducted at each meeting of the DMEC.

The roles and membership of the DMEC are described in more detail in Appendix II. Terms of reference oversight committees, section 16.2.3, page 77.

10.1.2 Trial Steering Committee (TSC)

The trial will also have a Trial Steering Committee (TSC) which will advise on the study amendments, monitor progress and quality of the trial, ensure participants' rights and safety are adhered to, review relevant new information from other sources and consider recommendations from the DMEC. The role and membership of the TSC are also described in more detail in section 16.2.2, Appendix II. Terms of reference oversight committees, page 75.

10.2 Safety Monitoring and reporting

The principles of ICH GCP require that both investigators and sponsors follow specific procedures when notifying and reporting adverse events or reactions in clinical trials.

10.2.1 Definitions

The following definitions apply to this protocol:

10.2.1.1 Adverse Event (AE)

Any untoward medical occurrence in a patient or clinical trial subject administered a medicinal product and which does not necessarily have a causal relationship with this treatment.

10.2.1.2 Adverse Reaction (AR)

Any untoward and unintended response to an investigational medicinal product related to any dose administered.

Comment: All adverse events judged by either the reporting investigator or the sponsor as having a reasonable causal relationship to a medicinal product would qualify as adverse reactions. The expression ‘reasonable causal relationship’ means to convey, in general, that there is evidence or argument to suggest a causal relationship.

10.2.1.3 Serious Adverse Event (SAE) or Serious Adverse Reaction (SAR)

Any adverse event or adverse reaction that results in death, is life-threatening*, requires hospitalisation or prolongation of existing hospitalisation, results in persistent or significant disability or incapacity, or is a congenital anomaly or birth defect.

Comment: Medical judgement should be exercised in deciding whether an adverse event/reaction should be classified as serious in other situations. Important adverse events/reactions that are not immediately life-threatening or do not result in death or hospitalisation but may jeopardise the subject or may require intervention to prevent one of the other outcomes listed in the definition above should also be considered serious.

*Life-threatening in the definition of a serious adverse event or serious adverse reaction refers to an event in which the subject was at risk of death at the time of the event. It does not refer to an event which hypothetically might have caused death if it were more severe.

10.2.1.4 Suspected Unexpected Serious Adverse Reaction (SUSAR)

An adverse reaction that is both unexpected (not consistent with the applicable product information) and also meets the definition of a Serious Adverse Event/Reaction.

10.2.1.5 Intensity

The intensity of each AE recorded in the case report form should be assigned to a grade (1-5), which will be determined following the MedDRA definitions set forth by the International Council for Harmonisation of Technical Requirements for Pharmaceuticals for Human Use (ICH). Use of these standardised guidelines will allow for uniform reporting. The grades are defined as follows:

- Grade 1: Mild AE
- Grade 2: Moderate AE
- Grade 3: Severe AE
- Grade 4: Life-threatening or disabling AE
- Grade 5: Death related to AE

10.2.2 Identifying, managing adverse events

Participants who develop adverse events because of the study interventions or other treatments will be identified at scheduled and unscheduled follow-up visits and referred to the designated hospital for evaluation and treatment according to local guidelines. Mild adverse events will be noted in the participant's case report form; no further action will be taken by study staff except in the case of vomiting, in which case the study medication may need to be re-administered. In the case of any SAE, subjects will be referred to the hospital for management. Transportation to the hospital will be provided where feasible, or transport reimbursed. All participants with SAEs will undergo a record review to identify potential adverse consequences of study participation.

10.2.3 Assessment of causality

The investigator is obligated to assess the relationship between the investigational product and the occurrence of each AE/SAE. The investigator will use clinical judgment to determine the relationship. Alternative causes, such as the natural history of the underlying diseases, concomitant therapy, other risk factors, and the temporal relationship of the event to the investigational product will be considered and investigated. The investigator will also consult the drug information and the DMEC as needed in the determination of his/her assessment.

There may be situations when an SAE has occurred, and the investigator has minimal information to include in the initial report. However, it is very important that the investigator always assess causality for every event prior to transmission of the SAE report. The investigator may change his/her opinion of causality in light of follow-up information, amending the SAE case report form accordingly.

10.2.4 Reporting adverse event procedures

All SAEs will be reported to the in-country principal investigator or an assigned representative within 24 hours of the staff becoming aware of it, using an SAE form, which should be completed and sent electronically. The SAE form asks for the nature of the event, date of onset, severity, corrective therapies given, outcome and causality (i.e. unrelated, unlikely, possible, probably, definitely). The responsible study clinician should assign the causality of the event.

10.2.4.1 Expedited reporting

SAEs that are unexpected and are at least 'possibly related' to the study drug require expedited reporting within 24 hours of the country principal investigator or assigned representative becoming aware of it (e-mail notification); i.e. this will be a maximum of 48 hours after the event occurred or the study team were made aware of the event (including the 24 hours required for the field staff to report to the principal investigator/representative). Additional information will be sent within 14 additional days (full SAE report) if the reaction had not resolved at the time of e-mail notification.

10.2.4.2 Scheduled reporting

Other SAEs and AEs will be reported annually (or more frequently if so required by the DMEC or ethics committees) in an aggregated report. AEs that will not be reported include common illnesses that do not result in hospitalization, including but not limited to clinical malaria, respiratory, gastrointestinal, and skin diseases unless they are considered at least possibly related to the intervention.

10.2.4.3 Recipients of reports

The study will comply with local regulations about reporting of SAEs to their local Research Ethics Committee and/or Research & regulatory offices. In addition to the primary ethics committees, we will report safety data to the DMEC, and the sponsor. In addition, and if so requested by the manufacturer, the sponsor will forward the reports to the manufacturer of DP. A copy of the final

study report will be provided to all study hospitals, ethics committees, TSC, DMEC, to local regulators, and, if so requested, to the manufacturer.

Figure 1: Safety reporting assessment flowchart[82]

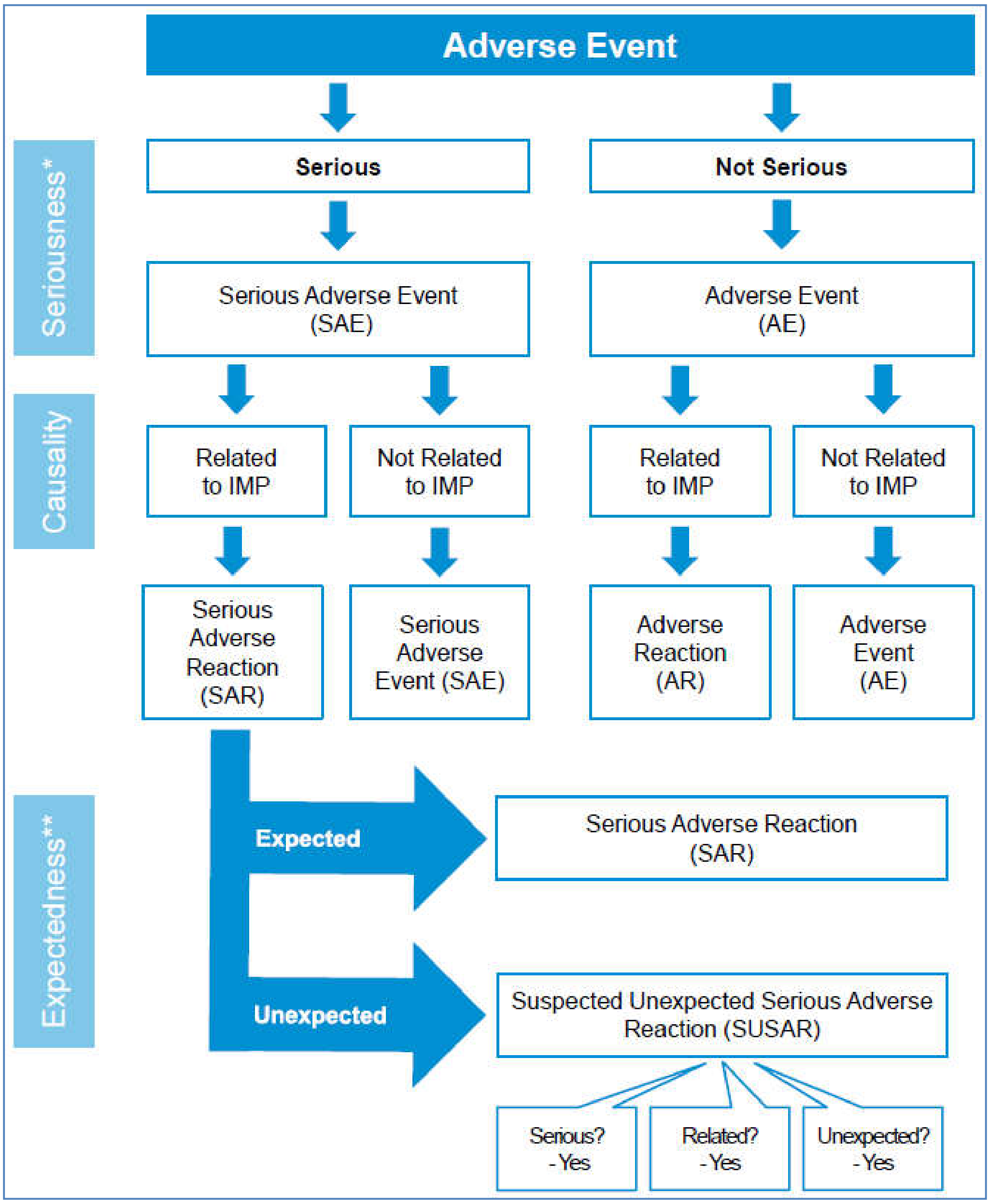

IMP: Investigational Medicinal Product
*See definition of SAE in section 10.2.1
**Assessed in line with the current approved Investigator's Brochure (IB)

10.3 TRIAL MONITORING AND AUDITING

10.3.1 Trial monitoring

External clinical trial monitoring visits are provided by the sponsor at trial initiation, and then regularly (at least yearly) thereafter and at trial closeout, or more frequently if so required; e.g. if the trial fieldwork is about 24 months, this means that each site is visited approximately 4 times by external monitors. The results from each monitoring visit will help inform whether more frequent or earlier

repeat visits are required. The frequency of these visits appeared appropriate for our experienced study sites in recent trials and the feedback was highly appreciated by the study teams.

10.3.2 Auditing

The independent clinical monitoring process will be audited by a study staff from the sponsor's research office at LSTM in Liverpool, UK. The auditor will accompany the clinical monitor during at least one of the site visits. After this visit, it will be determined by the sponsor if more auditing visits are required.

10.3.3 Role of sponsor

The Sponsor reserves the right to suspend temporarily or prematurely discontinue this study at any time for reasons including, but not limited to, safety or ethical issues or severe non-compliance. If the sponsor determines such action is needed, it will discuss this with the investigator. When feasible, the sponsor will provide advance notification to the investigator of the impending action prior to it taking effect. The sponsor will promptly inform the ethics committees and provide the reason for the suspension or termination.

10.4 Other quality control measures

10.4.1 Safety monitors

A local paediatrician and/or obstetrician will act as trial safety monitors, one per country.

10.4.2 Trial monitoring

Each of the partner's institutions have their own internal quality control teams who will conduct internal monitoring regularly, and to help prepare for external monitoring visits. The study will be externally monitored regularly by a monitor appointed by the sponsor's research office at LSTM in Liverpool, UK. The frequency will be at least yearly, but after each visit, the frequency of subsequent monitoring visits will be evaluated by the sponsor to see if more frequent monitoring is required.

10.4.3 Training

The country Principal Investigators are responsible for the conduct of the study at the study sites, including delegation of specified study responsibilities, and training of study staff. Each site will maintain a record of all individuals involved in the study (medical, nursing and other staff) and will ensure that all persons assisting with the trial receive the appropriate training about the protocol, the investigational product(s) and their trial-related duties and functions, including formal certified GCP training. During the study, the regular spot checks will be conducted to assess the performance of the study site staff members and re-training provided where necessary.

10.4.4 Quality assurance/control of laboratory tests

Regular audits of laboratory performance will be completed by experienced supervisors according to standard operating procedures. All malaria blood smears will be read by two different microscopists blinded to the allocation and each other's results, any significantly discordant results based on positive/negative results or difference in parasites above a defined threshold will be verified by a third expert microscopist. All malaria molecular analyses will include a set of positive *P. falciparum* controls and a set of randomly distributed negative controls on the PCR plates. If one or more of the negative controls are PCR positive the PCR run will be discarded. Ten percent of all histopathology slides will undergo external validation.

11 ETHICS AND DISSEMINATION

11.1 DECLARATION OF HELSINKI

The trial will be conducted in compliance with the principles of the Declaration of Helsinki (1996) (See 16.3, Appendix III. Declaration of Helsinki, page 79), the principles of GCP and in accordance with all applicable regulatory requirements in Kenya and Malawi.

11.2 RESEARCH ETHICS AND REGULATORY APPROVAL

11.2.1 Review process

This protocol, the informed consent document, patient information sheets will be reviewed and approved by the Research Ethics Committees at KEMRI, Nairobi, Kenya (SERU), and the College of Medicine in Malawi (COMREC) (or if requested at the national level, by the Malawian National Health Science Research Committee). In addition, the protocol will require approval from the Research and Ethics Committee of Liverpool School of Tropical Medicine, Liverpool (LSTM).

11.2.2 Protocol amendments

If it is necessary for the protocol to be amended, the protocol amendment will be submitted to the research ethics committees at LSTM (sponsor) and the primary ethics committees in each country for approval before implementation in that country. Any change to the informed consent form, except for layout, spelling errors and formatting, must also be approved by the sponsor and the primary ethics committee in each country, before the revised form is used.

No change will be made to the approved protocol without the agreement of the sponsor. The Chief Investigator, or a delegated person, will distribute amendments on behalf of the sponsor to each principal investigator, who in turn is responsible for the distribution of these documents to the staff at his/her study site.

11.2.3 Sanctioning of the protocol by hospitals

Where this is a requirement of participating hospitals, the approved protocol will subsequently be submitted to the participating hospitals' review committees or representatives for subsequent sanctioning of the protocol and procedures by the hospital.

11.2.4 Regulatory approval

Regulatory approval will be sought from the national regulators in Kenya and Malawi from the Pharmacy and Poisons Board; Pharmacy, Medicines and Poisons Board. CTX and DP are currently approved under various brand names in each of the two countries as outlined below:

11.2.4.1 Dihydroartemisinin-piperaquine

Dihydroartemisinin-piperaquine is registered for use as case-management for clinical malaria in both Kenya and Malawi. The main brands registered are DuoCotecxin (from Beijing Holley-Cotec Pharmaceutical Ltd, China), D-ARTEPP® from Guilin Pharma, a GMP and WHO pre-qualified, pharmaceutical company based in China, and Eurartesim®, a GMP manufactured product from AlfaSigma (formerly Sigma-Tau) approved by the European Medicines Agency (EMA), the European regulator for the case-management of malaria.

11.2.4.2 Cotrimoxazole (CTX)

CTX is approved in Kenya and Malawi under various brand names.

11.2.4.3 Fixed dose combinations of cART

The DTG-based formulations are approved as fixed dose combination of TLD (TDF/3TC/DTG) and available under various brands manufactured by Mylan Pharma, Cipla, Hetero labs limited, Aurobindo Pharma, Macleods Pharmaceuticals Limited and Laurus Labs Limited. Of these, by April 2019, TLD manufactured by Mylan, Hetero labs limited, Aurobindo and Macleods and Laurus labs limited are approved or tentatively approved antiretrovirals by the US FDA for use in association with Presidential Emergency Plan for AIDS Relief (PEPFAR).[83]

11.3 Informed consent procedures

Informed consent will be obtained before participants are enrolled in the study. See section 7.5.2.1, and 16.5, Appendix V. Participant information sheets and informed consent statements, page 83 for further details.

11.3.1 Consent procedures

Written, informed consent will be obtained in the local vernacular language. The consent process shall be initiated at the time of enrolment into the study and shall continue throughout the participant's participation. Participants meeting the initial eligibility criteria will have the study explained to them by a member of the study team. If the participant meets the study enrolment criteria, the full consent process will follow, with a written consent form provided. A copy of the informed consent document will be given to the participant for their records unless they state that they do not wish to have a copy.

For illiterate participants, an independent witness will be present during the informed consent process and will sign the consent form as a witness, while the participant will be asked to indicate consent by use of thumbprint. The participant may withdraw consent at any time throughout the course of the study, and this will be made clear in the informed consent process. A copy of the informed consent document will be given to the caregiver for their records unless they state that they do not wish to have a copy.

All individuals will be informed that there is no requirement to join the study and that standard medical care will remain the same regardless of study enrolment. If the participant chooses not to be enrolled in the study, they will be allowed to continue with routine care provided by the health facility without any consequences to the expected level of care provided.

Participants will be given the option to take the patient information sheet and consent statement home to provide them with the opportunity to discuss this with their partner and family members prior to deciding if they want to join the study.

11.4 Protection of privacy and confidentiality

11.4.1 Privacy

Personal and medical information relating to research participants will be treated as confidential. The risk of disclosure will be minimized by secure storage of documents and use of linked data by replacing personal identifiers with a unique study code to conceal the identity of the participant.

11.4.2 Privacy of individual

Individual data such as RDT tests for malaria, syphilis and anaemia will be reported to the participant at point of care, to relevant study staff and where appropriate will be recorded in the participants' medical record book in addition to study CRFs.

11.4.3 Confidentiality of data

All information regarding the participants will remain confidential to the extent allowed by law. Unique numerical identifiers will be used for data entry. All screening forms and case report forms will be kept in a secured location with access limited to authorized study staff. Unique numerical identifiers will be used for the computer-based data entry and blood samples. Publications will contain only aggregate data. No identifying information will be included to ensure individual patient anonymization of all data and results made public.

11.5 Declaration of interest

None of the principal investigators have paid consultancies with the pharmaceutical companies involved in the trial or other competing interest for the overall trial or in each study site.

11.6 Access to source data/documents

In addition to the clinical monitors, authorised representatives of the sponsor/CRO, an IEC/IRB or regulatory authority may visit the study site to perform audits or inspections, including source data verification. The investigator agrees to allow the sponsor and CRO representatives, including the monitor and study safety monitor, the DMEC, the IRB/IEC and regulatory authority direct access to source data and other relevant documents.

11.7 Risks and benefits

11.7.1 Risks to study participants

11.7.1.1 Safety of the study drugs in pregnancy

All three drugs (CTX, DP and TLD) are currently thought to be safe for the mother and foetus during the second and third trimesters of pregnancy and have been used in research settings with favourable safety profiles within the proposed doses to be used in the trial. For more details of the safety of the study drugs in pregnancy and drug-drug interactions see 3.4, Safety of study drugs in pregnancy, page 23 and section 3.3, Drug-drug interactions, page 23.

All adverse events, including those associated with the study medication, will be recorded and monitored throughout the trial. The trial may be stopped or temporarily suspended by the sponsor at any stage due to any arising safety concerns.

11.7.1.2 Blood and tissue sampling

All examinations undertaken as part of this study will be non-invasive, except for blood sampling. Wherever possible, blood sampling will be minimised. Blood samples of no more than 12 mL will be taken before enrolment, for eligibility screening/study-specific baseline testing. The sample will be taken at the screening stage. A similar blood sample shall be collected at all scheduled visits, and aliquoted, where necessary, into respective sample tubes for study investigations. An additional 2 ml venous blood sample is taken from participants participating in the cardiac safety study on day 2, 4-6 hours after administration of the 3rd intervention dose, and for two more times during pregnancy, 4-6 hours after a selected monthly course of DP.

A maternal blood sample (no more than 12 mL) will be taken at scheduled visits and at birth, and where possible routine care and study-specific samples will be combined in a single blood draw.

Blood sampling may be inconvenient to the participants and may cause minor discomfort and bruising. In some aspects of the trial, blood sampling has the potential to directly benefit the participants or their babies, as any malaria infection or anaemia detected because of the sampling will be treated. In other aspects of the trial, such as parasite genetics, CTX (or antifolate) and piperaquine resistance markers will not be of direct benefit to the individual woman, but the potential knowledge gained will eventually be used to benefit all pregnant participants and their babies in areas with similar characteristics in sub-Saharan Africa.

The volume of blood collected from each participant will be small, a maximum of 100 ml per woman over the course of the study. Only well-trained nursing and laboratory staff will be employed on the trial. Only new disposable needles and lancets will be used for blood sampling procedures, and these will be safely discarded immediately after their use. There no direct risks to the participants while collecting cord blood and placental samples. In the event of a needle stick to study staff during blood and tissue sampling, HIV post exposure prophylaxis (PEP) will be provided at the trial sites at the cost of the trial.

11.7.2 Benefits to study participants

By taking part in this trial, participants will receive either placebo-DP or active DP in addition to their daily CTX and cART. All these regimens have either been the routine standard of care (daily CTX) or have the potential to have significantly increased effectiveness on birth outcome (DP). Participants who do not attend scheduled appointments will receive reminders and active follow-up. Participants experiencing illness between visits will be seen and treated free of charge as part of the study though this is already routine practice in some of the participating countries, such as in Malawi. As a minimum, the participant will benefit from frequent reminders to attend the monthly ANC schedule, reduced waiting times, and ensured availability of study drugs and routine care. Where participants are diagnosed with malaria or an STI (e.g. syphilis) during scheduled and unscheduled visits, treatment will be provided as per the nationally recommended standard of care. This will be recorded for participants who received treatment during follow up.

11.7.3 Risk to the population

11.7.3.1 DP resistance

Monthly DP will also exert some drug pressure on piperaquine. Artemisinin-based combination therapies, including DP, have demonstrable parasite resistance in South East Asia and there is concern that resistance will spread to or develop within Africa.[84] Importantly we will monitor for signs that drug pressure is selecting for resistant parasite strains as outlined in section. We will determine if any mutations within these known drug resistance candidates increase because of exposure to either daily CTX (antifolate resistance) or DP (piperaquine) or the artemisinin(dihydro-artemisinin).

11.7.4 Other ethical considerations

11.7.4.1 Inclusion of young people under the age of 18

This study will include young participants aged <18y. Young pregnant participants, in tandem with the respective country laws of age of emancipation, will be considered emancipated, therefore being legally able to consent on their own behalf, and to be included in a clinical trial. It is important to include young participants in the trial, as adolescents are known to be particularly susceptible to malaria in pregnancy and are therefore one of the groups that may benefit the most from any improvements to practice to prevent adverse outcomes related to malaria in pregnancy.

11.7.4.2 Long term samples storage for future studies and shipment of samples to external laboratories

With respect to applicable country regulations, written informed consent will be sought from participants participating in the trial for long term storage of their samples for future research. Informed consent shall be sought from all participants for the shipping of their samples to external laboratories for relevant analyses.

11.8 ANCILLARY AND POST-TRIAL CARE

11.8.1 Health care during the trial

All care directly related to the proper and safe conduct of the trial, and the treatment of immediate adverse events related to trial procedures will be provided free of charge by the study in the study hospitals. The provision of ancillary care beyond that immediately required for the conduct of the trial will not be covered by the trial.

11.8.2 Trial insurance

The sponsor will take out trial insurance such that participants enrolled in the study are covered by indemnity for negligent harm and non-negligent harm associated with the protocol. This will include cover for additional health care, compensation or damages whether awarded voluntarily by the Sponsor, or by claims pursued through the courts. The liability of the manufacturer of the trial drug DP is limited to those claims arising from faulty manufacturing of the commercial product and not to any aspects of the conduct of the study.

11.8.3 Post-trial care

The study budget is not able to fund post-study care or implementation of monthly DP as policy. However, the investigators work in close collaboration with local and international policymakers (e.g. WHO) and funders (e.g. President's Emergency Plan for AIDS Relief [PEPFAR], President’s Malaria Initiative [PMI]) to ensure those policymakers and funders are informed early of germane research findings.

11.9 EXPENSES REIMBURSEMENT AND INCENTIVES

The study will provide payment for all study drugs, study procedures, study-related visits and reasonable medical expenses that are incurred in study clinics or hospitals because of the study, including expenses for transport for any study related visits including unscheduled visits in between scheduled visits to study clinics. The study will not cover the costs of any non-malaria or non-study related events, including scheduled or unscheduled surgery or trauma-related events (e.g. accidents, burns etc.) if this is not deemed to be related to the study by the principal investigators or their representative.

Table 4: Reimbursement of expenses and incentives provided by the study

To Who	What	Approximate Amount
Hospital	Improvement of infrastructure where required	up to ~$10,000/hospital
	Training of routine staff adult ward	~ $3,000/hospital
	Study procedure costs and study drugs and admission fees for inpatients	~ $100,000/hospital

Participant (mother or infant)	Travel expenses for the participant	Up to ~$10 per round trip*
	Compensation for each day they are scheduled to come to the ANC, out-patient clinic or delivery unit and need to stay or wait for more than 4 hours	Up to ~$ 3 per day (excluding any travel expenses)

*In exceptional cases, higher amounts of travel expenses can be reimbursed if distance requires. This would need to be decided on a case by case basis, courtesy of the site PI/coordinator.

11.10 Dissemination and application of the results

11.10.1 Result dissemination and publication policy

11.10.1.1 Dissemination to local stakeholders, global policy makers, academic beneficiaries and the general public

Our research findings will be communicated to country level stakeholders including the study participants, reproductive health and malaria programmes/national Ministries of Health, and the research partner institutions in Kenya and Malawi, and to other national and international research partners, NGOs, technical agencies and implementing partners, donor organizations, WHO, UNICEF and the general public. We will use multiple communication strategies to reach each target audience as outlined below.

11.10.1.2 Local Stakeholders

Communications with stakeholders involved in the trials and nested studies (i.e. study participants, health providers and trial staff), Ministries of Health and their technical and implementing partners, and donors in Kenya and Malawi will be through face-to-face meetings and local forum presentations at village, district, regional, county and national levels as may be applicable. The results of the trial and nested studies will be communicated through a dissemination meeting to all national level stakeholders held in one of the two trial countries, preferably linking to the national interagency and multidisciplinary technical committees on malaria, reproductive health, and HIV/AIDS (see 11.10.2, Impact, page 61). Policy briefs describing the results in clear, jargon-free language targeting country policy makers will be disseminated to facilitate policy adoption and subsequent implementation, as appropriate.

11.10.1.3 Academic beneficiaries

Communication of trial results to academic beneficiaries will be achieved through standard academic channels including open-access, peer-reviewed journal publications, articles in science review magazines such as International Innovation, symposia, conferences, and the dissemination workshop to multidisciplinary malaria technical committees (which include academics) in Malawi and Kenya. The full de-identified individual patient level dataset from the trial will be made publicly accessible via the WWARN platform (http://www.wwarn.org/working-together/sharing-data/accessing-data). The trial publications will also be disseminated via institutional websites of partners, and to all partners of the LSTM-led Malaria in Pregnancy Consortium by e-mail, their website, and the MiP library, which is the most comprehensive and reliable database of literature on malaria in pregnancy currently available (http://library.mip-consortium.org/). The research will also be disseminated to all academics within each of the participating institutions through monthly and annual dissemination seminars that are held routinely for information sharing and to encourage networking, cross disciplinary working, and development of new initiatives. The Chief Investigator will be responsible for ensuring dissemination in the UK, Drs Meghna Desai and Julie Gutman for dissemination in the US, and the country PIs (Kenya:

Hellen Barsosio and Simon Kariuki), (Malawi: Mwayiwawo Madanitsa, supported by Victor Mwapasa, Ken Maleta) for dissemination in Kenya and Malawi, respectively.

11.10.1.4 WHO and Roll Back Malaria (RBM) Partnership

Results of the trial are expected to be presented to WHO at a WHO-convened ERG meeting in 2020 or 2021 for consideration for policy. If the intervention is recommended for global policy, the research team will support WHO to prepare and disseminate policy guidance information aimed at policy users in sub-Saharan Africa. The results will also be disseminated to the Roll Back Malaria (RBM) Partnership MiP Working Group, responsible for generating consensus among RBM Partners, including national malaria and reproductive health programmes, technical agencies and donors, on key strategic issues and best practices for ensuring effective delivery and scale-up of interventions for the prevention and control of MiP.

11.10.1.5 General Public

Communication to a broader audience and the public will be via press releases and TV opportunities in the news media in the UK, Kenya and Malawi, news items on websites including partner websites and online networks such as Global Health Network https://globalhealthtrials.tghn.org, and publication portals including the MiP library.

11.10.2 Impact

11.10.2.1 Likelihood to result in major advances for the field of malaria control in pregnancy

The context for the trial is highly significant and timely. Several multi-centre trials for the control of malaria in pregnancy have been completed in the last decade. However, very few studies have looked at malaria prophylaxis for HIV-infected pregnant women. It is thought that antifolate cross-resistance with IPTp-SP and CTX may have a negative impact on daily CTX for malaria prophylaxis in this population, suggested by the recent trials that showed that adding an effective antimalarial (mefloquine) markedly improved the chemoprevention of malaria compared to daily CTX alone.[80] However, mefloquine was not well tolerated and cannot be considered as IPTp for chemoprevention in pregnancy. The results of the recently completed exploratory trials of IPTp with DP in Kenya and Uganda in HIV-negative women[29,30]are promising and DP has now emerged as the leading, and so far only, suitable candidate to replace SP for use in IPTp in HIV-negative women or in addition to daily CTX in HIV-positive women.[85]

11.10.2.2 Improving public health, economic and societal impacts

The ultimate beneficiaries of this research will be mothers and their infants in Kenya and Malawi, and other countries experiencing high levels of antifolate resistance, whose quality of life, health, and creative output will have been enhanced. Malaria poses a substantial economic and social burden in endemic countries, with costs arising at the household level through direct costs of seeking care and indirect (productivity) losses; and at the aggregate level by reducing economic growth. Malaria in pregnancy is estimated to be responsible for 35% of preventable low birthweight, which is a strong predictor of infant mortality, childhood morbidity and productivity in adult life.[13] Identifying more effective means of preventing malaria in pregnancy for these participants can reduce part of this burden and enhance the effectiveness of public investments in malaria control.

Approximately 10 to 19% of the pregnancies in East and Southern Africa occur among HIV-infected women. We estimate that each year approximately 620,000 to 1 million HIV-infected pregnancies will benefit from a switch to the new intervention if found to be superior to daily CTX. These are the pregnancies among HIV-infected women that occur in highly antifolate resistant areas in east and southern Africa where the quintuple dhfr/dhps haplotype has reached saturation, representing

approximately 24% of all pregnancies in HIV-infected women in malaria-endemic Africa. About one-third of these occur in settings with appreciable levels of the sextuple haplotype which severely compromises the effectiveness of antifolate drugs such as SP (Patrick Walker, Imperial College, unpublished observations).

11.10.2.3 Contribution to strengthening the capacity in sub-Saharan Africa to conduct clinical trials

The development of research capacity of African institutions is central to the achievement of the research objectives and includes training in trial methodologies, including in Good Clinical Practice, and strengthening of the quality assurance/control procedures. The multi-centre trial in both countries will enhance the visibility of the two African research institutions and the potential to improve research quality and the rate at which pivotal research findings can be translated into policy, thereby increasing its impact. The trial will furthermore result in the training of a cohort of one Malawian Post-Doc and two African PhD students, contributing skilled research staff to the national workforce. Health worker capacity in the feasibility study sites in Kenya will also be strengthened through training, thereby advancing knowledge, improving skills and increasing the effectiveness of public health services for pregnant participants.

11.10.3 Authorship and publications

Potential authors include all professionals that have participated in the trial for a minimum of six months. Authorship of any presentations or publications arising from this study will also be governed by the principles for authorship criteria of the International Committee of Medical Journal Editors has designed.[86] Disputes regarding authorship will be settled by the publications committee, with further involvement of the independent chair of the TSC if so required. The manufacturers of the study medications will be provided with a draft of the manuscript but will have no role in review, data interpretation, or writing of the article.

11.10.4 Data Sharing Statement

Biological samples and data will be shared using material and data transfer agreements with the collaborating institutions (see 2.2.4 Collaborators, page 9) to minimise the risk of unauthorised analysis beyond the scope of the agreed parameters.

The full protocol will be available on request to any interested professional and may be published in a peer reviewed journal or deposited in an online repository. Individual, de-identified participant data will be made available for meta-analyses as soon as the data analysis is completed, with the understanding that results of the meta-analysis will not be published prior to the results of the individual trial without prior agreement of the investigators. No later than five years after the publication of the trial a fully de-identified data set of the complete patient-level data will be available for sharing purposes, such as via the WWARN repository platform (http://www.wwarn.org/working-together/sharing-data/accessing-data). All requests for data for secondary analysis will be considered by a Data Access Committee to ensure that use of data is within the terms of consent and ethics approval.

12 Timeframe and duration of the study

12.1 Timeline

The total duration of the project is 48 months, including 36 months for the preparation and conduct of the trial, and 1 additional year for the PhD students to write up their theses. The 36 months trial period includes 12 months for trial preparation and 19 months field work (12 months recruitment plus 7

months of mother-infant follow-up until the child is 6 weeks old), and 5 months for completion of laboratory assays, data analysis and reporting. The 12 months for trial preparation are needed for ethical and regulatory clearances, trial insurance, trial registration, study drug shipment and packing, establishment of a DMEC, development of case-record forms (CRFs) and standard operating procedures (SOPs), and community sensitisation and staff training. The study will include 1 interim analysis of efficacy and safety data when approximately 75% of the patients have been enrolled.

12.1.1 Project Gantt chart

	Year 1				Year 2				Year 3				Year 4*			
	Q 1	Q 2	Q 3	Q 4	Q 1	Q 2	Q 3	Q 4	Q 1	Q 2	Q 3	Q 4	Q 1	Q 2	Q 3	Q 4
Activity																
Protocol development	■															
IRB approval / Annual Review	■	■	■	■			■				■					
Regulatory approval/ Annual Review		■	■	■			■				■					
CRF and SOP development		■	■	■												
Drug and placebo preparation		■	■													
Drug shipment			■	■												
Enrolment					■	■	■	■								
Study					■	■	■	■ .	■	■	■					
Closure												■				
Analysis												■	■	■	■	
Manuscript and results dissemination												.	■	■	■	■

13 References

1. Desai M, ter Kuile FO, Nosten F, McGready R, Asamoa K, Brabin B, Newman RD, 2007. Epidemiology and burden of malaria in pregnancy. Lancet Infect Dis 7: 93-104.
2. Gonzalez R, Sevene E, Jagoe G, Slutsker L, Menendez C, 2016. A Public Health Paradox: The Women Most Vulnerable to Malaria Are the Least Protected. PLoS Med 13: e1002014.
3. UNAIDS/WHO, 2013. Global report: UNAIDS report on the global AIDS Epidemic 2013. WHO Library Cataloguing-in-Publication Data 2013;UNAIDS(JC2502/1/E).
4. World Health Organization, 2017. World malaria report 2016. Geneva: World Health Organization.
5. Naing C, Sandhu NK, Wai VN, 2016. The Effect of Malaria and HIV Co-Infection on Anemia: A Meta-Analysis. Medicine (Baltimore) 95: e3205.

6. ter Kuile FO, Parise ME, Verhoeff FH, Udhayakumar V, Newman RD, van Eijk AM, Rogerson SJ, Steketee RW, 2004. The burden of co-infection with human immunodeficiency virus type 1 and malaria in pregnant women in sub-saharan Africa. Am J Trop Med Hyg 71: 41-54.
7. Gonzalez R, Ataide R, Naniche D, Menendez C, Mayor A, 2012. HIV and malaria interactions: where do we stand? Expert Rev Anti Infect Ther 10: 153-65.
8. Xiao PL, Zhou YB, Chen Y, Yang MX, Song XX, Shi Y, Jiang QW, 2015. Association between maternal HIV infection and low birth weight and prematurity: a meta-analysis of cohort studies. BMC Pregnancy Childbirth 15: 246.
9. Bailey PE, Keyes E, Moran AC, Singh K, Chavane L, Chilundo B, 2015. The triple threat of pregnancy, HIV infection and malaria: reported causes of maternal mortality in two nationwide health facility assessments in Mozambique, 2007 and 2012. BMC Pregnancy Childbirth 15: 293.
10. Menendez C, Ferenchick E, Roman E, Bardaji A, Mangiaterra V, 2015. Malaria in pregnancy: challenges for control and the need for urgent action. Lancet Glob Health 3: e433-e434.
11. World Health Organization, 2017. Intermittent preventive treatment in pregnancy (IPTp). Available at: http://www.who.int/malaria/areas/preventive_therapies/pregnancy/en/. Accessed 05 November 2017, 2017.
12. Weetman D, Kamgang B, Badolo A, Moyes CL, Shearer FM, Coulibaly M, Pinto J, Lambrechts L, McCall PJ, 2018. Aedes Mosquitoes and Aedes-Borne Arboviruses in Africa: Current and Future Threats. International journal of environmental research and public health 15: 220.
13. Charlier C, Beaudoin M-C, Couderc T, Lortholary O, Lecuit M, 2017. Arboviruses and pregnancy: maternal, fetal, and neonatal effects. The Lancet Child & Adolescent Health 1: 134-146.
14. C. King C, R. Ellington S, P. Kourtis A, 2013. The Role of Co-Infections in Mother-to-Child Transmission of HIV. Current HIV Research 11: 10-23.
15. McClure EM, Goldenberg RL, 2009. Infection and stillbirth. Semin Fetal Neonatal Med 14: 182-9.
16. Kapito-Tembo A, Meshnick SR, van Hensbroek MB, Phiri K, Fitzgerald M, Mwapasa V, 2011. Marked reduction in prevalence of malaria parasitemia and anemia in HIV-infected pregnant women taking cotrimoxazole with or without sulfadoxine-pyrimethamine intermittent preventive therapy during pregnancy in Malawi. J Infect Dis 203: 464-72.
17. Manyando C, Njunju EM, D'Alessandro U, 2013. Safety and efficacy of co-trimoxazole for treatment and prevention of Plasmodium falciparum malaria: a systematic review. PLos one 8: e56916.
18. Manyando C, Njunju EM, D'Alessandro U, Van Geertruyden JP, 2013. Safety and efficacy of co-trimoxazole for treatment and prevention of Plasmodium falciparum malaria: a systematic review. PLoS One 8: e56916.
19. Sridaran S, McClintock SK, Syphard LM, Herman KM, Barnwell JW, Udhayakumar V, 2010. Anti-folate drug resistance in Africa: meta-analysis of reported dihydrofolate reductase (dhfr) and dihydropteroate synthase (dhps) mutant genotype frequencies in African Plasmodium falciparum parasite populations. Malar J 9: 247.
20. White NJ, 2004. Antimalarial drug resistance. J Clin Invest 113: 1084-92.
21. Harrington WE, Mutabingwa TK, Kabyemela E, Fried M, Duffy PE, 2011. Intermittent treatment to prevent pregnancy malaria does not confer benefit in an area of widespread drug resistance. Clin Infect Dis 53: 224-30.
22. Green M, Otieno K, Katana A, Slutsker L, Kariuki S, Ouma P, Gonzalez R, Menendez C, ter Kuile F, Desai M, 2016. Pharmacokinetics of mefloquine and its effect on sulfamethoxazole and trimethoprim steady-state blood levels in intermittent preventive treatment (IPTp) of pregnant HIV-infected women in Kenya. Malar J 15: 7.

23. González R, Pons-Duran C, Piqueras M, Aponte JJ, ter Kuile FO, Menéndez C, 2018. Mefloquine for preventing malaria in pregnant women. The Cochrane Database of Systematic Reviews: CD011444.
24. Ter Kuile FO, Taylor SM, 2017. Gilding the Lily? Enhancing Antenatal Malaria Prevention in HIV-Infected Women. J Infect Dis 216: 4-6.
25. Desai M, Hill J, Fernandes S, Walker P, Pell C, Gutman J, Kayentao K, Gonzalez R, Webster J, Greenwood B, 2018. Prevention of malaria in pregnancy. The Lancet Infectious Diseases.
26. Gonzalez R, Mombo-Ngoma G, Ouedraogo S, Kakolwa MA, Abdulla S, Accrombessi M, Aponte JJ, Akerey-Diop D, Basra A, Briand V, Capan M, Cot M, Kabanywanyi AM, Kleine C, Kremsner PG, Macete E, Mackanga JR, Massougbodgi A, Mayor A, Nhacolo A, Pahlavan G, Ramharter M, Ruperez M, Sevene E, Vala A, Zoleko-Manego R, Menendez C, 2014. Intermittent preventive treatment of malaria in pregnancy with mefloquine in HIV-negative women: a multicentre randomized controlled trial. PLoS Med 11: e1001733.
27. Kimani J, Phiri K, Kamiza S, Duparc S, Ayoub A, Rojo R, Robbins J, Orrico R, Vandenbroucke P, 2016. Efficacy and Safety of Azithromycin-Chloroquine versus Sulfadoxine-Pyrimethamine for Intermittent Preventive Treatment of Plasmodium falciparum Malaria Infection in Pregnant Women in Africa: An Open-Label, Randomized Trial. PLoS One 11: e0157045.
28. Clerk CA, Bruce J, Affipunguh PK, Mensah N, Hodgson A, Greenwood B, Chandramohan D, 2008. A randomized, controlled trial of intermittent preventive treatment with sulfadoxine-pyrimethamine, amodiaquine, or the combination in pregnant women in Ghana. J Infect Dis 198: 1202-11.
29. Kakuru A, Jagannathan P, Muhindo MK, Natureeba P, Awori P, Nakalembe M, Opira B, Olwoch P, Ategeka J, Nayebare P, Clark TD, Feeney ME, Charlebois ED, Rizzuto G, Muehlenbachs A, Havlir DV, Kamya MR, Dorsey G, 2016. Dihydroartemisinin-Piperaquine for the Prevention of Malaria in Pregnancy. N Engl J Med 374: 928-39.
30. Desai M, Gutman J, L'Lanziva A, Otieno K, Juma E, Kariuki S, Ouma P, Were V, Laserson K, Katana A, Williamson J, Ter Kuile FO, 2015. Intermittent screening and treatment or intermittent preventive treatment with dihydroartemisinin-piperaquine versus intermittent preventive treatment with sulfadoxine-pyrimethamine for the control of malaria during pregnancy in western Kenya: an open-label, three-group, randomised controlled superiority trial. Lancet.
31. Tarning J, 2016. Treatment of Malaria in Pregnancy. N Engl J Med 374: 981-2.
32. Desai M, Hill J, Fernandes S, Walker P, Pell C, Gutman J, Kayentao K, Gonzalez R, Webster J, Greenwood B, Cot M, ter Kuile FO, 2018 (in press). Prevention of malaria in pregnancy. Lancet Infect Dis.
33. Natureeba P, Kakuru A, Muhindo M, Ochieng T, Ategeka J, Koss CA, Plenty A, Charlebois ED, Clark TD, Nzarubara B, Nakalembe M, Cohan D, Rizzuto G, Muehlenbachs A, Ruel T, Jagannathan P, Havlir DV, Kamya MR, Dorsey G, 2017. Intermittent Preventive Treatment With Dihydroartemisinin-Piperaquine for the Prevention of Malaria Among HIV-Infected Pregnant Women. J Infect Dis 216: 29-35.
34. Muhindo MK, Kakuru A, Natureeba P, Awori P, Olwoch P, Ategeka J, Nayebare P, Clark TD, Muehlenbachs A, Roh M, Mpeka B, Greenhouse B, Havlir DV, Kamya MR, Dorsey G, Jagannathan P, 2016. Reductions in malaria in pregnancy and adverse birth outcomes following indoor residual spraying of insecticide in Uganda. Malar J 15: 437.
35. Kajubi R, Huang L, Jagannathan P, Chamankhah N, Were M, Ruel T, Koss CA, Kakuru A, Mwebaza N, Kamya M, Havlir D, Dorsey G, Rosenthal PJ, Aweeka FT, 2017. Antiretroviral Therapy With Efavirenz Accentuates Pregnancy-Associated Reduction of Dihydroartemisinin-Piperaquine Exposure During Malaria Chemoprevention. Clin Pharmacol Ther 102: 520-528.
36. Khan M, Santhosh SR, Tiwari M, Lakshmana Rao PV, Parida M, 2010. Assessment of in vitro prophylactic and therapeutic efficacy of chloroquine against Chikungunya virus in vero cells. J Med Virol 82: 817-24.

37. Shiryaev SA, Mesci P, Pinto A, Fernandes I, Sheets N, Shresta S, Farhy C, Huang C-T, Strongin AY, Muotri AR, Terskikh AV, 2017. Repurposing of the anti-malaria drug chloroquine for Zika Virus treatment and prophylaxis. Scientific Reports 7: 15771.
38. Efferth T, Romero MR, Wolf DG, Stamminger T, Marin JJ, Marschall M, 2008. The antiviral activities of artemisinin and artesunate. Clin Infect Dis 47: 804-11.
39. Ho WE, Peh HY, Chan TK, Wong WS, 2014. Artemisinins: pharmacological actions beyond anti-malarial. Pharmacol Ther 142: 126-39.
40. World Health Organization, 2017. Transition to New Antiretrovirals in HIV Programmes. Geneva.
41. UNAIDS, 2015. Press release. Three new agreements announced with the potential to expand access to innovative HIV treatment in low- and middle-income countries. 30 November 2015. http://www.unaids.org/en/resources/presscentre/pressreleaseandstatementarchive/2015/november/20151130_PR_CHAI_UNITAID. Accessed.
42. Kanters S, Vitoria M, Doherty M, Socias ME, Ford N, Forrest JI, Popoff E, Bansback N, Nsanzimana S, Thorlund K, Mills EJ, 2016. Comparative efficacy and safety of first-line antiretroviral therapy for the treatment of HIV infection: a systematic review and network meta-analysis. Lancet HIV 3: e510-e520.
43. World Health Organisation, 2018. Updated recommendations on first-line and second-line antiretroviral regimens and post-exposure prophylaxis and recommendations on early infant diagnosis of HIV: Interim guidelines. Supplement to the 2016 consolidated guidelines on the use of antiretroviral drugs for treating and preventing HIV infection. December 2018. World Health Organisation, ed. HIV treatment. Geneva: World Health Organisation.
44. Khoo S, 2018. Dolutegravir in Pregnant HIV Mothers and Their Neonates (DolPHIN-2); https://clinicaltrials.gov/ct2/show/NCT03249181. Available at: https://clinicaltrials.gov/ct2/show/NCT03249181. Accessed.
45. Kintu K, Malaba T, Nakibuka J, Papamichael C, Colbers A, Seden K, Watson V, Reynolds H, Wang D, Waitt C, Orrell C, Lamorde M, Myer L, Khoo S, 2019. DolPHIN-2 : RCT of Dolutegravir vs Efavirenz-Based Therapy Initiated In Late Pregnancy. The annual Conference on Retroviruses and Opportunistic Infections (CROI 2019). March 4 to March 7, 2019. Seattle, Washington state, USA.
46. WHO, 2013. First-line ART for pregnant and breastfeeding women and ARV drugs for their infants. HIV/AIDS. Geneva: World Health Organization.
47. World Health Organisation, 2013. First-line ART for pregnant and breastfeeding women and ARV drugs for their infants. HIV/AIDS. Geneva: World Health Organization.
48. Ministry of Health Malawi, 2011. Clinical Management of HIV in Children and Adults.
49. Ministry of Health Kenya, 2012. Guidelines for the Prevention of Mother to Child Transmission (PMTCT) of HIV/AIDS in Kenya. 4th Edition. Nairobi: NATIONAL AIDS & STI CONTROL PROGRAMME.
50. Cottrell ML, Hadzic T, Kashuba AD, 2013. Clinical pharmacokinetic, pharmacodynamic and drug-interaction profile of the integrase inhibitor dolutegravir. Clin Pharmacokinet 52: 981-94.
51. Walimbwa SI, Lamorde M, Waitt C, Kaboggoza J, Else L, Byakika-Kibwika P, Amara A, Gini J, Winterberg M, Chiong J, Tarning J, Khoo SH, 2019. Drug Interactions between Dolutegravir and Artemether-Lumefantrine or Artesunate-Amodiaquine. Antimicrob Agents Chemother 63.
52. van Lunzen J, Maggiolo F, Arribas JR, Rakhmanova A, Yeni P, Young B, Rockstroh JK, Almond S, Song I, Brothers C, Min S, 2012. Once daily dolutegravir (S/GSK1349572) in combination therapy in antiretroviral-naive adults with HIV: planned interim 48 week results from SPRING-1, a dose-ranging, randomised, phase 2b trial. Lancet Infect Dis 12: 111-8.

53. Group PS, Pekyi D, Ampromfi AA, Tinto H, Traore-Coulibaly M, Tahita MC, Valea I, Mwapasa V, Kalilani-Phiri L, Kalanda G, Madanitsa M, Ravinetto R, Mutabingwa T, Gbekor P, Tagbor H, Antwi G, Menten J, De Crop M, Claeys Y, Schurmans C, Van Overmeir C, Thriemer K, Van Geertruyden JP, D'Alessandro U, Nambozi M, Mulenga M, Hachizovu S, Kabuya JB, Mulenga J, 2016. Four Artemisinin-Based Treatments in African Pregnant Women with Malaria. N Engl J Med 374: 913-27.
54. Benjamin JM, Moore BR, Salman S, Page-Sharp M, Tawat S, Yadi G, Lorry L, Siba PM, Batty KT, Robinson LJ, Mueller I, Davis TM, 2015. Population pharmacokinetics, tolerability, and safety of dihydroartemisinin-piperaquine and sulfadoxine-pyrimethamine-piperaquine in pregnant and nonpregnant Papua New Guinean women. Antimicrob Agents Chemother 59: 4260-71.
55. Moore BR, Salman S, Benjamin J, Page-Sharp M, Yadi G, Batty KT, Siba PM, Mueller I, Davis TM, 2015. Pharmacokinetics of piperaquine transfer into the breast milk of Melanesian mothers. Antimicrob Agents Chemother 59: 4272-8.
56. Moore KA, Simpson JA, Paw MK, Pimanpanarak M, Wiladphaingern J, Rijken MJ, Jittamala P, White NJ, Fowkes FJ, Nosten F, McGready R, 2016. Safety of artemisinins in first trimester of prospectively followed pregnancies: an observational study. Lancet Infect Dis.
57. Tarning J, Rijken MJ, McGready R, Phyo AP, Hanpithakpong W, Day NP, White NJ, Nosten F, Lindegardh N, 2012. Population pharmacokinetics of dihydroartemisinin and piperaquine in pregnant and nonpregnant women with uncomplicated malaria. Antimicrob Agents Chemother 56: 1997-2007.
58. Rijken MJ, McGready R, Phyo AP, Lindegardh N, Tarning J, Laochan N, Than HH, Mu O, Win AK, Singhasivanon P, White N, Nosten F, 2011. Pharmacokinetics of dihydroartemisinin and piperaquine in pregnant and nonpregnant women with uncomplicated falciparum malaria. Antimicrob Agents Chemother 55: 5500-6.
59. Rijken MJ, McGready R, Boel ME, Barends M, Proux S, Pimanpanarak M, Singhasivanon P, Nosten F, 2008. Dihydroartemisinin-piperaquine rescue treatment of multidrug-resistant Plasmodium falciparum malaria in pregnancy: a preliminary report. Am J Trop Med Hyg 78: 543-5.
60. Kajubi R, Ochieng T, Kakuru A, Jagannathan P, Nakalembe M, Ruel T, Opira B, Ochokoru H, Ategeka J, Nayebare P, Clark TD, Havlir DV, Kamya MR, Dorsey G, 2019. Monthly sulfadoxine-pyrimethamine versus dihydroartemisinin-piperaquine for intermittent preventive treatment of malaria in pregnancy: a double-blind, randomised, controlled, superiority trial. Lancet.
61. Ter Kuile FO, Ahmed R, Syafruddin D, 2013. ISRCTN34010937 DOI 10.1186/ISRCTN34010937 STOPMiP: Intermittent Screening and Treatment Or intermittent Preventive therapy for the control of Malaria in Pregnancy in Indonesia (http://www.isrctn.com/ISRCTN34010937). Accessed 2013.
62. Natureeba P, Kakuru A, Muhindo M, Ochieng T, Ategeka J, Koss CA, Plenty A, Charlebois ED, Clark TD, Nzarubara B, 2017. Intermittent preventive treatment with dihydroartemisinin-piperaquine for the prevention of malaria among HIV-infected pregnant women. The Journal of Infectious Diseases: jix110.
63. Mytton OT, Ashley EA, Peto L, Price RN, La Y, Hae R, Singhasivanon P, White NJ, Nosten F, 2007. Electrocardiographic safety evaluation of dihydroartemisinin piperaquine in the treatment of uncomplicated falciparum malaria. Am J Trop Med Hyg 77: 447-50.
64. Keating G, 2012. Dihydroartemisinin/Piperaquine: A Review of its Use in the Treatment of Uncomplicated Plasmodium falciparum Malaria. Drugs 72: 937-961.
65. Ter Kuile FO, 2013. ISRCTN34010937 DOI 10.1186/ISRCTN34010937 STOPMiP: Intermittent Screening and Treatment Or intermittent Preventive therapy for the control of Malaria in Pregnancy in Indonesia (http://www.isrctn.com/ISRCTN34010937). Accessed 2013.

66. Bigira V, Kapisi J, Clark TD, Kinara S, Mwangwa F, Muhindo MK, Osterbauer B, Aweeka FT, Huang L, Achan J, Havlir DV, Rosenthal PJ, Kamya MR, Dorsey G, 2014. Protective efficacy and safety of three antimalarial regimens for the prevention of malaria in young ugandan children: a randomized controlled trial. PLoS Med 11: e1001689.
67. Natureeba P, Kakuru A, Muhindo M, Ochieng T, Ategeka J, Koss CA, Plenty A, Charlebois E, Clark TD, Nzarubara B, Nakalembe M, Cohan D, Rizzuto G, Muehlenbachs A, Ruel T, Jagannathan P, Havlir D, Kamya M, Dorsey G, 2017. Intermittent Preventive Treatment with Dihydroartemisinin-piperaquine for the Prevention of Malaria among HIV-infected Pregnant Women. Journal of Infectious Diseases.
68. World Health Organization, 2017. WHO malaria policy advisory committee meeting: meeting report, October 2017.
69. ISGlobal, 2018. Improving Maternal and Infant Health by reducing malaria risks in African women: evaluation of the safety and efficacy of dihydroartemisinin-piperaquine for intermittent preventive treatment of malaria in HIV-infected pregnant women (MAMAH). Available at: https://www.isglobal.org/en/project/-/asset_publisher/qf6QOKuKkIC3/content/mamah. Accessed 07 May 2018, 2018.
70. Erika Wallender KV, Prasanna Jagannathan, Liusheng Huang, Paul Natureeba, Abel Kakura, Mary Muhindo, Mirium Nakalembe, Diane Havlir, Moses Kamya, Francesca Aweeka, Grant Dorsey, Philip J. Rosenthal, Radojka M. Savic, 2017. Predicting optimal dihydroartemisinin-piperaquine regimens to prevent malaria during pregnancy for HIV-infected women receiving efavirenz. Unpublished
71. Savic RM, Jagannathan P, Kajubi R, Huang L, Zhang N, Were M, Kakuru A, Muhindo MK, Mwebaza N, Wallender E, Clark TD, Opira B, Kamya M, Havlir DV, Rosenthal PJ, Dorsey G, Aweeka FT, 2018. Intermittent Preventive Treatment for Malaria in Pregnancy: Optimization of Target Concentrations of Dihydroartemisinin-Piperaquine. Clin Infect Dis.
72. Kajubi R, Ochieng T, Kakuru A, Jagannathan P, Nakalembe M, Ruel T, Opira B, Ochokoru H, Ategeka J, Nyebare P, Clark TD, Kamya MR, Dorsey G, in press. Monthly sulfadoxine-pyrimethamine versus dihydroartemisinin-piperaquine for intermittent preventive treatment of malaria in pregnancy: A randomized controlled trial. Lancet.
73. Madanitsa M, Kalilani M, L., Mwapasa V, van Eijk A, Khairallah C, Chaluluka E, Ali D, Pace C, Smedley J, Thwai KL, Wang D, Kang'ombe A, Faragher B, Taylor SM, Steve Meshnick S, ter Kuile FO, Submitted. Control of malaria in pregnancy with scheduled intermittent screening with rapid diagnostic tests and treatment with dihydroartemisinin-piperaquine versus intermittent preventive therapy with sulphadoxine-pyrimethamine in Malawi: a randomized clinical trial.
74. (BHIVA) BHA, 2016. BHIVA guidelines for the treatment of HIV-1-positive adults with ART 2015 (2016 interim update) (https://www.bhiva.org/file/RVYKzFwyxpgiI/treatment-guidelines-2016-interim-update.pdf). (BHIVA) BHA, ed.
75. Allavena C, Dailly E, Reliquet V, Bonnet B, Pineau S, Andre-Garnier E, Boutoille D, Bouquie R, Raveleau A, Bouchez S, Billaud E, Raffi F, 2014. Switching from tenofovir/emtricitabine and nevirapine to a tenofovir/emtricitabine/rilpivirine single-tablet regimen in virologically suppressed, HIV-1-infected subjects. J Antimicrob Chemother 69: 2804-8.
76. Villar J, Cheikh Ismail L, Victora CG, Ohuma EO, Bertino E, Altman DG, Lambert A, Papageorghiou AT, Carvalho M, Jaffer YA, Gravett MG, Purwar M, Frederick IO, Noble AJ, Pang R, Barros FC, Chumlea C, Bhutta ZA, Kennedy SH, International F, Newborn Growth Consortium for the 21st C, 2014. International standards for newborn weight, length, and head circumference by gestational age and sex: the Newborn Cross-Sectional Study of the INTERGROWTH-21st Project. Lancet 384: 857-68.
77. Wang Y, 2010. Vascular biology of the placenta. Colloquium Series on Integrated Systems Physiology: From Molecule to Function: Morgan & Claypool Life Sciences, 1-98.

78. Madanitsa M, Kalilani L, Mwapasa V, van Eijk AM, Khairallah C, Ali D, Pace C, Smedley J, Thwai KL, Levitt B, Wang D, Kang'ombe A, Faragher B, Taylor SM, Meshnick S, Ter Kuile FO, 2016. Scheduled Intermittent Screening with Rapid Diagnostic Tests and Treatment with Dihydroartemisinin-Piperaquine versus Intermittent Preventive Therapy with Sulfadoxine-Pyrimethamine for Malaria in Pregnancy in Malawi: An Open-Label Randomized Controlled Trial. PLoS Med 13: e1002124.
79. Ahmed R, Poespoprodjo JR, Syafruddin D, Khairallah C, Pace C, Lukito T, Maratina SS, Asih PBS, Santana-Morales MA, Adams ER, Unwin V, Williams C, Chen T, Smedley J, Wang D, Faragher B, Price RN, ter Kuile FO, 2019 (in press). Intermittent preventive treatment (IPT), intermittent screening and treatment (IST) and single screening and treatment (SST) with dihydroartemisinin-piperaquine for the control of malaria in pregnancy in Indonesia: A cluster-randomised, open-label superiority trial. Lancet Infectious Diseases.
80. Gonzalez R, Desai M, Macete E, Ouma P, Kakolwa MA, Abdulla S, Aponte JJ, Bulo H, Kabanywanyi AM, Katana A, Maculuve S, Mayor A, Nhacolo A, Otieno K, Pahlavan G, Ruperez M, Sevene E, Slutsker L, Vala A, Williamsom J, Menendez C, 2014. Intermittent preventive treatment of malaria in pregnancy with mefloquine in HIV-infected women receiving cotrimoxazole prophylaxis: a multicenter randomized placebo-controlled trial. PLoS Med 11: e1001735.
81. Altman DG, Bland JM, 2003. Interaction revisited: the difference between two estimates. BMJ 326: 219.
82. Research NIfH, 2014. CT-toolokit. Available at: http://www.ct-toolkit.ac.uk/glossary/suspected-unexpected-serious-adverse-reactions-susar. Accessed 02 July 2016, 2016.
83. US Food and Drug Administration (FDA), 2019. Approved and Tentatively Approved Antiretrovirals in Association with the President's Emergency Plan. Available at: https://www.fda.gov/InternationalPrograms/PEPFAR/ucm119231.htm. Accessed 27 March 2019.
84. Ashley EA, Dhorda M, Fairhurst RM, Amaratunga C, Lim P, Suon S, Sreng S, Anderson JM, Mao S, Sam B, Sopha C, Chuor CM, Nguon C, Sovannaroth S, Pukrittayakamee S, Jittamala P, Chotivanich K, Chutasmit K, Suchatsoonthorn C, Runcharoen R, Hien TT, Thuy-Nhien NT, Thanh NV, Phu NH, Htut Y, Han KT, Aye KH, Mokuolu OA, Olaosebikan RR, Folaranmi OO, Mayxay M, Khanthavong M, Hongvanthong B, Newton PN, Onyamboko MA, Fanello CI, Tshefu AK, Mishra N, Valecha N, Phyo AP, Nosten F, Yi P, Tripura R, Borrmann S, Bashraheil M, Peshu J, Faiz MA, Ghose A, Hossain MA, Samad R, Rahman MR, Hasan MM, Islam A, Miotto O, Amato R, MacInnis B, Stalker J, Kwiatkowski DP, Bozdech Z, Jeeyapant A, Cheah PY, Sakulthaew T, Chalk J, Intharabut B, Silamut K, Lee SJ, Vihokhern B, Kunasol C, Imwong M, Tarning J, Taylor WJ, Yeung S, Woodrow CJ, Flegg JA, Das D, Smith J, Venkatesan M, Plowe CV, Stepniewska K, Guerin PJ, Dondorp AM, Day NP, White NJ, Tracking Resistance to Artemisinin C, 2014. Spread of artemisinin resistance in Plasmodium falciparum malaria. N Engl J Med 371: 411-23.
85. Chico RM, Moss WJ, 2015. Prevention of malaria in pregnancy: a fork in the road? Lancet.
86. 2010. Uniform requirements for manuscripts submitted to biomedical journals: Writing and editing for biomedical publication. Journal of Pharmacology & Pharmacotherapeutics 1: 42-58.
87. Wooden J, Kyes S, Sibley CH, 1993. PCR and strain identification in Plasmodium falciparum. Parasitol Today 9: 303-5.
88. Hofmann N, Mwingira F, Shekalaghe S, Robinson LJ, Mueller I, Felger I, 2015. Ultra-sensitive detection of Plasmodium falciparum by amplification of multi-copy subtelomeric targets. PLoS Med 12: e1001788.
89. Organisation WH, 2007. Recommended Genotyping Procedures (RGPs) to identify parasite populations.

90. Nag S DMD, Kofoed PE, Ursing J, O'Brien.L, Aarestrup FM, et al, 2016. High through-put resistance profiling of P. falciparum infections based on filter paper samples, custom dual indexing and Illumina NGS-technology reveals increase in molecular markers of resistance towards sulfadoxine-pyrimethamine, lumefantrine and quinine in Guinea-Bissau.
91. Alifrangis M, Enosse S, Pearce R, Drakeley C, Roper C, Khalil IF, Nkya WM, Ronn AM, Theander TG, Bygbjerg IC, 2005. A simple, high-throughput method to detect Plasmodium falciparum single nucleotide polymorphisms in the dihydrofolate reductase, dihydropteroate synthase, and P. falciparum chloroquine resistance transporter genes using polymerase chain reaction- and enzyme-linked immunosorbent assay-based technology. Am J Trop Med Hyg 72: 155-62.
92. Atwell JE, Thumar B, Robinson LJ, Tobby R, Yambo P, Ome-Kaius M, Siba PM, Unger HW, Rogerson SJ, King CL, Karron RA, 2016. Impact of Placental Malaria and Hypergammaglobulinemia on Transplacental Transfer of Respiratory Syncytial Virus Antibody in Papua New Guinea. J Infect Dis 213: 423-31.
93. Moro L, Bardaji A, Nhampossa T, Mandomando I, Serra-Casas E, Sigauque B, Cistero P, Chauhan VS, Chitnis CE, Ordi J, Dobano C, Alonso PL, Menendez C, Mayor A, 2015. Malaria and HIV infection in Mozambican pregnant women are associated with reduced transfer of antimalarial antibodies to their newborns. J Infect Dis 211: 1004-14.
94. Ogolla S, Daud, II, Asito AS, Sumba OP, Ouma C, Vulule J, Middeldorp JM, Dent AE, Mehta S, Rochford R, 2015. Reduced Transplacental Transfer of a Subset of Epstein-Barr Virus-Specific Antibodies to Neonates of Mothers Infected with Plasmodium falciparum Malaria during Pregnancy. Clin Vaccine Immunol 22: 1197-205.
95. Piriou E, Kimmel R, Chelimo K, Middeldorp JM, Odada PS, Ploutz-Snyder R, Moormann AM, Rochford R, 2009. Serological evidence for long-term Epstein-Barr virus reactivation in children living in a holoendemic malaria region of Kenya. J Med Virol 81: 1088-93.
96. Chung AW, Kumar MP, Arnold KB, Yu WH, Schoen MK, Dunphy LJ, Suscovich TJ, Frahm N, Linde C, Mahan AE, Hoffner M, Streeck H, Ackerman ME, McElrath MJ, Schuitemaker H, Pau MG, Baden LR, Kim JH, Michael NL, Barouch DH, Lauffenburger DA, Alter G, 2015. Dissecting Polyclonal Vaccine-Induced Humoral Immunity against HIV Using Systems Serology. Cell 163: 988-98.
97. Su LF, Han A, McGuire HM, Furman D, Newell EW, Davis MM, 2013. The promised land of human immunology. Cold Spring Harb Symp Quant Biol 78: 203-13.
98. Burton GJ, Charnock-Jones DS, Jauniaux E, 2009. Regulation of vascular growth and function in the human placenta. Reproduction 138: 895-902.
99. Karimu AL, Burton GJ, 1995. The distribution of microvilli over the villous surface of the normal human term placenta is homogenous. Reprod Fertil Dev 7: 1269-73.
100. Chisholm KM, Heerema-McKenney A, Tian L, Rajani AK, Saria S, Koller D, Penn AA, 2016. Correlation of preterm infant illness severity with placental histology. Placenta 39: 61-9.
101. Ogunyemi D, Murillo M, Jackson U, Hunter N, Alperson B, 2003. The relationship between placental histopathology findings and perinatal outcome in preterm infants. J Matern Fetal Neonatal Med 13: 102-9.
102. Conroy AL, Silver KL, Zhong K, Rennie M, Ward P, Sarma JV, Molyneux ME, Sled J, Fletcher JF, Rogerson S, Kain KC, 2013. Complement activation and the resulting placental vascular insufficiency drives fetal growth restriction associated with placental malaria. Cell Host Microbe 13: 215-26.
103. Driggers RW, Ho C-Y, Korhonen EM, Kuivanen S, Jääskeläinen AJ, Smura T, Rosenberg A, Hill DA, DeBiasi RL, Vezina G, Timofeev J, Rodriguez FJ, Levanov L, Razak J, Iyengar P, Hennenfent A, Kennedy R, Lanciotti R, du Plessis A, Vapalahti O, 2016. Zika Virus Infection with Prolonged Maternal Viremia and Fetal Brain Abnormalities. New England Journal of Medicine 374: 2142-2151.

104. Gérardin P, Sampériz S, Ramful D, Boumahni B, Bintner M, Alessandri J-L, Carbonnier M, Tiran-Rajaoefera I, Beullier G, Boya I, Noormahomed T, Okoï J, Rollot O, Cotte L, Jaffar-Bandjee M-C, Michault A, Favier F, Kaminski M, Fourmaintraux A, Fritel X, 2014. Neurocognitive Outcome of Children Exposed to Perinatal Mother-to-Child Chikungunya Virus Infection: The CHIMERE Cohort Study on Reunion Island. PLOS Neglected Tropical Diseases 8: e2996.
105. Baudin M, Jumaa AM, Jomma HJE, Karsany MS, Bucht G, Näslund J, Ahlm C, Evander M, Mohamed N, 2016. Association of Rift Valley fever virus infection with miscarriage in Sudanese women: a cross-sectional study. The Lancet Global Health 4: e864-e871.
106. Braack L, Gouveia de Almeida AP, Cornel AJ, Swanepoel R, de Jager C, 2018. Mosquito-borne arboviruses of African origin: review of key viruses and vectors. Parasites & Vectors 11: 29.
107. Baba M, Logue CH, Oderinde B, Abdulmaleek H, Williams J, Lewis J, Laws TR, Hewson R, Marcello A, D'Agaro P, 2013. Evidence of arbovirus co-infection in suspected febrile malaria and typhoid patients in Nigeria. The Journal of Infection in Developing Countries 7: 051-059.
108. Delvecchio R, Higa LM, Pezzuto P, Valadão AL, Garcez PP, Monteiro FL, Loiola EC, Dias AA, Silva FJ, Aliota MT, 2016. Chloroquine, an endocytosis blocking agent, inhibits Zika virus infection in different cell models. Viruses 8: 322.
109. Ventola CL, 2014. Mobile devices and apps for health care professionals: uses and benefits. P T 39: 356-64.
110. Akter S, Ray P, 2010. mHealth - an Ultimate Platform to Serve the Unserved. Yearb Med Inform: 94-100.

14 Financial aspects and conflict of interest

14.1 Funding for the trial

Funding to conduct the trial is provided by:

1. The Joint Global Health Trials Scheme of the UK Medical Research Council (MRC/ Wellcome Trust of Great Britain (WT) and the UK Department of International Development (DFID).
2. The European and Developing Countries Clinical Trials Partnership (EDCTP 2) which is funded by the European Union (EU), European Participating States and third parties including Product Development Partners (PDPs), Private Sector Industry, and International Development Partners.

The funders had no role in the design of this trial and will not have any during the execution, analysis, interpretation of the data, or decision to submit the results.

14.2 Provision of study drugs

DP and DP placebo will be provided at reduced rates either by Guilin Pharma, based in China, or Alfasigma (formerly Sigma-Tau). CTX will be purchased a GMP manufacturer identified in section 7.3.1.5. Fixed-dose formulations of DTG-based combination will be obtained from the local Governments or a GMP manufacturer identified in section 7.3.1.5. When so requested by manufacturers, the study will provide copies of safety reports of SAEs and AEs to the manufacturers (expedited where required). The manufacturers will not be involved in the design of the trial.

15 Budget & budget justification

See 16.4, Appendix IV. Budget and budget justification, page 82.

16 APPENDICES

16.1 APPENDIX I. ROLE OF INVESTIGATORS AND COLLABORATORS

16.1.1 Definition investigator and collaborator

16.1.1.1 Investigators

Investigators are directly involved with the field research and, except for the trial statisticians, and live and work in Kenya and Malawi.

16.1.1.2 Collaborators

Collaborators are not involved with any of the field work and are either PhD supervisors living overseas, provide purely technical support, or support the study with the laboratory assays in their overseas laboratories. Each home institutions will sign MOUs in the form of MTA and/or DTAs with the overseas collaborators before samples or data can be shipped overseas.

16.1.2 Protocol development: authors' contributions

Hellen Barsosio (HB), Mwayiwawo Madanitsa (MM) and Feiko terKuile (FtK) wrote the first draft of the protocol. All investigators contributed to the refinement of the study protocol and approved the final versions.

16.1.3 Role Investigators

16.1.3.1 Chief Investigator

Prof Feiko ter Kuile (MD, PhD), is a Professor of Tropical Epidemiology from the Liverpool School of Tropical Medicine (LSTM) based at KEMRI in Kisumu. He is the Chief Investigator and grant holder and will carry overall responsibility for the coordination of the trial and for the linkages with the sponsor, funders and with international partners involved. He will also liaise with WHO.

16.1.3.2 Co-principal investigators

Dr Hellen Barsosio (MD, MSc), is a research medical officer with KEMRI/CDC/Liverpool collaboration, based in Kisumu, Kenya. She will be the co-Principal Investigator for Kenya and as overall trial Manager. She will also liaise with the local hospitals, the ethics committees, the regulators and the MoH in country. She will be supervised locally by *Dr Simon Kariuki,* the KEMRI-CDC Malaria Branch Chief and internationally by Prof Feiko ter Kuile.

Dr Mwayiwawo Madanitsa (MD, PhD), is a postdoctoral research fellow with COM and LSTM, based at COM in Blantyre, Malawi. He will act as the co-Principal Investigator for Malawi and as overall trial Manager. He will a l s o liaise with the local hospitals, the ethics committees, the regulators and the MoH in country. He will be supervised locally by Professor Victor Mwapasa and internationally by FtK.

Dr Simon Kariuki (PhD), is the KEMRI/CDC Malaria Branch Chief and will be the co-Principal Investigator for Kenya. Dr Kariuki the KEMRI-CDC Malaria Branch Chief and oversees all research studies that fall under the Malaria Branch. He will supervise Dr Hellen Barsosio in addition to providing local support with regulators and the MoH in country.

16.1.3.3 Co-investigators

Prof Duolao Wang, PhD is Professor of Medical Statistics at the Liverpool School of Tropical Medicine and will be the trial statistician.

James Dodd, MSc at the Liverpool School of Tropical Medicine is a statistician who will be involved with the data merging and analysis under supervision of Prof Duolao Wang.

George Githuka, MD, MSc from NASCOP, MoH, Kenya will provide technical advice and oversight of the PMTCT transmission component and cART guidelines in Kenya.

Clifford Banda (MD, MSc) is a medical officer from the CoM, Malawi with a special interest in clinical pharmacology and will be responsible for the conduct of the pharmacokinetic component of the study

Prof Victor Mwapasa (MD, MPH, PhD) is a Professor of Public Health and Dean of Research and Postgraduate Studies at the CoM. He will serve as the co-investigator in Malawi and provide site co-leadership and liaison with administrative and policy stakeholders of the trial and co-lead the capacity development activities at the College of Medicine, University of Malawi.

Prof Kamija Phiri (MD, PhD) is Professor in Clinical Epidemiology, Public Health Department, and Dean of the School of Public Health & Family Medicine, and Director, of the Training & Research Unit of Excellence (TRUE), College of Medicine, University of Malawi. He has over 10 years' experience in clinical research and will provide site co-leadership for the study site in Zomba, Malawi.

Prof Ken Maleta (MD PhD) is Professor in Community Health, College of Medicine, University of Malawi. He is a nutritional and growth expert and will support the activities in the Mangochi site in Malawi.

16.1.4 Collaborators

Non-engaged collaborators are not classified as investigators and have an advisory and supporting role and are not directly involved with the research activities on site.

16.2 APPENDIX II. TERMS OF REFERENCE OVERSIGHT COMMITTEES

16.2.1 Trial Management Group (TMG)

16.2.1.1 Purpose

The TMG is responsible for the administrative management and day to day running of the trial.

16.2.1.2 Membership

1. Overall trial manager (Dr Hellen Barsosio)
2. Other co-Principal Investigators (4x) or representatives
3. Country trial Coordinators (4x)
4. Lead Data manager
5. Lead administrators
6. Chief Investigator (ad hoc)
7. Others who are involved in the day to day running of the trial are invited ad hoc

16.2.1.3 Responsibilities:

- Study planning
- Organisation of the Trial Steering Committee and Data Monitoring and Ethics Committee (DMEC) meetings
- Provide risk report to regulators, manufactures and ethics committees
- SUSAR [Serious unexpected suspected adverse events] reporting
- Responsible for the trial master file
- Budget administration and contractual issues
- Advice for lead investigators
- The organisation of central data management and sample collection

16.2.2 Trial Steering Committee (TSC)

16.2.2.1 Membership TSC

16.2.2.1.1 Independent members

1. Chair: Dr Laurence Slutsker, PATH, is the director of malaria and neglected tropical diseases, at the Center for Malaria Control and Elimination, PATH. He is malaria and HIV expert, and member of the Malaria Policy Advisory Committee (MPAC) at WHO and chair of WHO' Evidence Review Group for malaria in pregnancy
2. Associate Professor Miriam Laufer, (PhD), University of Maryland, School of Medicine, Baltimore, USA (paediatrician, and malaria epidemiologist and public health expert with specific expertise in pregnancy, WHO ERG member);
3. Professor Grant Dorsey, MD, PhD University of California, San Francisco (malaria epidemiologist and expert on intermittent DP, CI of the previous trials in Uganda.[29,60]
4. Professor Per Ashorn, MD, PhD, Department of Maternal, Child, and Adolescent Health, World Health Organization, Geneva, Switzerland (paediatrician, infectious disease and growth and nutrition expert and CI of previous intervention trials in pregnancy participants using intermittent azithromycin combined with IPTp).
5. Dr Matt Cairns, from LSHTM, will be TSC statistician.

16.2.2.1.2 Trial members

- Prof Feiko ter Kuile, Chief Investigator
- Dr Hellen Barsosio, representing the co-Principal Investigators
- Prof Duolao Wang, trial statistician
- The other co-PIs, co-investigators, collaborators and the trial statistician will attend the meetings if and when required.

16.2.2.1.3 Observers

- EDCTP
- MRC

16.2.2.2 Roles and Responsibilities TSC

The TSC is a trial governing body which includes a majority of its members who are independent of the trial management group. The TSC concentrates on the progress of the trial and ensures that the trial is conducted to the standards set out in the Guidelines for Good Clinical Practice with consideration given to participant safety and provision of informed consent.

- To evaluate the progress of the trial in relation to the timeliness, data quality and other factors that can affect the overall objectives of the trial
- To ensure participant rights and safety are adhered to and that the protocol demands freely given informed consent
- To review relevant information from other sources
- To consider the recommendations of the Data Monitoring and Ethics Committee (DMEC) and in light of it to inform the Chief Investigator and TMG the need to make changes to the trial protocol
- To ensure that the trial results are disseminated appropriately, and consideration be given to the implementation of the results into policy

16.2.2.3 Operational TSC

The CI will present the full protocol to the TSC as an agenda before the start-up of data collection. The TSC members shall review the timeline set out in the protocol for participant recruitment, informed consent documents and plans for data safety monitoring.

The TSC shall see that the finalised protocol is sent to the sponsor and funders before the start of participant recruitment and data collection.

The TSC in its first meeting shall approve the nominated members of the DMEC and establish the DMEC which shall meet regularly to review and report on the data quality and the results of interim analyses.

In all their deliberations the TSC should consider any deviations from the trial protocol, participant safety and information provided to the participants and consenting procedures.

16.2.2.4 Frequency of Meetings

The TSC shall have a start-up meeting to discuss the protocol and establish the DMEC. A second meeting shall take place before the initiation of the trial to finalise the protocol and approve the commencement of the trial. After that, the TSC will generally meet once a year in the life span of the trial and one meeting at the closure of the trial.

The Chair and at least 2 of the three independent members together with the CI and trial co-ordinator shall constitute the quorum. If so required, in addition, a member of the funder can be invited to attend the meetings.

16.2.2.5 Trial Reports and actions TSC meeting

The TSC shall provide at each meeting a summary report of their findings and recommendations which must be submitted to the funder, the Sponsor and the TMG.

If the TSC makes a recommendation that the trial should be stopped or suspended, the Sponsor will take the necessary action to ensure that new recruitment to the trial is stopped whilst the TSC report is evaluated and the Research Ethics Committee is informed.

16.2.3 Data Monitoring and Ethics Committee (DMEC)

16.2.3.1 Membership DMEC (preliminary)

1. Professor Andy Stergachis, Professor of Pharmacy and Global Health, and pharmaco-epidemiologist, University of Washington will chair the DMEC. He chairs the safety working group of the Malaria in Pregnancy Consortium since 2008 and chaired several DMECs related to similar malaria in pregnancy trials.
2. Professor Ib Christian Bygbjerg, from the Institute for Public Health, University of Copenhagen, Denmark
3. Dr Billy Ngasala, from Department of Parasitology and Medical Entomology, Muhimbili University of Health and Allied Sciences (MUHAS), Tanzania
4. Dr Kathleen Wannemuehler (kpw9@cdc.gov), Centers for Disease Control and Prevention (CDC), Atlanta, USA (Statistician)

16.2.3.2 Role DMEC

The DMEC consist of 3-4 members (including one or more clinicians and one statistician, all with experience in clinical trials).

The DMEC shall assess the data regularly (before the annual TSC meeting) to review the data and the interim analysis. The assessment could be via email or another electronic medium annually before the TSC meeting. In the first year of recruitment more frequent assessment (bi-annually) is recommended for this trial.

The members should be the only personnel to see the results separated by treatment group during the trial. They are independent and look at the trial from an ethical point of view of the participant safety, future participants and society in general. It is their responsibility to prevent participants being exposed to any excess risks by recommending to the Trial Steering Committee (TSC) for the trial suspension or termination early if the safety or efficacy results are sufficiently convincing. The trial statistician is usually invited to attend part of the DMEC meeting to present the most current data from the trial. This will be blinded, unless the DMEC specifically requests for an unblinded analysis.

16.2.3.3 Responsibilities DMEC

- To determine how frequently interim analysis of trial data should be undertaken.
- To consider the blinded or unblinded interim data from the trial and relevant information from other sources.
- To consider any requests for unblinding and release of interim trial data and to recommend to the TSC on the importance of this.
- To report (following each DMEC meeting) to the TSC and to recommend whether the trial should continue, the protocol be modified or the trial be stopped.

A full confidential report should be submitted in writing to the TSC at the end of each DMEC meeting

16.3 Appendix III. Declaration of Helsinki

WORLD MEDICAL ASSOCIATION DECLARATION OF HELSINKI

Recommendations guiding physicians in biomedical research involving human subjects. Adopted by the 18th World Medical Assembly, Helsinki, Finland, June 1964. Amended by the 29th World Medical Assembly, Tokyo, Japan, October 1975, 35th World Medical Assembly, Venice, Italy, October 1983, 41st World Medical Assembly Hong Kong, September 1989 and the 48th General Assembly, Somerset West, Republic of South Africa, October 1996

INTRODUCTION

It is the mission of the physician to safeguard the health of the people. His or her knowledge and conscience are dedicated to the fulfilment of this mission.

The Declaration of Geneva of the World Medical Association binds the physician with the words, "The health of my patient will be my first consideration", and the International Code of Medical Ethics declares that, "A physician shall act only in the patient's interest when providing medical care which might have the effect of weakening the physical and mental condition of the patient."

The purpose of biomedical research involving human subjects must be to improve diagnostic, therapeutic and prophylactic procedures and the understanding of the aetiology and pathogenesis of disease.

In current medical practice most diagnostic, therapeutic or prophylactic procedures involve hazards. This applies especially to biomedical research.

Medical progress is based on research which ultimately must rest in part on experimentation involving human subjects.

In the field of biomedical research, a fundamental distinction must be recognized between medical research in which the aim is essentially diagnostic or therapeutic for a patient, and medical research, the essential object of which is purely scientific and without implying direct diagnostic or therapeutic value to the person subjected to the research.

Special caution must be exercised in the conduct of research which may affect the environment, and the welfare of animals used for research must be respected.

Because it is essential that the results of laboratory experiments be applied to human beings to further scientific knowledge and to help suffering humanity, the World Medical Association has prepared the following recommendations as a guide to every physician in biomedical research involving human subjects. They should be kept under review in the future. It must be stressed that the standards as drafted are only a guide to physicians all over the world. Physicians are not relieved from criminal, civil and ethical responsibilities under the laws of their own countries.

Basic principles

1. Biomedical research involving human subjects must conform to generally accepted scientific principles and should be based on adequately performed laboratory and animal experimentation and on a thorough knowledge of the scientific literature.
2. The design and performance of each experimental procedure involving human subjects should be clearly formulated in an experimental protocol which should be transmitted for consideration, comment and guidance to a specially appointed committee independent of the investigator and

the sponsor provided that this independent committee is in conformity with the laws and regulations of the country in which the research experiment is performed.

3. Biomedical research involving human subjects should be conducted only by scientifically qualified persons and under the supervision of a clinically competent medical person. The responsibility for the human subject must always rest with a medically qualified person and never rest on the subject of the research, even though the subject has given his or her consent.
4. Biomedical research involving human subjects cannot legitimately be carried out unless the importance of the objective is in proportion to the inherent risk to the subject.
5. Every biomedical research project involving human subjects should be preceded with careful assessment of predictable risks in comparison with foreseeable benefits to the subject or to others. Concern for the interests of the subject must always prevail over the interests of science and society.
6. The right of the research subject to safeguard his or her integrity must always be respected. Every precaution should be taken to respect the privacy of the subject and to minimize the impact of the study on the subject's physical and mental integrity and on the personality of the subject.
7. Physicians should abstain from engaging in research projects involving human subjects unless they are satisfied that the hazards involved are believed to be predictable. Physicians should cease any investigation if the hazards are found to outweigh the potential benefits.
8. In publication of the results of his or her research, the physician is obliged to preserve the accuracy of the results. Reports of experimentation not in accordance with the principles laid down in this Declaration should not be accepted for publication.
9. In any research on human beings, each potential subject must be adequately informed of the aims, methods, anticipated benefits and potential hazards of the study and the discomfort it may entail. He or she should be informed that he or she is at liberty to abstain from participation in the study and that he or she is free to withdraw his or her consent to participation at any time. The physician should then obtain the subject's freely-given informed consent, preferably in writing.
10. When obtaining informed consent for the research project, the physician should be particularly cautious if the subject is in a dependent relationship to him or her or may consent under duress. In that case the informed consent should be obtained by a physician who is not engaged in the investigation and who is completely independent of this official relationship.
11. In case of legal incompetence, informed consent should be obtained from the legal guardian in accordance with national legislation. Where physical or mental incapacity makes it impossible to obtain informed consent, or when the subject is a minor, permission from the responsible relative replaces that of the subject in accordance with national legislation. Whenever the minor child is in fact able to give a consent, the minor's consent must be obtained in addition to the consent of the minor's legal guardian.
12. The research protocol should always contain a statement of the ethical considerations involved and should indicate that the principles enunciated in the present Declaration are complied with.

Medical research combined with professional care (Clinical Research)

1. In the treatment of the sick person, the physician must be free to use a new diagnostic and therapeutic measure, if in his or her judgement it offers hope of saving life, re-establishing health or alleviating suffering.
2. The potential benefits, hazards and discomfort of a new method should be weighed against the advantages of the best current diagnostic and therapeutic methods.

3. In any medical study, every patient - including those of a control group, if any - should be assured of the best proven diagnostic and therapeutic method. This does not exclude the use of inert placebo in studies where no proven diagnostic or therapeutic method exists.
4. The refusal of the patient to participate in a study must never interfere with the physician-patient relationship.
5. If the physician considers it essential not to obtain informed consent, the specific reasons for this proposal should be stated in the experimental protocol for transmission to the independent committee (I,2).
6. The physician can combine medical research with professional care, the objective being the acquisition of new medical knowledge, only to the extent that medical research is justified by its potential diagnostic or therapeutic value for the patient.

Non-therapeutic biomedical research involving human subjects (Non-clinical biomedical research)

1. In the purely scientific application of medical research carried out on a human being, it is the duty of the physician to remain the protector of the life and health of that person on whom biomedical research is being carried out.
2. The subjects should be volunteers -- either healthy persons or patients for whom the experimental design is not related to the patient's illness.
3. The investigator or the investigating team should discontinue the research if in his/her or their judgement it may, if continued, be harmful to the individual.
4. In research on man, the interest of science and society should never take precedence over considerations related to the well-being of the subject.

16.4 Appendix IV. Budget and budget justification

16.4.1 Budget

Generic site budget estimates and summaries

Item Description	Total Cost Euro	Total Cost USD*
PERSONNEL		
Actual costs	437,340 €	$ 491,640
Unit costs	91,860 €	$ 103,260
Subtotal cost Personnel	529,200 €	$ 594,900
GOODS AND SERVICES		
Certificates on Financial Statements	6,000 €	$ 6,720
Equipment	3,900 €	$ 4,380
Consumables and supplies	43,560 €	$ 48,960
Training	2,100 €	$ 2,400
Subtotal cost Goods and Services	55,560 €	$ 62,460
PATIENT ADMINISTRATION		
Patient costs	114,180 €	$ 128,340
Subtotal costs Patient Administration	114,180 €	$ 128,340
TRAVEL		
In-country work-related travel	39,000 €	$ 43,800
Subtotal Travel	39,000 €	$ 43,800
TOTAL DIRECT COST		
Total Direct Costs	737,940 €	$ 829,500
Indirect Costs (25%)	184,485 €	$ 207,375
TOTAL COSTS	922,425 €	$ 1,036,875

*The original budget is in Euros and subject to exchange rate fluctuations with the US Dollar and local currency

* The above are generic budget estimates across Kenya and Malawi and specific costs will be subject to minimal variation between countries

16.4.2 Budget Justification

Funding has been approved for fieldwork starting in 2019 and an additional year to support completion of capacity development training. The overall budget includes central sponsorship support, trial insurance and trial monitoring, a central safety register, data management support and for trial coordination, international travel and academic capacity training. The budget also includes indirect costs.

16.5 Appendix V. Participant information sheets and informed consent statements

16.5.1 Participant Information Sheet: Main trial (all participants) (English)

See separate document for participant information sheets and informed consent statements

16.5.2 Consent statement for screening and participation in the main trial (all participants) (English)

See separate document for participant information sheets and informed consent statements

16.5.3 Consent Statement for long term storage of samples and use for future research (all participants) (English)

See separate document for participant information sheets and informed consent statements

16.5.4 Participant Information Sheet for cardiac safety monitoring (subgroup) (English)

See separate document for participant information sheets and informed consent statements

16.5.5 Consent Statement for cardiac safety monitoring (sub-group) (English)

See separate document for participant information sheets and informed consent statements

16.6 Appendix VI. Description of other clinical and laboratory methods

This section 'Appendix VI. Description of other clinical and laboratory methods' of the appendix, describes the clinical and laboratory-based assays for some secondary outcomes conducted in subgroups of participants in specific study sites or otherwise in a random sample of participants in all sites. The volume and timing of these samples are described in 'Table 1: Summary Table of Study Design and Schedule of Assessment, page 15, and in section 7.5 'Participants timeline', page 37 onwards. The purpose if this appendix text is to describe the assays and measures of the procedures in more detail.

16.6.1 Pharmacokinetic studies of drug-drug interactions between piperaquine and standard antiretroviral therapy

Collaborating institutions and contact persons: Dr Eva Maria Hodel (University of Liverpool/LSTM); Dr Clifford George Banda (COM, University of Malawi); Division of Clinical Pharmacology and Toxicology Laboratory-University Hospital (CHUV), Lausanne, Switzerland; Prof Karen Barnes (Division of Clinical Pharmacology, University of Cape Town.

Rationale and study aim: Because no previous studies have looked at the combination DP and DTG-based cARTs, there is a need for further PK profiling of piperaquine-DTG drug-drug interactions to verify the pharmacological model predictions. This includes an assessment of the effect of the cARTs on piperaquine pharmacokinetics, and the effect of repeated piperaquine dosing on the steady state concentration of the cARTs and CTX.

Design: We will conduct sparse pharmacokinetic/dynamic studies among approximately 80 women enrolled in the main trial (40 in each arm) who are on DTG-based cARTs and daily CTX. These women will also be part of the cardiac monitoring component.

Methods: Venous blood samples (2 ml) will be collected at baseline before the first dose and 4 hours after the last dose of the 3-day DP regimen, and then at approximately four weeks intervals to measure the CTX, cART and piperaquine trough levels just before the next monthly DP dose. Each woman will also contribute two piperaquine peak samples taken approximately four hours after a monthly course. At delivery, paired maternal plasma, placental blood, and umbilical cord blood samples collected at delivery will be assessed for piperaquine concentrations in approximately.

Laboratory assays: Collected blood samples will be centrifuged to obtain plasma which will be stored in a −80 °C freezer until shipment on dry ice to the Division of Clinical Pharmacology and Toxicology laboratory at the University Hospital (CHUV) in Lausanne, Switzerland for analysis. Plasma concentrations of cotrimoxazole, dihydroartemisinin, efavirenz, dolutegravir, two nucleoside reverse transcriptase inhibitors co-administered with dolutegravir and the antimalarial drug piperaquine, will be determined by a multiplex liquid chromatography coupled to tandem triple-stage mass spectrometry (LC-MS/MS) assay.

Pharmacokinetic analysis peripheral blood: We will measure CTX, DP and standard ART concentrations to characterise efficacy (troughs) and safety (peaks) of DP. The effect of piperaquine on dolutegravir and CTX mean steady-state (SS) concentrations will be assessed by comparing ARTs steady-state concentration in women in the piperaquine arm and those in the CTX-only arm (Placebo-DP). Piperaquine concentrations will also be compared against those in HIV-negative women from the ongoing IMPROVE trial.

Placental circulation: Concentrations will be compared with those in 25 HIV-negative women (i.e. not on cARTs) from the ongoing IMPROVE trial to assess the impact of cART on the plasma concentrations

of piperaquine that penetrates the placental circulation. Each sample will be 200 µL of venous blood (citrate).

Defining minimum inhibitory concentrations: To assess the association between piperaquine's PK profile and efficacy, we will use a matched nested case-control method, where a 'case' is defined women with evidence of malaria infection at delivery and a 'control' as women who are negative for malaria at delivery by PCR and placental histology. Exposure will be defined as trough levels collected at different scheduled follow-up visits.

16.6.2 Impact of antimalarial resistance on protective efficacy

Collaborating institutions and contact persons: Ass. Prof Dr Michael Alifrangis, University of Copenhagen

Objective: 1. To evaluate the contribution of recrudescence and reinfection to prevalent and incidence of malaria infection throughout pregnancy; 2. To investigate the prevalence of molecular antimalarial resistance markers in parasite isolates.

Rationale: The use of DP, a slowly eliminated antimalarial, for IPTp may pose as a source of drug pressure that may drive the emergence of piperaquine resistance in sub-Saharan Africa. The presence of high-grade sulphadoxine-pyrimethamine resistant parasites markers may also be predictive of the efficacy of cotrimoxazole resistance. To evaluate this, we will conduct molecular studies to detect malaria infections from enrolment to delivery, differentiate these as new or recrudescent infections and determine their molecular resistance status to artemisinins and piperaquine and CTX and sulphadoxine-pyrimethamine.

Methods

Diagnostic qPCR: Dry blood spots or redcell pellets will be prepared at enrolment, all scheduled and unscheduled (sick) visits and at delivery. DNA will be extracted using chelex-100 extraction[87] or Qiagen DNA extraction for further qPCR targeting the high-copy telomere-associated repetitive element 2 (limit of detection 0.15 parasites/µl blood).[88]

Genotyping to distinguish recrudescent and re-infections: qPCR positive samples from participants at two sequential monthly visits will be used for PCR genotyping of the polymorphic *P.falciparum* genes MSP2, GLURP and MSP1; interpretation of results will be performed using standardised methodologies.[89]

Molecular markers of antimalarial drug resistance: All *P. falciparum*-qPCR positive samples collected will be used for the evaluation of artemisinin, piperaquine, and antifolate resistance molecular markers. All PCR positive samples at enrolment will be examined for SNPs in the genes *Pfdhfr*, *Pfdhps*, *K13* and *Pfmdr*-1 and plasmepsin 2,3. A high-throughput next-generation platform for targeted sequencing using Illumina®-basedtechnology[90]will be applied as the main method. After index PCR, the gene fragments will be sequenced using the Illumina Miseq® platform available through collaboration with researchers at the Danish Technical University, Denmark. In addition, direct Sanger sequencing methods will be applied to a subset of samples targeting mainly K13 to confirm novel findings and as a gold standard comparison using published PCR methods [6] and after PCR, also send for commercial sequencing. A PCR sequence-specific oligonucleotide probe (SSOP)-ELISA[91] will be deployed as a reference method to the Illumina methodology regarding Pfdhfr and Pfdhps data.

Data analysis: The degree of antifolate resistance in the parasite population will be expressed as the frequency of the *Pfdhps*-A437G, K540E, or A581G mutations, which predict the frequency of quadruple, quintuple, and sextuple *Pfdhfr–Pfdhps* mutant haplotypes, respectively. In addition, we

will assess prevalence of molecular markers associated with tolerance/resistance to artemisinin and piperaquine (*K13* and *Pfmdr1,* plasmepsin-2). These molecular data will be used as potential effect modifiers in the impact analysis of the main trial.

16.6.3 Impact on maternal antibody, trans-placental antibody transfer and multi-pathogen neonatal cell-mediated immune responses

Collaborating institutions and contact persons: Ass Prof Ann Moormann, University of Massachusetts, Worcester, USA and Prof Stephen Rogerson, University of Melbourne, Australia.

Objective: To determine the impact of the interventions and of malaria and HIV during pregnancy on maternal immunity, transfer of maternal antibodies and the development of cell-mediate immunity in infants to other pathogens, in addition to malaria.

Rationale: Maternal antibodies may protect pregnant participants from malaria, but placental malaria has been associated with impaired transfer of maternal antibodies and hypergammaglobulinemia.[92,93] This negatively impacts the newborn's ability to be protected against malaria as well as viral infections.[92,94] The scope of this lack of maternal antibody protection tends to be studied one or two infections at a time. Using multiplex methods,[95,96] we will be able to simultaneously measure the impact of the study interventions on a panel of antibody titres and cell-mediated immune responses to multiple pathogens within the same study participants using extremely low volumes of peripheral blood.

Methods

Samples will be taken at all scheduled clinic visits, delivery and 1, and 6-8 weeks infant visit (heel prick) for:

a) Serology profiling: Using a Luminex, multiplex suspension bead-based assay to simultaneously measure antibody titers to a panel of individual antigens or immunologic peptides against malaria (ie MSP1, AMA1, CSP, LSA1, etc), EBV, CMV, RSV, measles virus, tetanus toxoid, arboviruses etc.[95] We will use a Systems Serology approach to identify protective antibodies.[96]

b) Cell-mediated immunity: Using multi-parameter flow cytometry and potentially single cell RNAseq[97] we will efficiently and comprehensively interrogate immunity specific to pathogens and EPI vaccine-induced responses.

16.6.4 Impact on infant adaptive and innate-like immunity

Collaborating institutions and contact persons: Dr Cristiana Cairo and Prof Miriam Laufer, University of Maryland), Baltimore, USA.

Objective: To determine the impact on adaptive and innate-like immune cell subsets of infants that are likely affected by prenatal exposure to HIV and/or malaria.

Rationale: Prevention of mother to child transmission strategies successfully avert congenital infection, but early studies showed that HIV exposed uninfected infants (HEU) are more susceptible to common infections than their unexposed counterparts and have higher mortality rate. Even after widespread availability of combination antiretroviral therapy (cART), differences in morbidity and mortality between HIV exposed and unexposed infants were still reported, though significantly less pronounced than in early studies. The immunologic alterations that are responsible for this phenomenon among HEU infants have not been identified conclusively, due to confounding variables and conflicting results between studies. In addition, pregnancy associated malaria, particularly placental malaria, also perturbs some immune cell subsets in neonates, and infants born to women

with placental infection are at increased risk of malaria in the first year of life. We will study adaptive and innate-like immune cell subsets that are likely affected by prenatal exposure to HIV and/or malaria and compare this between study arms.

Methods: Cord blood samples will be collected from 600 participants. Cord blood mononuclear cells (CBMC) will be isolated and cryopreserved in the vapor phase of liquid nitrogen at the University of Malawi College of Medicine by trained laboratory technicians. CBMC specimens will be shipped to the University of Maryland Baltimore for analyses requiring equipment that is not available in Malawi. At the University of Maryland School of Medicine, the specimens will be thawed and used for analyses of innate-like and adaptive immune cell subsets (including Mucosal Associated Invariant T cells, Th17 cells and T regulatory cells). In particular, we will characterize: (1) T cell differentiation and activation state ex vivo; (1) Activation of T cell cytokine production and proliferation in response to live BCG (the vaccine against TB, administered at birth in sub-Saharan Africa) in vitro. In order to collect the data described above, we will use polychromatic flow cytometry (14 parameters per panel).

16.6.5 Impact on biomarkers of placental function and adverse birth outcomes

Collaborating institutions and contact persons: Prof Kevin Kaine, University of Toronto, Canada

Objective: To determine the impact on biomarkers and pathways regulating placental development and function and that are predictive of adverse birth outcomes. e.g. fetal growth restriction (FGR), preterm birth (PTB) in a prospective study of pregnant participants at high risk of malaria.

Rationale: Normal placental development is essential to support the growth of the fetus throughout pregnancy. Malaria in early pregnancy can disrupt the normal the vasculogenic processes in the first trimester needed to regulate de novo formation and growth of blood vessels.[98] Angiogenic processes beginning in the second trimester induce remodeling of the underlying architecture of the placenta to allow for increased blood flow and surface area for nutrient exchange required for exponential fetal growth in the 3rd trimester.[98,99] Healthy birth outcomes require tight regulation of these vasculogenic, angiogenic, and inflammatory pathways and disruptions to these critical axes that can result in placental dysfunction and poor birth outcomes. For example, placental vascular dysfunction is associated with multiple pregnancy pathologies including gestational diabetes, preeclampsia, and malaria in pregnancy.[100-102] The early identification of pregnancies at risk of adverse birth outcomes would enable risk stratification for increased monitoring and interventions to improve outcome. We propose to externally validate a panel of biomarkers to examine these markers in the context of risk factors contributing to FGR and PTB, including malaria infection in pregnancy, and determine the impact of the two different interventions on these biomarkers.

Methods Venous blood samples (minimum required volume of 250 µL) collected from participants at study enrolment, scheduled antenatal visits and delivery will be processed via a custom multiplex Luminex®-based assay to quantify plasma levels of markers of placental function, inflammation and development including Angiopoietin-1 (Ang-1), Angiopoietin-2 (Ang-2), Angiopoietin-Like 3 (AngptL3), Vascular Endothelial Growth Factor (VEGF), Soluble fms-like tyrosine kinase 1 (sFlt-1), soluble Tumor Necrosis Factor Receptor 2 (sTNFR2), Placental Growth Factor (PGF), Interleukin-18 Binding Protein (IL-18BP), soluble Intercellular Adhesion Molecule-1 (sICAM-1), soluble Endoglin (sEndoglin), C-reactive protein (CRP), Chitinase-3-Like Protein-1 (CHI3L1), complement components including C5a (C5a), and brain derived neurotrophic factor (BDNF), and inflammatory biomarkers such as C-reactive protein (CRP). Proteins were selected based on evidence of a critical role of each protein in inflammatory pathways and placental vasculogenesis, as well as associations with birth and fetal outcomes.

16.6.6 Impact on arboviral co-infections

Collaborating institutions and contact persons: Dr George Warimwe, Dr Charles Nyaigoti and Prof Philip Bejon, KEMRI-Wellcome Trust, Kenya

Objectives: 1) To determine the effect of malaria-arboviral co-infections on adverse pregnancy outcomes in HIV infected women, 2) To evaluate the impact of piperaquine on arboviral co-infections.

Rationale: Mosquito-borne viruses (arboviruses) are gaining notoriety as pathogens associated with adverse outcomes in pregnancy including microcephaly with Zika virus[13,103], neurological complications seen with Chikungunya[104], miscarriages seen with Rift Valley fever virus (RVFV)[105] and pre-term delivery and still births seen with Dengue virus co-infection in India.[13] Concurrent arboviral-malaria infections have been documented in sub-Saharan Africa.[106,107] However, the impact of these co-infections on adverse pregnancy outcomes are unknown. It's also unclear whether anti-malarials used in prevention and treatment of malaria confers protection against arboviral co-infections. A recent study showed chloroquine inhibited Zika virus replication in-vitro cells and conferred protection against Zika virus infection in pregnant mice[37,108] Piperaquine is structurally similar to chloroquine. We will determine the impact of piperaquine on arboviral co-infections by comparing the incidence of arboviral infections between the CTX-DP and the CTX-alone arm.

Methods: All participants will be included. We will test for the presence of arbovirus and protective antibodies in all women at enrolment, some scheduled visits, sick visits and delivery in venous blood and urine samples at sick febrile visits. Arboviruses of interest will include: flaviviruses, alpha viruses, bunyaviruses and other viruses. Blood samples will be collected in EDTA bottles and plasma stored at -80 °C, before being shipped to and analysed in KEMRI. Serological assays will include IgG and IgM ELISAs and virus neutralising assays. The presence of virus will be determined by qRT-PCR assays using primers and probes targeting a broad range of flaviviruses, alphaviruses and bunyaviruses, and a randomly selected fraction processed by agnostic deep sequencing protocols to identify emerging viral pathogens not included in the directed PCRs/serological assays.

16.6.7 Use of mobile phone Unstructured Supplementary Service Data (USSD) platform to enhance protocol adherence and monitoring of tolerance

Collaborating institutions and contact persons: Prof Wilson Mandala, Malawi University of Science and Technology, Malawi; Mr Manota Mphande, Logic Bay Systems, Malawi

Objectives: 1) To enhance adherence to study drug and study schedule using a mobile phone USSD platform, 2) To enhance monitoring of tolerance and safety assessment of study drugs using a mobile phone USSD platform, 2

Rationale: In resource limited settings where polypharmacy is prevalent, mobile health technology is an essential platform to monitor and facilitate treatment adherence and enhance safety monitoring through a real time interactive platform to ensure improved outcomes.[109,110] We will explore to use of a USSD platform to boost the standard mobile phone contacting methods used in the trial.

Methods: Approximately, 100 participants will be included in an exploratory evaluation. If the exploratory evaluation is successful, we will explore, subject to funding, how the USSD platform can be scaled up to other women in the trial. In the exploratory evaluation, mothers will be registered on the USSD platform utilizing their existing mobile phone and Subscriber Identity Module (SIM); their own number or otherwise one identified by the mother as her main contact number. Mothers will receive a series of periodic messages pertaining to appointment schedules, treatment adherence and reporting of adverse events. Participant data shall be secured through relevant data security standards

of the information and communications technology regulator, and with the mobile network service provider. Data shall only be accessible by research staff.

16.6.8 Performance of a highly sensitive diagnostic test for malaria

Collaborating institutions and contact persons: Christine Bachman, from Intellectual Ventures Limited, Washington, USA; and Dr Gonzalo Domingo, from PATH, Seattle, Washington, USA, and Dr Emily Adams, LSTM, UK

Objective: To evaluate the diagnostic performance of novel diagnostic devices and highly sensitive diagnostic test for malaria in pregnant women.

Rationale: Current RDTs have limited sensitivity to detect placental malaria in asymptomatic pregnant women. New diagnostic tests are under development with lower limits of detection, including, but not limited to, automated readers of RDTs and an experimental, highly-sensitive RDT (HSRDT). We will determine the diagnostic performance of new diagnostic tests in samples taken from the pregnant women included in the trial and compare them against the PCR results obtained in the trial as a reference standard. Since these new diagnostics tests are still experimental they can be used for research only, but not yet for clinical care.

Methods: The RDT and HSRDT will be run on site on a 5 to 100 microlitre taken from the blood samples collected from participants. The RDTs will be read visually and also by placing the cartridge into automated readers. The results from the RDT Reader will be blinded to the health care worker and because these involve proto-types they cannot be used for patient care.

Also, a 75 microlitre aliquot of blood will be stored frozen in cryovials for a later multiplex quantitative ELISA (Q-ELISA) and standard RDTs (not for patient care) at PATH (Seattle, USA). The Q-ELISA assay contains spots for HRP2 and pan-malaria LDH. The capture and detection of antibodies are orthologous to those on the RDTs and therefore allow confirmation of antigenemia. All experimental assays shall be conducted for research purposes only within the clinical setting of the trial but will not inform care.

A total of 1,500 samples will be tested using the HSRDT and Q-Elisa providing 0.06 confidence interval at 0.95 confidence level for a 90% expected sensitivity, and a 1,500 sample for the comparison against the developmental readers. The primary outcome will be the sensitivity of the various diagnostic methods compared to qPCR or standard RDTs, including the threshold of detection of the experimental assays. Secondary outcomes will include other standard measures of diagnostic accuracy (specificity, positive predictive value, and negative predictive value) against PCR positivity.

16.7 Appendix VII. Product characteristics

16.7.1 Dihydroartemisinin-piperaquine product insert (D-ARTEPP, Guilin Pharma)

PATIENT INFORMATION LEAFLET: INFORMATION FOR THE USER

D-ARTEPP®

Dihydroartemisinin / Piperaquine phosphate

FOSUN PHARMA

Read all of this leaflet carefully before you start using this medicine
- Keep this leaflet. You may need to read it again.
- If you have any further questions, ask your doctor or pharmacist.
- This medicine has been prescribed for you. Do not pass it on to others. It may harm them, even if their symptoms are the same as yours.
- If any of the side effects gets serious, or if you notice any side effects not listed in this leaflet, please tell your doctor or pharmacist.

In this leaflet:
1 . What D-ARTEPP is and what it is used for
2 . Before you or your child takes D-ARTEPP
3 . How to take D-ARTEPP
4 . Possible side effects
5 . How to store D-ARTEPP
6 . Further information

1 . WHAT D-ARTEPP IS AND WHAT IT IS USED FOR
D-ARTEPP contains the ingredients piperaquine phosphate and dihydroartemisinin. It is used to treat uncomplicated malaria when use of a medicine given by mouth is appropriate.
Malaria is caused by infection with a parasite called *Plasmodium*, spread by the bite of an infected mosquito. There are different types of *Plasmodium* parasite. D-ARTEPP kills the *Plasmodium falciparum* parasite.
The medicine can be taken by adults, children and infants over 6 months old who weigh 5 kilograms or more.

2 . BEFORE YOU OR YOUR CHILD TAKE D-ARTEPP
Do not take D-ARTEPP if you or your child:
- is allergic (hypersensitive) to the active substances, piperaquine tetraphosphate or dihydroartemisinin, or to any of the other ingredients of D-ARTEPP (see section 6 for a list of these);
- has a severe type of malaria infection which has affected parts of your body such as the brain, lungs or kidneys;
- has a heart condition, such as changes to the rhythm or rate of your heart beat, or heart disease;
- knows that any member of your family (parents, grandparents, brothers or sisters) died suddenly due to a heart problem or was born with heart problems;
- suffers from changes to the levels of salts in your body (electrolyte imbalances);
- is taking other medicines that can have an effect on heart rhythm, such as:
- quinidine, disopyramide, procainamide, amiodarone, dofetilide, ibutilide, hydroquinidine or sotalol;
- medicines used to treat depression;
- medicines used to treat mental health problems such as phenothiazines, sertindole, sultopride, chlorpromazine, haloperidol, mesoridazine, pimozide, or thioridazine;
- medicines used to treat infections. These include some of the types of medicines used to treat bacterial infections (macrolides [such as erythromycin or clarithromycin] and fluoroquinolones [such as moxifloxacin and sparfloxacin]) or fungal infections (including fluconazole and imidazole) as well as pentamidine (used to treat a specific type of pneumonia) and saquinavir (for treatment of HIV);
- antihistamines used to treat allergies or inflammation such as terfenadine, astemizole or mizolastine;
- certain medicines used to treat stomach problems such as cisapride, domperidone or droperidol;
- other medicines such as vinca alkaloids and arsenic trioxide (used to treat certain cancers), bepridil (used to treat angina), diphemanil (used to treat stomach disturbances), levomethadyl and methadone (used to treat drug addiction), and probucol (used to treat high blood cholesterol levels).
- has recently (for example within about one month) been treated for malaria with certain medicines or has taken certain medicines to prevent malaria. These medicines include: mefloquine, halofantrine, lumefantrine, chloroquine or quinine

If any of the above applies to you or your child or if you are unsure, tell your doctor or pharmacist before taking or giving D-ARTEPP.

Take special care with D-ARTEPP
Check with your doctor or pharmacist before taking this medicine if you or your child:
- has liver or kidney problems;
- has a malaria infection caused by a parasite other than *Plasmodium falciparum;*
- is taking or has taken any other medicines for the treatment of malaria (other than those mentioned above);
- is pregnant or breastfeeding (see below);
- is female, elderly (over 65 years) or vomiting;
- is taking certain other medicines which could cause possible metabolic interactions. Examples are listed in the section "Taking other medicines".

If you are not sure about any of the above, please ask your doctor or pharmacist.

Use in children
Do not give this medicine to infants under 6 months or below 5 kg in weight.

Taking other medicines
Please tell your doctor or pharmacist if you or your child is taking or has recently taken any other medicines, including medicines obtained without a prescription. Some medicines can affect the way D-ARTEPP works and your doctor may decide that D-ARTEPP is not suitable or that extra checks are needed while you or your child is taking the medicinal products which could cause possible interactions. Examples are listed below (but there are several others):
- some medicines used to treat high cholesterol in the blood (such as atorvastatin, lovastatin, simvastatin);
- medicines used to treat hypertension and heart problems (such as diltiazem, nifedipine, nitrendipine, verapamil, felodipine, amlodipine);
- some medicines used to treat HIV (antiretroviral medicinal products): protease inhibitors (such as amprenavir, atazanavir, indinavir, nelfinavir, ritonavir), non-nucleoside reverse transcriptase inhibitors (such as efavirenz, nevirapine);
- some medicines used to treat microbial infections (such as telithromycin, rifampicin, dapsone);
- medicines used to help you fall asleep: benzodiazepines (such as midazolam, triazolam, diazepam, alprazolam), zaleplon, zolpidem;
- medicines used to prevent/treat epileptic seizures: barbiturates (such as phenobarbital), carbamazepine or phenytoin;
- medicines used after organ transplantation and in autoimmune diseases (such as cyclosporin,

Body weight(kg)	Daily dose (mg)	Total number of tablets
5 to less than 8	Half 40 / 320 mg tablet a day	1.5
8 to less than 11	Half 60/480mg tablet a day	1.5
11 to less than 17	One 40 / 320 mg tablet a day	3
17 to less than 25	One and half 40/ 320 mg tablets a day	4.5
25 to less than 36	Two 40 / 320 mg tablets a day	6
36 to less than 60	Two 60/480mg tablets a day	6
60 to less than 80	Two 80 / 640 mg tablets a day	6
over 80	Two and half 80 / 640 mg tablets a day	7.5

If you weigh more than 100 kg then follow the dose that your doctor has prescribed.

Vomiting when taking this medicine
If this happens within:
- 30 minutes of taking D-ARTEPP, the whole dose must be taken again.
- 31-60 minutes, half the dose must be taken again.

If you or your child vomits also the second dose, do not take or give your child another dose. Contact your doctor urgently to obtain an alternative treatment for malaria.

Taking this medicine, if the malaria infection returns
If you or your child gets another attack of malaria you may take a second course of D-ARTEPP if your doctor thinks this is a suitable treatment. You or your child should not take a second course of D-ARTEPP within 2 months of the last course.

If you or your child takes more D-ARTEPP tablets than you should
If you or your child takes more than the recommended dose, tell your doctor. Your doctor may suggest special monitoring for you or your child because doses higher than those recommended may have an unwanted, severe effect on your heart (see also section 4).

If you or your child forgets to take D-ARTEPP
If you or your child forgets to take the second dose of D-ARTEPP at the right time, take it as soon as you remember. Then take the third (last) dose approximately 24 hours after the second dose.
If you or your child forgets to take the third (last) dose at the right time, take it as soon as you remember.
Never take more than one dose on the same day to make up for a missed dose.
Check with your doctor or pharmacist if you are not sure.

If you or your child stops taking D-ARTEPP
For the medicine to work effectively, you or your child should take the tablets as instructed and should complete the 3 days course of treatment. If you or your child is not able to do this, talk to your doctor or pharmacist.
If you have any further questions on the use of this medicine, ask your doctor or pharmacist.

4 . POSSIBLE SIDE EFFECTS
Like all medicines, D-ARTEPP can cause side effects, although not everybody gets them. Most of the side effects are not severe and normally disappear within a few days or weeks after treatment.
If you or your child gets a rash, swelling of the face, lips, tongue or throat with difficulty in swallowing or breathing, these may be signs of an allergic reaction. Tell your doctor immediately, or go immediately to the emergency department of your nearest hospital, taking this leaflet with you.
A heart problem, called QT prolongation, can occur while taking D-ARTEPP and for some days after taking the last dose. This can cause a life-threatening abnormality of the heart rhythm.
Your doctor may take electrical recordings of your heart (electrocardiogram, ECG) while you are being treated and after the last dose is given. Your doctor will advise you when these readings will be taken.
If you notice anything different about your heart rhythm or have symptoms (such as palpitations or irregular heart beat) you should contact your doctor as soon as possible and before the next dose is due.

Side effects in adults
Common (affecting less than 1 in 10 patients but more than 1 in 100)
Anaemia, headache, heart rhythm disturbances (ECG changes or noticing unusually fast heart beats or palpitations), fever, general weakness.
Uncommon (affecting less than 1 in 100 patients but more than 1 in 1000)
Influenza, respiratory infections, poor appetite or loss of appetite, dizziness, convulsions (fits), irregular or slow heart rate, cough, vomiting, abdominal pain, diarrhoea, nausea, inflammation or enlargement of the liver, abnormal liver function tests, itching, pain in the muscles or joints.

Side effects in children
Very common (affecting more than 1 in 10 patients)
Influenza, cough, fever.
Common (affecting less than 1 in 10 patients but more than 1 in 100)
Respiratory infections, ear infection, anaemia, abnormalities in various types of blood cells (white blood cells and platelets), poor appetite or loss of appetite, eye infection, heart rhythm disturbances (change as in adults, ECG changes), abdominal pain, vomiting, diarrhoea, skin inflammation, rash, general weakness.
Uncommon (affecting less than 1 in 100 patients but more than 1 in 1000)
Abnormalities in red blood cells, excessive numbers of platelets, enlargement of some organs (such as liver or spleen), swollen lymph glands, convulsions (fits), headache, abnormal heart sounds (heard by your doctor with a stethoscope), nose bleeds, runny nose, nausea, inflammation of the mouth, inflammation or enlargement of the liver, jaundice, abnormal liver function blood tests, skin itching and inflammation, pain in the joints.
If any of the side effects gets serious, or if you notice any side effects not listed in this leaflet, please tell your doctor or pharmacist.

5 . HOW TO STORE D-ARTEPP
Keep D-ARTEPP tablets out of the reach and sight of children.
Do not take D-ARTEPP after the expiry date which is stated on the package after 'EXP'. The expiry date refers to the last day of that month.
Stored in tightly closed containers, protected from light, not above 25℃.

tacrolimus);
- sex hormones, including those contained in hormonal contraceptives (such as gestodene, progesterone, estradiol), testosterone;
- glucocorticoids (hydrocortisone, dexamethasone);
- omeprazole (used to treat diseases related to gastric acid production);
- paracetamol (used to treat pain and fever);
- theophylline (used to improve bronchial air flow);
- nefazodone (used to treat depression);
- aprepitant (used to treat nausea);
- some gases (such as enflurane, halothane and isoflurane) used to give a general anaesthetic.

Taking D-ARTEPP without food and drink

You should take D-ARTEPP tablets with water only.

You should take this medicine on an empty stomach. You should take each dose no less than 3 hours after the last food intake, and no food should be taken within 3 hours after each dose of D-ARTEPP.

You can drink water at any time. You should not take D-ARTEPP with grapefruit juice due to possible interactions.

Pregnancy and breast-feeding

Tell your doctor if you are pregnant, think you may be pregnant or become pregnant, or if you are breast-feeding.

D-ARTEPP must not be used in pregnancy if your doctor can give you an alternative medicine. If you receive D-ARTEPP while pregnant, please note that a pregnancy registry is in place to monitor the pregnancy outcomes.

You should not breast-feed your baby while taking this medicine.

If you are taking folate supplements to prevent possible neural tube birth defects, you can continue taking them at the same time as D-ARTEPP.

Ask your doctor or pharmacist for advice before taking any medicine during pregnancy or breast- feeding.

Driving and using machines

You can drive or use machines after taking D-ARTEPP.

3. HOW TO TAKE D-ARTEPP

Always take D-ARTEPP exactly as your doctor has told you to. You should check with your doctor or pharmacist if you are not sure.

Take this medicine with water and on an empty stomach. You or your child should take each dose at least 3 hours after your last meal. You should also avoid eating until 3 hours after taking D-ARTEPP. You can drink water at any time.

If the tablets are difficult to swallow, you can crush and mix them with water; drink the mixture immediately.

A course of D-ARTEPP lasts 3 consecutive days. Take one dose on each day. You should try to take the dose at about the same time on each of the three days.

The daily dose depends on the patient's **body weight.** Your doctor should have prescribed a dose that is appropriate for your weight or your child's weight as follows:

Do not use D-ARTEPP if you notice the blister package is open.

Medicines should not be disposed of via wastewater or household waste. Ask your pharmacist how to dispose of medicines no longer required. These measures will help to protect the environment.

6 . FURTHER INFORMATION

What D-ARTEPP contains

Each film-coated tablet contains 40 mg dihydroartemisinin and 320 mg Anhydrous piperaquine phosphate.

Each film-coated tablet contains 60 mg dihydroartemisinin and 480 mg Anhydrous piperaquine phosphate.

Each film-coated tablet contains 80 mg dihydroartemisinin and 640 mg Anhydrous piperaquine phosphate.

The other ingredients are:

Tablet core: Pregelatinized starch, Dextrin, Hypromellose, Croscarmellose sodium, Magnesium stearate.

Film coating: Titanium dioxide, Macrogol/Polyethylene glycol, Polyvinyl alcohol, Talc, FD& C Blue#2 indigo carmine aluminum lake.

What D-ARTEPP looks like and contents of the pack

D-ARTEPP are blue film-coated tablets, embossed and with a break line along the middle on one side.

The 40mg/320mg tablets, 9 tablets packaged in a Polyamide/Aluminum foil/Polyvinyl Chloride-Aluminum blister. Carton box containing 25 blister per pack.

The 60mg/480mg tablets, 6 tablets packaged in a Polyamide/Aluminum foil/Polyvinyl Chloride-Aluminum blister. Carton box containing 25 blister per pack.

The 80mg/640mg tablets, 6 tablets packaged in a Polyamide/Aluminum foil/Polyvinyl Chloride-Aluminum blister. Carton box containing 25 blister per pack.

Manufacturer

Name: Guilin Pharmaceutical Co.,Ltd.
Address: No. 43 Qilidian Road, Guilin 541004, Guangxi, China
Tel: +86 773 3841973
Fax: +86 773 3841973
Site Web: http://www.guilinpharma.com

Distributor

Guilin Pharmaceutical (Shanghai) Co.,Ltd.

This leaflet was last approved in 10/2018

4.2.04.20.027.01.02

16.7.2 Dihydroartemisinin-piperaquine product insert (Eurartesim, AlfaSigma)

PACKAGE LEAFLET: INFORMATION FOR THE USER

Eurartesim 320 mg/40 mg film-coated tablets
Piperaquine tetraphosphate/dihydroartemisinin

Read all of this leaflet carefully before you start using this medicine
- Keep this leaflet. You may need to read it again.
- If you have any further questions, ask your doctor or pharmacist.
- This medicine has been prescribed for you. Do not pass it on to others. It may harm them, even if their symptoms are the same as yours.
- If any of the side effects gets serious, or if you notice any side effects not listed in this leaflet, please tell your doctor or pharmacist.

In this leaflet:

1. What Eurartesim is and what it is used for
2. Before you or your child takes Eurartesim
3. How to take Eurartesim
4. Possible side effects
5. How to store Eurartesim
6. Further information

1. WHAT EURARTESIM IS AND WHAT IT IS USED FOR

Eurartesim contains the ingredients piperaquine tetraphosphate and dihydroartemisinin. It is used to treat uncomplicated malaria when use of a medicine given by mouth is appropriate.

Malaria is caused by infection with a parasite called *Plasmodium*, spread by the bite of an infected mosquito.There are different types of *Plasmodium* parasite. Eurartesim kills the *Plasmodium falciparum* parasite.

The medicine can be taken by adults, children and infants over 6 months old who weigh 5 kilograms or more.

2. BEFORE YOU OR YOUR CHILD TAKE EURARTESIM

Do not take Eurartesim if you or your child:
- is allergic (hypersensitive) to the active substances, piperaquine tetraphosphate or dihydroartemisinin, or to any of the other ingredients of Eurartesim (see section 6 for a list of these);
- has a severe type of malaria infection which has affected parts of your body such as the brain, lungs or kidneys;
- has a heart condition, such as changes to the rhythm or rate of your heart beat, or heart disease;
- knows that any member of your family (parents, grandparents, brothers or sisters) died suddenly due to a heart problem or was born with heart problems;
- suffers from changes to the levels of salts in your body (electrolyte imbalances);
- is taking other medicines that can have an effect on heart rhythm, such as:
  - quinidine, disopyramide, procainamide, amiodarone, dofetilide, ibutilide, hydroquinidine or sotalol;
  - medicines used to treat depression;
  - medicines used to treat mental health problems such as phenothiazines, sertindole, sultopride, chlorpromazine, haloperidol, mesoridazine, pimozide, or thioridazine;

- medicines used to treat infections. These include some of the types of medicines used to treat bacterial infections (macrolides [such as erythromycin or clarithromycin] and fluoroquinolones [such as moxifloxacin and sparfloxacin]) or fungal infections (including fluconazole and imidazole) as well as pentamidine (used to treat a specific type of pneumonia) and saquinavir (for treatment of HIV);
- antihistamines used to treat allergies or inflammation such as terfenadine, astemizole or mizolastine;
- certain medicines used to treat stomach problems such as cisapride, domperidone or droperidol;
- other medicines such as vinca alkaloids and arsenic trioxide (used to treat certain cancers), bepridil (used to treat angina), diphemanil (used to treat stomach disturbances), levomethadyl and methadone (used to treat drug addiction), and probucol (used to treat high blood cholesterol levels).

• has recently (for example within about one month) been treated for malaria with certain medicines or has taken certain medicines to prevent malaria. These medicines include: mefloquine, halofantrine, lumefantrine, chloroquine or quinine

If any of the above applies to you or your child or if you are unsure, tell your doctor or pharmacist before taking or giving Eurartesim.

Take special care with Eurartesim
Check with your doctor or pharmacist before taking this medicine if you or your child:

- has liver or kidney problems;
- has a malaria infection caused by a parasite other than *Plasmodium falciparum*;
- is taking or has taken any other medicines for the treatment of malaria (other than those mentioned above);
- is pregnant or breastfeeding (see below);
- is female, elderly (over 65 years) or vomiting;
- is taking certain other medicines which could cause possible metabolic interactions. Examples are listed in the section "Taking other medicines".

If you are not sure about any of the above, please ask your doctor or pharmacist.

Use in children
Do not give this medicine to infants under 6 months or below 5 kg in weight.

Taking other medicines
Please tell your doctor or pharmacist if you or your child is taking or has recently taken any other medicines, including medicines obtained without a prescription. Some medicines can affect the way Eurartesim works and your doctor may decide that Eurartesim is not suitable or that extra checks are needed while you or your child is taking the medicinal products which could cause possible interactions. Examples are listed below (but there are several others):

- some medicines used to treat high cholesterol in the blood (such as atorvastatin, lovastatin, simvastatin);
- medicines used to treat hypertension and heart problems (such as diltiazem, nifedipine, nitrendipine, verapamil, felodipine, amlodipine);
- some medicines used to treat HIV (antiretroviral medicinal products): protease inhibitors (such as amprenavir, atazanavir, indinavir, nelfinavir, ritonavir), non-nucleoside reverse transcriptase inhibitors (such as efavirenz, nevirapine);
- some medicines used to treat microbial infections (such as telithromycin, rifampicin, dapsone);
- medicines used to help you fall asleep: benzodiazepines (such as midazolam, triazolam, diazepam, alprazolam), zaleplon, zolpidem;
- medicines used to prevent/treat epileptic seizures: barbiturates (such as phenobarbital), carbamazepine or phenytoin;
- medicines used after organ transplantation and in autoimmune diseases (such as cyclosporin, tacrolimus);

- sex hormones, including those contained in hormonal contraceptives (such as gestodene, progesterone, estradiol), testosterone;
- glucocorticoids (hydrocortisone, dexamethasone);
- omeprazole (used to treat diseases related to gastric acid production);
- paracetamol (used to treat pain and fever);
- theophylline (used to improve bronchial air flow);
- nefazodone (used to treat depression);
- aprepitant (used to treat nausea);
- some gases (such as enflurane, halothane and isoflurane) used to give a general anaesthetic.

Taking Eurartesim without food and drink
You should take Eurartesim tablets with water only.
You should take this medicine on an empty stomach. You should take each dose no less than 3 hours after the last food intake, and no food should be taken within 3 hours after each dose of Eurartesim. You can drink water at any time. You should not take Eurartesim with grapefruit juice due to possible interactions.

Pregnancy and breast-feeding
Tell your doctor if you are pregnant, think you may be pregnant or become pregnant, or if you are breast-feeding.

Eurartesim must not be used in pregnancy if your doctor can give you an alternative medicine. If you receive Eurartesim while pregnant, please note that a pregnancy registry is in place to monitor the pregnancy outcomes.

You should not breast-feed your baby while taking this medicine.

If you are taking folate supplements to prevent possible neural tube birth defects, you can continue taking them at the same time as Eurartesim.

Ask your doctor or pharmacist for advice before taking any medicine during pregnancy or breast-feeding.

Driving and using machines
You can drive or use machines after taking Eurartesim.

3. HOW TO TAKE EURARTESIM

Always take Eurartesim exactly as your doctor has told you to. You should check with your doctor or pharmacist if you are not sure.

Take this medicine with water and on an empty stomach. You or your child should take each dose at least 3 hours after your last meal. You should also avoid eating until 3 hours after taking Eurartesim. You can drink water at any time.
If the tablets are difficult to swallow, you can crush and mix them with water; drink the mixture immediately.

A course of Eurartesim lasts 3 consecutive days. Take one dose on each day. You should try to take the dose at about the same time on each of the three days.

The daily dose depends on the patient's **body weight**. Your doctor should have prescribed a dose that is appropriate for your weight or your child's weight as follows:

Body weight (kg)	Daily dose (mg)	Total number of tablets for treatment
5 to less than 7	Half 160 mg/20 mg tablet a day	1.5 tablet
7 to less than 13	One 160 mg/20 mg tablet a day	3 tablets
13 to less than 24	One 320 mg/40 mg tablet a day	3 tablets
24 to less than 36	Two 320 mg/40 mg tablets a day	6 tablets
36 to less than 75	Three 320 mg/40 mg tablets a day	9 tablets
75 to 100	Four 320 mg/40 mg tablets a day	12 tablets

If you weigh more than 100 kg then follow the dose that your doctor has prescribed.

Vomiting when taking this medicine
If this happens within:

- 30 minutes of taking Eurartesim, the whole dose must be taken again.
- 31-60 minutes, half the dose must be taken again.

If you or your child vomit also the second dose, do not take or give your child another dose. Contact your doctor urgently to obtain an alternative treatment for malaria.

Taking this medicine, if the malaria infection returns

- If you or your child gets another attack of malaria you may take a second course of Eurartesim within one year if your doctor thinks this is a suitable treatment. You or your child must not take more than two courses within one year. If this happens, talk to your doctor. You or your child should not take a second course of Eurartesim within 2 months of the first course.
- If you or your child is infected more than twice in a year, your doctor will prescribe an alternative treatment.

If you or your child takes more Eurartesim tablets than you should
If you or your child takes more than the recommended dose, tell your doctor. Your doctor may suggest special monitoring for you or your child because doses higher than those recommended may have an unwanted, severe effect on your heart (see also section 4).

If you or your child forgets to take Eurartesim
If you or your child forgets to take the second dose of Eurartesim at the right time, take it as soon as you remember. Then take the third (last) dose approximately 24 hours after the second dose.
If you or your child forgets to take the third (last) dose at the right time, take it as soon as you remember.
Never take more than one dose on the same day to make up for a missed dose.
Check with your doctor or pharmacist if you are not sure.

If you or your child stops taking Eurartesim
For the medicine to work effectively, you or your child should take the tablets as instructed and should complete the 3 days course of treatment. If you or your child is not able to do this, talk to your doctor or pharmacist.

If you have any further questions on the use of this medicine, ask your doctor or pharmacist.

4. POSSIBLE SIDE EFFECTS

Like all medicines, Eurartesim can cause side effects, although not everybody gets them. Most of the side effects are not severe and normally disappear within a few days or weeks after treatment.

Do not use Eurartesim if you notice the blister package is open.

Medicines should not be disposed of via wastewater or household waste. Ask your pharmacist how to dispose of medicines no longer required. These measures will help to protect the environment.

6. FURTHER INFORMATION

What Eurartesim contains
Each film-coated tablet contains 320 mg piperaquine tetraphosphate (as the tetrahydrate) and 40 mg dihydroartemisinin.

The other ingredients are:
Tablet core: pre-gelatinised starch, dextrin, hypromellose (E464), croscarmellose sodium, magnesium stearate (E572).
Film coating: hypromellose, titanium dioxide (E171), macrogol 400.

What Eurartesim looks like and contents of the pack
Eurartesim are white film-coated tablets, embossed and with a break line along the middle.

The 320 mg/40 mg tablets have two 'σ' letters on one side and come in blister strips containing 3, 6, 9 or 12 tablets.

Marketing Authorisation Holder

Sigma-Tau Industrie Farmaceutiche Riunite s.p.a.
Viale Shakespeare 47
00144 Rome
Italy

Tel: +39 06 5926443
Fax: +39 06 5926600
Email: sigmatauinfo@sigma-tau.it

Manufacturer

Sigma-Tau Industrie Farmaceutiche Riunite s.p.a.
Via Pontina km. 30,400
00040 Pomezia (Rome)
Italy

Tel: +39 06 91391
Fax: +39 06 91166976
Email: sigmatauinfo@sigma-tau.it

For any information about this medicine, please contact the local representative of the Marketing Authorisation Holder:

België/Belgique/Belgien
MPCA bvba
Tél/Tel: + 32 9 344 53 38

Luxembourg/Luxemburg
MPCA bvba
Belgique/Belgien
Tél/Tel: + 32 9 344 53 38

Nederland
Sigma-Tau BV
Tel: +31 30 6702020
info@sigma-tau.nl

Deutschland
Sigma-Tau Arzneimittel GmbH
Tel.: +49 211 687717 0
info@sigma-tau.de

España
Sigma-Tau España, S.A.
Tel: +34 91 888 36 00
sigmatau@sigma-tau.es

France
Sigma-Tau France
Tél: + 33 1 45 21 02.69

Ireland
Sigma-Tau Pharma Limited UK
United Kingdom
Tel: +44 (0) 8000431268
medical.information@sigma-tau.co.uk

Portugal
Defiante Farmacêutica, S.A.
Tel: +351 291 214 090
info@defiante.com

United Kingdom
Sigma-Tau Pharma Limited UK
Tel: +44 (0) 8000431268
medical.information@sigma-tau.co.uk

България, Magyarország, Ceská republika, Malta, Danmark, Norge, Eesti, Österreich, Ελλάδα, Polska, România, Slovenija, Ísland, Slovenská republika, Italia, Suomi/Finland, Κύπρος, Sverige,Latvija, Lietuva

Sigma-Tau Industrie Farmaceutiche Riunite s.p.a.
Италия, Olaszország, Itàlie, Italja, Italien, Italia, Itaalia, Ιταλία, Włochy, Italija, Ítalía, taliansko, Itālija
Тел/Tel/Tlf/Τηλ/Sími/Puh: +39 06 9139.3796
Daniela.Campanelli@sigma-tau.it

This leaflet was last revised in

Detailed information on this medicine is available on the European Medicines Agency web site: http://www.ema.europa.eu

#

16.7.3 Cotrimoxazole

Package Leaflet: Information for the User

Co-trimoxazole 160 mg/800 mg Forte Tablets

This medicine is available as the above name but will be referred to as Co-trimoxazole throughout the following leaflet.

Read all of this leaflet carefully before you start taking this medicine because it contains important information for you.

- Keep this leaflet. You may need to read it again.
- If you have any further questions, ask your doctor or pharmacist.
- This medicine has been prescribed for you only. Do not pass it on to others. It may harm them, even if their signs of illness are the same as yours.
- If you get any side effects, talk to your doctor or pharmacist. This includes any possible side effects not listed in this leaflet. See section 4.

What is in this leaflet:

1. What Co-trimoxazole is and what it is used for
2. What you need to know before you take Co-trimoxazole
3. How to take Co-trimoxazole
4. Possible side effects
5. How to store Co-trimoxazole
6. Contents of the pack and other information

1. What Co-trimoxazole is and what it is used for

Co-trimoxazole is a combination of two different antibiotics called sulfamethoxazole and trimethoprim, which is used to treat infections caused by certain bacteria. Like all antibiotics, Co-trimoxazole only works against some types of bacteria. This means that it is only suitable for treating some types of infections. Co-trimoxazole can be used to treat or prevent:

- Lung infections (pneumonia or PJP) caused by a bacteria called *Pneumocystis jiroveci* (previously known as *Pneumocystis carinii* or PCP).
- Infections caused by a bacteria called Toxoplasma (toxoplasmosis).

Co-trimoxazole can be used to treat:

- Urinary bladder or urinary tract infections (water infections)
- Respiratory tract infections such as bronchitis
- Ear infections such as otitis media
- An infection called nocardiosis which can affect the lungs, skin and brain.

2. What you need to know before you take Co-trimoxazole

Do not take Co-trimoxazole if:

- You are allergic (hypersensitive) to sulfamethoxazole, trimethoprim or co-trimoxazole or any of the other ingredients of Co-trimoxazole (see section 6: Contents of the pack and other information).
- You are allergic to sulphonamide medicines. Examples include sulphonylureas (such as gliclazide and glibenclamide) or thiazide diuretics (such as bendroflumethiazide - a water tablet).
- You have severe liver or kidney problems.
- You have ever had a problem with your blood causing bruises or bleeding (thrombocytopenia).
- You have been told that you have a rare blood problem called porphyria, which can affect your skin or nervous system.

If it is for your child, Co-trimoxazole should not be given if they are less than 6 weeks old or were premature unless it is for the treatment or prevention of PJP. In this case, Co-trimoxazole should not be given if they are less than 4 weeks old.

If you are not sure if any of the above apply to you, talk to your doctor or pharmacist before taking Co-trimoxazole.

Warnings and precautions

Talk to your doctor or pharmacist before taking Co-trimoxazole:

- If you have severe allergies or asthma.
- Potentially life-threatening skin rashes (Stevens-Johnson syndrome, toxic epidermal necrolysis) have been reported with the use of Co-trimoxazole appearing initially as reddish target-like spots or circular patches often with central blisters on the trunk.
- Additional signs to look for include ulcers in the mouth, throat, nose, genitals and conjunctivitis (red and swollen eyes).
- These potentially life-threatening skin rashes are often accompanied by flu-like symptoms. The rash may progress to widespread blistering or peeling of the skin.
- The highest risk for occurrence of serious skin reactions is within the first weeks of treatment.
- If you have developed Stevens-Johnson syndrome or toxic epidermal necrolysis with the use of Co-trimoxazole you must not be re-started on Co-trimoxazole at any time.
- If you develop a rash or these skin symptoms, stop taking Co-trimoxazole, seek urgent advice from a doctor and tell him that you are taking this medicine.
- If you have been told that you are at risk for a rare blood disorder called porphyria.
- If you have a kidney disease.
- If you don't have enough folic acid (a vitamin) in your body – which can make your skin pale and make you feel tired, weak and breathless. This is known as anaemia.
- If you have a disease called glucose-6-phosphate dehydrogenase deficiency, which can cause jaundice or spontaneous destruction of red blood cells. If you have a problem with your metabolism called phenylketonuria and are not on a special diet to help your condition.
- If you are elderly.
- If you are underweight or malnourished.
- If you have been told by your doctor that you have a lot of potassium in your blood.
- If you have a severe blood disorder, such as a low number of red blood cells (anaemia), a low number of white blood cells (leucopenia) or a low number of platelets, which may cause bleeding and bruising (thrombocytopenia).

Other medicines and Co-trimoxazole

Tell your doctor or pharmacist if you are taking, have recently taken or may take any other medicines. This is because Co-trimoxazole can affect the way some medicines work. Also some other medicines can affect the way Co-trimoxazole works.

In particular tell your doctor or pharmacist if you are taking any of the following medicines:

- Diuretics (water tablets), which help increase the amount of urine you produce.
- Pyrimethamine, used to treat and prevent malaria, and to treat diarrhoea.
- Ciclosporin, used after organ transplant surgeries.
- Blood thinners such as warfarin.
- Phenytoin, used to treat epilepsy (fits).
- Medicines used to treat diabetes, such as glibenclamide, glipizide or tolbutamide (sulphonylureas) and repaglinide.
- Rifampicin, an antibiotic.
- Medicines to treat problems with the way your heart beats such as digoxin or procainamide.
- Amantadine, used to treat Parkinson's disease, multiple sclerosis, 'flu or shingles.
- Medicines to treat HIV (Human Immunodeficiency Virus), called zidovudine or lamivudine.
- Medicines that can increase the amount of potassium in your blood, such as diuretics (water tablets, which help increase the amount of urine you produce), steroids (like prednisolone) and digoxin.
- Methotrexate, a medicine used to treat certain cancers or certain diseases affecting your immune system.
- Folinic acid.
- Contraceptive medicines.

Co-trimoxazole with food and drink

You should take Co-trimoxazole with some food or drink. This will stop you feeling sick (nausea) or having diarrhoea. Although it is better to take it with food, you can still take it on an empty stomach.
Drink plenty of fluid such as water while you are taking Co-trimoxazole.

Pregnancy and breast-feeding

If you are pregnant or breast-feeding, think you may be pregnant or are planning to have a baby, ask your doctor or pharmacist for advice before taking this medicine.

Driving and using machines

Effects on the ability to drive and operate machinery in patients taking this medicine have not been studied.

3. How to take Co-trimoxazole

Always take this medicine exactly as your doctor has told you. Check with your doctor or pharmacist if you are not sure.

Usual Dose
Adults and children over 12 years
- The usual dose is one tablet in a morning and one tablet in an evening.
- Co-trimoxazole should be taken for at least five days.
- Make sure that you finish the course of Co-trimoxazole which your doctor has prescribed.

Co-trimoxazole is not usually given to children under 12 years old. If they have been given to your child under 12 years please speak to your doctor or pharmacist for more information.

Special Dose
The dose of Co-trimoxazole and how long you need to take it depends on the infection you have and how bad it is. Your doctor may prescribe you a different dose or length of course of Co-trimoxazole to:
- Treat urinary tract (water) infections.
- Treat and prevent lung infections caused by the bacteria *Pneumocystis jiroveci*.
- Treat infections caused by the bacteria Toxoplasma (toxoplasmosis) or Nocardia (nocardiosis).

If you have kidney problems your doctor may:
- Prescribe a lower dose of Co-trimoxazole.
- Take blood to test whether the medicine is working properly.

If you take Co-trimoxazole for a long time your doctor may:
- Take blood to test whether the medicine is working properly.
- Prescribe folic acid (a vitamin) for you to take at the same time as Co-trimoxazole.

If you take more Co-trimoxazole than you should
If you take more Co-trimoxazole than you should, talk to your doctor or go to a hospital straight away. Take the medicine pack with you. If you have taken too much Co-trimoxazole you may:
- Feel or be sick.
- Feel dizzy or confused.

If you forget to take Co-trimoxazole
- If you forget to take a dose, take it as soon as you remember it.
- Do not take a double dose to make up for the forgotten dose.

4. Possible side effects

Like all medicines Co-trimoxazole can cause side effects, although not everybody gets them. You may experience the following side effects with this medicine.

Stop taking Co-trimoxazole and tell your doctor immediately if you have an allergic reaction. Chances of an allergic reaction is very rare (fewer than 1 in 10,000 people are affected), signs of an allergic reaction include:

Allergic reactions
- Difficulty in breathing
- Fainting
- Swelling of face
- Swelling of mouth, tongue or throat which may be red and painful and/or cause difficulty in swallowing
- Chest pain
- Red patches on the skin

Very Common (more than 1 in 10 people)
- High levels of potassium in your blood, which can cause abnormal heart beats (palpitations)

Common (less than 1 in 10 people)
- A fungal infection called thrush or candidiasis which can affect your mouth or vagina
- Headache
- Feeling sick (nausea)
- Diarrhoea
- Skin rashes

Uncommon (less than 1 in 100)
- Being sick (vomiting)

Very Rare (less than 1 in 10,000 people)
- Fever (high temperature) or frequent infections
- Sudden wheeziness or difficulty breathing
- Potentially life-threatening skin rashes (Stevens-Johnson syndrome, toxic epidermal necrolysis) have been reported (see Warnings and precautions)
- Mouth ulcers, cold sores and ulcers or soreness of your tongue
- Skin lumps or hives (raised, red or white, itchy patches of skin)
- Blisters on your skin or inside your mouth, nose, vagina or bottom
- Inflammation of the eye which causes pain and redness
- The appearance of a rash or sunburn when you have been outside (even on a cloudy day)
- Low levels of sodium in your blood
- Changes in blood tests
- Feeling weak, tired or listless, pale skin (anaemia)
- Heart problems
- Jaundice (the skin and the whites of your eyes turn yellow). This can occur at the same time as unexpected bleeding or bruising
- Pains in your stomach, which can occur with blood in your faeces (stools)
- Pains in your chest, muscles or joints and muscle weakness
- Arthritis
- Problems with your urine. Difficulty passing urine. Passing more or less urine than usual.
- Blood or cloudiness in your urine
- Kidney problems
- Sudden headache or stiffness of your neck, accompanied by fever (high temperature)
- Problems controlling your movements
- Fits (convulsions or seizures)
- Feeling unsteady or giddy
- Ringing or other unusual sounds in your ears
- Tingling or numbness in your hands and feet
- Seeing strange or unusual sights (hallucinations)
- Depression
- Muscle pain and/or muscle weakness in HIV patients
- Loss of appetite

Reporting of side effects
If you get any side effects, talk to your doctor, pharmacist or nurse. This includes any possible side effects not listed in this leaflet. You can also report side effects directly via the Yellow Card Scheme at: www.mhra.gov.uk/yellowcard. By reporting side effects, you can help provide more information on the safety of this medicine.

5. How to store Co-trimoxazole

- Keep out of the sight and reach of children.
- Do not store above 25°C. Store in the original package.
- Do not take the tablets after the expiry date shown on the carton. The expiry date refers to the last day of that month.
- Medicines should not be disposed of via wastewater or household waste. Ask your pharmacist how to dispose of medicines no longer required. These measures will help protect the environment.
- If the tablets become discoloured or show any other signs of deterioration, you should seek the advice of your doctor or pharmacist, who will tell you what to do.

6. Contents of the pack and other information

What Co-trimoxazole contains
Co-trimoxazole is made up of two different medicines called sulfamethoxazole and trimethoprim.
Each tablet contains 160 mg of trimethoprim and 800 mg of sulfamethoxazole.
The other ingredients of Co-trimoxazole are: sodium starch glycollate (type A) (from potato starch), povidone K30, docusate sodium and magnesium stearate.

What Co-trimoxazole looks like and contents of the pack
Co-trimoxazole is a white, oval shaped, biconvex tablet scored in half on one side and plain on the other side.
Co-trimoxazole is supplied to you in a blister pack, containing 50 or 100 tablets.

PL: 15814/1215 **POM**

Manufactured by Alcala Farma, S.L, Ctra. M-300, Km 29,920 Alcala de Henares, 28802 Madrid, Spain **OR** Alcala Farma, S.L, Avenida de Madrid 82, Alcala de Henares, 28802 Madrid, Spain.
Procured from within the EU and repackaged by the Product Licence holder: O.P.D. Laboratories Ltd., Colonial Way, Watford, Herts WD24 4PR.

Leaflet revision and issue date (Ref.): 02.05.2017.

To request a copy of this leaflet in Braille, large print or audio please call 01923 332 796.

16.7.1 Fixed dose combination of TLD (TDF/3TC/DTG)

The summary of product characteristics for TLD is provided below using the package insert for TLD from Cipla is shown for illustrative purposes but other generic brands may be considered subject to local policy.

SCHEDULING STATUS: S4

PROPRIETARY NAME (AND DOSAGE FORM):

REYDIN (Film-coated tablets)

COMPOSITION:
Each **REYDIN** film-coated tablet contains:
Dolutegravir 50 mg (as dolutegravir sodium).
Lamivudine 300 mg.
Tenofovir disoproxil 300 mg (equivalent to tenofovir disoproxil fumarate).

Inactive ingredients: croscarmellose sodium, hydroxypropyl methyl cellulose, magnesium stearate, mannitol, microcrystalline cellulose, opadry blue (containing polyvinyl alcohol, talc, titanium dioxide, indigo carmine, glycerol esters of fatty acids, sodium lauryl sulfate, brilliant blue aluminium lake), povidone, sodium starch glycolate.

Contains mannitol 145 mg (sugar).

WARNING:
LACTIC ACIDOSIS AND SEVERE HEPATOMEGALY WITH STEATOSIS, INCLUDING FATAL CASES, HAVE BEEN REPORTED WITH THE USE OF NUCLEOSIDE ANALOGUES ALONE OR IN COMBINATION WITH OTHER ANTIRETROVIRALS (SEE "WARNINGS AND SPECIAL PRECAUTIONS"). REYDIN IS NOT INDICATED FOR TREATMENT OF CHRONIC HEPATITIS B VIRUS (HBV) INFECTION. THE SAFETY AND EFFICACY OF REYDIN HAS NOT BEEN ESTABLISHED IN PATIENTS CO-INFECTED WITH HBV AND HIV. SEVERE ACUTE EXACERBATIONS OF HEPATITIS B HAVE BEEN REPORTED IN PATIENTS WHO ARE CO-INFECTED WITH HBV AND HIV AND HAVE DISCONTINUED THE COMBINATION TABLET. HEPATIC FUNCTION SHOULD BE MONITORED CLOSELY WITH BOTH CLINICAL AND LABORATORY FOLLOW-UP FOR AT LEAST SEVERAL MONTHS IN PATIENTS WHO ARE CO-INFECTED WITH HIV AND HBV AND WHO DISCONTINUED THE COMBINATION TABLET. IF APPROPRIATE, INITIATION OF ANTI-HEPATITIS B THERAPY MAY BE WARRANTED (SEE "WARNINGS AND SPECIAL PRECAUTIONS").

PHARMACOLOGICAL CLASSIFICATION:
A 20.2.8 – Antimicrobial (chemotherapeutic) medicine. Other than antibiotics. Antiviral medicine.

PHARMACOLOGICAL ACTION:
Pharmacodynamic properties:
Dolutegravir:
Dolutegravir inhibits HIV integrase by binding to the integrase active sites and blocking the strand transfer step of retroviral deoxyribonucleic acid (DNA) integration which is essential for the HIV replication cycle. *In vitro*, dolutegravir dissociates slowly from the active site of the wild type integrase-DNA complex ($t_{1/2}$ 71 hours).

Resistance in vitro:
Isolation from wild type HIV-1: Viruses highly resistant to dolutegravir have not been observed during HIV-1 passage. During wild type HIV-1 passage in the presence of dolutegravir, integrase substitutions observed were S153Y and S153F with FCs ≤ 4,1 for strain IIIB, or E92Q with FC = 3,1 and G193E with FC = 3,2 for strain NL432. Additional passage of wildtype subtype B, C and A/G viruses in the presence of dolutegravir selected for R263K, G118R, and S153T.

Anti-HIV activity against resistant strains: Reverse transcriptase inhibitor (RTI)- and protease inhibitor (PI)-resistant strains: Dolutegravir demonstrated equivalent potency against 2 non-nucleoside (NN)-RTI-resistant, 3 nucleoside (N)-RTI-resistant and 2 PI-resistant HIV-1 mutant clones (1 triple and 1 sextuple) compared to the wild type strain.

Integrase inhibitor-resistant HIV-1 strains: Dolutegravir showed anti-HIV activity (susceptibility) with FC < 5 against 27 of 28 integrase inhibitor-resistant mutant viruses with single substitutions including T66A/I/K, E92Q/V, Y143C/H/R, Q148H/K/R, and N155H.

Integrase inhibitor-resistant HIV-2 strains: Site directed mutant HIV-2 viruses were constructed based on subjects infected with HIV-2 and treated with raltegravir who showed virologic failure. Overall the HIV-2 FCs observed were similar to HIV-1 FCs observed for similar pathway mutations.

Resistance in vivo:
Integrase inhibitor-naïve patients: No integrase inhibitor (INI) resistant mutations or treatment emergent resistance to the NRTI backbone therapy were isolated with dolutegravir 50 mg once daily in treatment-naïve studies.

Effects on renal function:
The effect of dolutegravir on serum creatinine clearance (CrCl), glomerular filtration rate (GFR) using iohexol as the probe and effective renal plasma flow (ERPF) using para-aminohippurate (PAH) as the probe was evaluated. A small decrease of 10 – 14 % in mean serum creatinine clearance (CrCl) was observed with dolutegravir within the first week of treatment. Dolutegravir has no significant effect on glomerular filtration rate (GFR) or the effective renal plasma flow (ERPF). *In vitro* studies suggest that the increases in creatinine observed in clinical studies are due to the non-pathologic inhibition of the organic cation transporter 2 (OCT2) in the proximal renal tubules, which mediates the tubular secretion of creatinine.

Lamivudine:
Lamivudine, a nucleoside reverse transcriptase inhibitor (NRTI), is a selective inhibitor of HIV-1 and HIV-2 replication *in vitro*. Lamivudine is metabolised intracellularly to the 5'-triphosphate which has an intracellular half-life of 16 – 19 hours. Lamivudine 5'-triphosphate is a weak inhibitor of the RNA- and DNA-dependent activities of HIV reverse transcriptase; its mode of action is a chain terminator of HIV reverse transcription.

Reduced *in vitro* sensitivity to lamivudine has been reported for HIV isolates from patients who have received lamivudine therapy. Lamivudine-resistant HIV-1 mutants are cross-resistant to didanosine and zalcitabine. In some patients treated with zidovudine plus didanosine or zalcitabine, isolates resistant to multiple reverse transcriptase inhibitors, including lamivudine, have emerged.

Lamivudine does not interfere with cellular deoxynucleotide metabolism and has little effect on mammalian cell and mitochondrial DNA content.

Tenofovir:
Tenofovir disoproxil fumarate, is an acyclic nucleoside phosphonate diester analogue of adenosine monophosphate and is converted *in vivo* to tenofovir. It is a nucleoside reverse transcriptase inhibitor.

Tenofovir is phosphorylated by cellular enzymes to form tenofovir diphosphate. Tenofovir diphosphate inhibits the activity of HIV-1 reverse transcriptase by competing with the natural substrate deoxyadenosine 5'-triphosphate and, after incorporation into DNA, by chain termination. Tenofovir diphosphate is a weak inhibitor of mammalian DNA polymerases α, β, and mitochondrial DNA polymerase γ.

Medicine resistance:
HIV-1 isolates with reduced susceptibility to tenofovir have been selected *in vitro* and a K65R mutation in reverse transcriptase have been selected *in vitro* and, in some patients, treated with tenofovir in combination with certain antiretroviral medicines. In treatment-naïve patients treated with tenofovir + lamivudine + efavirenz, viral isolates from 17 % of patients with virologic failure showed reduced susceptibility to tenofovir.

In treatment-experienced patients, some of the tenofovir-treated patients with virologic failure through week 96 showed reduced susceptibility to tenofovir. Genotypic analysis of the resistant isolates showed a mutation in the HIV-1 reverse transcriptase gene resulting in the K65R amino acid substitution.

Lamivudine:
Absorption:
Following oral administration, lamivudine is rapidly absorbed and extensively distributed. Absolute bioavailability in 12 adult subjects was 86 % ± 16 % (mean ± SD) for the 150 mg tablet and 87 % ± 13 % for the oral solution. After oral administration of 2 mg/kg twice a day to 9 adults with HIV-1, the peak serum lamivudine concentration (C_{max}) was 1,5 ± 0,5 µg/mL (mean ± SD). The area under the plasma concentration versus time curve (AUC) and C_{max} increased in proportion to oral dose over the range from 0,25 to 10 mg/kg. The accumulation ratio of lamivudine in HIV-1 positive asymptomatic adults with normal renal function was 1,50 following 15 days of oral administration of 2 mg/kg twice daily. The mean time (T_{max}) to maximum serum concentration (C_{max}) is about an hour.

Effects of food on oral absorption:
Lamivudine tablets and oral solution may be administered with or without food. An investigational 25 mg dosage form of lamivudine was administered orally to 12 asymptomatic, HIV-1 infected subjects on 2 occasions, once in the fasted state and once with food (1,099 kcal; 75 g fat, 34 g protein, 72 g carbohydrate). Absorption of lamivudine was slower in the fed state (T_{max}: 3,2 ± 1,3 hours) compared with the fasted state (T_{max}: 0,9 ± 0,3 hours); C_{max} in the fed state was 40 % ± 23 % (mean ± SD) lower than in the fasted state. There was no significant difference in systemic exposure (AUC_{∞}) in the fed and fasted states.

Distribution:
The apparent volume of distribution after IV administration of lamivudine to 20 subjects was 1,3 ± 0,4 L/kg, suggesting that lamivudine distributes into extravascular spaces. Volume of distribution was independent of dose and did not correlate with body weight.

Binding of lamivudine to human plasma proteins is low (less than 36 %). *In vitro* studies showed that over the concentration range of 0,1 to 100 µg/mL, the amount of lamivudine associated with erythrocytes ranged from 53 % to 57 % and was independent of concentration.

Metabolism:
Metabolism of lamivudine is a minor route of elimination. In humans, the only known metabolite of lamivudine is the trans-sulfoxide metabolite (approximately 5 % of an oral dose after 12 hours). Serum concentrations of this metabolite have not been determined. Lamivudine is not significantly metabolised by CYP450 enzymes.

Elimination:
The majority of lamivudine is eliminated unchanged in urine by active organic cationic secretion. In 9 healthy subjects given a single 300 mg oral dose of lamivudine, renal clearance was 199,7 ± 56,9 mL/min (mean ± SD). In 20 HIV-1 infected subjects given a single IV dose, renal clearance was 280,4 ± 75,2 mL/min (mean ± SD), representing 71 % ± 16 % (mean ± SD) of total clearance of lamivudine.

In most single-dose trials in HIV-1 infected subjects, HBV infected subjects, or healthy subjects with serum sampling for 24 hours after dosing, the observed mean elimination half-life (t½) ranged from 5 to 7 hours. In HIV-1 infected subjects, total clearance was 398,5 ± 69,1 mL/min (mean ± SD). Oral clearance and elimination half-life were independent of dose and body weight over an oral dosing range of 0,25 to 10 mg/kg.

No dose adjustment is needed when co-administered with food as lamivudine bioavailability is not altered, although a delay in T_{max} and reduction in C_{max} have been observed. Lamivudine exhibits linear pharmacokinetics over the therapeutic dose range and displays limited binding to the major plasma protein albumin. Lamivudine elimination will be affected by renal impairment.

Co-administration of zidovudine results in a 13 % increase in zidovudine exposure and a 28 % increase in peak plasma levels. This is not considered to be of significance to patient safety and therefore no dosage adjustments are necessary. The likelihood of adverse drug interactions with lamivudine is low due to the limited metabolism and plasma protein binding and almost complete renal clearance.

An interaction with trimethoprim, a constituent of co-trimoxazole, causes a 40 % increase in lamivudine exposure at therapeutic doses. This does not require dose adjustment unless the patient also has renal impairment. Administration of co-trimoxazole with the 3TC/zidovudine combination in patients with renal impairment should be carefully assessed. Limited data shows lamivudine penetrates the central nervous system and reaches the cerebrospinal fluid (CSF). The mean ratio CSF / serum lamivudine concentration 2 – 4 hours after oral administration is approximately 0,12. The true extent of penetration or relationship with any clinical efficacy is unknown.

Special populations:
Pharmacokinetics in children:
In general, lamivudine pharmacokinetics in paediatric patients are similar to adults. However, absolute bioavailability (approximately 55 – 65 %) was reduced in paediatric patients below 12 years of age. In addition, systemic clearance values were greater in younger paediatric patients and decreased with age approaching adult values around 12 years of age. Recent findings indicate that exposure in children 2 to < 6 years of age may be reduced by about 30 % compared with other pharmacokinetic data for patients < 3 months of age. In neonates one week of age, lamivudine oral clearance was reduced when compared to paediatric patients and is likely due to immature renal function and variable absorption.

Pharmacokinetics in pregnancy:
Following oral administration, lamivudine pharmacokinetics in late-pregnancy were similar to non-pregnant adults. Administration of lamivudine in animal toxicity studies at very high doses was not associated with any major organ toxicity. The clinically relevant effects noted were a reduction in red blood cell count and neutropenia. Lamivudine was not mutagenic in bacterial tests but, like many nucleoside analogues, showed activity in an *in vitro* cytogenic assay.

Lamivudine was not genotoxic *in vivo* at doses that gave plasma concentrations around 30 – 40 times higher than the anticipated clinical plasma levels. As the *in vitro* mutagenic activity of lamivudine could not be confirmed in *in vivo* tests it is concluded that lamivudine should not represent a genotoxic hazard to patients undergoing treatment. There is as yet no information on the tumorigenic risk in animals, and therefore any potential risk to humans must be balanced against the expected benefits of treatment.

Tenofovir disoproxil fumarate:
The pharmacokinetics of tenofovir disoproxil fumarate have been evaluated in healthy volunteers and HIV-1 infected individuals. Tenofovir pharmacokinetics are similar between these populations.

Absorption:
Tenofovir disoproxil fumarate is a water soluble diester prodrug of the active ingredient tenofovir. The oral bioavailability of tenofovir from tenofovir disoproxil fumarate in fasted patients is approximately 25 %. Following oral administration of a single 300 mg dose of tenofovir disoproxil fumarate to HIV-1 infected subjects in the fasted state, maximum serum concentrations (C_{max}) were achieved in 1,0 ± 0,4 hrs (mean ± SD), and C_{max} and AUC values were 0,30 ± 0,09 µg/mL and 2,29 ± 0,69 µg•hr/mL, respectively. The pharmacokinetics of tenofovir are dose-proportional over a dose range of 75 to 600 mg and are not affected by repeated dosing.
In a single-dose bioequivalence study conducted under non-fasted conditions (dose administered with 113,4 g apple sauce) in healthy adult volunteers, the mean C_{max} of tenofovir was 26 % lower for the oral powder relative to the tablet formulation. Mean AUC of tenofovir was similar between the oral powder and tablet formulations.

Effects of food on oral absorption:
Administration of tenofovir following a high-fat meal (~700 to 1000 kcal containing 40 % to 50 % fat) increases the oral bioavailability, with an increase in tenofovir $AUC_{0-\infty}$ of approximately 40 % and an increase in C_{max} of approximately 14 %. However, administration of tenofovir with a light meal does not have a significant effect on the pharmacokinetics of tenofovir when compared to fasted administration of the medicine. Food delays the time to tenofovir C_{max} by approximately 1 hour. C_{max} and AUC of tenofovir are 0,33 ± 0,12 µg/mL and 3,32 ± 1,37 µg•hr/mL following multiple doses of tenofovir disoproxil 300 mg once daily in the fed state, when meal content was not controlled.

Distribution:
In vitro binding of tenofovir to human plasma protein is less than 0,7 % and 7,2 %, respectively over the tenofovir concentration range of 0,01 – 25 µg/mL. The volume of distribution at steady-state is 1,3 ± 0,6 L/kg and 1,2 ± 0,4 L/kg, following intravenous administration of tenofovir 1,0 mg/kg and 3,0 mg/kg.

Cross-resistance:

Cross-resistance among certain reverse transcriptase inhibitors has been recognised. The K65R mutation can also be selected by abacavir, didanosine, or zalcitabine and results in reduced susceptibility to these medicines plus lamivudine, emtricitabine and tenofovir. Tenofovir disoproxil fumarate should be avoided in antiretroviral experienced patients with strains harbouring the K65R mutation. Patients with HIV-1 expressing three or more thymidine analogue associated mutations (TAMs) that included either the M41L or L210W reverse transcriptase mutation showed reduced susceptibility to tenofovir disoproxil fumarate.

Antiviral activity:

The *in vitro* antiviral activity of tenofovir against laboratory and clinical isolates of HIV-1 has been assessed in lymphoblastoid cell lines, primary monocyte / macrophage cells and peripheral blood lymphocytes. The IC_{50} (50 % inhibitory concentration) values for tenofovir were in the range of 0,04 µM – 8,5 µM. In medicine combination studies of tenofovir with nucleoside reverse transcriptase inhibitors (abacavir, didanosine, lamivudine, stavudine, zalcitabine, zidovudine), non-nucleoside reverse transcriptase inhibitors (delavirdine, efavirenz, nevirapine), and protease inhibitors (amprenavir, indinavir, nelfinavir, ritonavir, saquinavir), additive to synergistic effects were observed. Tenofovir displayed antiviral activity *in vitro* against HIV-1 clades A, B, C, D, E, F, G and O (IC_{50} values ranged from 0,5 µM to 2,2 µM). The IC_{50} values of tenofovir against HIV-2 ranged from 1,6 µM to 4,9 µM.

Pharmacokinetic properties:

Dolutegravir:

Dolutegravir pharmacokinetics are similar between healthy and HIV-infected subjects. The PK variability of dolutegravir is between low to moderate. In Phase 1 studies in healthy subjects, between-subjects CVb % for AUC and C_{max} ranged from -20 to 40 % and C_T from 30 to 56 % across studies. The between-subject PK variability of dolutegravir was higher in HIV-infected subjects than healthy subjects. Within-subject variability (CVw %) is lower than between-subject variability.

Absorption:

Following oral administration of dolutegravir, peak plasma concentrations were observed 2 to 3 hours post-dose. With once-daily dosing, pharmacokinetic steady-state is achieved within approximately 5 days with average accumulation ratios for AUC, C_{max}, and C_{24h} ranging from 1,2 to 1,5.

Dolutegravir plasma concentrations increased in a less than dose-proportional manner above 50 mg. Dolutegravir is a Pgp substrate *in vitro*. The absolute bioavailability of dolutegravir has not been established.

Dolutegravir may be administered with or without food. Food increased the extent and slowed the rate of absorption of dolutegravir. Bioavailability of dolutegravir depends on meal content: low, moderate and high fat meals increased dolutegravir $AUC_{(0-\infty)}$ by 33 %, 41 % and 66 %, increased C_{max} by 46 %, 52 % and 67 % and prolonged T_{max} to 3, 4, and 5 hours from 2 hours under fasted conditions, respectively. These increases are not clinically significant.

Distribution:

Dolutegravir is highly bound (greater than or equal to 98,9 %) to human plasma proteins based on *in vivo* data and binding is independent of plasma concentration of dolutegravir. The apparent volume of distribution (Vd/F) following 50 mg once daily administration is estimated at 17,4 L based on a population pharmacokinetic analysis. Binding of dolutegravir to plasma proteins is independent of concentration. Total blood and plasma drug-related radioactivity concentration ratios averaged between 0,441 to 0,535 indicating minimal association of radioactivity with blood cellular components. Free fraction of dolutegravir in plasma is estimated at approximately 0,2 to 1,1 % in healthy subjects with moderate hepatic impairment, and 0,8 to 1,0 % in subjects with severe renal impairment and 0,5 % in HIV-1 infected patients. Dolutegravir is present in cerebrospinal fluid (CSF). In 12 treatment-naïve subjects on dolutegravir 50 mg daily plus abacavir/ lamivudine, the median dolutegravir concentration in CSF was 13,2 ng/mL (range: 3,74 ng/mL to 18,3 ng/mL) 2 to 6 hours post-dose after 16 weeks of treatment. The clinical relevance of this finding has not been established.

Metabolism:

Dolutegravir is primarily metabolised via UGT1A1 with some contribution from CYP3A. Dolutegravir is the predominant circulating compound in plasma. After a single oral dose of [^{14}C] dolutegravir, 53 % of the total oral dose was excreted unchanged in faeces. It is unknown if all or part of this is due to unabsorbed medicine or biliary excretion of the glucuronidate conjugate, which can be further degraded to form the parent compound in the gut lumen. Thirty-one percent of the total oral dose is excreted in the urine, represented by ether glucuronide of dolutegravir (18,9 % of total dose), N-dealkylation metabolite (3,6 % of total dose) and a metabolite formed by oxidation at the benzylic carbon (3,0 % of total dose). Renal elimination of unchanged drug was low (less than 1 % of the dose).

Elimination:

Dolutegravir has a terminal half-life of ~14 hours and an apparent oral clearance (CL/F) of 1,0 L per hour based on population pharmacokinetic analysis.

Special populations:

Adolescents:

The pharmacokinetics of dolutegravir in 10 antiretroviral treatment-experienced HIV-1 infected adolescents (12 to 18 years of age) showed that dolutegravir 50 mg once daily dosage resulted in dolutegravir exposure comparable to that observed in adults who receive dolutegravir 50 mg once daily.

Adolescent pharmacokinetic parameters:

Age / weight	Dolutegravir dose	Dolutegravir pharmacokinetic parameter estimates geometric mean (CV %)		
		$AUC_{(0-24)}$ µg.hr/mL	C_{max} µg/mL	C_{24} µg/mL
12 to < 18 years ≥ 40 kg[a]	50 mg once daily[a]	46 (43)	3,49 (38)	0,90 (59)

[a]One subject weighing 37 kg received 35 mg once daily.

Elderly:

Population pharmacokinetic analysis of dolutegravir using data in HIV-1 infected adults showed there is no clinically relevant effect of age on dolutegravir exposure. Pharmacokinetic data for dolutegravir in subjects of > 65 years old are limited.

Renal impairment:

Renal clearance of unchanged medicine is a minor pathway of elimination for dolutegravir. A study of the pharmacokinetics of dolutegravir was performed in subjects with severe renal impairment (CLcr < 30 mL/min). No clinically important pharmacokinetic differences between subjects with severe renal impairment (CLcr < 30 mL/min) and matching healthy subjects were observed. AUC, C_{max}, and C_{24} of dolutegravir were decreased by 40 %, 23 % and 43 % respectively, compared with those in matched healthy subjects. No dosage adjustment is necessary for patients with renal impairment. Dolutegravir has not been studied in patients on dialysis, though differences in exposure are not expected.

Hepatic impairment:

Dolutegravir is primarily metabolised and eliminated by the liver. In a study comparing 8 subjects with moderate hepatic impairment (Child-Pugh category B score 7 to 9) to 8 matched healthy adult controls, the single 50 mg dose exposure of dolutegravir was similar between the two groups. No dosage adjustment is necessary for patients with mild to moderate hepatic impairment. The effect of severe hepatic impairment on the pharmacokinetics of dolutegravir has not been studied.

Polymorphisms in metabolising enzymes:

There is no evidence that common polymorphisms in metabolising enzymes alter dolutegravir pharmacokinetics to a clinically meaningful extent. In a meta-analysis using pharmacogenomics samples collected in clinical studies in healthy subjects, subjects with UGT1A1 (n = 7) genotypes conferring poor dolutegravir metabolism had a 32 % lower clearance of dolutegravir and 46 % higher AUC compared with subjects with genotypes associated with normal metabolism via UGT1A1 (n = 41). Polymorphisms in CYP3A4, CYP3A5 and NR1I2 are not associated with differences in the pharmacokinetics of dolutegravir.

Co-infection with hepatitis B or C:

Population pharmacokinetic analysis indicate that hepatitis C virus co-infection has no clinically relevant effect on the exposure to dolutegravir. There are limited data on patients with hepatitis B co-infection.

Metabolism:

In vitro studies indicate that neither tenofovir disoproxil nor tenofovir are substrates of CYP450 enzymes. Following IV administration of tenofovir, approximately 70 – 80 % of the dose is recovered in the urine as unchanged tenofovir within 72 hours of dosing. Following single dose oral administration of tenofovir, the terminal elimination half-life of tenofovir is approximately 17 hours. After multiple oral doses of tenofovir 300 mg once daily (under fed conditions), 32 ± 10 % of the administered dose is recovered in urine over 24 hours.

Elimination:

Tenofovir is eliminated by a combination of glomerular filtration and active tubular secretion. There may be competition for elimination with other compounds that are also renally eliminated.

Special populations:

Paediatrics and the elderly:

Pharmacokinetic studies have not been performed in children (< 18 years) or in the elderly (> 65 years).

Hepatic impairment:

Tenofovir pharmacokinetics after a 300 mg single dose have been studied in non-HIV infected patients with moderate to severe hepatic impairment. There were no substantial alterations in tenofovir pharmacokinetics in patients with hepatic impairment compared with unimpaired patients. Change in tenofovir dosing is not required in patients with hepatic impairment.

Renal impairment:

Tenofovir pharmacokinetics are altered in patients with renal impairment. In patients with creatinine clearance < 50 mL/min or with end-stage renal disease (ESRD) requiring dialysis, C_{max} and $AUC_{0-\infty}$ of tenofovir were increased. It is recommended that the dosing interval for tenofovir be modified in patients with creatinine clearance < 50 mL/min or in patients with ESRD who require dialysis (see **"DOSAGE AND DIRECTIONS FOR USE"**). Tenofovir is efficiently removed by haemodialysis with an extraction coefficient of approximately 54%. Following a single 300 mg dose of tenofovir, a four-hour haemodialysis session removed approximately 10 % of the administered tenofovir dose.

INDICATIONS:

REYDIN is indicated for the treatment of HIV-1 infection in adults aged 18 years and older.

CONTRAINDICATIONS:

REYDIN is contraindicated in:

- Patients with known hypersensitivity to dolutegravir, lamivudine, tenofovir disoproxil fumarate or any of the components of **REYDIN**.
- Uncontrolled renal failure (see **"WARNINGS AND SPECIAL PRECAUTIONS"**).
- Pregnancy and lactation (see **"HUMAN REPRODUCTION"**).
- Women of child-bearing age not using highly effective contraception.
- Concomitant use with adefovir dipivoxil.
- Co-administration with dofetilide and pilsicainide.
- Co-administration with didanosine.
- Co-administration with metformin.
- Patients younger than 18 years of age.
- Moderate and severe hepatic impairment.

WARNINGS AND SPECIAL PRECAUTIONS:

Safety and efficacy of the individual active ingredients in various antiretroviral combination regimens with similar dosages as contained in **REYDIN** have been established in clinical studies for the treatment of HIV patients. However, safety and efficacy of the fixed-drug combination as in **REYDIN** for the treatment of HIV have not been established in clinical studies. The complete professional information of the other medicines used in combination should be consulted before initiation of therapy.

Metabolic abnormalities:

Combination antiretroviral therapy, including **REYDIN** has been associated with metabolic abnormalities such as hypertriglyceridaemia, hypercholesterolaemia, insulin resistance, hyperglycaemia and hyperlactataemia.

Lipodystrophy:

Combination antiretroviral therapy, including **REYDIN**, has also been associated with the redistribution / accumulation of body fat, including central obesity, dorso-cervical fat enlargement (buffalo hump), peripheral wasting, facial wasting and breast enlargement in HIV patients.

A higher risk of lipodystrophy has been associated with individual factors such as older age, and with medicine related factors such as longer duration of antiretroviral treatment and associated metabolic disturbances. Clinical examination should include evaluation for physical signs of fat redistribution. Fasting serum lipids and blood glucose levels should be monitored. Lipid disorders should be managed as clinically appropriate. Patients with evidence of lipodystrophy should also have a thorough cardiovascular risk assessment.

Immune reconstitution inflammatory syndrome:

Immune reconstitution inflammatory syndrome (IRIS) is an immunopathological response resulting from the rapid restoration of pathogen-specific immune responses to pre-existing antigens combined with immune dysregulation, which occurs shortly after starting combination anti-retroviral therapy (cART). Typically, such a reaction presents by paradoxical deterioration of opportunistic infections being treated or with unmasking of an asymptomatic opportunistic disease, often with an atypical inflammatory presentation. IRIS usually develops within the first three months of initiation of ART and occurs more commonly in patients with low CD4 counts. Common examples of IRIS reactions to opportunistic diseases are tuberculosis, atypical mycobacterial infections, cytomegalovirus retinitis, *Pneumocystis jirovecii*, and cryptococcal meningitis.

Appropriate treatment of the opportunistic disease should be instituted or continued, and ART continued. Inflammatory manifestations generally subside after a few weeks. Severe cases may respond to glucocorticoids, but there is only limited evidence for this in patients with tuberculosis IRIS. Autoimmune disorders (such as Graves' disease, Guillain-Barre syndrome, polymyositis) have also been reported as IRIS reactions; however, the reported time to onset is more variable and these events can occur many months after initiation of treatment.

Osteonecrosis:

Although the aetiology is considered to be multifactorial (including corticosteroid use, alcohol consumption, severe immunosuppression, higher body mass index), cases of osteonecrosis have been reported particularly in patients with advanced HIV-disease and/or long-term exposure to combination antiretroviral therapy (cART), including components of **REYDIN**. Patients should be advised to seek medical advice if they experience joint aches and pain, joint stiffness or difficulty in movement.

Opportunistic infections:

Patients receiving **REYDIN** may continue to develop opportunistic infections and other complications of HIV infection and therefore should remain under close clinical observation by doctors experienced in the treatment of patients with HIV associated diseases.

The risk of HIV transmission to others:

Patients must be advised that treatment with **REYDIN**, has not been proven to prevent the risk of transmission of HIV to others through sexual contact or blood contamination. Appropriate precautions must continue to be used.

Lactic acidosis / severe hepatomegaly with steatosis:

Lactic acidosis, usually associated with hepatic steatosis, including fatal cases, has been reported with the use of nucleoside analogues, such as in **REYDIN**. Early symptoms (symptomatic hyperlactataemia) include benign digestive symptoms (nausea, vomiting and abdominal pain), non-specific malaise, loss of appetite, weight loss, respiratory symptoms (rapid and/or deep breathing) or neurological symptoms (including motor weakness). Lactic acidosis has a high mortality and may be associated with pancreatitis, liver failure or renal failure.

Lactic acidosis generally occurs after a few or several months of treatment. Treatment with nucleoside analogues should be discontinued in the setting of symptomatic hyperlactataemia and metabolic / lactic acidosis, progressive hepatomegaly, or rapidly elevating aminotransferase levels.

Suspicious biochemical features include mild raised transaminases, raised lactate dehydrogenase (LDH) and/or creatine kinase. In patients with suspicious symptoms or biochemistry, measure the venous lactate level (normal < 2 mmol/L) and respond as follows:

- Lactate 2 – 5 mmol/L: monitor regularly and be alert for clinical signs.
- Lactate 5 – 10 mmol/L without symptoms: monitor closely.
- Lactate 5 – 10 mmol/L with symptoms: STOP all therapy. Exclude other causes (e.g. sepsis, uraemia, diabetic ketoacidosis, thyrotoxicosis, lymphoma).
- Lactate > 10 mmol/L: STOP all therapy (80 % mortality in case studies).

The above lactate values may not be applicable to paediatric patients.

Diagnosis of lactic acidosis is confirmed by demonstrating metabolic acidosis with an increased anion gap and raised lactate level. Therapy should be stopped in any acidotic patient with a raised lactate level.

Lactic acidosis and severe hepatomegaly with steatosis, including fatal cases, have been reported with the use of **REYDIN** alone or in combination, in the treatment of HIV infection. Most cases were women. Caution should be exercised when administering **REYDIN** to patients with known risk factors for liver disease.

Treatment with **REYDIN** should be suspended in any patient who develops clinical or laboratory findings suggestive of lactic acidosis or hepatotoxicity. Caution should be exercised when administering nucleoside analogues, as contained in **REYDIN**, to any patient (particularly obese women) with hepatomegaly, hepatitis or other known risk factors for liver disease and hepatic steatosis (including certain medicines and alcohol). Patients co-infected with hepatitis C and treated with alpha interferon and ribavirin may constitute a special risk. Patients at increased risk should be followed closely. However, cases have also been reported in patients with no known risk factors.

Patients at increased risk should be followed closely. There are no study results demonstrating the effect of **REYDIN** on clinical progression of HIV-1.

Mitochondrial dysfunction:
Nucleoside and nucleotide analogues, as contained in **REYDIN**, have been demonstrated *in vitro* and *in vivo* to cause a variable degree of mitochondrial damage. There have been reports of mitochondrial dysfunction in HIV negative infants exposed *in utero* and/or postnatally to nucleoside analogues. The main adverse events reported are haematological disorders (anaemia, neutropenia), and metabolic disorders (hyperlactataemia, hyperlipidaemia). These events are often transitory. Some late-onset neurological disorders have been reported (hypertonia, convulsion, abnormal behaviour). Whether the neurological disorders are transient or permanent is unknown. Any child exposed *in utero* to nucleoside and nucleotide analogues, even HIV negative children, should have clinical and laboratory follow-up and should be fully investigated for possible mitochondrial dysfunction in case of relevant signs or symptoms.

Pancreatitis:
Pancreatitis has been observed in some patients receiving lamivudine, as in **REYDIN**. It is unclear whether this is due to lamivudine or to underlying HIV disease. Pancreatitis must be considered whenever a patient develops abdominal pain, nausea, vomiting or elevated biochemical markers. Discontinue use of **REYDIN** until diagnosis of pancreatitis is excluded.

Patients with moderate to severe renal impairment:
In patients with moderate to severe renal impairment, the terminal half-life of **REYDIN** is increased due to decreased clearance. The dose of **REYDIN** should therefore be adjusted (see **"DOSAGE AND DIRECTIONS FOR USE"**).

Liver disease:
Use of **REYDIN** can result in hepatomegaly due to non-alcoholic fatty liver disease (hepatic steatosis). The safety and efficacy of **REYDIN** has not been established in patients with significant underlying liver disorders. Patients with pre-existing liver dysfunction, including chronic active hepatitis, have an increased frequency of liver function abnormalities during combination antiretroviral therapy and should be monitored according to standard practice. If there is evidence of worsening liver disease in such patients, interruption or discontinuation of treatment must be considered.

Renal impairment:
REYDIN is a combination medicine and the dose of the individual components cannot be altered. Tenofovir and lamivudine are principally eliminated by the kidney. **REYDIN** is not recommended for patients with creatinine clearance < 50 mL/min or patients who require haemodialysis. Renal impairment, including cases of acute renal failure and Fanconi syndrome (renal tubular injury with severe hypophosphataemia) has been reported with the use of tenofovir disoproxil fumarate in clinical practice. Careful monitoring of renal function (serum creatinine and serum phosphate) is therefore recommended before taking **REYDIN**.

Renal function:
Since **REYDIN** is primarily eliminated by the kidneys, co-administration of **REYDIN** with medicines that reduce renal function or compete for active tubular secretion may increase serum concentrations of **REYDIN** and/or increase the concentrations of other renally eliminated medicines. Some examples include, but are not limited to adefovir dipivoxil, cidofovir, aciclovir, valaciclovir, ganciclovir and valganciclovir.

Renal safety with tenofovir has only been studied to a very limited degree in adult patients with impaired renal function (creatinine clearance < 80 mL/min).

Renal monitoring:
It is recommended that renal function (creatinine clearance and serum phosphate) is assessed in all patients prior to initiating therapy with tenofovir disoproxil fumarate and that it is also monitored every four weeks during the first year of tenofovir disoproxil fumarate therapy, and then every three months. In patients at risk for renal impairment, including patients who have previously experienced renal events while receiving adefovir dipivoxil, consideration should be given to more frequent monitoring of renal function.

Co-administration and risk of renal toxicity:
Use of tenofovir disoproxil fumarate should be avoided with concurrent or recent use of a nephrotoxic medicine (e.g. aminoglycosides, amphotericin B, foscarnet, ganciclovir, pentamidine, vancomycin, cidofovir or interleukin-2). If concomitant use of tenofovir disoproxil fumarate and nephrotoxic medicines is unavoidable, renal function should be monitored weekly.

Tenofovir disoproxil fumarate has not been clinically evaluated in patients receiving medicines which are secreted by the same renal pathway, including the transport proteins human organic anion transporter (hOAT) 1 and 3 or MRP 4 (e.g. cidofovir, a known nephrotoxic medicine). These renal transport proteins may be responsible for tubular secretion and in part, renal elimination of tenofovir and cidofovir. Consequently, the pharmacokinetics of these medicines, which are secreted by the same renal pathway, including transport proteins hOAT 1 and 3 or MRP 4, might be modified if they are co-administered. Unless clearly necessary, concomitant use of these medicines which are secreted by the same renal pathway is not recommended, but if such use is unavoidable, renal function should be monitored weekly.

REYDIN should be avoided with concurrent or recent use of a nephrotoxic medicine. Patients at risk of, or with a history of renal dysfunction and patients receiving concomitant nephrotoxic substances should be carefully monitored for changes in serum creatinine and phosphorus.

K65R mutation:
REYDIN should be avoided in antiretroviral experienced patients with HIV-1 harbouring the K65R mutation.

Bone mineral density:
Decreases in bone mineral density of the spine and changes in bone biomarkers from baseline are significantly greater with tenofovir disoproxil fumarate, as contained in **REYDIN**. Decreases in bone mineral density of the hip are significantly greater. Clinically relevant bone fractures are reported. If bone abnormalities are suspected, then appropriate consultation should be obtained. Bone monitoring should be considered for HIV infected patients who have a history of pathologic bone fracture or are at risk of osteopenia.

REYDIN may cause a reduction in bone mineral density. The effects of tenofovir disoproxil fumarate associated changes in bone mineral density on long-term bone health and future fracture risk are currently unknown.

Bone monitoring should be considered for HIV infected patients who have a history of pathologic bone fracture or are at risk for osteopenia. Although the effect of supplementation with calcium and vitamin D was not studied, such supplementation may be beneficial for all patients. If bone abnormalities are suspected, then appropriate consultation should be obtained. Bone abnormalities (infrequently contributing to fractures) may be associated with proximal renal tubulopathy.

Patients with HIV and hepatitis B or C virus co-infection:
REYDIN is not indicated for the treatment of chronic HBV infection. The safety and efficacy of **REYDIN** has not been established for the treatment of patients co-infected with HBV and HIV.

Patients with chronic hepatitis B or C and treated with antiretroviral therapy are at an increased risk for severe and potentially fatal hepatic adverse reactions. Medical practitioners should refer to current HIV treatment guidelines for the optimal management of HIV infection in patients co-infected with hepatitis B virus (HBV). In case of concomitant antiviral therapy for hepatitis B or C, please refer also to the relevant professional information for these medicines.

Patients with chronic hepatitis B or C treated with **REYDIN** are at an increased risk for severe and potentially fatal hepatic adverse reactions. Doctors should refer to current HIV treatment guidelines for the optimal management of HIV infection in patients co-infected with hepatitis B virus (HBV).

Following multiple dosing to HIV-negative subjects receiving either chronic methadone maintenance therapy, oral contraceptives, or single doses of ribavirin, steady-state tenofovir pharmacokinetics were similar to those observed in previous studies, indicating a lack of clinically significant medicine interactions between these medicines and tenofovir disoproxil fumarate.

Table 2:
Medicine interactions: Changes in pharmacokinetic parameters for co-administered medicines in the presence of tenofovir:

Co-administered medicine	Dose of co-administered medicine (mg)	N	% Change of co-administered medicine pharmacokinetic parameters[1]		
			C_{max}	AUC	C_{min}
Abacavir	300 once	8	↑ 122 (↑ 1 to ↑ 26)	↔	NA
Adefovir dipivoxil	10 once	22	↔	↔	NA
Efavirenz	600 mg once daily x 14 days	30	↔	↔	↔
Emtricitabine	200 mg once daily x 7 days	17	↔	↔	↔
Indinavir	800 mg three times daily x 7 days	12	↑ 14 (↓ 3 to ↑33)	↔	↔
Lamivudine	150 mg twice daily x 7 days	15	↔	↔	↔
Lopinavir/ Ritonavir	400/100 mg twice daily x 14 days	21	↔	↔	↔
Methadone[2]	40 – 110 once daily x 14 days[3]	13	↔	↔	↔
Oral contraceptives[4]	Ethinyl oestradiol/ norgestimate (Ortho-Tricyclen®) Once daily x 7 days	20	↔	↔	↔
Ribavirin	600 once	22	↔	↔	NA
Ritonavir	Lopinavir/ritonavir 400/100 twice daily x 14 days	24	↔	↔	↔
Atazanavir[5]	400 once daily x 14 days	29	↔	↔	↔
Atazanavir[6]	Atazanavir/ritonavir 300/100 once daily x 42 days	10	↑ 28 (↑ 50 to ↑ 5)	↑ 25 (↑ 42 to ↑ 3)	↑ 23[6] (↑ 46 to ↑ 10)

1. Increase = ↑; Decrease = ↓; No effect = ↔; NA = Not applicable
2. R-(active), S- and total methadone exposures were equivalent when dosed alone or with tenofovir as tenofovir disoproxil fumarate 300 mg.
3. Individual subjects were maintained on their stable methadone dose. No pharmacodynamic alterations (opiate toxicity or withdrawal signs or symptoms) were reported.
4. Ethinyl oestradiol and 17-deacetyl norgestimate (pharmacologically active metabolite) exposures were equivalent when dosed alone or with tenofovir as tenofovir disoproxil fumarate 300 mg.
5. Reyataz US prescribing information (Bristol-Meyers Squibb).
6. In HIV-infected patients, addition of tenofovir disoproxil fumarate to atazanavir 300 mg plus ritonavir 100 mg, resulted in AUC and C_{min} values of atazanavir that were 2,3- and 4-fold higher than the respective values observed for atazanavir 400 mg when given alone.

Lamivudine:
The likelihood of metabolic interactions is low due to limited metabolism and plasma protein binding and almost complete renal clearance.

Zidovudine plasma levels are not significantly altered when co-administered with **REYDIN**. Zidovudine has no effect on the pharmacokinetics of **REYDIN**.

Table 3:
Medicine interaction: Changes in pharmacokinetic parameters for co-administered medicines in the presence of lamivudine.

Co-administered medicine	Dose of co-administered medicine (mg)	% Change of co-administered medicine pharmacokinetic parameters[1]		
		C_{max}	AUC	C_{min}
Trimethoprim/ Sulphametoxazole	160/800 mg	NS	↑ 40	NS
Zidovudine	150/300 mg twice daily	↑ 28	↑ 13	NS

1. Increase = ↑; NS = Not studied

Co-administration of zidovudine results in a 13 % increase in zidovudine exposure and a 28 % increase in peak plasma levels. This is not considered to be of significance to patient safety and therefore no dosage adjustments are necessary.

REYDIN may inhibit the intracellular phosphorylation of zalcitabine when the two medicines are used concurrently. **REYDIN** is therefore not recommended to be used in combination with zalcitabine.

Administration of trimethoprim, a constituent of co-trimoxazole causes an increase in **REYDIN** plasma levels. Unless the patient has renal impairment, no dosage adjustment of **REYDIN** is necessary. **REYDIN** has no effect on the pharmacokinetics of co-trimoxazole.

The possibility of interactions with other medicines administered concurrently should be considered, particularly when the main route is renal.

The co-administration of **REYDIN** with etravirine (ETR) is not recommended unless the patient is also receiving concomitant atazanavir + ritonavir (ATV + RTV), lopinavir + ritonavir (LPV + RTV) or darunavir + ritonavir (DRV + RTV).

Dolutegravir:
Rifampicin decreases the blood levels of dolutegravir. A supplementary dose of dolutegravir should be given to patients taking **REYDIN**.

There is evidence that the concentration of isoniazid is increased by dolutegravir, as contained in **REYDIN**.

Effects of REYDIN on the pharmacokinetics of other medicines:
In vitro, dolutegravir demonstrated no direct, or weak inhibition of the enzymes cytochrome P450, uridine diphosphate glucuronosyl transferase or the transporter Pgp. *In vitro*, dolutegravir did not induce CYP1A2, CYP2B6 or CYP3A4, *in vivo*, dolutegravir did not have an effect on midazolam, a CYP3A4 probe. Therefore, dolutegravir, as in **REYDIN**, is not expected to affect the pharmacokinetics of medicines that are substrates of these enzymes or transporters [i.e. reverse transcriptase and protease inhibitors, opioid analgesics, antidepressants, statins, azole antifungals (such as fluconazole, itraconazole, clotrimazole), proton pump inhibitors (such as esomeprazole, lansoprazole, omeprazole), anti-erectile dysfunction agents (such as sildenafil, tadalafil, vardenafil), acyclovir, valaciclovir, sitagliptin, adefovir].

Dolutegravir, as in **REYDIN**, does not have any clinically relevant effect on the pharmacokinetics of the following: tenofovir, methadone, efavirenz, lopinavir, atazanavir, darunavir, etravirine, fosamprenavir, rilpivirine, telaprevir and oral contraceptives containing norgestimate and ethinyl oestradiol.

In vitro, dolutegravir inhibits the renal organic cation transporter 2 (OCT2). Based on this, **REYDIN** may increase plasma concentrations of medicines in which excretion is dependent upon OCT2 (dofetilide, metformin).

Effects of other medicines on the pharmacokinetics of dolutegravir, as in REYDIN:
Dolutegravir, as in **REYDIN**, is eliminated mainly through metabolism by UGT1A1. Dolutegravir is also a substrate of UGT1A3, UGT1A9, CYP3A4, Pgp and BCRP; therefore, medicines that induce those enzymes may theoretically decrease dolutegravir plasma concentrations and reduce the therapeutic effect of dolutegravir in **REYDIN**. Co-administration of **REYDIN** and other medicines that inhibit UGT1A1, UGT1A3, UGT1A9, CYP3A4, and/or Pgp may increase dolutegravir plasma concentration.

Exacerbations of hepatitis:

Flares on treatment:

Spontaneous exacerbations in chronic hepatitis B are relatively common and are characterised by transient increases in serum ALT. After initiating antiviral therapy, serum ALT may increase in some patients. In patients with compensated liver disease, these increases in serum ALT are generally not accompanied by an increase in serum bilirubin concentrations or hepatic decompensation. Patients with cirrhosis may be at a higher risk for hepatic decompensation following hepatitis exacerbation, and therefore should be monitored closely during therapy.

Flares after treatment discontinuation:

Acute exacerbations of hepatitis have been reported in patients after the discontinuation of hepatitis B therapy. Post-treatment exacerbations are usually associated with rising HBV DNA, and the majority appears to be self-limited. However, severe exacerbations, including fatalities, have been reported. Hepatic function should be monitored at repeated intervals with both clinical and laboratory follow-up for at least 6 months after discontinuation of hepatitis B therapy. If appropriate, resumption of hepatitis B therapy may be warranted. In patients with advanced liver disease or cirrhosis, treatment discontinuation is not recommended since post-treatment exacerbation of hepatitis may lead to hepatic decompensation. Liver flares are especially serious, and sometimes fatal in patients with decompensated liver disease.

Hypersensitivity reactions:

Hypersensitivity reactions have been reported with integrase inhibitors, including dolutegravir and were characterised by rash, constitutional findings and sometimes organ dysfunction, including liver injury. Discontinue **REYDIN** and other suspect agents immediately if signs or symptoms of hypersensitivity reactions develop (including, but not limited to severe rash or rash accompanied by fever, general malaise, fatigue, muscle or joint aches, blisters, oral lesions, conjunctivitis, facial oedema, hepatitis, eosinophilia, angioedema). Clinical status, including liver aminotransferases, should be monitored and appropriate therapy initiated. Delay in stopping treatment with **REYDIN**, or other suspect agents, after the onset of hypersensitivity may result in a life-threatening reaction.

Paediatric use:

Safety and effectiveness in paediatric patients and patients < 18 years of age have not been established.

Use in elderly:

Clinical studies did not include sufficient numbers of subjects aged 65 and over to determine whether they respond differently from younger subjects.

Effects on ability to drive and use machines:

REYDIN may cause dizziness, impaired concentration and/or drowsiness and may affect the ability to drive and use machines. Patients should ensure that they do not engage in driving or using machines until they know how **REYDIN** affects them.

INTERACTIONS:

The likelihood of interactions is low due to the limited metabolism and plasma protein binding and almost complete renal clearance. Zidovudine plasma levels are not significantly altered when co-administered with lamivudine. Zidovudine has no effect on the pharmacokinetics of lamivudine. Lamivudine may inhibit the intracellular phosphorylation of zalcitabine when the two medicines are used concurrently. Lamivudine is therefore not recommended to be used in combination with zalcitabine. Administration of trimethoprim, a constituent of co-trimoxazole, causes an increase in lamivudine plasma levels. However, unless the patient has renal impairment, no dosage adjustment of lamivudine is necessary. Lamivudine has no effect on the pharmacokinetics of co-trimoxazole. The possibility of interactions with other drugs administered concurrently should be considered, particularly when the main route is renal.

No medicine interaction studies have been conducted using **REYDIN**. As **REYDIN** contains tenofovir disoproxil fumarate and lamivudine, any interactions that have been identified with these individual medicines may occur with **REYDIN**. Important medicine interaction information for **REYDIN** is summarised in Table 1, 2 and 3. The medicine interactions described are based on studies conducted with tenofovir disoproxil fumarate or lamivudine as individual medicines or are potential medicine interactions. While the tables include potentially significant interactions, they are not all inclusive. Based on the results of *in vitro* experiments and the known elimination pathway of tenofovir, the potential for CYP450-mediated interactions involving tenofovir with other medicines is low.

An interaction with trimethoprim, a constituent of co-trimoxazole, causes a 40 % increase in lamivudine exposure at therapeutic doses. This does not require dose adjustment unless the patient also has renal impairment. Administration of co-trimoxazole with the lamivudine/ zidovudine combination in patients with renal impairment should be carefully assessed.

Tenofovir:

Renally eliminated medicines:

Tenofovir, as in **REYDIN**, is primarily excreted by the kidneys by a combination of glomerular filtration and active tubular secretion. Co-administration of **REYDIN** with medicines that are eliminated by active tubular secretion may increase serum concentrations of either tenofovir or the co-administered medicines due to competition for this elimination pathway. Medicines that decrease renal function may also increase serum concentrations of tenofovir, as in **REYDIN**.

Tenofovir has been evaluated in healthy volunteers in combination with abacavir, adefovir dipivoxil, atazanavir, didanosine, efavirenz, emtricitabine, indinavir, lamivudine, lopinavir/ritonavir, methadone, oral contraceptives and ribavirin. Tables 1 and 2 summarise pharmacokinetic effects of co-administered medicine on tenofovir pharmacokinetics and effects of tenofovir on the pharmacokinetics of co-administered medicine.

When administered with multiple doses of tenofovir, the C_{max} and AUC of didanosine 400 mg increased significantly. The mechanism of this interaction is unknown. When didanosine 250 mg enteric-coated capsules were administered with tenofovir, systemic exposures to didanosine were similar to those seen with the 400 mg enteric-coated capsules alone under fasted conditions.

Table 1:

Medicine interactions: Changes in pharmacokinetic parameters for tenofovir[1] in the presence of co-administered medicines:

Co-administered medicine	Dose of co-administered medicine (mg)	N	% Change of tenofovir pharmacokinetic parameters[2] (90 % CI)		
			C_{max}	AUC	C_{min}
Abacavir	300 once	8	↔	↔	NC
Adefovir dipivoxil	10 once	22	↔	↔	↔
Atazanavir	400 once daily x 14 days	33	↑ 14 (↑ 8 to ↑ 20)	↑ 24 (↑ 21 to ↑ 28)	↑ 22 (↑ 15 to ↑ 30)
Didanosine (enteric-coated)	400 once	25	↔	↔	↔
Didanosine (buffered)	250 or 400 once daily x 7 days	14	↔	↔	↔
Efavirenz	600 once daily x 14 days	29	↔	↔	↔
Emtricitabine	200 once daily x 7 days	17	↔	↔	↔
Indinavir	800 three times daily x 7 days	13	↑ 14 (↓ 3 to ↑33)	↔	↔
Lamivudine	150 twice daily x 7 days	15	↔	↔	↔
Lopinavir/ Ritonavir	400/100 twice daily x 14 days	24	↔	↑ 32 (↑ 25 to ↑ 38)	↑ 51 (↑ 37 to ↑ 66)

1. Patients received tenofovir DF 300 mg once daily
2. Increase = ↑; Decrease = ↓; No effect = ↔; NC = Not calculated

Efavirenz, nevirapine, rifampicin and tipranavir in combination with ritonavir each reduces the plasma concentrations of dolutegravir significantly and requires dolutegravir dose adjustment of 50 mg twice daily. Etravirine also reduces plasma concentrations, but the effect of etravirine was mitigated by co-administration of the CYP3A4 inhibitors lopinavir/ritonavir, darunavir/ritonavir and is expected to be mitigated by atazanavir/ritonavir. Therefore, no dose adjustment is necessary when co-administered with etravirine. Another inducer, fosamprenavir in combination with ritonavir decreased plasma concentrations of dolutegravir but does not require a dosage adjustment. Caution is warranted, and clinical monitoring is recommended when these combinations are given in INI-resistant patients. A medicine interaction study with the UGT1A1 inhibitor, atazanavir, did not result in a clinically meaningful increase in the plasma concentrations of dolutegravir. Tenofovir, ritonavir, lopinavir/ritonavir, darunavir/ritonavir, rilpivirine, boceprevir, telaprevir, prednisone, rifabutin and omeprazole had no or a minimal effect on dolutegravir pharmacokinetics, therefore no dose adjustment of dolutegravir is required when co-administered with these medicines.

Dolutegravir medicine interactions:

Concomitant medicine class: Medicine name	Effect on concentration of dolutegravir or concomitant medicine			
	Dolutegravir / concomitant medicine	AUC	C_{max}	C_T
HIV-1 antiviral agents				
Non-nucleoside reverse transcriptase inhibitor: Etravirine (ETR)	Dolutegravir ↑ ETR ↔	↓ 71 %	↓ 52 %	↓ 75 %
Non-nucleoside reverse transcriptase inhibitor: Efavirenz (EFV)	Dolutegravir ↓ EFV ↔	↓ 57 %	↓ 39 %	↓ 75 %
Non-nucleoside reverse transcriptase inhibitor: Nevirapine	Dolutegravir ↓	NS	NS	NS
Protease inhibitor: Atazanavir (ATV)	Dolutegravir ↑ ATV ↔	↑ 91 %	↑ 49 %	↑ 180 %
Protease inhibitor: Atazanavir/ritonavir (ATV + RTV)	Dolutegravir ↑ ATV ↔ RTV ↔	↑ 62 %	↑ 33 %	↑ 121 %
Protease inhibitor: Tipranavir/ritonavir (TPV + RTV)	Dolutegravir ↓ TPV ↔ RTV ↔	↓ 59 %	↓ 47 %	↓ 76 %
Protease inhibitor: Fosamprenavir/ritonavir (FPV + RTV)	Dolutegravir ↓ FPV ↔ RTV ↔	↓ 35 %	↓ 24 %	↓ 49 %
Protease inhibitor: Nelfinavir	Dolutegravir ↔	NS	NS	NS
Protease inhibitor: Lopinavir/ritonavir (LPV + RTV)	Dolutegravir ↔ LPV ↔ RTV ↔	↔	↔	↔
Protease inhibitor: Darunavir/ritonavir (DRV/RTV)	Dolutegravir ↓ DRV ↔ RTV ↔	↓ 32 %	↓ 11 %	↓ 38 %
Nucleoside reverse transcriptase inhibitor: Tenofovir (TDV)	Dolutegravir ↔TDV ↔	NA	NA	NA
Protease inhibitor: Lopinavir/ritonavir + etravirine (LPV/RTV + ETR)	Dolutegravir ↔ LPV ↔ RTV ↔ ETR ↔	↑ 10 %	↑ 7 %	↑ 28 %
Other medicines				
Dofetilide Pilsicainide	Dofetilide ↑ Pilsicainide ↑	NS	NS	NS
Oxcarbazepine Phenytoin Phenobarbitone Carbamazepine St. John's Wort	Dolutegravir ↓	NS	NS	NS
Antacids containing polyvalent cations (e.g. Mg, Al, or Ca)	Dolutegravir ↓	↓ 74 %	↓ 72 %	↓ 74 %
Calcium supplements	Dolutegravir ↓	↓ 39 %	↓ 37 %	↓ 39 %
Iron supplements	Dolutegravir ↓	↓ 54 %	↓ 57 %	↓ 56 %
Metformin	Metformin ↑	N/A	N/A	N/A
Rifampicin	Dolutegravir ↓	↓ 54 %	↓ 43 %	↓ 72 %
Oral contraceptives [ethinyl oestradiol (EE) and norgestromin (NGMN)]	EE ↔	↑ 3 %	↓ 1 %	↑ 2 %
	NGMN ↔	↓ 2%	↓ 11 %	↓ 7 %
Methadone	Methadone ↔	↓ 2 %	↔ 0 %	↓ 1 %

Abbreviations: ↑ = Increase; ↓ = Decrease; ↔ = No significant change; NA = Not applicable; NS = Not studied.

Co-administration of dolutegravir has the potential to increase dofetilide or pilsicainide plasma concentration via inhibition of OCT2 transporter. Dofetilide or pilsicainide co-administration with **REYDIN** is contraindicated due to the potential life-threatening toxicity caused by high dofetilide or pilsicainide concentrations (see **"CONTRAINDICATIONS"**).

Co-administration of antacids containing polyvalent cations (e.g., Mg, Al, Fe or Ca) decreases dolutegravir plasma concentration. **REYDIN** should not be co-administered with polyvalent cation-containing antacids. **REYDIN** is recommended to be administered 2 hours before or 6 hours after these medicines.

Metformin concentrations may be increased by **REYDIN**. Metformin is contraindicated in patients taking **REYDIN** (see **"CONTRAINDICATIONS"**).

HUMAN REPRODUCTION:

Pregnancy:

REYDIN is contraindicated in human pregnancy and lactation. Neural tube defects have been noted in an observational study in humans, where dolutegravir-based regimens were used at the time of conception and early pregnancy (see **"CONTRAINDICATIONS"**).

Tenofovir, dolutegravir and lamivudine were shown to cross the placenta in reproductive toxicity studies in animals. Late onset neurological disorders, including seizures, have been observed in children who have been exposed to nucleoside analogues such as tenofovir and lamivudine (see **Mitochondrial dysfunction** under **"WARNINGS AND SPECIAL PRECAUTIONS"**).

REYDIN should not be prescribed in women who plan to become pregnant. Women of child-bearing age should not use **REYDIN** unless they are reliably using highly effective contraception. Treatment with **REYDIN** should not be initiated without a medically supervised negative pregnancy test. This test should be repeated at frequent intervals during treatment with **REYDIN**, and especially in the event that pregnancy is suspected.

Lactation:

Mothers breastfeeding their infants should not use **REYDIN**. Lamivudine is excreted in human milk at similar concentrations to those found in serum; tenofovir is excreted in breast milk and it is not known whether dolutegravir is excreted in human milk.

DOSAGE AND DIRECTIONS FOR USE:

Therapy should be initiated by a medical practitioner experienced in the management of HIV infection.

Adults:

The dose of **REYDIN** is one tablet taken orally, once daily, without regard to food.

Paediatrics:

REYDIN is not recommended for use in patients younger than 18 years of age.

Dose adjustment for renal impairment:

Significantly increased exposure occurred when tenofovir, as in **REYDIN**, was administered to patients with moderate to severe renal impairment (see **"CONTRAINDICATIONS"**).

The pharmacokinetics of tenofovir, as in **REYDIN**, have not been evaluated in non-haemodialysis patients with creatinine clearance < 50 mL/min, therefore, no dosing recommendations is available for these patients. **REYDIN** is not suitable for use in patients with renal impairment with creatinine clearance less than 50 mL/min.

Rifampicin decreases the blood levels of dolutegravir. A supplementary dose of dolutegravir should be given to patients taking **REYDIN**.

SIDE EFFECTS:

REYDIN can have side effects.

Dolutegravir:

Immune system disorders:

Less frequent: Hypersensitivity, immune reconstitution syndrome.

Psychiatric disorders:

Frequent: Insomnia.

Nervous system disorders:

Frequent: Headache, dizziness, abnormal dreams.

Gastrointestinal disorders:

Frequent: Nausea, diarrhoea.
Less frequent: Vomiting, flatulence, upper abdominal pain.
Frequency unknown: Abdominal pain, abdominal discomfort.

Hepatobiliary disorders:

Frequency not known: Hepatitis.

Skin and subcutaneous tissue disorders:

Frequent: Rash, pruritus.

Lamivudine:

The following side effects have been reported during therapy for HIV disease with **REYDIN** tablets alone and in combination with other antiretrovirals:

Blood and lymphatic system disorders:

Less frequent: Neutropenia, anaemia, thrombocytopenia.
Frequency unknown: Pure red cell aplasia.

Metabolism and nutrition disorders:

Frequent: Hyperlactataemia.
Less frequent: Lactic acidosis, lipodystrophy (redistribution / accumulation of body fat) (see **"WARNINGS AND SPECIAL PRECAUTIONS"**).

Nervous system disorders:

Frequent: Headache, insomnia.
Frequency unknown: Peripheral neuropathy (or paraesthesia), late onset neurological disorders in children exposed *in utero*.

Gastrointestinal disorders:

Frequent: Nausea, vomiting, upper abdominal pain or cramps, diarrhoea.
Less frequent: Pancreatitis, elevations in serum amylase.

Hepatobiliary disorders:

Less frequent: Transient elevations in liver enzymes (AST, ALT).

Skin and subcutaneous tissue disorders:

Frequent: Rash, alopecia.

Musculoskeletal, connective tissue and bone disorders:

Frequent: Arthralgia, muscle disorders.
Less frequent: Rhabdomyolysis, decrease in bone mineral density, osteopenia, fractures.

General disorders and administration site conditions:

Frequent: Fatigue, malaise, fever.

Tenofovir:

Immune system disorders:

Less frequent: Allergic reactions.

Metabolism and nutrition disorders:

Frequency unknown: Hypophosphataemia, lactic acidosis.

Respiratory, thoracic and mediastinal disorders:

Frequency unknown: Dyspnoea.

Gastrointestinal disorders:

Frequent: Anorexia, dyspepsia, flatulence, abdominal pain.
Less frequent: Increased amylase, pancreatitis.

Hepatobiliary disorders:

Frequent: Increased liver enzymes, hepatitis.

Renal and urinary disorders:

Frequent: Renal insufficiency, increased creatinine, proximal renal tubulopathy, renal failure, acute tubular necrosis, nephrogenic diabetes insipidus, proteinuria.

KNOWN SYMPTOMS OF OVERDOSAGE AND PARTICULARS OF ITS TREATMENT:

If overdose occurs, the patient must be monitored for evidence of toxicity, and standard supportive treatment applied as necessary.

Dolutegravir:

Management should be as clinically indicated or as recommended by the national poisons centre, where available. There is no specific treatment for an overdose of **REYDIN**. If overdose occurs, the patient should be treated supportively with appropriate monitoring as necessary. As **REYDIN** is highly bound to plasma proteins, it is unlikely that it will be significantly removed by dialysis.

Lamivudine:

Limited data are available on the consequences of ingestion of acute overdose in humans. If overdosage occurs, the patient should be monitored, and palliative supportive treatment applied as required.

Tenofovir disoproxil fumarate:
If overdose occurs, the patient must be monitored for evidence of toxicity and palliative supportive treatment be applied as necessary. Tenofovir can be removed by haemodialysis; the median haemodialysis clearance of tenofovir is 134 mL/min. The elimination of tenofovir by peritoneal dialysis has not been studied.

IDENTIFICATION:
Blue coloured, capsule shaped, biconvex film-coated tablet, debossed with 'C' on one side and plain on other side.

PRESENTATION:

**Containers of 28:**	**REYDIN** is packed in a 85 cc white HDPE bottle with 38 mm white non-child resistant cap containing 28 tablets and two silica gel bags of 2 g each, with or without a cardboard carton.
**Containers of 30:**	**REYDIN** is packed in a 85 cc white HDPE bottle with 38 mm white non-child resistant cap containing 30 tablets and two silica gel bags of 2 g each, packed in a cardboard carton.

STORAGE INSTRUCTIONS:
Store at or below 30 °C.
Keep the container tightly closed.
Keep in the original container until required for use.

KEEP OUT OF REACH OF CHILDREN.

REGISTRATION NUMBER:
52/20.2.8/0451.450

NAME AND BUSINESS ADDRESS OF THE HOLDER OF THE CERTIFICATE OF REGISTRATION:
CIPLA MEDPRO (PTY) LTD
Building 9, Parc du Cap,
Mispel Street, Bellville,
7530, RSA

DATE OF PUBLICATION OF THIS PROFESSIONAL INFORMATION:
31 August 2018

This product has been produced under licence from the Medicines Patent Pool. Any other use is not authorised.

Cipla

21073749

16.8 APPENDIX VIII. SPIRIT 2013 CHECKLIST CLINICAL TRIAL PROTOCOL

SPIRIT 2013 Checklist: Recommended items to address in a clinical trial protocol and related documents*

Section/item	Item No	Description	Addressed on page number
Administrative information			
Title	1	Descriptive title identifying the study design, population, interventions, and, if applicable, trial acronym	1, 9
Trial registration	2a	Trial identifier and registry name. If not yet registered, name of intended registry	1, 10
	2b	All items from the World Health Organization Trial Registration Data Set	10
Protocol version	3	Date and version identifier	1
Funding	4	Sources and types of financial, material, and other support	1, 30, 82
Roles and responsibilities	5a	Names, affiliations, and roles of protocol contributors	1, 9, 73
	5b	Name and contact information for the trial sponsor	1, 9
	5c	Role of study sponsor and funders, if any, in study design; collection, management, analysis, and interpretation of data; writing of the report; and the decision to submit the report for publication, including whether they will have ultimate authority over any of these activities	54
	5d	Composition, roles, and responsibilities of the coordinating centre, steering committee, endpoint adjudication committee, data management team, and other individuals or groups overseeing the trial, if applicable (see Item 21a for data monitoring committee)	75 to 77
Introduction			

Background and rationale	6a	Description of research question and justification for undertaking the trial, including summary of relevant studies (published and unpublished) examining benefits and harms for each intervention	25to 26
	6b	Explanation for choice of comparators	25
Objectives	7	Specific objectives or hypotheses	25 to 26
Trial design	8	Description of trial design including type of trial (eg, parallel group, crossover, factorial, single group), allocation ratio, and framework (eg, superiority, equivalence, noninferiority, exploratory)	26
Methods: Participants, interventions, and outcomes			
Study setting	9	Description of study settings (eg, community clinic, academic hospital) and list of countries where data will be collected. Reference to where list of study sites can be obtained	28
Eligibility criteria	10	Inclusion and exclusion criteria for participants. If applicable, eligibility criteria for study centres and individuals who will perform the interventions (eg, surgeons, psychotherapists)	29
Interventions	11a	Interventions for each group with sufficient detail to allow replication, including how and when they will be administered	29
	11b	Criteria for discontinuing or modifying allocated interventions for a given trial participant (eg, drug dose change in response to harms, participant request, or improving/worsening disease)	32
	11c	Strategies to improve adherence to intervention protocols, and any procedures for monitoring adherence (eg, drug tablet return, laboratory tests)	34
	11d	Relevant concomitant care and interventions that are permitted or prohibited during the trial	30 34
Outcomes	12	Primary, secondary, and other outcomes, including the specific measurement variable (eg, systolic blood pressure), analysis metric (eg, change from baseline, final value, time to event), method of aggregation (eg, median, proportion), and time point for each outcome. Explanation of the clinical relevance of chosen efficacy and harm outcomes is strongly recommended	36
Participant timeline	13	Time schedule of enrolment, interventions (including any run-ins and washouts), assessments, and visits for participants. A schematic diagram is highly recommended (see Figure)	37 to 41

Sample size	14	Estimated number of participants needed to achieve study objectives and how it was determined, including clinical and statistical assumptions supporting any sample size calculations	41
Recruitment	15	Strategies for achieving adequate participant enrolment to reach target sample size	42
Methods: Assignment of interventions (for controlled trials)			
Allocation:			43
Sequence generation	16a	Method of generating the allocation sequence (eg, computer-generated random numbers), and list of any factors for stratification. To reduce predictability of a random sequence, details of any planned restriction (eg, blocking) should be provided in a separate document that is unavailable to those who enrol participants or assign interventions	43
Allocation concealment mechanism	16b	Mechanism of implementing the allocation sequence (eg, central telephone; sequentially numbered, opaque, sealed envelopes), describing any steps to conceal the sequence until interventions are assigned	43
Implementation	16c	Who will generate the allocation sequence, who will enrol participants, and who will assign participants to interventions	43
Blinding (masking)	17a	Who will be blinded after assignment to interventions (eg, trial participants, care providers, outcome assessors, data analysts), and how	43
	17b	If blinded, circumstances under which unblinding is permissible, and procedure for revealing a participant’s allocated intervention during the trial	44
Methods: Data collection, management, and analysis			
Data collection methods	18a	Plans for assessment and collection of outcome, baseline, and other trial data, including any related processes to promote data quality (eg, duplicate measurements, training of assessors) and a description of study instruments (eg, questionnaires, laboratory tests) along with their reliability and validity, if known. Reference to where data collection forms can be found, if not in the protocol	44
	18b	Plans to promote participant retention and complete follow-up, including list of any outcome data to be collected for participants who discontinue or deviate from intervention protocols	42

Data management	19	Plans for data entry, coding, security, and storage, including any related processes to promote data quality (eg, double data entry; range checks for data values). Reference to where details of data management procedures can be found, if not in the protocol	47 to 48
Statistical methods	20a	Statistical methods for analysing primary and secondary outcomes. Reference to where other details of the statistical analysis plan can be found, if not in the protocol	48 to 50
	20b	Methods for any additional analyses (eg, subgroup and adjusted analyses)	49
	20c	Definition of analysis population relating to protocol non-adherence (eg, as randomised analysis), and any statistical methods to handle missing data (eg, multiple imputation)	48
Methods: Monitoring			
Data monitoring	21a	Composition of data monitoring committee (DMC); summary of its role and reporting structure; statement of whether it is independent from the sponsor and competing interests; and reference to where further details about its charter can be found, if not in the protocol. Alternatively, an explanation of why a DMC is not needed	77
	21b	Description of any interim analyses and stopping guidelines, including who will have access to these interim results and make the final decision to terminate the trial	50
Harms	22	Plans for collecting, assessing, reporting, and managing solicited and spontaneously reported adverse events and other unintended effects of trial interventions or trial conduct	51
Auditing	23	Frequency and procedures for auditing trial conduct, if any, and whether the process will be independent from investigators and the sponsor	53
Ethics and dissemination			
Research ethics approval	24	Plans for seeking research ethics committee/institutional review board (REC/IRB) approval	55
Protocol amendments	25	Plans for communicating important protocol modifications (eg, changes to eligibility criteria, outcomes, analyses) to relevant parties (eg, investigators, REC/IRBs, trial participants, trial registries, journals, regulators)	55

Consent or assent	26a	Who will obtain informed consent or assent from potential trial participants or authorised surrogates, and how (see Item 32)	56
	26b	Additional consent provisions for collection and use of participant data and biological specimens in ancillary studies, if applicable	56 and 83
Confidentiality	27	How personal information about potential and enrolled participants will be collected, shared, and maintained in order to protect confidentiality before, during, and after the trial	56
Declaration of interests	28	Financial and other competing interests for principal investigators for the overall trial and each study site	57
Access to data	29	Statement of who will have access to the final trial dataset, and disclosure of contractual agreements that limit such access for investigators	57
Ancillary and post-trial care	30	Provisions, if any, for ancillary and post-trial care, and for compensation to those who suffer harm from trial participation	59
Dissemination policy	31a	Plans for investigators and sponsor to communicate trial results to participants, healthcare professionals, the public, and other relevant groups (eg, via publication, reporting in results databases, or other data sharing arrangements), including any publication restrictions	60
	31b	Authorship eligibility guidelines and any intended use of professional writers	62
	31c	Plans, if any, for granting public access to the full protocol, participant-level dataset, and statistical code	62
Appendices			
Informed consent materials	32	Model consent form and other related documentation given to participants and authorised surrogates	Separate document
Biological specimens	33	Plans for collection, laboratory evaluation, and storage of biological specimens for genetic or molecular analysis in the current trial and for future use in ancillary studies, if applicable	14 and 38

16.9 Appendix IX. Questionnaires

The questionnaires are provided as a separate document.
